# Protocol for measuring above-threshold ionization spectra driven by few-cycle laser pulses in frustrated tunneling ionization regime

**DOI:** 10.1016/j.xpro.2026.104845

**Published:** 2026-09-23

**Authors:** Lifeng Wang, Boning Wei, Bing Yu, Wei Zhang, Peng Ye

**Affiliations:** 1 School of Optoelectronic Engineering, Xidian University, Xi’an, China; 2 Beijing National Laboratory for Condensed Matter Physics, Institute of Physics, Chinese Academy of Sciences, Beijing, China; 3 Xi’an Institute of Applied Optics, Xi’an, China; 4 Beijing Institute of Ophthalmology, Beijing Tongren Hospital, Capital Medical University, Beijing, China; 5 Songshan Lake Materials Laboratory, Dongguan, China; 6 University of Chinese Academy of Sciences, Beijing, China; 7 College of Biochemical Engineering, Beijing Union University, Beijing, China

**Keywords:** Molecular/Chemical Probes, Physics, Chemistry

## Abstract

Above-threshold ionization (ATI) spectra in the frustrated tunneling ionization (FTI) regime demonstrate control of bound-state populations via the carrier-envelope phase (CEP). Here, we present a protocol for acquiring ATI spectra from argon gas driven by few-cycle laser pulses. We describe steps of few-cycle pulse generation, CEP stabilization and control, ATI spectral measurement, and data analysis. This approach enables potential applications in isolated attosecond pulse characterization.

For complete details on the use and execution of this protocol, please refer to Wang et al.^1^

## Highlights

• Steps for measuring ATI spectra in frustrated tunneling ionization regime
• Capabilities for electric field engineering of few-cycle laser pulses
• Potential way to achieve absolute CEP measurement with single time of flight spectrometer

## Abstract (editor highlights)

Publisher’s note: Undertaking any experimental protocol requires adherence to local institutional guidelines for laboratory safety and ethics.

## Graphical abstract

**Figure:**
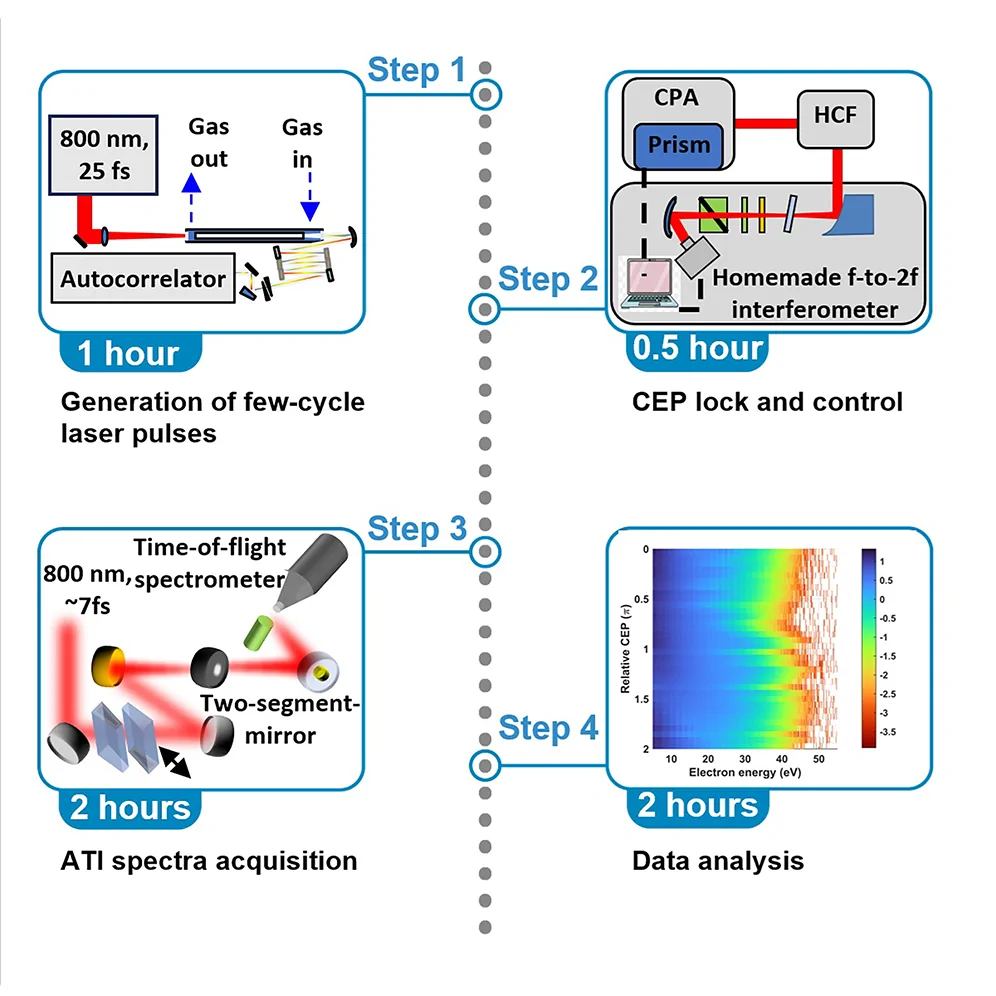

## Before you begin

Strong-field electron dynamics is the initial step for several ultrafast phenomena, such as tunneling ionization, high-harmonic generation,^2^ filamentation^3^ and isolated attosecond pulse generation.^4^ Measuring the above-threshold ionization (ATI) spectrum of electrons is a common method in ultrafast field to study electron dynamics. Wang et al., investigated ATI spectra of argon in field of XUV and infrared lasers, and proposed a semi-classical electron ionization process.^5^

In this protocol, we present an experimental approach to measure ATI spectra in the frustrated tunneling ionization (FTI) region using few-cycle pulses.^1^ This protocol details key experimental components, including the use of ultrafast lasers, spectral broadening and compression techniques, carrier-envelope phase (CEP) lock and control, ATI spectral measurement, and data analysis. The critical steps involve generation of few-cycle laser pulses, CEP lock and control, and focusing few-cycle femtosecond laser pulses into argon (Ar) gas to trigger ATI in FTI region. Controlling the incident laser intensity to ensure the ionization process occurs within the tunneling ionization regime. Before conducting experiments, please carefully read the laser safety guidelines to understand the potential hazards posed by lasers. During laser operation and maintenance, strictly adhere to the laboratory’s laser safety manual.

### Innovation

We apply a standardized protocol to measure above-threshold ionization (ATI) spectra driven by few-cycle laser pulses in the regime of FTI. Our approach allows the electric field of the few-cycle pulse to be engineered and the peak laser intensity to be accurately adjusted within the FTI regime. Unlike commonly used methods for measuring ATI spectra, our technique utilizes a two-segment mirror in vacuum to tailor the few-cycle laser pulse, shown in Figure 1. Moreover, compared with absolute CEP measurements using ATI spectra with two time-of-flight (TOF) spectrometers,^6^ our method in principle may have possibility to determine the absolute CEP with only one TOF spectrometer. However, an ambiguous absolute CEP determination would require further validation against an established CEP-measurement method, which is beyond the scope of the present work. The protocol was successfully applied in our recent experiment. For the first time, we observed a π/6 oscillation of whole ATI spectrum for relative CEP∈[0 π], to the best of our knowledge.

**Figure 1:**
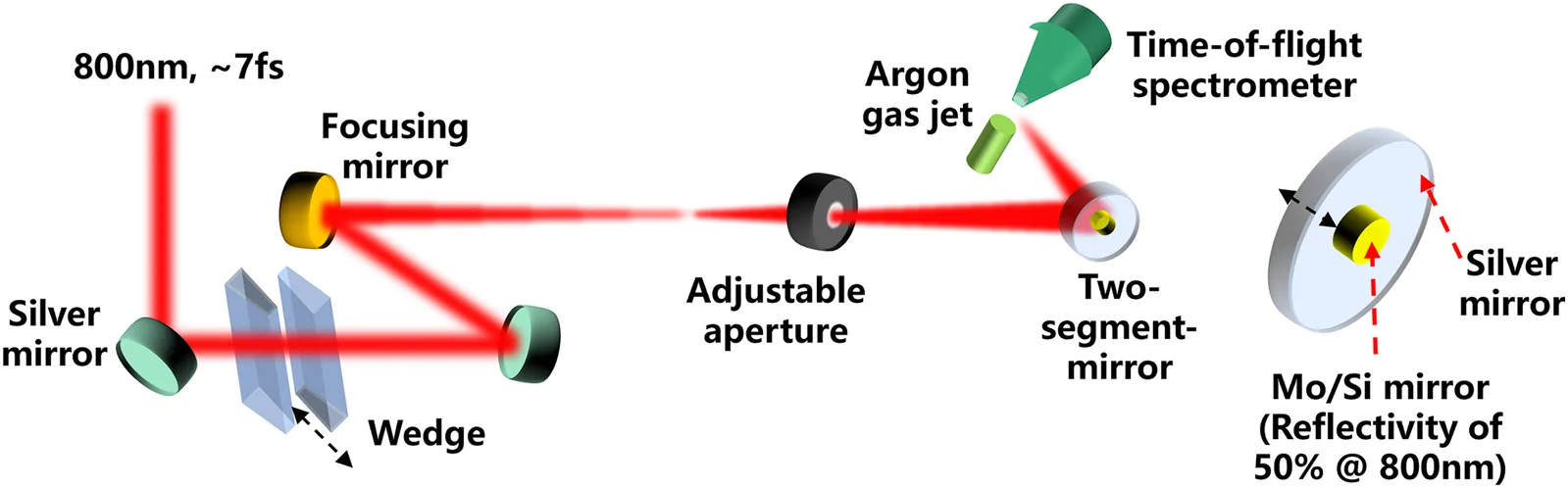
Schematic of experimental setup

### Warm up laser

**Timing: 1 h**

The femtosecond laser system (Femtopower compact PRO) delivers pulses with central wavelength of 800 nm, 0.8 mJ, 25 fs at 1kHz. The laser system is designed based on chirped pulse amplification technology and includes oscillator, stretcher, amplifier and compressor. The oscillator delivers a pulsed laser with a central wavelength of 800 nm, power of 0.5 W, pulse width of 10 fs, and repetition rate of 80 MHz. After the pulse is stretched to the picosecond level, it is injected into a multipass amplifier, which outputs a laser with a pulse energy of 1 mJ at a repetition rate of 1 kHz. The femtosecond laser then passes through a prism compressor, ultimately achieving a pulse width of 25 fs. Before performing experiments, the laser is warmed up at least one hours to deliver stable output parameters.

1. Measuring spectrum.
  a. To measure laser’s spectrum, place a block in the beamline after laser output to scatter light.
  b. Collect the scattering light into a spectrometer (OCEAN Optics USB4000).

***Note:*** This device analyzes the wavelength-dependent intensity, revealing the laser's spectral profile—including peak wavelength and bandwidth. The method provides a straightforward way to characterize laser emissions across UV to near-IR ranges.

2. Measuring power.
  a. To measure the laser’s output power, use a calibrated power meter (Thorlabs PM400) in the beam path using a stable optical mount.
  b. The PM400’s thermal power meter detects the beam, with the system pre-configured to 0.8 W range. The average power stability is less than 1% over 8 h.
  c. To make sure the mirrors are correctly adjusted, one may monitor the beam position at multiple reference points along the beam path, confirming that the beam propagates through the center of the irises/apertures.
  d. Fine tune the mirrors if there is misalignment.

**CRITICAL:** In the experiments, one need to set the wavelength at 800 nm to ensure accuracy. Measurements are performed in a controlled environment to minimize environmental noise.

## Key resources table

**Table:** 

REAGENT or RESOURCE	SOURCE	IDENTIFIER
**Other**		

CPA Laser	Femtolasers Produktions GmbH	Femtopower compact PRO
Spectrometer in visible range	OCEAN Optics	USB4000
Power meter	Thorlabs	PM400
Interferometric autocorrelator	Femtolasers Produktions GmbH	FEMTOMETER™
Time of flight spectrometer	Stefan Kaesdorf	ETF 10
Two-segment-mirror	Ultrafast Innovation	NA
Data process and analysis software	MATLAB	NA

## Step-by-step method details

### Generation of few-cycle laser pulses

**Timing: 1 h**

This section describes how to generate few-cycle laser pulses with hollow core fiber compressor. The laser pulses are used to ionize argon atoms in frustrated tunneling ionization regime.

1. Focusing laser into a hollow-core fiber.
  a. Set the beam diameter (1/e^2^) from laser system to 12 mm.
  b. Focus the laser with a focusing lens with focal length of 1.5 m and set the focal spot diameter about 190 μm at a hollow-core fiber (HCF) entrance. The RMS of the beam pointing stability is less than 5 μrad.
  c. Fix the HCF with inner diameter of 250 μm and length of 1 m in a v-groove.
  d. Use UV fused silica plates with thickness of 0.5 mm as input and output windows.
  e. Fill the HCF system with 2.5 bar neon and differentially pump the HCF by a dry pump.
  f. For safe alignment, focus the laser into HCF at low power lever (∼100 mW) without gas.
  g. Fix the HCF system by two two-dimensional stages. Place one stage at HCF’s input and place another placed at HCF’s output.
  h. Fine tune the two stages to align HCF with laser beam, and the output efficiency is usually measured at 60% and 70% at maximum in our experiment.
  i. The M^2^ of beam after HCF is smaller than 1.4.
  j. Slowly increase laser power to 0.8 W to make sure the output efficiency doesn’t change much.
2. Tuning gas pressure to have optimal spectrum broadening.
  a. Slowly increase neon gas pressure to 2.5 bar to broaden the spectrum after HCF. Meanwhile, remain a good beam profile and transmission after HCF.
  b. Once increase laser power at a setup of 0.2 W, check the output beam profile and transmission.
    ***Note:*** To avoid laser damage at HCF entrance, one must be very careful to slowly increase input laser power to 0.8 W.
  c. Fine tune the HCF to get output power of 0.4 W with broaden spectrum, as shown in Figure 2.

**Figure 2:**
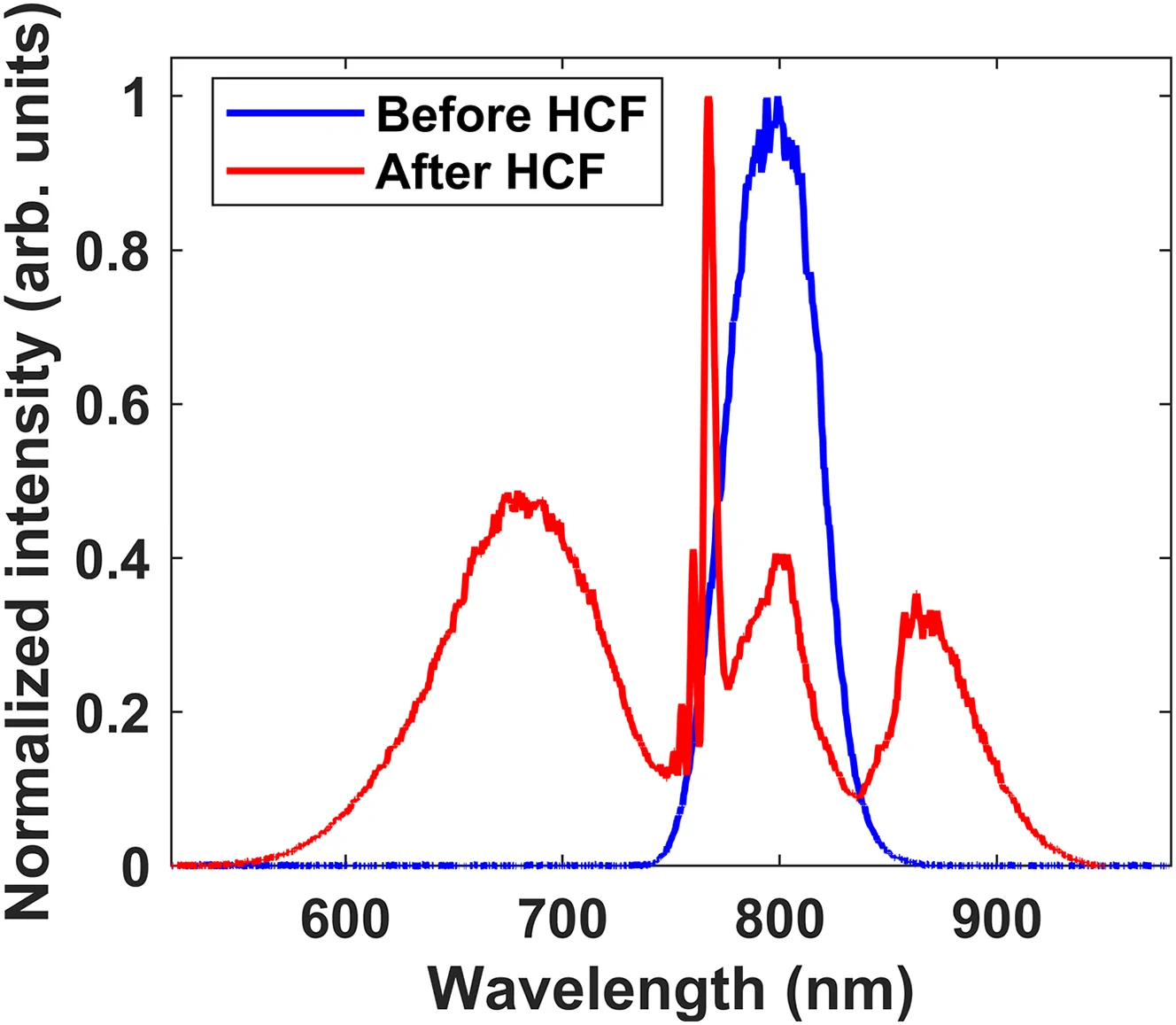
Spectra before and after broadening in hollow-core fiber
3. Compress pulse duration with chirped mirrors and wedges.
  a. Align the output laser to 8 pieces of chirped mirrors with small incident angle (<5°). The chirped mirrors provide GDD of −90 fs^2^ per bounce and the transmission is 99.5% for each mirror.
  b. Place two wedges after chirped mirrors in the beamline.
  c. Mount one wedge on a one-dimensional stage to fine tune the group delay dispersion.
  d. After the wedges, align the compressed laser pulse into interferometric autocorrelator to measure the pulse duration.
  e. To minimize the pulse duration, carefully tune the insert of the wedge.

**Figure 3:**
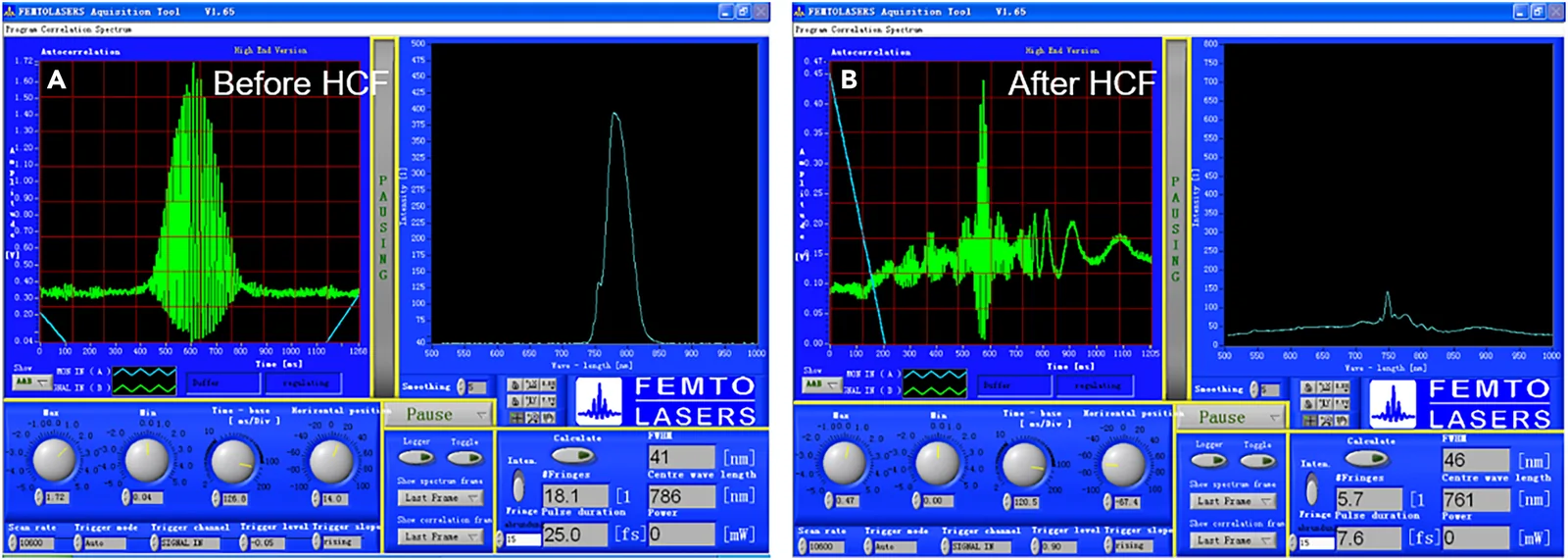
Autocorrelator measurement of the laser pulse The measurement of pulse duration before (A) and after (B) hollow-core fiber.

***Note:*** In our experiment, the wedge insertion range is less than 1 mm in the beamline. The results before and after HCF are shown in Figure 3.

### CEP lock and control

**Timing: 0.5 h**

This section describes how to lock the CEP of few-cycle laser pulses based on slow and fast loops. The CEP is key tuning parameters to generate fast oscillation in ATI spectra in frustrated tunneling ionization regime.

In our laser system, a two-stage locking system is used for feedback control to lock the CEP, as shown in Figure 4. The CEP of the output laser from oscillator is locked at 80 MHz, which is called fast loop. Then the CEP of the output laser after hollow-core fiber is locked at 1 kHz, which is called slow loop. A home-made f-to-2f spectrometer is used to extract the CEP error signal for CEP lock.

4. CEP lock in fast loop.
  a. Send laser pulses from oscillator into a 0-to-f interferometer.^7^
  b. Employ the fast-loop control scheme in the laboratory to generate a beat frequency by producing a difference frequency process in a periodically poled lithium niobate (PPLN) crystal.
  c. Feed the beam frequency back into the circuit system (Menlo system).
  d. Apply the processed feedback signal to an acousto-optic modulator (AOM).
  e. Change the voltage on the AOM to adjust the intensity of the zero-order light from the AOM (which serves as the pump light) in order to lock the CEP of the oscillator.
  f. Maximize the beat signal by tuning laser alignment to PPLN crystal.
  g. Shift the beat signal to 20 Mhz on a frequency analysis by tuning the voltage on the AOM.
  h. Turn on the locking system as long as the beat signal is higher than 35 db.
5. CEP lock in slow loop.
  a. Use a home-built f-2f interferometer to obtain the CEP signal in the slow-loop feedback circuit as shown in Figure 4.
  b. Focus the femtosecond laser into a BBO crystal to generate second-harmonic radiation.
  c. Use an optical filter to isolate the green light component (500 nm) from other spectral components.
  d. Interfere the 500 nm component from the fundamental frequency (ω) and the 500 nm component from the second harmonic (2ω) in a polarizing beam splitter (PBS).
  e. Rotate a half-wave plate (WP) to adjust the relative intensity between the two beams in order to obtain distinct interference spectra.
  f. Extract the CEP drift error by performing a Fourier transform on the interference fringes in the spectrum.
  g. Sent the CE phase error signal to a PID control algorithm, and then fed back to drive a PZT stage where a prism is mounted in the laser’s compressor. The locking bandwidth is about 10 kHz.
    ***Note:*** In our CEP locking experiment, the integration time is 50 ms. In the fast loop, the successful CEP locking is indicating by phase-discriminator signal, as shown in Figure 5. The phase-discriminator signal from the Menlo CEP stabilization system shows a large periodic excursion before locking [Figure 5A]. After the feedback loop is engaged, the discriminator signal is stabilized close to zero and remains within the feedback range without cycle slips or abrupt phase jumps [Figure 5B].

**Figure 5:**
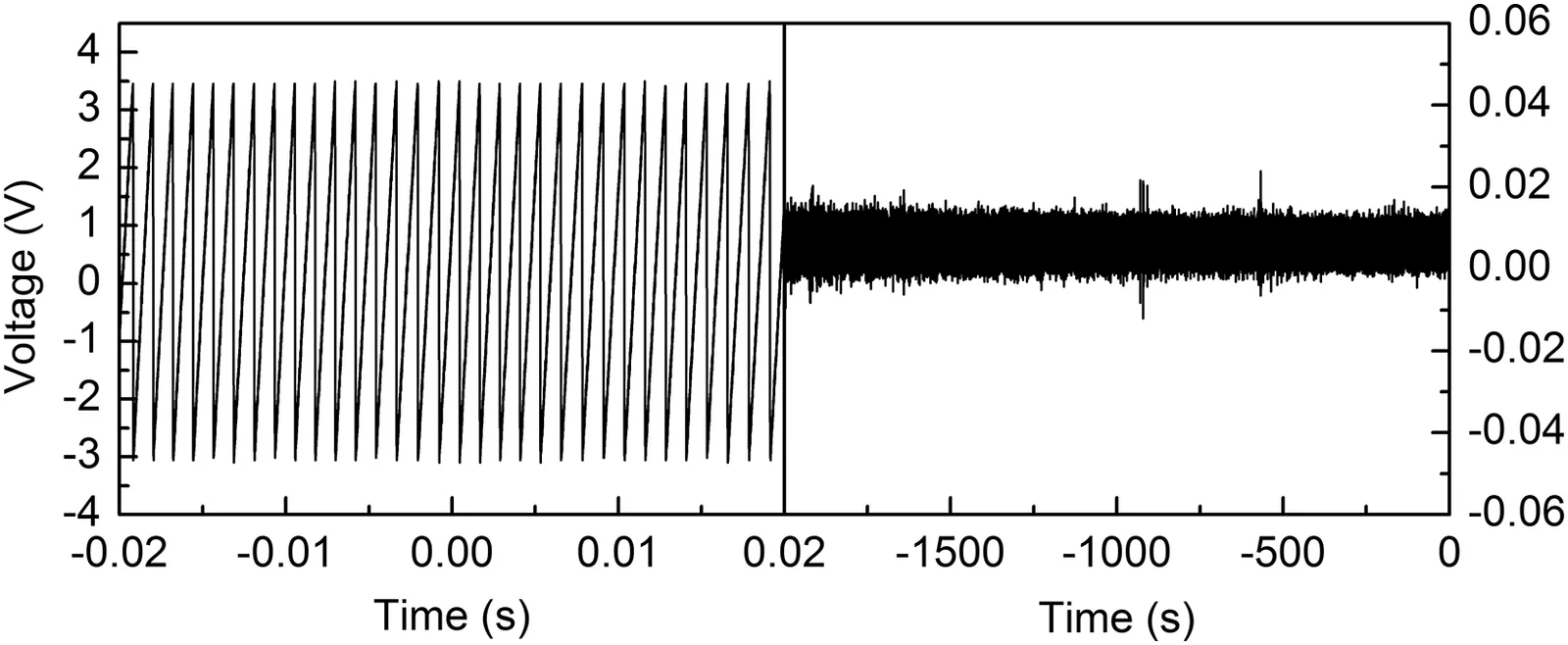
Phase-discriminator signal Phase-discriminator signal before (Left) and after (Right) locking.
  h. Employ a spectrometer in the slow loop to measure the interference fringes, as shown in Figure 6A.

**Figure 6:**
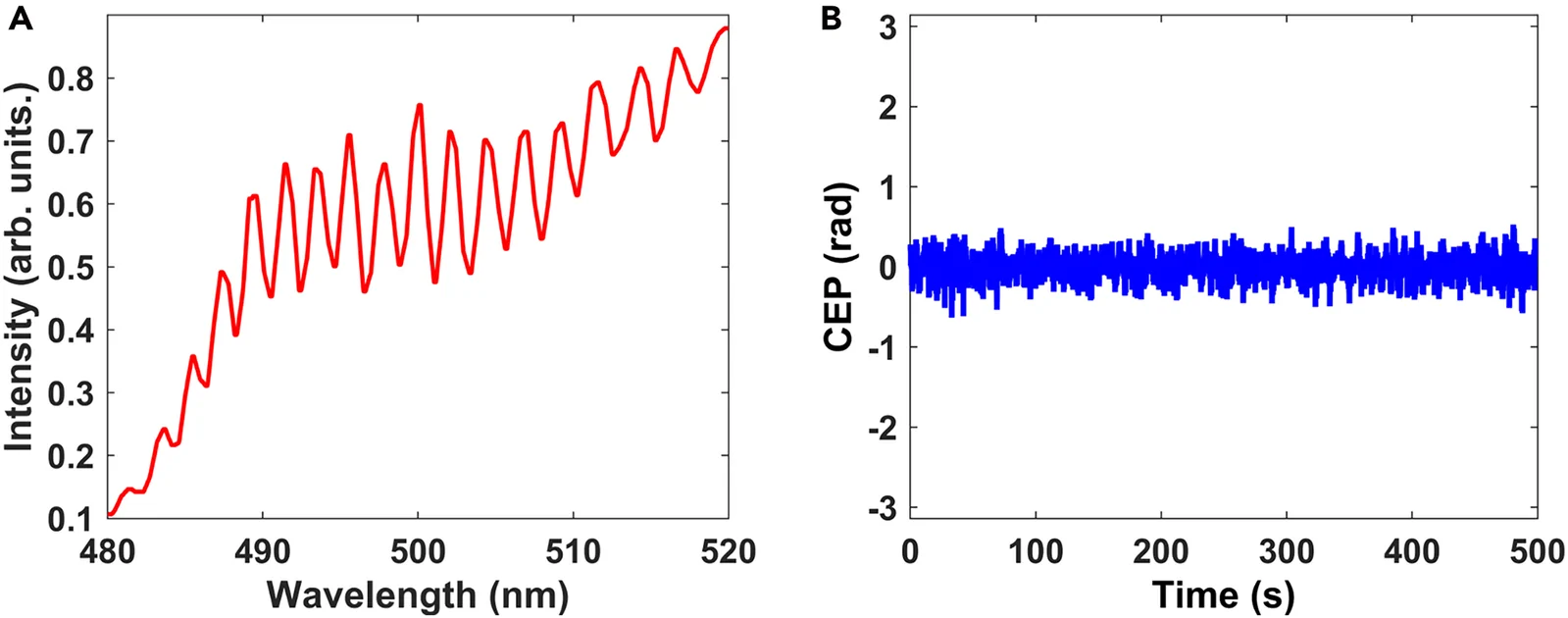
CEP Measurement Fringes (A) and CEP lock (B) after hollow-core fiber in the slow loop.
  i. Tune the insert of the prism to stabilize the CEP after hollow core fiber, as shown in Figure 6B.^8^ Before locking the CEP jitter is 677.1 mrad and 53.1 mrad over 360 s for short-term control, indicating a successful CEP stabilization.
    ***Note:*** Based on our scheme, the CEP is long-term controlled with 85 mrad phase error (RMS) over 7 h and the typical lock duration is 3 h.^8^ The locked CEP is relative CEP.
6. CEP control with wedges.
  a. Use two fused silica wedges after chirped mirrors to control the CEP in our experiments.
    ***Note:*** The size of the wedge is 20×30 mm^2^ with small wedge angle of 2.8°. The thickness of 0.2 mm at thinner side. There are two objectives of these two wedges, one is to compress the few-cycle laser pulse and another one is to control the CEP.
  b. Mount one wedge on a picomotor stage.
  c. Fine tune the insert of the wedge to control the CEP.
    ***Note:*** Theoretically, varying the thickness of the wedge by 57.86 μm induces a 2π shift in the CEP of 800 nm laser pulses. Experimentally, we have calibrated the CEP step according to HHG spectra.^9^ During the calibration, the fused-silica wedge was inserted stepwise, and the corresponding HHG spectrum was recorded at each insertion position. Because the HHG spectrum is periodic with respect to CEP, the spectral modulation pattern recovered its original shape after a CEP shift of π. From this periodicity, the wedge-insertion-to-CEP conversion factor was determined to be approximately π/17 per calibration step.

**Figure 4:**
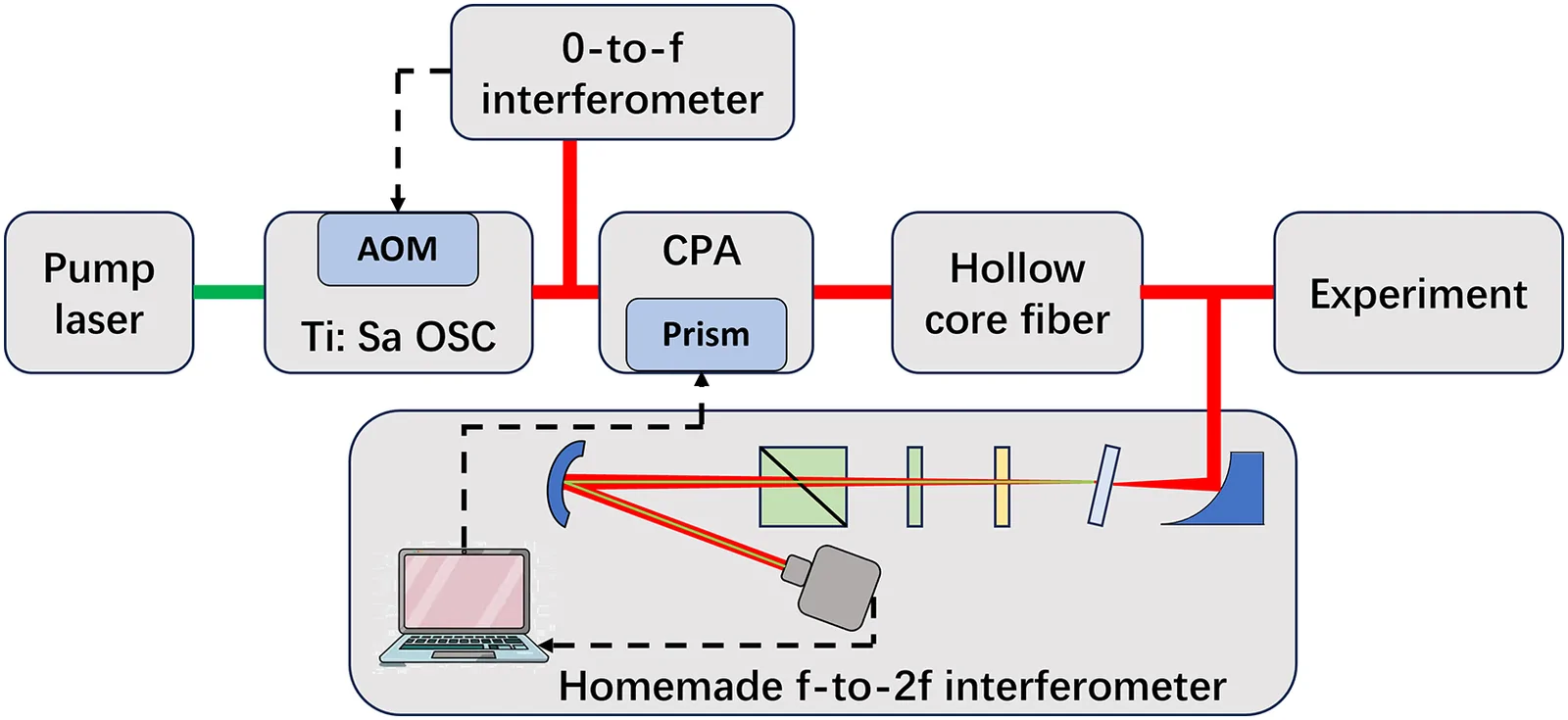
Schematic of CEP lock in fast and slow loop

### ATI spectra acquisition

**Timing: 2 h**

This section describes how to experimentally get ATI spectra in terms of CEP in frustrated tunneling ionization regime.

ATI spectra are generated by the interaction between intense femtosecond laser and gases.

7. Focusing laser into a gas jet.
  a. Focus the linearly polarized laser beam with energy of 0.4 mJ and 7 fs into a vacuum chamber by a concave mirror with 500 mm focal length.
  b. Set a motorized aperture to control the laser power, which is important in our experiment.
    ***Note:*** We control the IR peak intensity by tuning the motorized aperture in the frustrated tunneling ionization regime. In our experiment, the beam waist at the argon gas jet is approximately 25 μm. The IR intensity is estimated around 1.9×10^14^ W/cm^2^, and the corresponding ponderomotive energy is 10.2 eV and Keldysh parameter is 0.89. For a pulse of 7.8 fs, the ionization probability is about 0.1% at pulse peak and 2% after pulse propagation based on ADK model.^10^
    ***Note:*** The motorized aperture does not only control the transmitted pulse energy, but also the beam size and spatial mode before focusing. The calculated beam profiles and focus intensity are shown in Figures S2 and S3 in supplemental information.
  c. Send the IR beam into next chamber with backing vacuum pressure of 10^−7^ mbar.
  d. Focus the laser by a two-segment-mirror which is assembled by an inner Mo/Si multilayer mirror and an outer silver mirror with the same focal length of 125 mm.
  e. Fix the delay of the two IR laser pulses after the two-segment-mirror.
  f. Inject argon atoms by a gas jet to interact with IR laser in the range of focus to trigger above-threshold ionization (ATI) process.
  g. Record the photoelectron spectrum for ATI by tuning the CEP of input few-cycle IR laser with a TOF spectrometer.
8. Obtaining an ATI spectrum in terms of CEP.
  a. Use a TOF spectrometer to measure the kinetic energy of electrons in our experiments.
    ***Note:*** The laboratory's TOF energy spectrometer was procured from Stefan Kaesdorf GmbH (Germany) with model of ETF10. The principle of the TOF spectrometer is based on the fact that electrons with different energies have different velocities, resulting in distinct flight times when traveling through a vacuum tube of identical length. By measuring these flight times, the electron energy spectrum can be inversely derived. The total length of TOF is 57.3 cm.
  b. Set the extraction voltage at 2.3 keV in our experiment.
  c. Before acquiring ATI spectrum, turn on the vacuum pump until the vacuum level in TOF chamber at 10^−8^ mbar level.
  d. Apply lens voltage on TOF at 300 V across both ends of the vacuum tube to enhance count of electrons during operation.
  e. At a fixed CEP, generally control the argon gas pressure less than level of 10^−5^ mbar to make sure no space charge effect exists. Set the record ATI spectrum with integration time of 30 s.
    ***Note:*** In our experiment, the background of electron counts is kept very low, typical number is 2–5 counts. Therefore, there is no need to subtract background in data process.
  f. Tune the picomotor to change the CEP, then repeat step d to record ATI spectrum in terms of CEP.

### Data analysis

**Timing: 2 h**

This section describes how to process the relationship between experimentally obtained ATI spectra and CEP, and analyzes the fast oscillations in the spectra.

9. Getting ATI spectrogram vs CEP.
  a. The horizontal axis of the measured raw spectrum represents time of flight, and the vertical axis represents electron counts.
  b. In our TOF spectrometer, define the time zero by the signal produced by an optical pulse upon detection following its traversal of the full TOF drift region.
    ***Note:*** In this experiment, the “zero point” is around 30 ns, as shown in Figure S3 in the supplemental information.
  c. To calculate signal-to-noise ratio, use data in Figure S3 in the supplemental information. The noise is around 3 counts, and signal is around 40 counts, therefore the signal-to-noise ratio is 38/3=12.3, or 10.9 dB.
    ***Note:*** There is no background subtraction since SNR is high.
  d. To convert to the energy domain—where the horizontal axis is energy and the vertical axis remains electron counts—two aspects of calibration are required.
  e. For the horizontal axis, use the time-energy calibration curve provided by the manufacturer.
  f. For the vertical axis, to ensure the same number of electrons is maintained when transitioning from the time domain to the energy domain, the relationship shown in Equation applies: g(E) = f(t) × dt/dE ∝ f(t)t^3^, where f(t) and g(E) are the electron counts in time and energy domain, respectively. The calibration curve is shown in Figure 7.
    ***Note:*** The calibration data is provided as supplemental information. Column 1 lists the electron energy in eV; Column 2, the time-of-flight in microseconds (μs); and Column 3, the scaling factor for electron counts in the energy domain.

**Figure 7:**
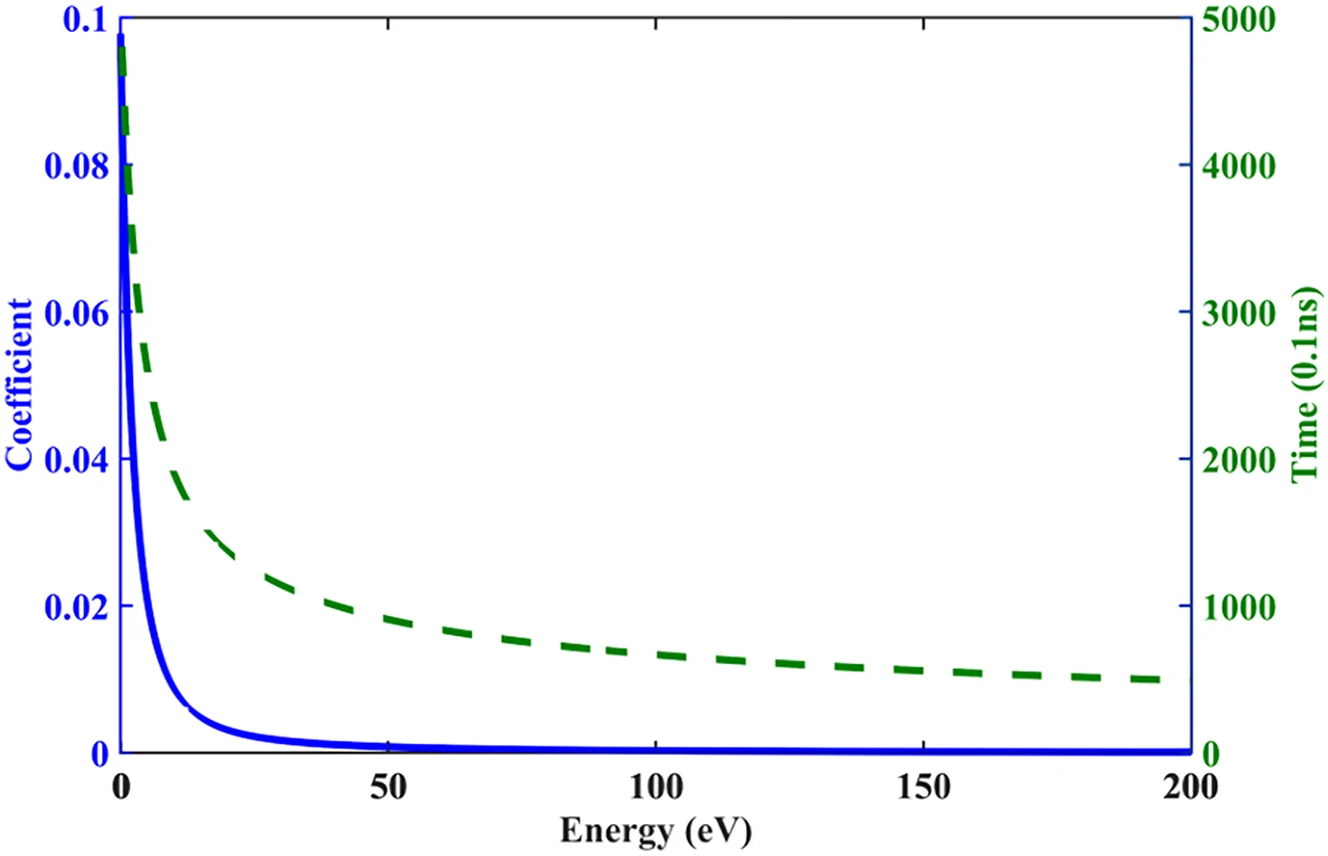
Calibration curves for ATI spectrum measured by TOF spectrometer
  g. At a fixed CEP, calibrate the original ATI spectrum in time domain into the energy domain.
  h. In frustrated tunneling ionization regime, a represented TOF trace is shown in Figure 8A. The x axis represents numbers and y axis represents electron counts. The original data in the inset is electron counts in range of 1200 to 2200.

**Figure 8:**
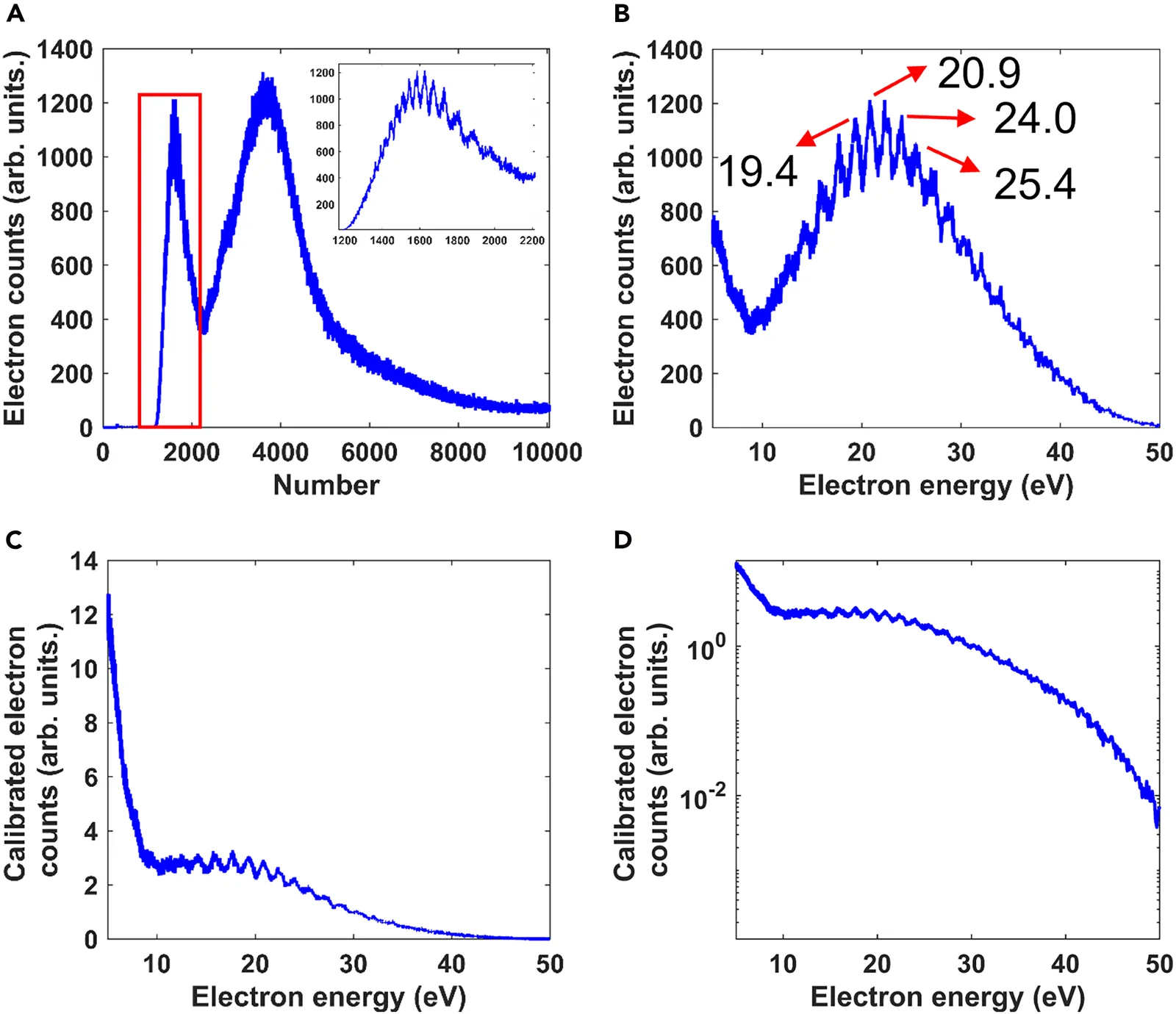
Data process of a row TOF trace (A) Raw data of the TOF trace. (B) TOF trace after Calibration of x axis. (C) Linear scale TOF Trace After calibration of y axis. (D) Logarithm scale TOF Trace After calibration of y axis.
  i. After calibration of x axis, show the result in Figure 8B.
  j. Find clearly peaks in range around 20–30 eV.
    ***Note:*** Typical peak values are labels, which is almost integer multiples of the driving laser’s photon energy. These results indicate that there is no strong space charge effect in our measurement.
  k. After calibration of y axis, transform the electron counts into energy domain and the results are shown in linear and logarithm scale in Figures 8C and 8D, respectively.
  l. Repeat this calibration process at different CEPs.
  m. Plot all ATI spectrum in a single graph in logarithm scale for better visualization of the CEP effect, where the horizontal axes represent CEP and electron energy, the vertical axis represents electron counts, and colour is used to denote the magnitude of the counts.
    ***Note:*** During data processing, MATLAB's built-in piecewise interpolation function (interp1) is used to apply the calibration curve to the TOF measurement data. MATLAB code is provided as supplemental information.

## Expected outcomes

By varying the CEP of the output ultrashort pulses, the relationship between the ATI electron spectra and the CEP can be measured. Consequently, one can observe how the electron spectra generated by ultrashort pulses change in terms of different CEPs, as shown in Figure 9. The calibrated electron counts are in range of 0 to 10 in our experiment, the corresponding range of colour bar is −4 to 1. If the peak intensity is not right, one may observe ATI spectrum with periodicity of π, as shown in Figure S4 in the supplemental information. Then the peak intensity should be tuned by size of iris or laser’s parameters (such as power and pulse duration) to repeat experimental procedure. Alternatively, the variation of photoelectrons at a specific energy or within a certain energy range with respect to the CEP can be analysed. Combined with theoretical models, this approach enables the investigation of ultrafast electron dynamics processes.^1^

**Figure 9:**
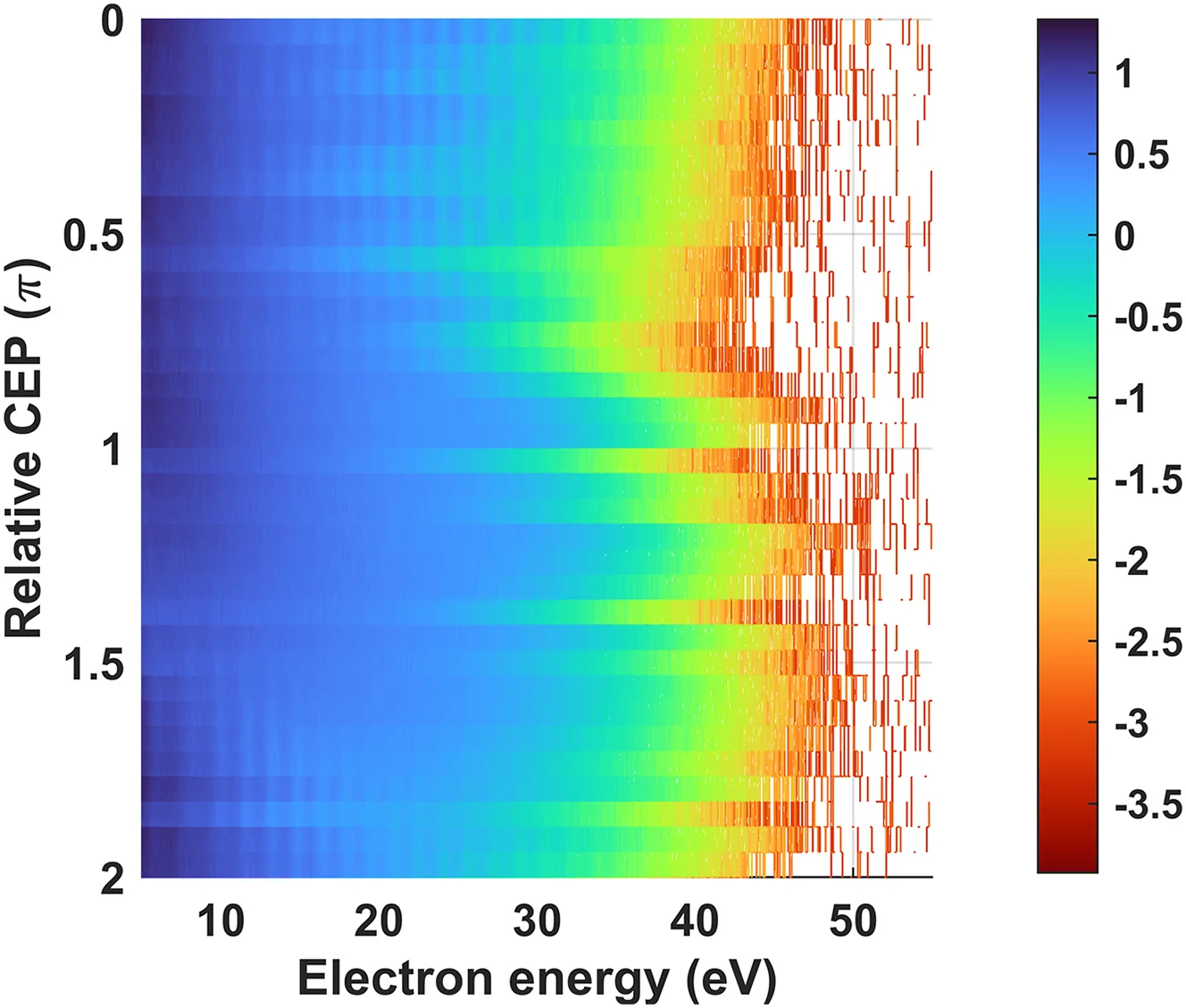
ATI spectrum in logarithm scale measured by TOF spectrometer vs relative CEP

On the other hand, this setup can also be used for the pulse duration measurement of attosecond pulses with attosecond streaking spectroscopy. In this experiment, gas (such as neon, helium, etc.) is injected at the first focal position to generate isolated attosecond pulse in XUV range. Then the XUV pulse beam is collinearly propagated with IR beam and a metal filter is used to filter out the XUV beam in the center and the IR beam in the outsize. In the second vacuum chamber, the XUV beam is focused by the inner Mo/Si mirror and the IR beam is focused by the outer silver annual mirror. The IR intensity at the gas jet is controlled at the level of 10^12^ W/cm^2^ so that no electron is generated by IR itself. The delay of the XUV and IR is controlled and tuned by the inner Mo/Si multilayer mirror on a piezo stage, facilitating an attosecond streak camera experiment to measure single attosecond pulses with durations on the order of hundreds of attoseconds.^4^

## Limitations

Theoretical simulations indicate that our experimental results are highly sensitive to key parameters such as laser field strength and carrier-envelope phase. There are several experimental parameters may affect the outcomes. Firstly, precise control of peak power needs to be achieved through fine adjustment of the aperture. Although a vacuum electric aperture is used in this experiment, limited by the mechanical design accuracy, each time the same aperture size is set, pulse energy jitter is still introduced. Secondly, laser’s beam profile after hollow-core fiber may affect the beam quality at focus and peak intensity. Lastly, due to the use of differential pumping for spectral broadening, fluctuations in gas flow, ambient temperature drift, and mechanical vibrations cause certain changes in laser pulse duration, which in turn lead to changes in peak power in the argon medium. Eventually, the experimental conditions may deviate from the optimal region of frustrated tunneling ionization. These factors collectively constitute the main limitations of the current experimental setup and procedures.

## Troubleshooting

### Problem 1

Unstable HCF broadening after hollow-core fiber (related to Step 2a). Possible reason is unstable laser beam pointing before HCF system.

### Potential solution

Realign the laser beam into beam pointing stabilization system and restart the system.

### Problem 2

Low power of lasers after hollow-core fiber (related to Step 2c). One possible is the beam size at entrance of hollow core fiber is not 0.64a, where a is the inner diameter of hollow-core fiber, so that the transmission of laser is not maximized.^11^ Another one is the entrance of hollow core fiber is damaged by femtosecond laser.

### Potential solution

For the first one, a potential solution is changing a suitable focal length to modify the focal spot size at entrance of hollow-core fiber to maximize the output laser power. For the second one, a potential solution is changing a new hollow-core fiber.

### Problem 3

Pulse duration of lasers after hollow-core fiber is not well compressed to few-cycle regime or drift. (related to Step 3d). One possible reason is a leakage in hollow core fiber compressor. Another one is the group delay dispersion of chirped mirrors is not suitable.

### Potential solution

For the first one, one may need to find the leakage in the hollow core fiber compressor with leakage detector. After the leakage is fixed, optimize the gas pressure to get a broad spectrum, usually covers 550–950 nm. For the second one, a potential solution is changing the number of the chirped mirrors. Minimize the pulse duration by checking the autocorrelator trace.

### Problem 4

CEP locking is lost in the fast loop (related to Step 4c). Possible reason is too weak beat signal measured by Menlo system.

### Potential solution

Firstly, optimize the beat signal as strong as possible (>35 db) by carefully align laser into PPLN crystal. Secondly, change the voltage on AOM to make sure the beat signal goes to 20 MHz on frequency analysis. Lastly, turn on the Menlo system to lock the CEP again.

### Problem 5

Unable CEP fringes in slow loop (related to Step 5e). Possible reason is that the SHG signal in f-2f system is not strong enough.

### Potential solution

Tune laser alignment and rotate SHG crystal to improve fringes signal.

### Problem 6

CEP locking is lost in the slow loop (related to Step 5g). Possible reason is that the fringes measured by f-2f system has low contrast.

### Potential solution

The CEP in the slow loop is detected by a home-made locking system. One may check the fringes in the f-2f system. If the fringes are not clear, one may need to tune the parabolic mirror in the f-2f system to maximize the contrast of the fringes.

### Problem 7

Gas jet misalignment (related to Step 7i). Possible reason is long term vibration in the laboratory.

### Potential solution

Move gas jet and check ATI signal at the same time, optimize gas jet position with increasing ATI counts.

### Problem 8

Poor vacuum recovery (related to Step 8c). Possible reason is leakage.

### Potential solution

One may need to find the leakage with leakage detector. Check chambers one by one.

### Problem 9

Cannot measure an ATI spectrum (related to Step 8d). Possible reasons are wrong voltage on the TOF or laser alignment with gas jet.

### Potential solution

Firstly, check the voltage on the TOF, if the voltage is not applied on TOF, it may reduce the counts of electron significantly. Secondly, check the alignment of gas jet and laser’s focus. Setup an image system to image the input laser’s focus. When the gas jet is inserted into laser beam, the image became blurred. Move the gas jet until the image became clear so that the overlap between gas jet and laser focus is OK.

### Problem 10

Space-charge effect (related to Step 8e). Possible reason is IR intensity is too strong to generate too much electrons.

### Potential solution

In the experiment, we slowly increase IR intensity to suppress space-charge effect. One way to check there is no strong space-charge effect is to check the peak positions of ATI spectra, as shown in Figure 8.

### Problem 11

Weak ATI signal despite correct TOF voltage (related to Step 9c). Possible reason is IR intensity is not strong enough.

### Potential solution

Check laser power and pulse duration and increase laser power or optimize insert of wedge to improve ATI signal.

### Problem 12

Distorted energy calibration (related to Step 9e). Possible reasons are unverified TOF calibration or space-charge effect.

### Potential solution

The TOF calibration should be verified before the ATI measurement. In our procedure, the high-harmonic photon spectrum is first measured and used as a reference for calibrating the corresponding photoelectron peaks in the TOF spectrum. After the TOF-energy calibration is confirmed, the same TOF voltage configuration and acquisition settings are kept unchanged for the CEP-dependent ATI measurement. If the converted energy spectrum shows shifted, compressed, or distorted ATI features, the user should recheck the time-zero definition, TOF voltage settings, calibration curve, and background subtraction. For space-charge effect, please check problem 10.

## Resource availability

### Lead contact

Further information and requests for resources should be directed to and will be fulfilled by the lead contact, Peng Ye (ye.peng@iphy.ac.cn).

### Technical contact

Further information and requests for technical issues should be directed to and will be fulfilled by the technical contact, Lifeng Wang (wanglifeng@xidian.edu.cn).

### Materials availability

All materials used in this experiment, including optics and noble gases, are easy to access in the market. No unique materials are generated in this study.

### Data and code availability

Data and code underlying the findings of this study are available from the corresponding author upon reasonable request.

## Acknowledgments

The work is financially supported by the 10.13039/501100001809National Natural Science Foundation of China (nos. 62475172 and 12574476), ShenZhen Science and Technology Program (grant no. JCYJ20250604183851069), the CAS Project for Young Scientists in Basic Research (YSBR-143), CAS project (no. 2024000052), 10.13039/100022814Songshan Lake Materials Laboratory (grant no. 24D1221LS11), and the Synergic Extreme Condition User Facility.

## Author contributions

L.W. conceived the study and led the project. L.W. and P.Y. performed the experiments and prepared the figures. L.W. and B.W. drafted the manuscript. All authors critically reviewed and revised the manuscript.

## Declaration of interests

The authors declare no competing interests.

## Supplemental information

Document S1. Figures S1–S4

Data S1. Calibration curve (related to step9): calibrate raw TOF data into X axis (Time) and Y axis (Counts)

Data S2. MATLAB code for ATI spectra data analysis, related to Step9
