# Supplementary material for: Protocol for measuring above-threshold ionization spectra driven by few-cycle laser pulses in frustrated tunneling ionization regime
Source: STAR Protoc. 2026 Sep 23;7(4):104845. doi: 10.1016/j.xpro.2026.104845 (PMC13632067; doi:10.1016/j.xpro.2026.104845)
Supplement: Supplementary file 1 [file mmc1.pdf]

Supplemental Materials

S1. Calculated beam profiles and focus intensity in the setup

The calculated the beam profiles behind the aperture are shown in Fig. S1 (b), on the two-segment mirror in (c) and at the focus (d). The focus intensity was calculated by considering the transmitted pulse energy, the beam size after the aperture, and the focusing geometry. The resulting dependence of the estimated average intensity on the motorized-aperture diameter is shown in Fig. S2. This calibration defines the practical operating window used in the ATI/FTI measurements.

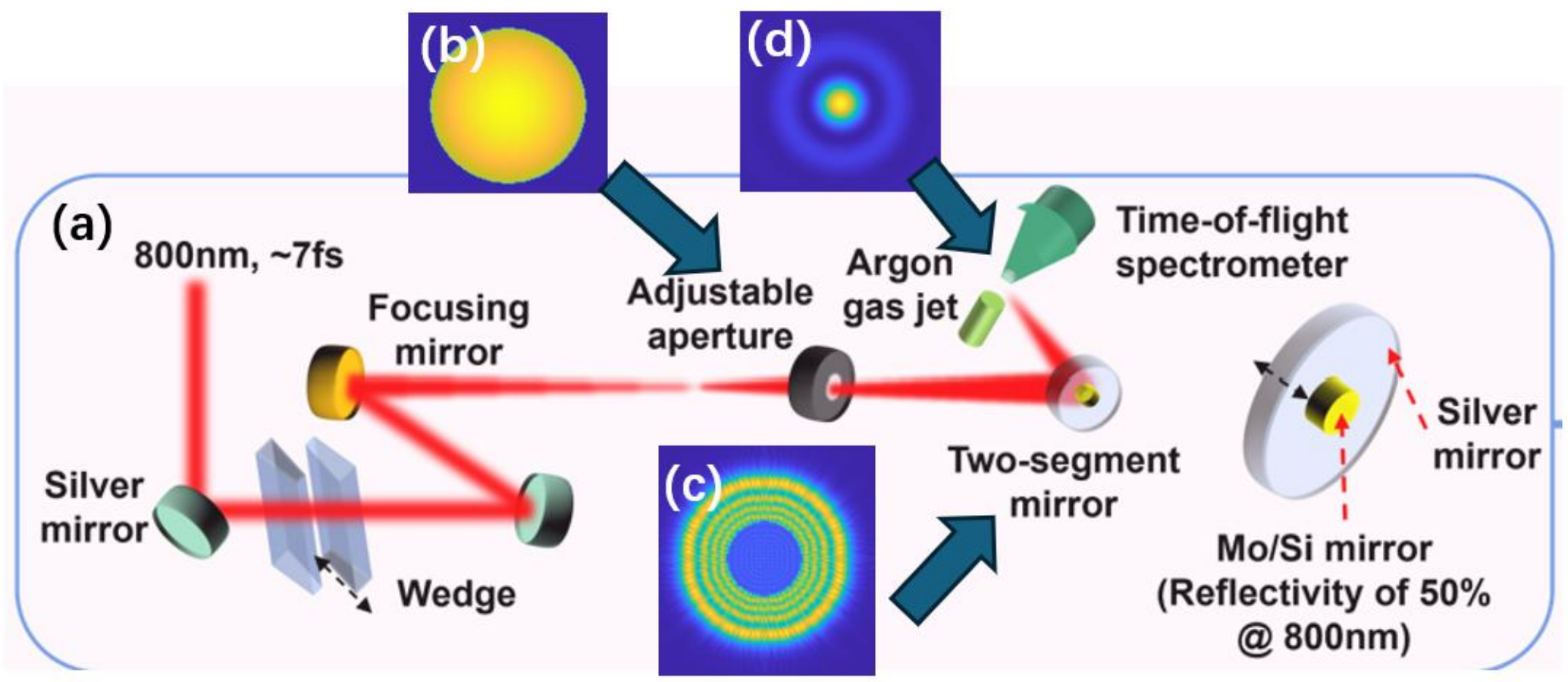

Fig.S1 (a) The schematic beam path. The transmitted beam after the aperture (b), on the two-segment mirror (c) and at focus (d).

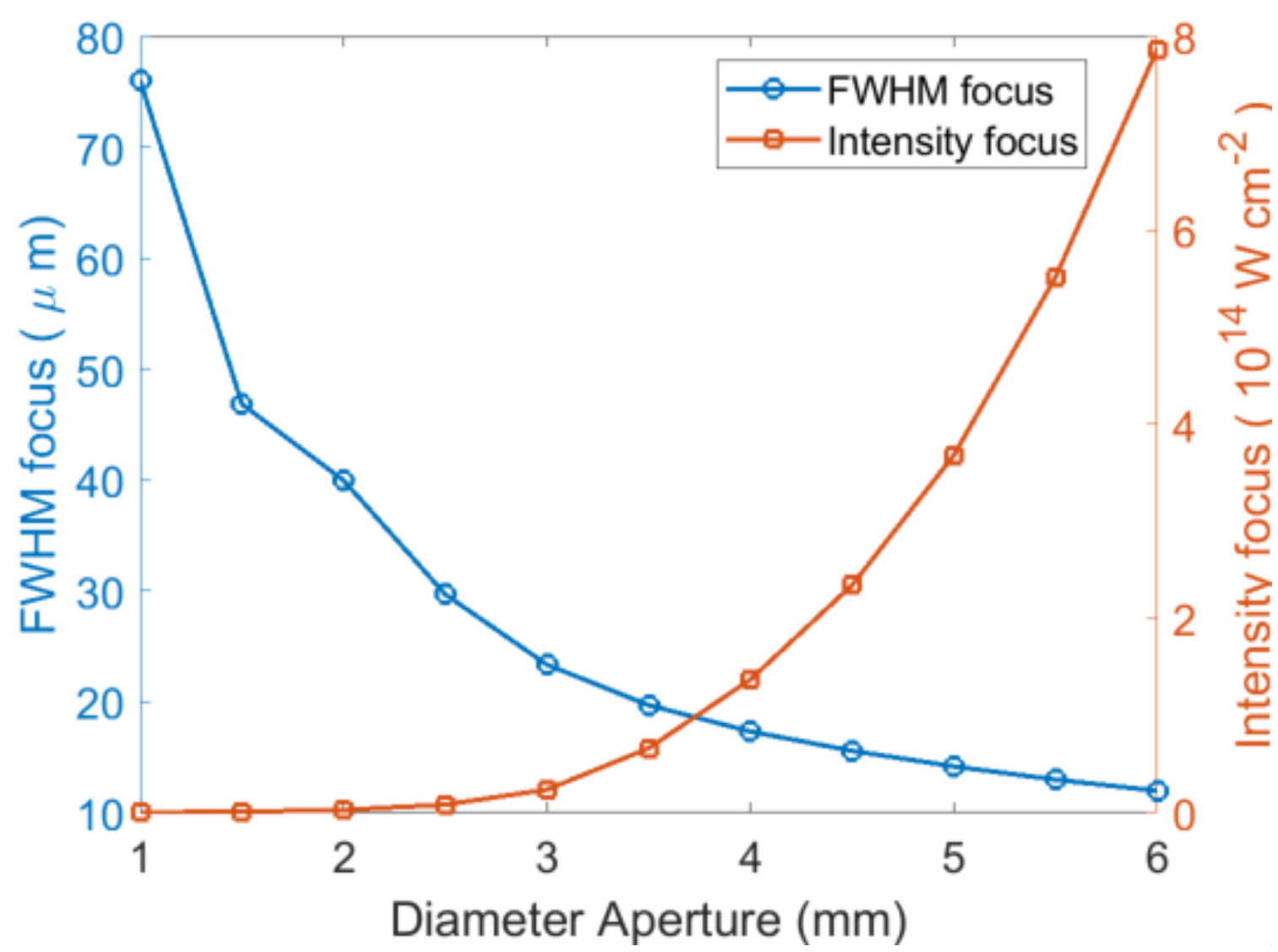

Fig. S2 The focus intensity at focus changes with the diameter of the motorized aperture.

S2. “Zero point” of TOF spectrometer

In a typical ATI spectrum, the sharp peak appearing at approximately 30 ns originates from the scattered light rather than from photoelectrons, and was therefore used as the temporal reference.

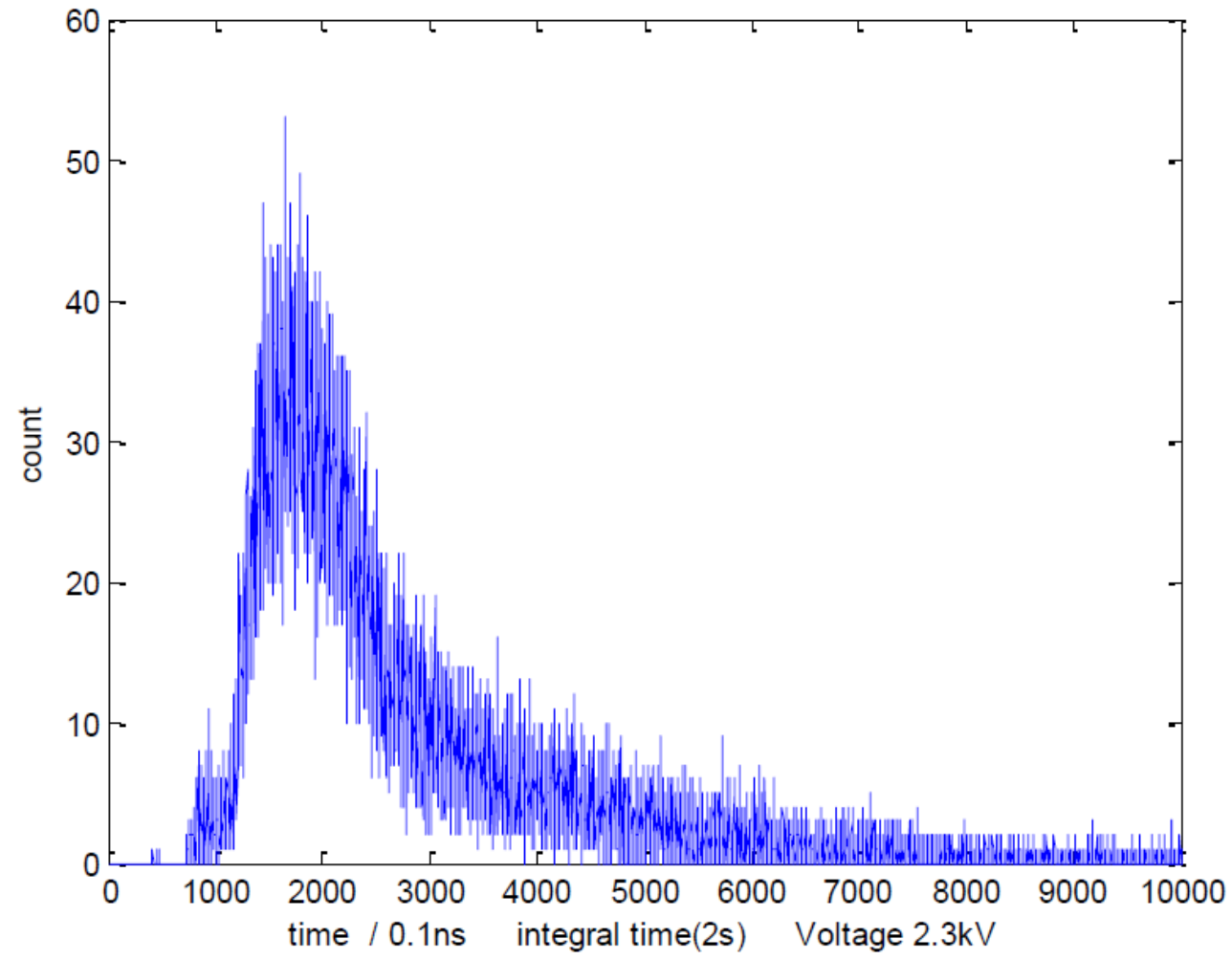

Fig. S3. Typical ATI spectra ionized by IR laser with “zero point” around 30 ns.

S3. ATI spectra with periodicity of π.

A representative CEP-dependent ATI spectrum showing the expected π -periodic modulation with CEP.

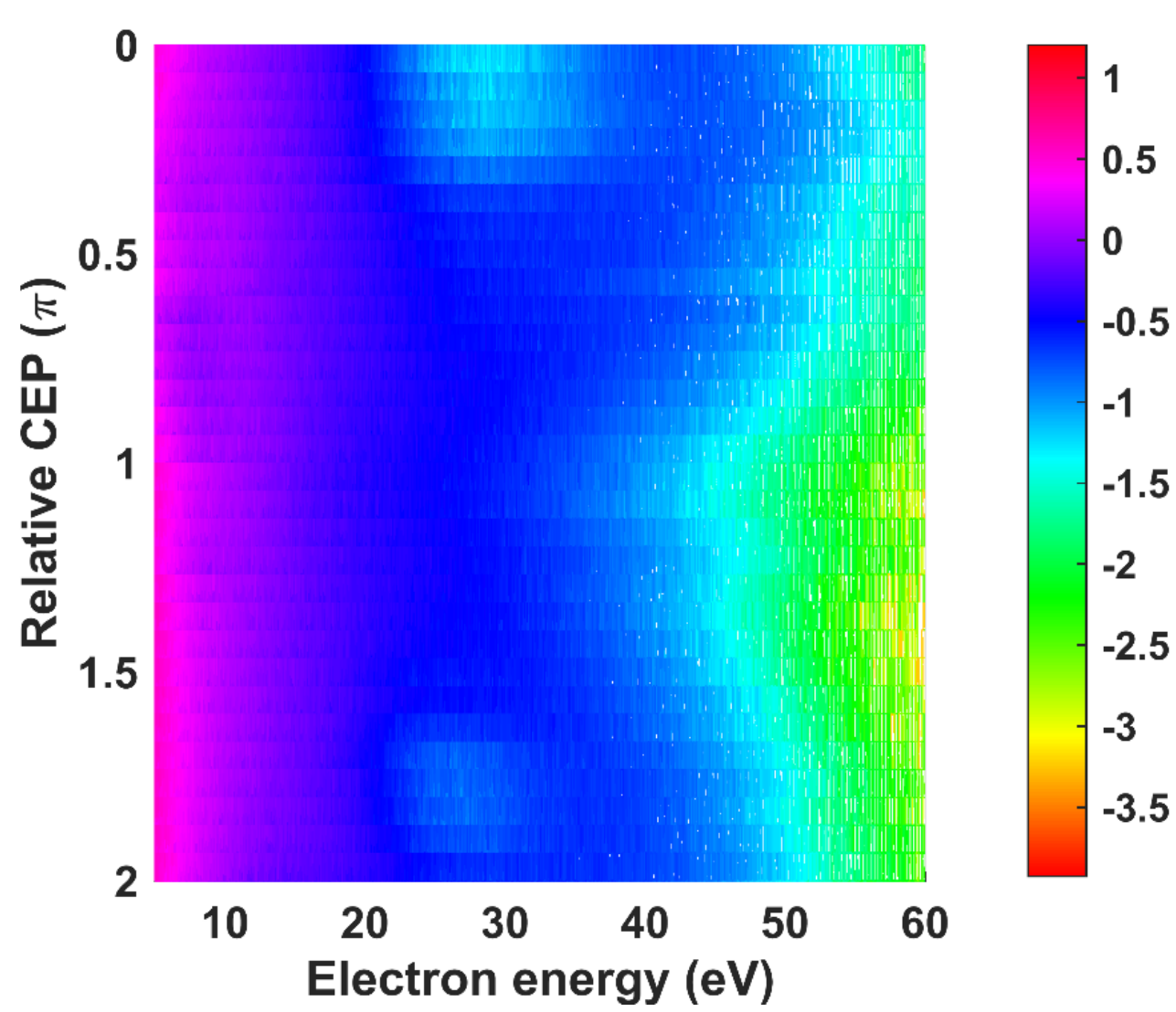

Fig. S4. Typical ATI spectrum in logarithm scale ionized by IR laser with periodicity of π.
