# Supplementary material for: Protocol for measuring above-threshold ionization spectra driven by few-cycle laser pulses in frustrated tunneling ionization regime
Source: STAR Protoc. 2026 Sep 23;7(4):104845. doi: 10.1016/j.xpro.2026.104845 (PMC13632067; doi:10.1016/j.xpro.2026.104845)
Supplement: Supplementary file 2 [file mmc2.pdf]

Calibration curve (related to step9): calibrate raw TOF data into X axis (Time) and Y axis (Counts)

0 0.480387725418098 0.110860212343916
0.02000200020002 0.478449170580721 0.109523526279056
0.04000400040004 0.476528471572243 0.108209791887435
0.06000600060006 0.474625383501464 0.106918506385948
0.08000800080008 0.472739665933061 0.10564918031203
0.1000100010001 0.470871082786694 0.104401337110869
0.12001200120012 0.46901940223885 0.103174512737247
0.14001400140014 0.467184396627328 0.101968255271407
0.16001600160016 0.465365842358285 0.100782124548405
0.18001800180018 0.463563519815757 0.0996156918004157
0.2000200020002 0.461777213273594 0.0984685393114701
0.22002200220022 0.460006710809715 0.0973402600841554
0.24002400240024 0.458251804222624 0.0962304575178026
0.26002600260026 0.456512288950119 0.0951387450977176
0.28002800280028 0.454787963990118 0.0940647460950323
0.3000300030003 0.453078631823544 0.093008093276767
0.32003200320032 0.45138409833921 0.0919684286257135
0.34003400340034 0.449704172760633 0.0909454030697653
0.36003600360036 0.448038667574728 0.089938676220336
0.38003800380038 0.44638739846233 0.0889479161195253
0.4000400040004 0.444750184230476 0.0879727989957011
0.42004200420042 0.443126846746404 0.087013009027185
0.44004400440044 0.44151721087322 0.0860682381137383
0.46004600460046 0.43992110440718 0.0851381856555581
0.48004800480048 0.438338358016539 0.0842225583395094
0.5000500050005 0.436768805181925 0.0833210699323221
0.52005200520052 0.435212282138195 0.0824334410805028
0.54005400540054 0.433668627817723 0.0815593991167131
0.56005600560056 0.432137683795084 0.0806986778723796
0.58005800580058 0.430619294233098 0.0798510174963125
0.6000600060006 0.429113305830188 0.0790161642791132
0.62006200620062 0.427619567769014 0.078193870483166
0.64006400640064 0.42613793166636 0.0773838941780119
0.66006600660066 0.424668251524217 0.076585999080914
0.68006800680068 0.423210383682053 0.0757999544024315
0.7000700070007 0.421764186770215 0.075025534696823
0.72007200720072 0.420329521664449 0.0742625197171117
0.74007400740074 0.418906251441491 0.0735106942746468
0.76007600760076 0.417494241335718 0.0727698481030075
0.78007800780078 0.416093358696807 0.0720397757260939

0.8000800080008 0.414703472948401 0.0713202763302659
0.82008200820082 0.413324455547727 0.0706111536403842
0.84008400840084 0.411956179946166 0.0699122157996254
0.86008600860086 0.410598521550727 0.0692232752529363
0.88008800880088 0.409251357686418 0.0685441486340084
0.9000900090009 0.407914567559477 0.0678746566556499
0.92009200920092 0.40658803222145 0.0672146240034417
0.94009400940094 0.405271634534085 0.0665638792325663
0.96009600960096 0.403965259135029 0.0659222546677032
0.98009800980098 0.402668792404299 0.0652895863058882
1.000100010001 0.401382122431507 0.0646657137222388
1.02010201020102 0.400105138983832 0.0640504799784488
1.04010401040104 0.398837733474697 0.0634437315339621
1.06010601060106 0.397579798933152 0.0628453181597367
1.08010801080108 0.396331229973939 0.0622550928545153
1.1001100110011 0.395091922768218 0.0616729117635179
1.12011201120112 0.393861775014935 0.0610986340994798
1.14011401140114 0.392640685912829 0.0605321220659588
1.16011601160116 0.391428556133043 0.0599732407828372
1.18011801180118 0.390225287792338 0.0594218582139479
1.2001200120012 0.389030784426892 0.0588778450967581
1.22012201220122 0.387844950966658 0.0583410748740425
1.24012401240124 0.386667693710287 0.0578114236274849
1.26012601260126 0.385498920300578 0.0572887700131451
1.28012801280128 0.384338539700469 0.0567729951987333
1.3001300130013 0.383186462169529 0.0562639828026347
1.32013201320132 0.382042599240954 0.0557616188346291
1.34013401340134 0.380906863699054 0.0552657916382529
1.36013601360136 0.379779169557213 0.054776391834752
1.38013801380138 0.378659432036313 0.0542933122685763
1.4001400140014 0.377547567543612 0.0538164479543683
1.42014201420142 0.376443493652066 0.0533456960253993
1.44014401440144 0.375347129080075 0.0528809556834081
1.46014601460146 0.374258393671659 0.0524221281498022
1.48014801480148 0.373177208377039 0.0519691166181752
1.5001500150015 0.372103495233616 0.0515218262081046
1.52015201520152 0.371037177347348 0.051080163920189
1.54015401540154 0.369978178874504 0.0506440385922876
1.56015601560156 0.368926425003791 0.0502133608569264
1.58015801580158 0.367881841938848 0.0497880430998355
1.6001600160016 0.366844356881088 0.0493679994195834
1.62016201620162 0.365813898012895 0.0489531455882773
1.64016401640164 0.364790394481154 0.0485433990132948
1.66016601660166 0.363773776381114 0.0481386787000202

1.68016801680168 0.362763974740574 0.0477389052155517
1.7001700170017 0.361760921504381 0.0473440006533552
1.72017201720172 0.360764549519242 0.0469538885988328
1.74017401740174 0.359774792518834 0.0465684940957828
1.76017601760176 0.358791585109204 0.0461877436137229
1.78017801780178 0.357814862754465 0.045811565016053
1.8001800180018 0.356844561762765 0.0454398875290323
1.82018201820182 0.355880619272534 0.0450726417115477
1.84018401840184 0.354922973238997 0.0447097594256515
1.86018601860186 0.353971562420953 0.0443511738078444
1.88018801880188 0.353026326367806 0.0439968192410851
1.9001900190019 0.352087205406846 0.0436466313275036
1.92019201920192 0.35115414063078 0.0433005468617994
1.94019401940194 0.350227073885496 0.0429585038053054
1.96019601960196 0.349305947758064 0.0426204412606978
1.98019801980198 0.348390705564963 0.0422862994473349
2.000200020002 0.347481291340541 0.0419560196772064
2.02020202020202 0.346577649825676 0.0416295443314769
2.04020402040204 0.34567972645667 0.041306816837606
2.06020602060206 0.34478746735434 0.0409877816470308
2.08020802080208 0.343900819313318 0.0406723842133932
2.1002100210021 0.343019729791547 0.0403605709712981
2.12021202120212 0.34214414689998 0.0400522893155884
2.14021402140214 0.341274019392467 0.0397474875811213
2.16021602160216 0.340409296655822 0.0394461150230336
2.18021802180218 0.33954992870009 0.0391481217974816
2.2002200220022 0.338695866148977 0.0388534589428432
2.22022202220222 0.337847060230461 0.0385620783613702
2.24022402240224 0.337003462767583 0.0382739328012777
2.26022602260226 0.336165026169393 0.0379889758392594
2.28022802280228 0.335331703422069 0.0377071618634175
2.3002300230023 0.334503448080194 0.0374284460565958
2.32023202320232 0.333680214258193 0.0371527843801053
2.34023402340234 0.332861956621922 0.0368801335578327
2.36023602360236 0.332048630380408 0.0366104510607197
2.38023802380238 0.331240191277742 0.036343695091606
2.4002400240024 0.330436595585106 0.036079824570424
2.42024202420242 0.329637800092957 0.0358187991197376
2.44024402440244 0.328843762103337 0.0355605790506149
2.46024602460246 0.328054439422325 0.0353051253488277
2.48024802480248 0.327269790352619 0.0350523996613673
2.5002500250025 0.326489773686257 0.0348023642832699
2.52025202520252 0.325714348697452 0.0345549821447431
2.54025402540254 0.324943475135567 0.0343102167985859

2.56025602560256 0.324177113218199 0.0340680324078942
2.58025802580258 0.323415223624394 0.0338283937340451
2.6002600260026 0.322657767487977 0.0335912661249525
2.62026202620262 0.32190470639099 0.0333566155035871
2.64026402640264 0.321156002357256 0.0331244083567548
2.66026602660266 0.320411617846041 0.0328946117241251
2.68026802680268 0.319671515745837 0.032667193187506
2.7002700270027 0.318935659368237 0.0324421208603562
2.72027202720272 0.31820401244193 0.0322193633775315
2.74027402740274 0.317476539106787 0.0319988898852565
2.76027602760276 0.316753203908048 0.0317806700313194
2.78027802780278 0.316033971790617 0.031564673955481
2.8002800280028 0.315318808093439 0.031350872280095
2.82028202820282 0.314607678543984 0.0311392361009338
2.84028402840284 0.313900549252816 0.0309297369782133
2.86028602860286 0.313197386708256 0.0307223469278142
2.88028802880288 0.312498157771131 0.0305170384126928
2.9002900290029 0.311802829669616 0.0303137843344785
2.92029202920292 0.311111369994156 0.0301125580252517
2.94029402940294 0.310423746692472 0.0299133332394993
2.96029602960296 0.309739928064654 0.029716084146242
2.98029802980298 0.309059882758334 0.0295207853213309
3.000300030003 0.308383579763929 0.0293274117399076
3.02030203020302 0.307710988409976 0.0291359387690253
3.04030403040304 0.307042078358532 0.0289463421604262
3.06030603060306 0.306376819600657 0.0287585980434717
3.08030803080308 0.305715182451961 0.0285726829182214
3.1003100310031 0.30505713754823 0.0283885736486587
3.12031203120312 0.304402655841123 0.0282062474560574
3.14031403140314 0.303751708593935 0.0280256819124879
3.16031603160316 0.303104267377426 0.0278468549344585
3.18031803180318 0.302460304065726 0.0276697447766891
3.2003200320032 0.301819790832291 0.0274943300260143
3.22032203220322 0.301182700145941 0.0273205895954125
3.24032403240324 0.300549004766942 0.0271485027181585
3.26032603260326 0.299918677743168 0.0269780489420959
3.28032803280328 0.299291692406308 0.0268092081240279
3.3003300330033 0.298668022368145 0.0266419604242225
3.32033203320332 0.298047641516887 0.0264762863010295
3.34033403340334 0.297430524013556 0.0263121665056085
3.36033603360336 0.296816644288434 0.0261495820767624
3.38033803380338 0.296205977037566 0.0259885143358765
3.4003400340034 0.295598497219317 0.0258289448819598
3.42034203420342 0.294994180050979 0.0256708555867857

3.44034403440344	0.294393001005435	0.0255142285901313
3.46034603460346	0.293794935807871	0.0253590462951111
3.48034803480348	0.293199960432543	0.0252052913636048
3.5003500350035	0.292608051099585	0.0250529467117759
3.52035203520352	0.29201918427188	0.0249019955056795
3.54035403540354	0.291433336651961	0.0247524211569573
3.56035603560356	0.290850485178978	0.0246042073186179
3.58035803580358	0.290270607025694	0.0244573378808999
3.6003600360036	0.289693679595538	0.024311796967217
3.62036203620362	0.289119680519699	0.0241675689301826
3.64036403640364	0.288548587654262	0.0240246383477119
3.66036603660366	0.28798037907739	0.0238829900192007
3.68036803680368	0.287415033086546	0.0237426089617781
3.7003700370037	0.286852528195759	0.0236034804066326
3.72037203720372	0.286292843132929	0.0234655897954087
3.74037403740374	0.28573595683717	0.023328922776674
3.76037603760376	0.285181848456199	0.023193465202454
3.78037803780378	0.284630497343758	0.0230592031248333
3.8003800380038	0.284081883057075	0.0229261227926227
3.82038203820382	0.283535985354364	0.0227942106480897
3.84038403840384	0.282992784192362	0.0226634533237514
3.86038603860386	0.282452259723899	0.0225338376392285
3.88038803880388	0.281914392295509	0.0224053505981594
3.9003900390039	0.28137916244507	0.0222779793851722
3.92039203920392	0.280846550899481	0.0221517113629147
3.94039403940394	0.280316538572376	0.0220265340691398
3.96039603960396	0.279789106561862	0.0219024352138458
3.98039803980398	0.279264236148304	0.0217794026764706
4.000400040004	0.278741908792125	0.0216574245031386
4.02040204020402	0.278222106131654	0.0215364889039584
4.04040404040404	0.27770480998099	0.0214165842503714
4.06040604060406	0.277190002327911	0.0212976990725492
4.08040804080408	0.276677665331799	0.0211798220568399
4.1004100410041	0.276167781321607	0.0210629420432609
4.12041204120412	0.275660332793845	0.0209470480230386
4.14041404140414	0.275155302410601	0.0208321291361928
4.16041604160416	0.274652672997587	0.0207181746691659
4.18041804180418	0.274152427542214	0.0206051740524953
4.2004200420042	0.273654549191693	0.0204931168585283
4.22042204220422	0.273159021251163	0.0203819927991791
4.24042404240424	0.272665827181846	0.0202717917237256
4.26042604260426	0.272174950599227	0.0201625036166476
4.28042804280428	0.27168637527126	0.0200541185955024
4.3004300430043	0.271200085116599	0.0199466269088406

4.32043204320432 0.270716064202853 0.0198400189341578
4.34043404340434 0.270234296744862 0.0197342851758843
4.36043604360436 0.269754767103007 0.0196294162634102
4.38043804380438 0.269277459781531 0.0195254029491459
4.4004400440044 0.268802359426893 0.0194222361066174
4.42044204420442 0.268329450826135 0.0193199067285956
4.44044404440444 0.267858718905282 0.0192184059252584
4.46044604460446 0.267390148727755 0.0191177249223855
4.48044804480448 0.266923725492812 0.0190178550595857
4.5004500450045 0.266459434534006 0.0189187877885544
4.52045204520452 0.265997261317662 0.0188205146713628
4.54045404540454 0.265537191441387 0.0187230273787766
4.56045604560456 0.265079210632581 0.0186263176886039
4.58045804580458 0.264623304746985 0.0185303774840727
4.6004600460046 0.264169459767239 0.0184351987522361
4.62046204620462 0.263717661801464 0.0183407735824057
4.64046404640464 0.263267897081857 0.0182470941646114
4.66046604660466 0.262820151963315 0.0181541527880889
4.68046804680468 0.262374412922066 0.0180619418397925
4.7004700470047 0.261930666554328 0.0179704538029341
4.72047204720472 0.261488899574976 0.017879681255547
4.74047404740474 0.261049098816237 0.0177896168690742
4.76047604760476 0.260611251226395 0.0177002534069817
4.78047804780478 0.260175343868514 0.0176115837233941
4.8004800480048 0.259741363919183 0.0175236007617549
4.82048204820482 0.25930929866727 0.0174362975535087
4.84048404840484 0.258879135512695 0.017349667216806
4.86048604860486 0.258450861965226 0.0172637029552297
4.88048804880488 0.258024465643279 0.0171783980565437
4.9004900490049 0.25759993427274 0.0170937458914621
4.92049204920492 0.257177255685804 0.0170097399124391
4.94049404940494 0.256756417819826 0.0169263736524794
4.96049604960496 0.256337408716185 0.0168436407239687
4.98049804980498 0.255920216519168 0.0167615348175229
5.000500050005 0.255504829474866 0.0166800497008577
5.02050205020502 0.255091235930081 0.0165991792176761
5.04050405040504 0.254679424331251 0.0165189172865745
5.06050605060506 0.254269383223388 0.0164392578999674
5.08050805080508 0.25386110124903 0.0163601951230291
5.1005100510051 0.253454567147202 0.0162817230926537
5.12051205120512 0.253049769752398 0.0162038360164315
5.14051405140514 0.252646697993569 0.0161265281716427
5.16051605160516 0.252245340893126 0.0160497939042671
5.18051805180518 0.251845687565958 0.0159736276280107

5.2005200520052 0.251447727218458 0.0158980238233471
5.22052205220522 0.251051449147567 0.0158229770365759
5.24052405240524 0.250656842739822 0.0157484818788951
5.26052605260526 0.250263897470425 0.0156745330254892
5.28052805280528 0.249872602902316 0.015601125214632
5.3005300530053 0.249482948685263 0.0155282532468039
5.32053205320532 0.24909492455496 0.0154559119838229
5.34053405340534 0.248708520332137 0.0153840963479907
5.36053605360536 0.248323725921682 0.0153128013212509
5.38053805380538 0.247940531311774 0.0152420219443622
5.4005400540054 0.247558926573023 0.015171753316084
5.42054205420542 0.247178901857627 0.0151019905923749
5.44054405440544 0.246800447398534 0.0150327289856044
5.46054605460546 0.246423553508616 0.0149639637637767
5.48054805480548 0.246048210579855 0.0148956902497674
5.5005500550055 0.245674409082535 0.0148279038205711
5.52055205520552 0.245302139564447 0.0147605999065623
5.54055405540554 0.244931392650105 0.0146937739907666
5.56055605560556 0.244562159039965 0.0146274216081447
5.58055805580558 0.244194429509661 0.0145615383448861
5.6005600560056 0.243828194909245 0.0144961198377153
5.62056205620562 0.243463446162441 0.0144311617732078
5.64056405640564 0.243100174265901 0.014366659887117
5.66056605660566 0.24273837028848 0.0143026099637121
5.68056805680568 0.242378025370508 0.0142390078351255
5.7005700570057 0.242019130723078 0.0141758493807109
5.72057205720572 0.241661677627345 0.0141131305264107
5.74057405740574 0.241305657433825 0.014050847244134
5.76057605760576 0.240951061561707 0.0139889955511431
5.78057805780578 0.240597881498176 0.0139275715094507
5.8005800580058 0.240246108797738 0.0138665712252249
5.82058205820582 0.239895735081559 0.0138059908482047
5.84058405840584 0.239546752036805 0.0137458265711231
5.86058605860586 0.239199151415997 0.0136860746291404
5.88058805880588 0.238852925036367 0.0136267312992847
5.9005900590059 0.238508064779228 0.0135677928999018
5.92059205920592 0.238164562589346 0.0135092557901131
5.94059405940594 0.237822410474322 0.013451116369282
5.96059605960596 0.237481600503982 0.0133933710764878
5.98059805980598 0.237142124809769 0.0133360163900078
6.000600060006 0.236803975584151 0.0132790488268075
6.02060206020602 0.236467145080025 0.013222464942038
6.04060406040604 0.23613162561014 0.0131662613285408
6.06060606060606 0.235797409546515 0.0131104346163606

6.08060806080608 0.235464489319871 0.0130549814722648
6.1006100610061 0.23513285741907 0.0129998985992707
6.12061206120612 0.234802506390553 0.0129451827361785
6.14061406140614 0.234473428837793 0.0128908306571133
6.16061606160616 0.23414561742075 0.0128368391710716
6.18061806180618 0.233819064855333 0.0127832051214761
6.2006200620062 0.233493763912867 0.0127299253857363
6.22062206220622 0.23316970741957 0.0126769968748159
6.24062406240624 0.232846888256029 0.0126244165328064
6.26062606260626 0.232525299356691 0.0125721813365066
6.28062806280628 0.232204933709349 0.0125202882950091
6.3006300630063 0.231885784354645 0.0124687344492919
6.32063206320632 0.23156784438557 0.0124175168718167
6.34063406340634 0.231251106946974 0.0123666326661323
6.36063606360636 0.230935565235082 0.0123160789664845
6.38063806380638 0.230621212497009 0.0122658529374312
6.4006400640064 0.230308042030291 0.0122159517734631
6.42064206420642 0.229996047182412 0.0121663726986298
6.44064406440644 0.229685221350339 0.0121171129661716
6.46064606460646 0.229375557980067 0.0120681698581563
6.48064806480648 0.229067050566161 0.0120195406851214
6.5006500650065 0.22875969265131 0.0119712227857211
6.52065206520652 0.22845347782588 0.0119232135263785
6.54065406540654 0.228148399727477 0.0118755103009433
6.56065606560656 0.227844452040513 0.0118281105303533
6.58065806580658 0.227541628495776 0.0117810116623015
6.6006600660066 0.227239922870004 0.0117342111709078
6.62066206620662 0.226939328985468 0.0116877065563951
6.64066406640664 0.226639840709553 0.0116414953447702
6.66066606660666 0.226341451954349 0.0115955750875091
6.68066806680668 0.226044156676246 0.0115499433612465
6.7006700670067 0.225747948875529 0.0115045977674704
6.72067206720672 0.225452822595981 0.0114595359322199
6.74067406740674 0.225158771924492 0.0114147555057884
6.76067606760676 0.224865790990668 0.0113702541624296
6.78067806780678 0.224573873966446 0.0113260296000692
6.8006800680068 0.224283015065715 0.0112820795400192
6.82068206820682 0.223993208543936 0.0112384017266965
6.84068406840684 0.223704448697774 0.0111949939273464
6.86068606860686 0.223416729864725 0.0111518539317686
6.88068806880688 0.223130046422757 0.0111089795520474
6.9006900690069 0.222844392789942 0.0110663686222862
6.92069206920692 0.222559763424105 0.0110240189983449
6.94069406940694 0.222276152822469 0.0109819285575813

6.96069606960696 0.221993555521306 0.0109400951985958
6.98069806980698 0.221711966095591 0.01089851684098
7.000700070007 0.221431379158658 0.0108571914250684
7.02070207020702 0.221151789361868 0.0108161169116932
7.04070407040704 0.220873191394268 0.0107752912819433
7.06070607060706 0.220595579982261 0.0107347125369259
7.08070807080708 0.220318949889283 0.0106943786975313
7.1007100710071 0.22004329591547 0.0106542878042014
7.12071207120712 0.219768612897348 0.0106144379167007
7.14071407140714 0.219494895707505 0.0105748271138909
7.16071607160716 0.219222139254286 0.0105354534935084
7.18071807180718 0.218950338481473 0.0104963151719444
7.2007200720072 0.218679488367987 0.0104574102840286
7.22072207220722 0.218409583927574 0.0104187369828154
7.24072407240724 0.218140620208513 0.0103802934393729
7.26072607260726 0.21787259229331 0.0103420778425749
7.28072807280728 0.217605495298407 0.0103040883988957
7.3007300730073 0.21733932437389 0.0102663233322074
7.32073207320732 0.217074074703197 0.0102287808835801
7.34073407340734 0.216809741502837 0.0101914593110848
7.36073607360736 0.216546320022102 0.0101543568895985
7.38073807380738 0.21628380554279 0.0101174719106123
7.4007400740074 0.216022193378924 0.010080802682042
7.42074207420742 0.215761478876483 0.010044347528041
7.44074407440744 0.215501657413125 0.0100081047888156
7.46074607460746 0.215242724397922 0.00997207282044296
7.48074807480748 0.214984675271091 0.00993624999469145
7.5007500750075 0.214727505503732 0.00990063469884302
7.52075207520752 0.214471210597568 0.00986522533551825
7.54075407540754 0.214215786084683 0.0098300203225034
7.56075607560756 0.213961227527271 0.00979501809258
7.58075807580758 0.213707530517382 0.00976021709335633
7.6007600760076 0.213454690676668 0.00972561578710132
7.62076207620762 0.21320270365614 0.00969121265058053
7.64076407640764 0.21295156513592 0.00965700617489413
7.66076607660766 0.212701270824997 0.0096229948653172
7.68076807680768 0.212451816460989 0.00958917724114182
7.7007700770077 0.212203197809904 0.00955555183552147
7.72077207720772 0.211955410665903 0.00952211719531713
7.74077407740774 0.211708450851067 0.00948887188094555
7.76077607760776 0.211462314215169 0.00945581446622948
7.78077807780778 0.211216996635438 0.00942294353824958
7.8007800780078 0.210972494016342 0.00939025769719851
7.82078207820782 0.210728802289356 0.00935775555562366

7.84078407840784 0.210485917412742 0.00932543574134962
7.86078607860786 0.210243835371331 0.0092932968912081
7.88078807880788 0.210002552176304 0.00926133765702862
7.9007900790079 0.209762063864976 0.00922955670243671
7.92079207920792 0.209522366500584 0.00919795270333157
7.94079407940794 0.209283456172072 0.00916652434775248
7.96079607960796 0.209045328993886 0.00913527033574688
7.98079807980798 0.208807981105764 0.00910418937924003
8.000800080008 0.208571408672534 0.00907328020190644
8.02080208020802 0.208335607883904 0.00904254153904276
8.04080408040804 0.208100574954269 0.00901197213744243
8.06080608060806 0.207866306122506 0.00898157075527167
8.08080808080808 0.207632797651779 0.00895133616194725
8.1008100810081 0.207400045829341 0.00892126713801557
8.12081208120812 0.207168046966343 0.00889136247503334
8.14081408140814 0.206936797397644 0.00886162097544981
8.16081608160816 0.206706293481615 0.00883204145249026
8.18081808180818 0.206476531599955 0.00880262273004106
8.2008200820082 0.206247508157506 0.00877336364253622
8.22082208220822 0.206019219582065 0.00874426303484509
8.24082408240824 0.205791662324203 0.00871531976216169
8.26082608260826 0.205564832857084 0.0086865326898952
8.28082808280828 0.205338727676286 0.00865790069356201
8.3008300830083 0.205113343299623 0.00862942265867881
8.32083208320832 0.204888676266969 0.00860109748065725
8.34083408340834 0.204664723140084 0.00857292406469966
8.36083608360836 0.20444148050244 0.00854490132569623
8.38083808380838 0.204218944959054 0.00851702818812328
8.4008400840084 0.203997113136314 0.0084893035859429
8.42084208420842 0.203775981681815 0.00846172646250363
8.44084408440844 0.20355554726419 0.00843429577044256
8.46084608460846 0.203335806572947 0.00840701047158841
8.48084808480848 0.203116756318306 0.00837986953686587
8.5008500850085 0.202898393231037 0.00835287194620112
8.52085208520852 0.2026807140623 0.00832601668842838
8.54085408540854 0.202463715583486 0.00829930276119771
8.56085608560856 0.202247394586062 0.00827272917088376
8.58085808580858 0.202031747881412 0.00824629493249579
8.6008600860086 0.201816772300687 0.00821999906958854
8.62086208620862 0.201602464694649 0.00819384061417444
8.64086408640864 0.20138882193352 0.00816781860663658
8.66086608660866 0.201175840906835 0.00814193209564286
8.68086808680868 0.200963518523289 0.00811618013806123
8.7008700870087 0.200751851710595 0.00809056179887573

8.72087208720872 0.200540837415334 0.00806507615110364
8.74087408740874 0.200330472602811 0.00803972227571372
8.76087608760876 0.200120754256917 0.00801449926154513
8.78087808780878 0.199911679379978 0.00798940620522756
8.8008800880088 0.199703244992623 0.00796444221110216
8.82088208820882 0.199495448133642 0.00793960639114344
8.84088408840884 0.199288285859844 0.00791489786488211
8.86088608860886 0.199081755245927 0.00789031575932874
8.88088808880888 0.198875853384339 0.0078658592088984
8.9008900890089 0.198670577385143 0.00784152735533613
8.92089208920892 0.198465924375885 0.00781731934764325
8.94089408940894 0.198261891501463 0.0077932343420046
8.96089608960896 0.198058475923996 0.00776927150171657
8.98089808980898 0.197855674822694 0.00774542999711595
9.000900090009 0.197653485393729 0.00772170900550965
9.02090209020902 0.197451904850111 0.00769810771110521
9.04090409040904 0.197250930421558 0.00767462530494206
9.06090609060906 0.197050559354374 0.00765126098482371
9.08090809080908 0.196850788911324 0.00762801395525052
9.1009100910091 0.196651616371511 0.00760488342735339
9.12091209120912 0.196453039030257 0.0075818686188282
9.14091409140914 0.19625505419898 0.0075589687538709
9.16091609160916 0.196057659205074 0.00753618306311343
9.18091809180918 0.195860851391794 0.00751351078356037
9.2009200920092 0.195664628118137 0.00749095115852621
9.22092209220922 0.195468986758723 0.00746850343757352
9.24092409240924 0.195273924703685 0.00744616687645161
9.26092609260926 0.195079439358552 0.00742394073703601
9.28092809280928 0.194885528144135 0.00740182428726862
9.3009300930093 0.194692188496417 0.00737981680109849
9.32093209320932 0.19449941786644 0.00735791755842337
9.34093409340934 0.194307213720195 0.00733612584503175
9.36093609360936 0.194115573538515 0.00731444095254573
9.38093809380938 0.193924494816965 0.00729286217836439
9.4009400940094 0.193733975065732 0.00727138882560791
9.42094209420942 0.193544011809525 0.00725002020306219
9.44094409440944 0.193354602587464 0.00722875562512423
9.46094609460946 0.193165744952976 0.00720759441174797
9.48094809480948 0.192977436473696 0.00718653588839089
9.5009500950095 0.192789674731358 0.00716557938596107
9.52095209520952 0.192602457321698 0.0071447242407649
9.54095409540954 0.19241578185435 0.00712396979445536
9.56095609560956 0.192229645952746 0.00710331539398087
9.58095809580958 0.192044047254022 0.00708276039153474

9.6009600960096 0.191858983408911 0.00706230414450511
9.62096209620962 0.191674452081651 0.00704194601542546
9.64096409640964 0.191490450949889 0.00702168537192577
9.66096609660966 0.191306977704582 0.007001521586684
9.68096809680968 0.191124030049904 0.00698145403737836
9.7009700970097 0.190941605703149 0.00696148210663987
9.72097209720972 0.190759702394644 0.00694160518200564
9.74097409740974 0.190578317867647 0.00692182265587245
9.76097609760976 0.190397449878265 0.00690213392545105
9.78097809780978 0.190217096195354 0.00688253839272082
9.8009800980098 0.190037254600436 0.00686303546438495
9.82098209820982 0.189857922887606 0.0068436245518261
9.84098409840984 0.189679098863442 0.00682430507106264
9.86098609860986 0.18950078034692 0.00680507644270516
9.88098809880988 0.189322965169326 0.0067859380919137
9.9009900990099 0.189145651174168 0.0067668894483552
9.92099209920992 0.18896883621709 0.00674792994616159
9.94099409940994 0.188792518165791 0.00672905902388828
9.96099609960996 0.188616694899936 0.00671027612447299
9.98099809980998 0.188441364311073 0.0066915806951952
10.00100010001 0.188266524302553 0.00667297218763589
10.02100210021 0.188092172789446 0.00665445005763782
10.04100410041 0.187918307698457 0.00663601376526615
10.0610061006101 0.187744926967851 0.00661766277476948
10.0810081008101 0.187572028547365 0.0065993965545414
10.1010101010101 0.187399610398137 0.00658121457708234
10.1210121012101 0.187227670492619 0.0065631163189619
10.1410141014101 0.187056206814506 0.00654510126078154
10.1610161016102 0.186885217358653 0.00652716888713766
10.1810181018102 0.186714700131 0.00650931868658512
10.2010201020102 0.186544653148497 0.00649155015160107
10.2210221022102 0.186375074439026 0.00647386277854923
10.2410241024102 0.186205962041328 0.00645625606764457
10.2610261026103 0.186037314004927 0.00643872952291819
10.2810281028103 0.185869128390057 0.00642128265218279
10.3010301030103 0.185701403267589 0.00640391496699839
10.3210321032103 0.185534136718957 0.00638662598263839
10.3410341034103 0.185367326836089 0.00636941521805603
10.3610361036104 0.185200971721333 0.0063522821958512
10.3810381038104 0.185035069487384 0.00633522644223757
10.4010401040104 0.184869618257221 0.00631824748701007
10.4210421042104 0.184704616164029 0.00630134486351277
10.4410441044104 0.184540061351135 0.00628451810860694
10.4610461046105 0.18437595197194 0.00626776676263961

10.4810481048105 0.184212286189846 0.00625109036941238
10.5010501050105 0.184049062178193 0.00623448847615053
10.5210521052105 0.183886278120192 0.00621796063347251
10.5410541054105 0.183723932208855 0.00620150639535968
10.5610561056106 0.183562022646933 0.00618512531912641
10.5810581058106 0.18340054764685 0.00616881696539047
10.6010601060106 0.183239505430635 0.00615258089804372
10.6210621062106 0.183078894229863 0.00613641668422311
10.6410641064106 0.182918712285585 0.00612032389428197
10.6610661066107 0.182758957848272 0.00610430210176161
10.6810681068107 0.182599629177747 0.00608835088336315
10.7010701070107 0.182440724543122 0.00607246981891975
10.7210721072107 0.182282242222742 0.00605665849136903
10.7410741074107 0.182124180504119 0.00604091648672576
10.7610761076108 0.181966537683871 0.0060252433940549
10.7810781078108 0.181809312067666 0.00600963880544492
10.8010801080108 0.181652501970157 0.00599410231598124
10.8210821082108 0.181496105714927 0.00597863352372015
10.8410841084108 0.181340121634426 0.00596323202966284
10.8610861086109 0.181184548069919 0.00594789743772978
10.8810881088109 0.181029383371419 0.00593262935473525
10.9010901090109 0.180874625897638 0.00591742739036227
10.9210921092109 0.180720274015926 0.00590229115713768
10.9410941094109 0.180566326102214 0.0058872202704075
10.961096109611 0.18041278054096 0.00587221434831253
10.981098109811 0.18025963572509 0.00585727301176424
11.001100110011 0.180106890055948 0.00584239588442077
11.021102110211 0.179954541943235 0.00582758259266338
11.041104110411 0.179802589804961 0.00581283276557293
11.0611061106111 0.179651032067385 0.00579814603490671
11.0811081108111 0.179499867164966 0.00578352203507546
11.1011101110111 0.179349093540306 0.00576896040312063
11.1211121112111 0.179198709644103 0.00575446077869192
11.1411141114111 0.179048713935091 0.00574002280402485
11.1611161116112 0.178899104879995 0.00572564612391886
11.1811181118112 0.178749880953476 0.00571133038571529
11.2011201120112 0.178601040638081 0.00569707523927587
11.2211221122112 0.17845258242419 0.00568288033696123
11.2411241124112 0.17830450480997 0.00566874533360967
11.2611261126113 0.178156806301322 0.00565466988651624
11.2811281128113 0.178009485411831 0.0056406536554118
11.3011301130113 0.177862540662719 0.00562669630244256
11.3211321132113 0.177715970582797 0.00561279749214961
11.3411341134113 0.17756977370841 0.00559895689144872

11.3611361136114 0.177423948583399 0.00558517416961038
11.3811381138114 0.177278493759046 0.00557144899823996
11.4011401140114 0.177133407794027 0.00555778105125811
11.4211421142114 0.176988689254369 0.00554417000488137
11.4411441144114 0.176844336713402 0.00553061553760289
11.4611461146115 0.176700348751711 0.00551711733017336
11.4811481148115 0.176556723957089 0.00550367506558226
11.5011501150115 0.176413460924497 0.00549028842903907
11.5211521152115 0.176270558256015 0.00547695710795482
11.5411541154115 0.176128014560796 0.00546368079192374
11.5611561156116 0.175985828455023 0.00545045917270517
11.5811581158116 0.175843998561867 0.00543729194420554
11.6011601160116 0.175702523511439 0.00542417880246057
11.6211621162116 0.17556140194075 0.00541111944561768
11.6411641164116 0.175420632493666 0.00539811357391849
11.6611661166117 0.175280213820865 0.00538516088968162
11.6811681168117 0.175140144579795 0.00537226109728539
11.7011701170117 0.175000423434633 0.00535941390315106
11.7211721172117 0.174861049056241 0.00534661901572587
11.7411741174117 0.174722020122125 0.0053338761454665
11.7611761176118 0.174583335316394 0.00532118500482254
11.7811781178118 0.17444499332972 0.00530854530822015
11.8011801180118 0.174306992859296 0.00529595677204596
11.8211821182118 0.174169332608796 0.00528341911463097
11.8411841184118 0.174032011288333 0.00527093205623472
11.8611861186119 0.173895027614425 0.0052584953190296
11.8811881188119 0.173758380309949 0.00524610862708523
11.9011901190119 0.173622068104105 0.00523377170635305
11.9211921192119 0.173486089732377 0.00522148428465109
11.9411941194119 0.173350443936494 0.00520924609164876
11.961196119612 0.173215129464392 0.00519705685885194
11.981198119812 0.173080145070174 0.00518491631958807
12.001200120012 0.172945489514077 0.00517282420899145
12.021202120212 0.172811161562428 0.0051607802639887
12.041204120412 0.172677159987614 0.00514878422328426
12.0612061206121 0.172543483568037 0.00513683582734616
12.0812081208121 0.172410131088086 0.00512493481839177
12.1012101210121 0.172277101338092 0.00511308094037386
12.1212121212121 0.172144393114301 0.00510127393896659
12.1412141214121 0.172012005218828 0.00508951356155178
12.1612161216122 0.17187993645963 0.00507779955720527
12.1812181218122 0.171748185650467 0.00506613167668339
12.2012201220122 0.171616751610867 0.00505450967240953
12.2212221222122 0.17148563316609 0.00504293329846089

12.2412241224122 0.171354829147097 0.00503140231055532
12.2612261226123 0.171224338390512 0.0050199164660383
12.2812281228123 0.171094159738589 0.00500847552387001
12.3012301230123 0.170964292039181 0.00499707924461252
12.3212321232123 0.170834734145701 0.00498572739041715
12.3412341234123 0.170705484917093 0.00497441972501192
12.3612361236124 0.170576543217798 0.00496315601368904
12.3812381238124 0.170447907917719 0.00495193602329261
12.4012401240124 0.170319577892192 0.00494075952220645
12.4212421242124 0.170191552021951 0.00492962628034191
12.4412441244124 0.170063829193094 0.00491853606912592
12.4612461246125 0.169936408297057 0.0049074886614891
12.4812481248125 0.169809288230577 0.00489648383185397
12.5012501250125 0.169682467895662 0.00488552135612326
12.5212521252125 0.169555946199561 0.00487460101166838
12.5412541254125 0.169429722054731 0.00486372257731794
12.5612561256126 0.169303794378808 0.00485288583334634
12.5812581258126 0.169178162094574 0.00484209056146263
12.6012601260126 0.16905282412993 0.00483133654479923
12.6212621262126 0.168927779417862 0.00482062356790094
12.6412641264126 0.168803026896416 0.00480995141671399
12.6612661266127 0.168678565508663 0.00479931987857513
12.6812681268127 0.168554394202671 0.00478872874220095
12.7012701270127 0.168430511931479 0.00477817779767715
12.7212721272127 0.168306917653063 0.00476766683644797
12.7412741274127 0.16818361033031 0.00475719565130577
12.7612761276128 0.168060588930991 0.00474676403638061
12.7812781278128 0.167937852427725 0.00473637178712998
12.8012801280128 0.167815399797962 0.00472601870032859
12.8212821282128 0.167693230023943 0.00471570457405826
12.8412841284128 0.167571342092683 0.00470542920769793
12.8612861286129 0.167449734995933 0.00469519240191371
12.8812881288129 0.167328407730162 0.00468499395864906
12.9012901290129 0.167207359296523 0.00467483368111503
12.9212921292129 0.167086588700828 0.00466471137378057
12.9412941294129 0.166966094953522 0.00465462684236301
12.961296129613 0.166845877069655 0.0046445798938185
12.981298129813 0.166725934068855 0.00463457033633265
13.001300130013 0.166606264975303 0.00462459797931115
13.021302130213 0.166486868817707 0.00461466263337057
13.041304130413 0.166367744629275 0.00460476411032918
13.0613061306131 0.166248891447686 0.00459490222319784
13.0813081308131 0.166130308315072 0.00458507678617104
13.1013101310131 0.166011994277986 0.00457528761461792

13.1213121312131 0.165893948387379 0.00456553452507347
13.1413141314131 0.165776169698574 0.00455581733522974
13.1613161316132 0.165658657271243 0.00454613586392712
13.1813181318132 0.165541410169379 0.00453648993114575
13.2013201320132 0.165424427461274 0.00452687935799697
13.2213221322132 0.165307708219495 0.00451730396671486
13.2413241324132 0.165191251520857 0.00450776358064781
13.2613261326133 0.165075056446399 0.00449825802425022
13.2813281328133 0.164959122081365 0.00448878712307424
13.3013301330133 0.164843447515172 0.00447935070376161
13.3213321332133 0.164728031841394 0.00446994859403552
13.3413341334133 0.164612874157734 0.00446058062269258
13.3613361336134 0.164497973566004 0.00445124661959486
13.3813381338134 0.164383329172095 0.00444194641566197
13.4013401340134 0.164268940085965 0.00443267984286323
13.4213421342134 0.164154805421606 0.00442344673420994
13.4413441344134 0.164040924297026 0.00441424692374761
13.4613461346135 0.163927295834225 0.00440508024654836
13.4813481348135 0.163813919159176 0.00439594653870339
13.5013501350135 0.163700793401798 0.00438684563731541
13.5213521352135 0.163587917695935 0.00437777738049122
13.5413541354135 0.163475291179338 0.00436874160733437
13.5613561356136 0.163362912993637 0.00435973815793778
13.5813581358136 0.163250782284324 0.00435076687337656
13.6013601360136 0.163138898200732 0.00434182759570075
13.6213621362136 0.163027259896008 0.00433292016792824
13.6413641364136 0.162915866527098 0.00432404443403768
13.6613661366137 0.162804717254723 0.00431520023896148
13.6813681368137 0.162693811243357 0.00430638742857884
13.7013701370137 0.162583147661209 0.00429760584970889
13.7213721372137 0.162472725680201 0.00428885535010382
13.7413741374137 0.162362544475947 0.00428013577844214
13.7613761376138 0.162252603227733 0.00427144698432195
13.7813781378138 0.162142901118497 0.00426278881825426
13.8013801380138 0.162033437334808 0.00425416113165638
13.8213821382138 0.161924211066847 0.00424556377684543
13.8413841384138 0.161815221508389 0.00423699660703175
13.8613861386139 0.161706467856777 0.00422845947631256
13.8813881388139 0.161597949312909 0.00421995223966548
13.9013901390139 0.161489665081216 0.00421147475294224
13.9213921392139 0.161381614369642 0.00420302687286242
13.9413941394139 0.161273796389627 0.00419460845700716
13.961396139614 0.161166210356083 0.00418621936381304
13.981398139814 0.161058855487383 0.00417785945256593

14.001400140014 0.160951731005333 0.00416952858339492
14.021402140214 0.160844836135161 0.00416122661726627
14.041404140414 0.160738170105494 0.00415295341597746
14.0614061406141 0.160631732148342 0.00414470884215129
14.0814081408141 0.160525521499077 0.00413649275922994
14.1014101410141 0.160419537396416 0.00412830503146919
14.1214121412141 0.160313779082406 0.00412014552393262
14.1414141414141 0.160208245802399 0.0041120141024859
14.1614161416142 0.160102936805041 0.00410391063379104
14.1814181418142 0.159997851342251 0.00409583498530086
14.2014201420142 0.159892988669203 0.0040877870252533
14.2214221422142 0.15978834804431 0.00407976662266593
14.2414241424142 0.159683928729207 0.00407177364733039
14.2614261426143 0.15957972998873 0.00406380796980704
14.2814281428143 0.159475751090904 0.00405586946141941
14.3014301430143 0.15937199130692 0.00404795799424895
14.3214321432143 0.159268449911125 0.00404007344112964
14.3414341434143 0.159165126181 0.00403221567564275
14.3614361436144 0.159062019397143 0.00402438457211156
14.3814381438144 0.158959128843257 0.0040165800055962
14.4014401440144 0.158856453806128 0.00400880185188849
14.4214421442144 0.158753993575613 0.00400104998750683
14.4414441444144 0.15865174744462 0.0039933242896911
14.4614461446145 0.158549714709095 0.00398562463639769
14.4814481448145 0.158447894668004 0.00397795090629445
14.5014501450145 0.158346286623316 0.0039703029787558
14.5214521452145 0.15824488987999 0.00396268073385777
14.5414541454145 0.158143703745957 0.00395508405237317
14.5614561456146 0.158042727532105 0.00394751281576675
14.5814581458146 0.157941960552264 0.0039399669061904
14.6014601460146 0.157841402123189 0.00393244620647841
14.6214621462146 0.157741051564544 0.00392495060014276
14.6414641464146 0.157640908198891 0.00391747997136843
14.6614661466147 0.15754097135167 0.00391003420500876
14.6814681468147 0.157441240351185 0.00390261318658087
14.7014701470147 0.157341714528591 0.00389521680226108
14.7214721472147 0.157242393217877 0.00388784493888037
14.7414741474147 0.157143275755852 0.00388049748391993
14.7614761476148 0.15704436148213 0.00387317432550666
14.7814781478148 0.156945649739116 0.00386587535240874
14.8014801480148 0.15684713987199 0.00385860045403133
14.8214821482148 0.156748831228694 0.00385134952041211
14.8414841484148 0.156650723159916 0.00384412244221702
14.8614861486149 0.156552815019078 0.00383691911073597

14.8814881488149 0.156455106162319 0.00382973941787861
14.9014901490149 0.156357595948484 0.00382258325617007
14.9214921492149 0.156260283739108 0.00381545051874682
14.9414941494149 0.1561631688984 0.00380834109935249
14.961496149615 0.156066250793236 0.00380125489233378
14.981498149815 0.155969528793136 0.00379419179263636
15.001500150015 0.155873002270258 0.00378715169580084
15.021502150215 0.155776670599381 0.00378013449795871
15.041504150415 0.155680533157891 0.00377314009582839
15.0615061506151 0.15558458932577 0.00376616838671125
15.0815081508151 0.155488838485579 0.00375921926848769
15.1015101510151 0.15539328002245 0.00375229263961325
15.1215121512151 0.155297913324067 0.00374538839911473
15.1415141514151 0.155202737780656 0.00373850644658641
15.1615161516152 0.155107752784973 0.00373164668218613
15.1815181518152 0.155012957732288 0.00372480900663164
15.2015201520152 0.154918352020375 0.00371799332119678
15.2215221522152 0.154823935049498 0.00371119952770778
15.2415241524152 0.154729706222396 0.00370442752853957
15.2615261526153 0.154635664944276 0.00369767722661213
15.2815281528153 0.154541810622795 0.00369094852538685
15.3015301530153 0.154448142668051 0.00368424132886293
15.3215321532153 0.154354660492567 0.00367755554157379
15.3415341534153 0.154261363511282 0.00367089106858357
15.3615361536154 0.154168251141539 0.00366424781548351
15.3815381538154 0.154075322803071 0.0036576256883886
15.4015401540154 0.153982577917986 0.00365102459393397
15.4215421542154 0.153890015910764 0.00364444443927154
15.4415441544154 0.153797636208234 0.00363788513206657
15.4615461546155 0.153705438239572 0.00363134658049432
15.4815481548155 0.153613421436281 0.00362482869323658
15.5015501550155 0.153521585232186 0.00361833137947847
15.5215521552155 0.153429929063417 0.00361185454890503
15.5415541554155 0.1533384523684 0.00360539811169799
15.5615561556156 0.153247154587848 0.00359896197853245
15.5815581558156 0.153156035164742 0.00359254606057374
15.6015601560156 0.153065093544328 0.00358615026947412
15.6215621562156 0.1529743291741 0.00357977451736961
15.6415641564156 0.152883741503792 0.00357341871687689
15.6615661566157 0.152793329985366 0.00356708278109008
15.6815681568157 0.152703094072998 0.00356076662357768
15.7015701570157 0.152613033223072 0.00355447015837946
15.7215721572157 0.152523146894164 0.00354819330000337
15.7415741574157 0.152433434547036 0.00354193596342257

15.7615761576158 0.152343895644621 0.0035356980640723
15.7815781578158 0.152254529652015 0.00352947951784699
15.8015801580158 0.152165336036463 0.00352328024109721
15.8215821582158 0.152076314267353 0.00351710015062673
15.8415841584158 0.151987463816201 0.00351093916368963
15.8615861586159 0.151898784156643 0.00350479719798733
15.8815881588159 0.151810274764425 0.00349867417166576
15.9015901590159 0.151721935117389 0.00349257000331243
15.9215921592159 0.151633764695466 0.00348648461195367
15.9415941594159 0.151545762980667 0.00348041791705171
15.961596159616 0.151457929457068 0.00347436983850196
15.981598159816 0.151370263610803 0.00346834029663017
16.001600160016 0.151282764930053 0.00346232921218973
16.021602160216 0.151195432905037 0.00345633650635887
16.041604160416 0.151108267028002 0.00345036210073796
16.0616061606161 0.15102126679321 0.00344440591734682
16.0816081608161 0.150934431696933 0.00343846787862206
16.1016101610161 0.150847761237438 0.00343254790741436
16.1216121612161 0.150761254914982 0.00342664592698589
16.1416141614161 0.1506749122318 0.00342076186100768
16.1616161616162 0.150588732692094 0.00341489563355697
16.1816181618162 0.150502715802027 0.00340904716911468
16.2016201620162 0.15041686106971 0.00340321639256286
16.2216221622162 0.150331168005195 0.00339740322918206
16.2416241624162 0.150245636120464 0.00339160760464892
16.2616261626163 0.150160264929419 0.00338582944503357
16.2816281628163 0.150075053947876 0.00338006867679719
16.3016301630163 0.149990002693552 0.00337432522678953
16.3216321632163 0.149905110686059 0.00336859902224646
16.3416341634163 0.149820377446892 0.00336288999078753
16.3616361636164 0.149735802499421 0.00335719806041354
16.3816381638164 0.149651385368883 0.00335152315950419
16.4016401640164 0.149567125582372 0.00334586521681563
16.4216421642164 0.149483022668829 0.00334022416147818
16.4416441644164 0.149399076159037 0.0033345999229939
16.4616461646165 0.149315285585606 0.00332899243123432
16.4816481648165 0.149231650482971 0.00332340161643809
16.5016501650165 0.149148170387377 0.00331782740920872
16.5216521652165 0.149064844836875 0.00331226974051227
16.5416541654165 0.148981673371312 0.00330672854167511
16.5616561656166 0.14889865553232 0.00330120374438167
16.5816581658166 0.148815790863313 0.00329569528067216
16.6016601660166 0.148733078909471 0.00329020308294047
16.6216621662166 0.148650519217739 0.00328472708393184

16.6416641664166 0.148568111336812 0.00327926721674081
16.6616661666167 0.148485854817133 0.00327382341480892
16.6816681668167 0.148403749210879 0.00326839561192267
16.7016701670167 0.148321794071958 0.00326298374221131
16.7216721672167 0.148239988955995 0.00325758774014479
16.7416741674167 0.148158333420329 0.00325220754053158
16.7616761676168 0.148076827024003 0.00324684307851665
16.7816781678168 0.147995469327755 0.00324149428957934
16.8016801680168 0.147914259894011 0.00323616110953133
16.8216821682168 0.147833198286876 0.0032308434745146
16.8416841684168 0.147752284072128 0.00322554132099938
16.8616861686169 0.14767151681721 0.00322025458578216
16.8816881688169 0.147590896091218 0.00321498320598364
16.9016901690169 0.147510421464899 0.00320972711904682
16.9216921692169 0.14743009251064 0.00320448626273494
16.9416941694169 0.147349908802461 0.0031992605751296
16.961696169617 0.147269869916006 0.00319404999462875
16.981698169817 0.147189975428539 0.00318885445994482
17.001700170017 0.147110224918932 0.00318367391010275
17.021702170217 0.147030617967661 0.00317850828443811
17.041704170417 0.146951154156796 0.0031733575225952
17.0617061706171 0.146871833069995 0.0031682215645252
17.0817081708171 0.146792654292496 0.00316310035048427
17.1017101710171 0.14671361741111 0.00315799382103171
17.1217121712171 0.146634722014214 0.00315290191702816
17.1417141714171 0.146555967691742 0.0031478245796337
17.1617161716172 0.146477354035181 0.00314276175030612
17.1817181718172 0.146398880637559 0.00313771337079904
17.2017201720172 0.146320547093443 0.00313267938316021
17.2217221722172 0.146242352998929 0.00312765972972966
17.2417241724172 0.146164297951635 0.00312265435313799
17.2617261726173 0.146086381550694 0.00311766319630459
17.2817281728173 0.146008603396747 0.00311268620243591
17.3017301730173 0.145930963091939 0.00310772331502376
17.3217321732173 0.145853460239907 0.00310277447784354
17.3417341734173 0.145776094445775 0.00309783963495262
17.3617361736174 0.145698865316151 0.00309291873068857
17.3817381738174 0.145621772459114 0.00308801170966754
17.4017401740174 0.145544815484212 0.00308311851678256
17.4217421742174 0.14546799400245 0.00307823909720191
17.4417441744174 0.145391307626292 0.00307337339636746
17.4617461746175 0.145314755969645 0.00306852135999306
17.4817481748175 0.145238338647858 0.00306368293406287
17.5017501750175 0.145162055277714 0.00305885806482983

17.5217521752175 0.145085905477421 0.003054046698814
17.5417541754175 0.145009888866611 0.00304924878280098
17.5617561756176 0.144934005066329 0.00304446426384037
17.5817581758176 0.144858253699027 0.00303969308924418
17.6017601760176 0.144782634388559 0.00303493520658525
17.6217621762176 0.144707146760175 0.00303019056369578
17.6417641764176 0.144631790440511 0.00302545910866571
17.6617661766177 0.144556565057589 0.00302074078984127
17.6817681768177 0.144481470240805 0.00301603555582342
17.7017701770177 0.144406505620924 0.00301134335546638
17.7217721772177 0.144331670830077 0.00300666413787611
17.7417741774177 0.14425696550175 0.00300199785240887
17.7617761776178 0.144182389270783 0.0029973444486697
17.7817781778178 0.144107941773359 0.002992703876511
17.8017801780178 0.144033622647001 0.00298807608603105
17.8217821782178 0.143959431530566 0.0029834610275726
17.8417841784178 0.143885368064237 0.00297885865172138
17.8617861786179 0.143811431889518 0.00297426890930478
17.8817881788179 0.143737622649229 0.00296969175139032
17.9017901790179 0.143663939987498 0.00296512712928434
17.9217921792179 0.14359038354976 0.00296057499453057
17.9417941794179 0.143516952982742 0.00295603529890873
17.961796179618 0.143443647934467 0.0029515079944332
17.981798179818 0.143370468054243 0.0029469930333516
18.001800180018 0.143297412992658 0.00294249036814351
18.021802180218 0.143224482401573 0.00293799995151904
18.041804180418 0.14315167593412 0.00293352173641755
18.0618061806181 0.143078993244694 0.0029290556760063
18.0818081808181 0.143006433988946 0.00292460172367914
18.1018101810181 0.142933997823781 0.00292015983305519
18.1218121812181 0.142861684407347 0.00291572995797754
18.1418141814181 0.142789493399036 0.00291131205251196
18.1618161816182 0.142717424459475 0.00290690607094564
18.1818181818182 0.142645477250519 0.00290251196778586
18.2018201820182 0.142573651435248 0.00289812969775877
18.2218221822182 0.142501946677962 0.00289375921580811
18.2418241824182 0.142430362644173 0.00288940047709397
18.2618261826183 0.142358899000602 0.00288505343699153
18.2818281828183 0.142287555415174 0.00288071805108987
18.3018301830183 0.142216331557009 0.00287639427519068
18.3218321832183 0.14214522709642 0.0028720820653071
18.3418341834183 0.142074241704909 0.00286778137766247
18.3618361836184 0.142003375055157 0.00286349216868918
18.3818381838184 0.141932626821024 0.00285921439502742

18.4018401840184 0.141861996677538 0.00285494801352401
18.4218421842184 0.141791484300897 0.00285069298123126
18.4418441844184 0.141721089368457 0.00284644925540576
18.4618461846185 0.141650811558733 0.00284221679350722
18.4818481848185 0.141580650551389 0.00283799555319734
18.5018501850185 0.141510606027235 0.00283378549233866
18.5218521852185 0.141440677668221 0.00282958656899339
18.5418541854185 0.141370865157436 0.00282539874142232
18.5618561856186 0.141301168179098 0.00282122196808365
18.5818581858186 0.141231586418549 0.00281705620763194
18.6018601860186 0.141162119562257 0.00281290141891692
18.6218621862186 0.141092767297801 0.00280875756098245
18.6418641864186 0.141023529313875 0.0028046245930654
18.6618661866187 0.140954405300279 0.00280050247459458
18.6818681868187 0.140885394947913 0.00279639116518961
18.7018701870187 0.140816497948775 0.00279229062465993
18.7218721872187 0.140747713995957 0.00278820081300365
18.7418741874187 0.140679042783634 0.00278412169040654
18.7618761876188 0.140610484007069 0.00278005321724097
18.7818781878188 0.140542037362599 0.00277599535406485
18.8018801880188 0.140473702547636 0.0027719480616206
18.8218821882188 0.140405479260662 0.00276791130083412
18.8418841884188 0.140337367201221 0.00276388503281377
18.8618861886189 0.140269366069919 0.00275986921884934
18.8818881888189 0.140201475568413 0.00275586382041104
18.9018901890189 0.140133695399416 0.00275186879914849
18.9218921892189 0.140066025266681 0.00274788411688975
18.9418941894189 0.139998464875008 0.00274390973564027
18.961896189619 0.139931013930231 0.00273994561758197
18.981898189819 0.139863672139217 0.00273599172507222
19.001900190019 0.13979643920986 0.00273204802064286
19.021902190219 0.139729314851081 0.00272811446699928
19.041904190419 0.139662298772817 0.00272419102701939
19.0619061906191 0.139595390686022 0.00272027766375274
19.0819081908191 0.139528590302659 0.00271637434041951
19.1019101910191 0.1394618973357 0.0027124810204096
19.1219121912191 0.139395311499116 0.00270859766728167
19.1419141914191 0.139328832507878 0.00270472424476225
19.1619161916192 0.139262460077949 0.00270086071674479
19.1819181918192 0.139196193926283 0.0026970070472887
19.2019201920192 0.139130033770818 0.00269316320061853
19.2219221922192 0.139063979330472 0.00268932914112299
19.2419241924192 0.138998030325143 0.00268550483335406
19.2619261926193 0.138932186475698 0.00268169024202612

19.2819281928193 0.138866447503975 0.00267788533201505
19.3019301930193 0.138800813132774 0.00267409006835734
19.3219321932193 0.138735283085859 0.00267030441624918
19.3419341934193 0.138669857087946 0.00266652834104568
19.3619361936194 0.138604534864707 0.0026627618082599
19.3819381938194 0.13853931614276 0.00265900478356205
19.4019401940194 0.138474200649668 0.00265525723277859
19.4219421942194 0.138409188113934 0.00265151912189144
19.4419441944194 0.138344278264997 0.00264779041703709
19.4619461946195 0.138279470833229 0.00264407108450574
19.4819481948195 0.138214765549931 0.00264036109074054
19.5019501950195 0.138150162147328 0.00263666040233667
19.5219521952195 0.138085660358564 0.00263296898604059
19.5419541954195 0.138021259917702 0.00262928680874919
19.5619561956196 0.137956960559718 0.00262561383750896
19.5819581958196 0.137892762020496 0.00262195003951521
19.6019601960196 0.137828664036825 0.00261829538211125
19.6219621962196 0.137764666346398 0.00261464983278759
19.6419641964196 0.137700768687803 0.00261101335918117
19.6619661966197 0.137636970800525 0.00260738592907454
19.6819681968197 0.137573272424935 0.00260376751039509
19.7019701970197 0.137509673302296 0.00260015807121427
19.7219721972197 0.13744617317475 0.00259655757974683
19.7419741974197 0.13738277178532 0.00259296600435001
19.7619761976198 0.137319468877904 0.00258938331352283
19.7819781978198 0.137256264197272 0.00258580947590528
19.8019801980198 0.137193157489064 0.00258224446027761
19.8219821982198 0.137130148499783 0.00257868823555955
19.8419841984198 0.137067236976792 0.00257514077080956
19.8619861986199 0.137004422668316 0.00257160203522411
19.8819881988199 0.13694170532343 0.00256807199813694
19.9019901990199 0.136879084692061 0.00256455062901833
19.9219921992199 0.136816560524983 0.00256103789747434
19.9419941994199 0.136754132573815 0.00255753377324613
19.96199619962 0.136691800591014 0.00255403822620923
19.98199819982 0.136629564329874 0.00255055122637282
20.00200020002 0.136567423544524 0.002547072743879
20.02200220022 0.136505377989921 0.00254360274900214
20.04200420042 0.136443427421848 0.00254014121214812
20.0620062006201 0.136381571596914 0.00253668810385367
20.0820082008201 0.136319810272543 0.00253324339478567
20.1020102010201 0.13625814320698 0.00252980705574045
20.1220122012201 0.136196570159279 0.00252637905764312
20.1420142014201 0.136135090889306 0.0025229593715469

20.1620162016202 0.136073705157733 0.00251954796863241
20.1820182018202 0.136012412726035 0.00251614482020704
20.2020202020202 0.135951213356485 0.00251274989770424
20.2220222022202 0.135890106812155 0.0025093631726829
20.2420242024202 0.135829092856908 0.00250598461682667
20.2620262026203 0.135768171255399 0.00250261420194329
20.2820282028203 0.135707341773068 0.00249925189996397
20.3020302030203 0.13564660417614 0.00249589768294272
20.3220322032203 0.135585958231619 0.00249255152305574
20.3420342034203 0.135525403707288 0.00248921339260072
20.3620362036204 0.135464940371703 0.00248588326399626
20.3820382038204 0.135404567994191 0.00248256110978122
20.4020402040204 0.135344286344846 0.0024792469026141
20.4220422042204 0.135284095194528 0.00247594061527239
20.4420442044204 0.135223994314859 0.00247264222065198
20.4620462046205 0.135163983478218 0.00246935169176654
20.4820482048205 0.135104062457741 0.00246606900174689
20.5020502050205 0.135044231027315 0.00246279412384041
20.5220522052205 0.134984488961577 0.0024595270314104
20.5420542054205 0.134924836035912 0.00245626769793555
20.5620562056206 0.134865272026446 0.00245301609700926
20.5820582058206 0.134805796710046 0.0024497722023391
20.6020602060206 0.134746409864317 0.00244653598774621
20.6220622062206 0.134687111267598 0.00244330742716468
20.6420642064206 0.134627900698961 0.00244008649464102
20.6620662066207 0.134568777938205 0.00243687316433353
20.6820682068207 0.134509742765855 0.00243366741051177
20.7020702070207 0.134450794963158 0.00243046920755593
20.7220722072207 0.134391934312083 0.00242727852995632
20.7420742074207 0.134333160595315 0.00242409535231278
20.7620762076208 0.134274473596252 0.00242091964933408
20.7820782078208 0.134215873099004 0.00241775139583743
20.8020802080208 0.134157358888391 0.00241459056674786
20.8220822082208 0.134098930749936 0.00241143713709773
20.8420842084208 0.134040588469868 0.00240829108202611
20.8620862086209 0.133982331835113 0.00240515237677828
20.8820882088209 0.133924160633295 0.0024020209967052
20.9020902090209 0.133866074652735 0.0023988969172629
20.9220922092209 0.133808073682443 0.00239578011401204
20.9420942094209 0.133750157512117 0.0023926705626173
20.962096209621 0.133692325932146 0.00238956823884687
20.982098209821 0.133634578733598 0.00238647311857194
21.002100210021 0.133576915708224 0.00238338517776618
21.022102210221 0.133519336648452 0.00238030439250517

21.042104210421 0.133461841347386 0.00237723073896595
21.0621062106211 0.133404429598804 0.00237416419342645
21.0821082108211 0.133347101197153 0.002371104732265
21.1021102110211 0.133289855937547 0.00236805233195984
21.1221122112211 0.133232693615766 0.00236500696908857
21.1421142114211 0.133175614028252 0.00236196862032771
21.1621162116212 0.133118616972106 0.00235893726245212
21.1821182118212 0.133061702245087 0.00235591287233457
21.2021202120212 0.133004869645607 0.00235289542694522
21.2221222122212 0.132948118972733 0.00234988490335113
21.2421242124212 0.132891450026178 0.00234688127871577
21.2621262126213 0.132834862606304 0.00234388453029854
21.2821282128213 0.132778356514116 0.00234089463545429
21.3021302130213 0.132721931551262 0.00233791157163283
21.3221322132213 0.132665587520029 0.00233493531637845
21.3421342134213 0.132609324223339 0.00233196584732948
21.3621362136214 0.132553141464751 0.00232900314221776
21.3821382138214 0.132497039048455 0.00232604717886824
21.4021402140214 0.132441016779268 0.00232309793519846
21.4221422142214 0.132385074462638 0.0023201553892181
21.4421442144214 0.132329211904634 0.00231721951902854
21.4621462146215 0.13227342891195 0.00231429030282237
21.4821482148215 0.132217725291896 0.00231136771888298
21.5021502150215 0.132162100852403 0.00230845174558406
21.5221522152215 0.132106555402014 0.00230554236138916
21.5421542154215 0.132051088749886 0.00230263954485129
21.5621562156216 0.131995700705786 0.0022997432746124
21.5821582158216 0.131940391080088 0.00229685352940301
21.6021602160216 0.131885159683772 0.0022939702880417
21.6221622162216 0.131830006328421 0.00229109352943475
21.6421642164216 0.131774930826219 0.00228822323257562
21.6621662166217 0.131719932989946 0.00228535937654462
21.6821682168217 0.131665012632982 0.00228250194050837
21.7021702170217 0.131610169569297 0.00227965090371945
21.7221722172217 0.131555403613456 0.00227680624551597
21.7421742174217 0.13150071458061 0.0022739679453211
21.7621762176218 0.131446102286499 0.0022711359826427
21.7821782178218 0.131391566547448 0.00226831033707287
21.8021802180218 0.131337107180363 0.00226549098828756
21.8221822182218 0.13128272400273 0.00226267791604614
21.8421842184218 0.131228416832615 0.00225987110019099
21.8621862186219 0.131174185488657 0.00225707052064712
21.8821882188219 0.131120029790072 0.00225427615742173
21.9021902190219 0.131065949556645 0.0022514879906038

21.9221922192219 0.13101194460873 0.00224870600036373
21.9421942194219 0.13095801476725 0.00224593016695293
21.962196219622 0.130904159853691 0.00224316047070338
21.982198219822 0.130850379690102 0.00224039689202729
22.002200220022 0.130796674099094 0.0022376394114167
22.022202220222 0.130743042903834 0.00223488800944306
22.042204220422 0.130689485928048 0.00223214266675686
22.0622062206221 0.130636002996014 0.00222940336408724
22.0822082208221 0.130582593932563 0.00222667008224164
22.1022102210221 0.130529258563076 0.00222394280210536
22.1222122212221 0.130475996713482 0.00222122150464124
22.1422142214221 0.130422808210255 0.00221850617088923
22.1622162216222 0.130369692880414 0.00221579678196606
22.1822182218222 0.13031665055152 0.00221309331906484
22.2022202220222 0.130263681051671 0.0022103957634547
22.2222222222222 0.130210784209506 0.00220770409648043
22.2422242224222 0.130157959854196 0.0022050182995621
22.2622262226223 0.130105207815449 0.0022023383541947
22.2822282228223 0.130052527923502 0.00219966424194777
22.3022302230223 0.129999920009124 0.00219699594446506
22.3222322232223 0.129947383903608 0.00219433344346417
22.3422342234223 0.129894919438776 0.00219167672073616
22.3622362236224 0.129842526446972 0.00218902575814525
22.3822382238224 0.129790204761062 0.00218638053762841
22.4022402240224 0.129737954214431 0.00218374104119506
22.4222422242224 0.129685774640983 0.00218110725092671
22.4422442244224 0.129633665875135 0.00217847914897656
22.4622462246225 0.129581627751821 0.00217585671756926
22.4822482248225 0.129529660106486 0.00217323993900047
22.5022502250225 0.129477762775084 0.00217062879563656
22.5222522252225 0.129425935594078 0.0021680232699143
22.5422542254225 0.129374178400436 0.00216542334434046
22.5622562256226 0.129322491031632 0.00216282900149153
22.5822582258226 0.129270873325641 0.00216024022401336
22.6022602260226 0.129219325120939 0.00215765699462082
22.6222622262226 0.129167846256501 0.00215507929609752
22.6422642264226 0.129116436571799 0.00215250711129542
22.6622662266227 0.129065095906798 0.00214994042313454
22.6822682268227 0.129013824101958 0.00214737921460263
22.7022702270227 0.12896262099823 0.00214482346875486
22.7222722272227 0.128911486437054 0.00214227316871347
22.7422742274227 0.128860420260359 0.00213972829766748
22.7622762276228 0.128809422310557 0.00213718883887235
22.7822782278228 0.128758492430547 0.00213465477564971

22.8022802280228 0.128707630463708 0.00213212609138697
22.8222822282228 0.128656836253901 0.00212960276953708
22.8422842284228 0.128606109645465 0.0021270847936182
22.8622862286229 0.128555450483215 0.00212457214721336
22.8822882288229 0.128504858612443 0.00212206481397021
22.9022902290229 0.128454333878913 0.00211956277760065
22.9222922292229 0.128403876128862 0.0021170660218806
22.9422942294229 0.128353485208995 0.00211457453064962
22.962296229623 0.128303160966486 0.00211208828781068
22.982298229823 0.128252903248976 0.00210960727732984
23.002300230023 0.12820271190457 0.0021071314832359
23.022302230223 0.128152586781836 0.00210466088962021
23.042304230423 0.128102527729804 0.00210219548063628
23.0623062306231 0.128052534597963 0.00209973524049954
23.0823082308231 0.128002607236261 0.00209728015348703
23.1023102310231 0.1279527454951 0.00209483020393713
23.1223122312231 0.127902949225339 0.00209238537624925
23.1423142314231 0.127853218278288 0.00208994565488357
23.1623162316232 0.127803552505709 0.00208751102436072
23.1823182318232 0.127753951759815 0.00208508146926155
23.2023202320232 0.127704415893265 0.00208265697422681
23.2223222322232 0.127654944759164 0.00208023752395686
23.2423242324232 0.127605538211064 0.00207782310321144
23.2623262326233 0.127556196102958 0.00207541369680936
23.2823282328233 0.127506918289282 0.00207300928962822
23.3023302330233 0.12745770462491 0.00207060986660417
23.3223322332233 0.127408554965155 0.0020682154127316
23.3423342334233 0.127359469165769 0.00206582591306289
23.3623362336234 0.127310447082935 0.00206344135270816
23.3823382338234 0.127261488573273 0.00206106171683495
23.4023402340234 0.127212593493832 0.00205868699066801
23.4223422342234 0.127163761702094 0.00205631715948899
23.4423442344234 0.127114993055968 0.00205395220863623
23.4623462346235 0.12706628741379 0.00205159212350443
23.4823482348235 0.127017644634324 0.00204923688954446
23.5023502350235 0.126969064576756 0.00204688649226304
23.5223522352235 0.126920547100695 0.00204454091722252
23.5423542354235 0.126872092066171 0.00204220015004063
23.5623562356236 0.126823699333634 0.00203986417639018
23.5823582358236 0.126775368763952 0.00203753298199887
23.6023602360236 0.12672710021841 0.00203520655264897
23.6223622362236 0.126678893558707 0.00203288487417713
23.6423642364236 0.126630748646955 0.0020305679324741
23.6623662366237 0.126582665345681 0.00202825571348447

23.6823682368237 0.12653464351782 0.00202594820320646
23.7023702370237 0.126486683026716 0.00202364538769165
23.7223722372237 0.126438783736122 0.00202134725304474
23.7423742374237 0.126390945510197 0.00201905378542329
23.7623762376238 0.126343168213503 0.00201676497103752
23.7823782378238 0.126295451711007 0.00201448079615005
23.8023802380238 0.126247795868078 0.00201220124707564
23.8223822382238 0.126200200550484 0.00200992631018097
23.8423842384238 0.126152665624392 0.00200765597188441
23.8623862386239 0.126105190956369 0.00200539021865579
23.8823882388239 0.126057776413374 0.00200312903701613
23.9023902390239 0.126010421862765 0.00200087241353746
23.9223922392239 0.12596312717229 0.00199862033484254
23.9423942394239 0.12591589221009 0.00199637278760466
23.962396239624 0.125868716844697 0.00199412975854739
23.982398239824 0.125821600945031 0.00199189123444439
24.002400240024 0.125774544380401 0.00198965720211912
24.022402240224 0.125727547020501 0.00198742764844469
24.042404240424 0.12568060873541 0.00198520256034356
24.0624062406241 0.125633729395593 0.0019829819247874
24.0824082408241 0.125586908871893 0.00198076572879678
24.1024102410241 0.125540147035538 0.00197855395944102
24.1224122412241 0.125493443758133 0.00197634660383793
24.1424142414241 0.125446798911662 0.0019741436491536
24.1624162416242 0.125400212368486 0.00197194508260221
24.1824182418242 0.12535368400134 0.00196975089144577
24.2024202420242 0.125307213683335 0.00196756106299393
24.2224222422242 0.125260801287954 0.00196537558460376
24.2424242424242 0.125214446689052 0.00196319444367956
24.2624262426243 0.125168149760853 0.0019610176276726
24.2824282428243 0.125121910377952 0.00195884512408096
24.3024302430243 0.12507572841531 0.00195667692044927
24.3224322432243 0.125029603748254 0.00195451300436857
24.3424342434243 0.124983536252478 0.00195235336347604
24.3624362436244 0.124937525804036 0.0019501979854548
24.3824382438244 0.12489157227935 0.00194804685803375
24.4024402440244 0.124845675555198 0.00194589996898734
24.4224422442244 0.12479983550872 0.00194375730613532
24.4424442444244 0.124754052017415 0.00194161885734264
24.4624462446245 0.124708324959139 0.00193948461051915
24.4824482448245 0.124662654212105 0.00193735455361947
24.5024502450245 0.124617039654879 0.00193522867464273
24.5224522452245 0.124571481166381 0.00193310696163244
24.5424542454245 0.124525978625886 0.00193098940267624

24.5624562456246 0.124480531913017 0.00192887598590572
24.5824582458246 0.124435140907748 0.00192676669949624
24.6024602460246 0.124389805490402 0.00192466153166672
24.6224622462246 0.12434452554165 0.00192256047067944
24.6424642464246 0.124299300942509 0.00192046350483988
24.6624662466247 0.124254131574339 0.00191837062249649
24.6824682468247 0.124209017318848 0.00191628181204053
24.7024702470247 0.124163958058083 0.00191419706190586
24.7224722472247 0.124118953674434 0.00191211636056878
24.7424742474247 0.124074004050631 0.00191003969654781
24.7624762476248 0.124029109069744 0.00190796705840353
24.7824782478248 0.12398426861518 0.00190589843473837
24.8024802480248 0.123939482570683 0.00190383381419646
24.8224822482248 0.123894750820332 0.00190177318546342
24.8424842484248 0.123850073248543 0.00189971653726617
24.8624862486249 0.123805449740061 0.00189766385837279
24.8824882488249 0.123760880179967 0.0018956151375923
24.9024902490249 0.12371636445367 0.00189357036377448
24.9224922492249 0.123671902446913 0.00189152952580975
24.9424942494249 0.123627494045762 0.0018894926126289
24.962496249625 0.123583139136615 0.00188745961320299
24.982498249825 0.123538837606196 0.00188543051654314
25.002500250025 0.123494589341552 0.00188340531170035
25.022502250225 0.123450394230055 0.00188138398776535
25.042504250425 0.123406252159402 0.00187936653386842
25.0625062506251 0.123362163017609 0.0018773529391792
25.0825082508251 0.123318126693016 0.00187534319290653
25.1025102510251 0.123274143074279 0.00187333728429828
25.1225122512251 0.123230212050377 0.00187133520264118
25.1425142514251 0.123186333510603 0.00186933693726067
25.1625162516252 0.123142507344569 0.00186734247752068
25.1825182518252 0.123098733442199 0.00186535181282353
25.2025202520252 0.123055011693735 0.00186336493260971
25.2225222522252 0.12301134198973 0.00186138182635774
25.2425242524252 0.122967724221049 0.00185940248358401
25.2625262526253 0.122924158278871 0.00185742689384258
25.2825282528253 0.122880644054681 0.00185545504672508
25.3025302530253 0.122837181440276 0.00185348693186048
25.3225322532253 0.122793770327759 0.00185152253891497
25.3425342534253 0.12275041060954 0.0018495618575918
25.3625362536254 0.122707102178336 0.00184760487763111
25.3825382538254 0.122663844927168 0.00184565158880976
25.4025402540254 0.122620638749361 0.00184370198094117
25.4225422542254 0.122577483538542 0.00184175604387522

25.4425442544254 0.12253437918864 0.00183981376749801
25.4625462546255 0.122491325593886 0.00183787514173175
25.4825482548255 0.122448322648808 0.00183594015653462
25.5025502550255 0.122405370248233 0.00183400880190057
25.5225522552255 0.12236246828 7289 0.0018320810678592
25.5425542554255 0.122319616661397 0.00183015694447561
25.5625562556256 0.122276815266273 0.00182823642185022
25.5825582558256 0.122234063997932 0.00182631949011864
25.6025602560256 0.122191362752677 0.00182440613945151
25.6225622562256 0.122148711427107 0.00182249636005438
25.6425642564256 0.122106109918113 0.00182059014216751
25.6625662566257 0.122063558122875 0.00181868747606575
25.6825682568257 0.122021055938861 0.00181678835205841
25.7025702570257 0.121978603263832 0.00181489276048907
25.7225722572257 0.121936199995833 0.00181300069173549
25.7425742574257 0.121893846033198 0.0018111121362094
25.7625762576258 0.121851541274544 0.0018092270843564
25.7825782578258 0.121809285618775 0.00180734552665582
25.8025802580258 0.121767078965079 0.00180546745362054
25.8225822582258 0.121724921212925 0.00180359285579688
25.8425842584258 0.121682812262065 0.00180172172376444
25.8625862586259 0.121640752012532 0.00179985404813599
25.8825882588259 0.121598740364639 0.00179798981955728
25.9025902590259 0.121556777218977 0.00179612902870693
25.9225922592259 0.121514862476417 0.00179427166629629
25.9425942594259 0.121472996038105 0.00179241772306932
25.962596259626 0.121431177805464 0.00179056718980241
25.982598259826 0.121389407680195 0.00178872005730426
26.002600260026 0.121347685564269 0.00178687631641576
26.022602260226 0.121306011359933 0.00178503595800985
26.042604260426 0.121264384969706 0.00178319897299137
26.0626062606261 0.121222806296379 0.00178136535229692
26.0826082608261 0.121181275243013 0.00177953508689478
26.1026102610261 0.12113979171294 0.00177770816778468
26.1226122612261 0.12109835560976 0.00177588458599779
26.1426142614261 0.121056966837341 0.00177406433259647
26.1626162616262 0.121015625299819 0.00177224739867422
26.1826182618262 0.120974330901596 0.00177043377535553
26.2026202620262 0.120933083547338 0.00176862345379571
26.2226222622262 0.120891883141977 0.00176681642518082
26.2426242624262 0.120850729590709 0.00176501268072751
26.2626262626263 0.120809622798991 0.00176321221168289
26.2826282628263 0.120768562672543 0.00176141500932442
26.3026302630263 0.120727549117345 0.00175962106495976

26.3226322632263 0.12068658203964 0.00175783036992669
26.3426342634263 0.120645661345926 0.0017560429155929
26.3626362636264 0.120604786942963 0.00175425869335595
26.3826382638264 0.120563958737766 0.00175247769464312
26.4026402640264 0.120523176637609 0.00175069991091126
26.4226422642264 0.12048244055002 0.0017489253336467
26.4426442644264 0.120441750382782 0.0017471539543651
26.4626462646265 0.120401106043934 0.00174538576461136
26.4826482648265 0.120360507441766 0.00174362075595946
26.5026502650265 0.120319954484822 0.00174185892001239
26.5226522652265 0.120279447081898 0.00174010024840197
26.5426542654265 0.120238985142039 0.00173834473278878
26.5626562656266 0.120198568574541 0.00173659236486201
26.5826582658266 0.12015819728895 0.00173484313633937
26.6026602660266 0.12011787119506 0.00173309703896694
26.6226622662266 0.12007759020291 0.00173135406451907
26.6426642664266 0.120037354222789 0.00172961420479828
26.6626662666267 0.119997163165231 0.00172787745163511
26.6826682668267 0.119957016941014 0.00172614379688802
26.7026702670267 0.119916915461162 0.00172441323244328
26.7226722672267 0.119876858636941 0.00172268575021486
26.7426742674267 0.11983684637986 0.00172096134214429
26.7626762676268 0.11979687860167 0.00171924000020058
26.7826782678268 0.119756955214364 0.00171752171638008
26.8026802680268 0.119717076130173 0.00171580648270639
26.8226822682268 0.119677241261571 0.00171409429123022
26.8426842684268 0.119637450521268 0.00171238513402933
26.8626862686269 0.119597703822212 0.00171067900320833
26.8826882688269 0.11955800107759 0.00170897589089869
26.9026902690269 0.119518342200823 0.00170727578925852
26.9226922692269 0.119478727105571 0.00170557869047253
26.9426942694269 0.119439155705725 0.00170388458675188
26.962696269627 0.119399627915413 0.00170219347033411
26.982698269827 0.119360143648994 0.00170050533348299
27.002700270027 0.119320702821062 0.00169882016848846
27.022702270227 0.119281305346441 0.00169713796766649
27.042704270427 0.119241951140188 0.00169545872335897
27.0627062706271 0.119202640117587 0.00169378242793364
27.0827082708271 0.119163372194156 0.00169210907378395
27.1027102710271 0.119124147285637 0.00169043865332896
27.1227122712271 0.119084965308004 0.00168877115901326
27.1427142714271 0.119045826177456 0.00168710658330685
27.1627162716272 0.11900672981042 0.00168544491870503
27.1827182718272 0.118967676123548 0.0016837861577283

27.2027202720272 0.118928665033718 0.00168213029292228
27.2227222722272 0.118889696458031 0.00168047731685757
27.2427242724272 0.118850770313813 0.00167882722212969
27.2627262726273 0.118811886518613 0.00167718000135895
27.2827282728273 0.1187730449902 0.00167553564719036
27.3027302730273 0.118734245646568 0.00167389415229352
27.3227322732273 0.118695488405929 0.00167225550936256
27.3427342734273 0.118656773186717 0.00167061971111598
27.3627362736274 0.118618099907584 0.0016689867502966
27.3827382738274 0.1185794684874 0.00166735661967143
27.4027402740274 0.118540878845256 0.00166572931203162
27.4227422742274 0.118502330900457 0.00166410482019229
27.4427442744274 0.118463824572526 0.00166248313699251
27.4627462746275 0.118425359781203 0.00166086425529515
27.4827482748275 0.118386936446441 0.00165924816798681
27.5027502750275 0.118348554488409 0.0016576348679777
27.5227522752275 0.118310213827489 0.00165602434820159
27.5427542754275 0.118271914384276 0.00165441660161568
27.5627562756276 0.118233656079578 0.00165281162120051
27.5827582758276 0.118195438834415 0.00165120939995987
27.6027602760276 0.118157262570017 0.00164960993092071
27.6227622762276 0.118119127207825 0.00164801320713306
27.6427642764276 0.118081032669489 0.00164641922166991
27.6627662766277 0.118042978876871 0.00164482796762713
27.6827682768277 0.118004965752037 0.00164323943812339
27.7027702770277 0.117966993217263 0.00164165362630005
27.7227722772277 0.117929061195033 0.00164007052532111
27.7427742774277 0.117891169608035 0.00163849012837305
27.7627762776278 0.117853318379165 0.0016369124286648
27.7827782778278 0.117815507431522 0.00163533741942764
27.8027802780278 0.117777736688411 0.00163376509391508
27.8227822782278 0.11774000607334 0.00163219544540282
27.8427842784278 0.11770231551002 0.00163062846718861
27.8627862786279 0.117664664922365 0.0016290641525922
27.8827882788279 0.117627054234491 0.00162750249495526
27.9027902790279 0.117589483370714 0.00162594348764125
27.9227922792279 0.117551952255552 0.00162438712403537
27.9427942794279 0.117514460813722 0.00162283339754445
27.962796279628 0.11747700897014 0.0016212823015969
27.982798279828 0.117439596649921 0.00161973382964259
28.002800280028 0.117402223778379 0.00161818797515278
28.022802280228 0.117364890281024 0.00161664473162001
28.042804280428 0.117327596083562 0.00161510409255808
28.0628062806281 0.117290341111898 0.0016135660515019

28.0828082808281	0.117253125292131	0.00161203060200743
28.1028102810281	0.117215948550553	0.00161049773765161
28.1228122812281	0.117178810813654	0.00160896745203227
28.1428142814281	0.117141712008114	0.00160743973876804
28.1628162816282	0.117104652060808	0.00160591459149826
28.1828182818282	0.117067630898804	0.00160439200388293
28.2028202820282	0.11703064844936	0.0016028719696026
28.2228222822282	0.116993704639927	0.00160135448235832
28.2428242824282	0.116956799398145	0.0015998395358715
28.2628262826283	0.116919932651846	0.00159832712388392
28.2828282828283	0.116883104329049	0.00159681724015757
28.3028302830283	0.116846314357963	0.0015953098784746
28.3228322832283	0.116809562666986	0.00159380503263725
28.3428342834283	0.116772849184703	0.00159230269646778
28.3628362836284	0.116736173839885	0.00159080286380835
28.3828382838284	0.116699536561492	0.00158930552852099
28.4028402840284	0.116662937278666	0.00158781068448748
28.4228422842284	0.116626375920739	0.00158631832560931
28.4428442844284	0.116589852417224	0.00158482844580759
28.4628462846285	0.116553366697819	0.00158334103902296
28.4828482848285	0.116516918692407	0.00158185609921554
28.5028502850285	0.116480508331052	0.00158037362036482
28.5228522852285	0.116444135544003	0.00157889359646962
28.5428542854285	0.116407800261687	0.00157741602154799
28.5628562856286	0.116371502414716	0.00157594088963716
28.5828582858286	0.11633524193388	0.00157446819479343
28.6028602860286	0.116299018750151	0.00157299793109214
28.6228622862286	0.11626283279468	0.00157153009262755
28.6428642864286	0.116226683998796	0.0015700646735128
28.6628662866287	0.116190572294009	0.00156860166787984
28.6828682868287	0.116154497612003	0.00156714106987932
28.7028702870287	0.116118459884642	0.00156568287368056
28.7228722872287	0.116082459043968	0.00156422707347145
28.7428742874287	0.116046495022195	0.0015627736634584
28.7628762876288	0.116010567751717	0.00156132263786624
28.7828782878288	0.115974677165102	0.00155987399093818
28.8028802880288	0.115938823195089	0.00155842771693572
28.8228822882288	0.115903005774597	0.00155698381013859
28.8428842884288	0.115867224836714	0.00155554226484467
28.8628862886289	0.115831480314703	0.00155410307536992
28.8828882888289	0.115795772141999	0.00155266623604834
28.9028902890289	0.115760100252209	0.00155123174123184
28.9228922892289	0.11572446457911	0.00154979958529024
28.9428942894289	0.115688865056652	0.00154836976261114

28.962896289629 0.115653301618954 0.00154694226759992
28.982898289829 0.115617774200305 0.00154551709467959
29.002900290029 0.115582282735164 0.0015440942382908
29.022902290229 0.115546827158157 0.00154267369289172
29.042904290429 0.115511407404081 0.00154125545295799
29.0629062906291 0.115476023407897 0.00153983951298268
29.0829082908291 0.115440675104736 0.00153842586747615
29.1029102910291 0.115405362429896 0.00153701451096609
29.1229122912291 0.115370085318838 0.00153560543799735
29.1429142914291 0.115334843707191 0.00153419864313194
29.1629162916292 0.11529963753075 0.00153279412094895
29.1829182918292 0.115264466725472 0.00153139186604446
29.2029202920292 0.115229331227479 0.00152999187303153
29.2229222922292 0.115194230973059 0.00152859413654006
29.2429242924292 0.115159165898659 0.00152719865121681
29.2629262926293 0.115124135940892 0.00152580541172526
29.2829282928293 0.115089141036531 0.0015244144127456
29.3029302930293 0.115054181122511 0.00152302564897462
29.3229322932293 0.115019256135929 0.00152163911512571
29.3429342934293 0.114984366014043 0.00152025480592875
29.3629362936294 0.114949510694268 0.00151887271613004
29.3829382938294 0.114914690114183 0.00151749284049227
29.4029402940294 0.114879904211522 0.00151611517379444
29.4229422942294 0.11484515292418 0.00151473971083182
29.4429442944294 0.114810436190211 0.00151336644641585
29.4629462946295 0.114775753947824 0.00151199537537412
29.4829482948295 0.114741106135386 0.00151062649255026
29.5029502950295 0.114706492691423 0.00150925979280393
29.5229522952295 0.114671913554614 0.00150789527101076
29.5429542954295 0.114637368663796 0.00150653292206222
29.5629562956296 0.11460285795796 0.00150517274086565
29.5829582958296 0.114568381376253 0.00150381472234414
29.6029602960296 0.114533938857974 0.0015024588614365
29.6229622962296 0.11449953034258 0.00150110515309718
29.6429642964296 0.114465155769676 0.00149975359229623
29.6629662966297 0.114430815079025 0.00149840417401924
29.6829682968297 0.114396508210538 0.00149705689326727
29.7029702970297 0.114362235104283 0.0014957117450568
29.7229722972297 0.114327995700474 0.00149436872441966
29.7429742974297 0.114293789939481 0.001493027826403
29.7629762976298 0.114259617761821 0.00149168904606921
29.7829782978298 0.114225479108163 0.00149035237849586
29.8029802980298 0.114191373919325 0.00148901781877566
29.8229822982298 0.114157302136275 0.00148768536201639

29.8429842984298 0.114123263700129 0.00148635500334087
29.8629862986299 0.114089258552151 0.00148502673788686
29.8829882988299 0.114055286633754 0.00148370056080704
29.9029902990299 0.114021347886497 0.00148237646726892
29.9229922992299 0.113987442252089 0.00148105445245485
29.9429942994299 0.113953569672382 0.00147973451156188
29.96299629963 0.113919730089375 0.00147841663980177
29.98299829983 0.113885923445214 0.00147710083240091
30.00300030003 0.113852149682189 0.00147578708460026
30.02300230023 0.113818408742736 0.00147447539165532
30.04300430043 0.113784700569433 0.00147316574883604
30.0630063006301 0.113751025105005 0.0014718581514268
30.0830083008301 0.113717382292318 0.00147055259472635
30.1030103010301 0.113683772074383 0.00146924907404773
30.1230123012301 0.113650194394351 0.00146794758471826
30.1430143014301 0.113616649195517 0.00146664812207944
30.1630163016302 0.113583136421318 0.00146535068148695
30.1830183018302 0.113549656015331 0.00146405525831056
30.2030203020302 0.113516207921276 0.00146276184793407
30.2230223022302 0.113482792083011 0.00146147044575529
30.2430243024302 0.113449408444536 0.00146018104718599
30.2630263026303 0.113416056949989 0.00145889364765182
30.2830283028303 0.113382737543648 0.00145760824259225
30.3030303030303 0.11334945016993 0.00145632482746059
30.3230323032303 0.113316194773391 0.00145504339772384
30.3430343034303 0.113282971298723 0.00145376394886273
30.3630363036304 0.113249779690757 0.00145248647637161
30.3830383038304 0.113216619894461 0.00145121097575841
30.4030403040304 0.11318349185494 0.00144993744254463
30.4230423042304 0.113150395517433 0.00144866587226524
30.4430443044304 0.113117330827319 0.00144739626046865
30.4630463046305 0.113084297730109 0.00144612860271667
30.4830483048305 0.11305129617145 0.00144486289458445
30.5030503050305 0.113018326097124 0.00144359913166043
30.5230523052305 0.112985387453048 0.0014423373095463
30.5430543054305 0.112952480185273 0.00144107742385694
30.5630563056306 0.11291960423998 0.0014398194702204
30.5830583058306 0.112886759563488 0.0014385634442778
30.6030603060306 0.112853946102245 0.00143730934168334
30.6230623062306 0.112821163802834 0.00143605715810421
30.6430643064306 0.112788412611968 0.00143480688922056
30.6630663066307 0.112755692476491 0.00143355853072545
30.6830683068307 0.112723003343381 0.00143231207832482
30.7030703070307 0.112690345159743 0.0014310675277374

30.7230723072307 0.112657717872816 0.00142982487469471
30.7430743074307 0.112625121429967 0.001428584114941
30.7630763076308 0.112592555778691 0.00142734524423318
30.7830783078308 0.112560020866616 0.0014261082583408
30.8030803080308 0.112527516641496 0.001424873153046
30.8230823082308 0.112495043051213 0.00142363992414346
30.8430843084308 0.11246260004378 0.00142240856744035
30.8630863086309 0.112430187567334 0.00142117907875629
30.8830883088309 0.112397805570142 0.00141995145392331
30.9030903090309 0.112365454000597 0.0014187256887858
30.9230923092309 0.112333132807218 0.00141750177920047
30.9430943094309 0.112300841938651 0.00141627972103629
30.963096309631 0.112268581343667 0.00141505951017447
30.983098309831 0.112236350971163 0.00141384114250839
31.003100310031 0.11220415077016 0.00141262461394358
31.023102310231 0.112171980689805 0.00141140992039765
31.043104310431 0.112139840679369 0.00141019705780028
31.0631063106311 0.112107730688245 0.00140898602209315
31.0831083108311 0.112075650665953 0.00140777680922992
31.1031103110311 0.112043600562134 0.00140656941517613
31.1231123112311 0.112011580326551 0.00140536383590924
31.1431143114311 0.111979589909091 0.00140416006741854
31.1631163116312 0.111947629259763 0.00140295810570511
31.1831183118312 0.111915698328698 0.00140175794678177
31.2031203120312 0.111883797066147 0.00140055958667307
31.2231223122312 0.111851925422483 0.00139936302141522
31.2431243124312 0.111820083348199 0.00139816824705605
31.2631263126313 0.111788270793909 0.00139697525965497
31.2831283128313 0.111756487710347 0.00139578405528297
31.3031303130313 0.111724734048367 0.00139459463002249
31.3231323132313 0.11169300975894 0.00139340697996746
31.3431343134313 0.111661314793158 0.00139222110122324
31.3631363136314 0.111629649102232 0.00139103698990655
31.3831383138314 0.11159801263749 0.00138985464214544
31.4031403140314 0.111566405350377 0.00138867405407929
31.4231423142314 0.111534827192457 0.00138749522185872
31.4431443144314 0.111503278115412 0.00138631814164556
31.4631463146315 0.111471758071037 0.00138514280961283
31.4831483148315 0.111440267011249 0.00138396922194469
31.5031503150315 0.111408804888077 0.0013827973748364
31.5231523152315 0.111377371653666 0.00138162726449425
31.5431543154315 0.111345967260279 0.00138045888713561
31.5631563156316 0.111314591660291 0.00137929223898876
31.5831583158316 0.111283244806195 0.00137812731629299

31.6031603160316	0.111251926650596	0.00137696411529844
31.6231623162316	0.111220637146214	0.00137580263226614
31.6431643164316	0.111189376245883	0.00137464286346795
31.6631663166317	0.111158143902549	0.00137348480518651
31.6831683168317	0.111126940069274	0.0013723284537152
31.7031703170317	0.11109576469923	0.00137117380535814
31.7231723172317	0.111064617745703	0.0013700208564301
31.7431743174317	0.11103349916209	0.0013688696032565
31.7631763176318	0.111002408901902	0.00136772004217334
31.7831783178318	0.110971346918758	0.00136657216952721
31.8031803180318	0.110940313166391	0.0013654259816752
31.8231823182318	0.110909307598644	0.0013642814749849
31.8431843184318	0.11087833016947	0.00136313864583436
31.8631863186319	0.110847380832934	0.00136199749061201
31.8831883188319	0.110816459543208	0.0013608580057167
31.9031903190319	0.110785566254575	0.00135972018755759
31.9231923192319	0.110754700921429	0.00135858403255416
31.9431943194319	0.110723863498269	0.00135744953713615
31.963196319632	0.110693053939707	0.00135631669774353
31.983198319832	0.110662272200459	0.00135518551082649
32.003200320032	0.110631518235353	0.00135405597284534
32.023202320232	0.110600791999321	0.00135292808027056
32.043204320432	0.110570093447405	0.00135180182958269
32.0632063206321	0.110539422534753	0.00135067721727233
32.0832083208321	0.11050877921662	0.00134955423984011
32.1032103210321	0.110478163448366	0.00134843289379662
32.1232123212321	0.11044757518546	0.00134731317566245
32.1432143214321	0.110417014383475	0.00134619508196803
32.1632163216322	0.110386480998088	0.00134507860925375
32.1832183218322	0.110355974985085	0.00134396375406978
32.2032203220322	0.110325496300353	0.00134285051297615
32.2232223222322	0.110295044899886	0.00134173888254264
32.2432243224322	0.110264620739782	0.00134062885934877
32.2632263226323	0.110234223776241	0.00133952043998379
32.2832283228323	0.110203853965571	0.00133841362104662
32.3032303230323	0.110173511264178	0.00133730839914582
32.3232323232323	0.110143195628575	0.00133620477089954
32.3432343234323	0.110112907015376	0.00133510273293554
32.3632363236324	0.110082645381298	0.0013340022818911
32.3832383238324	0.110052410683161	0.00133290341441302
32.4032403240324	0.110022202877885	0.00133180612715756
32.4232423242324	0.109992021922494	0.00133071041679044
32.4432443244324	0.109961867774111	0.00132961627998678
32.4632463246325	0.109931740389961	0.00132852371343109

32.4832483248325 0.109901639727371 0.00132743271381722
32.5032503250325 0.109871565743766 0.00132634327784832
32.5232523252325 0.109841518396672 0.00132525540223686
32.5432543254325 0.109811497643717 0.00132416908370451
32.5632563256326 0.109781503442626 0.00132308431898218
32.5832583258326 0.109751535751224 0.00132200110480999
32.6032603260326 0.109721594527435 0.00132091943793717
32.6232623262326 0.109691679729283 0.00131983931512211
32.6432643264326 0.109661791314888 0.00131876073313226
32.6632663266327 0.109631929242471 0.00131768368874415
32.6832683268327 0.109602093470348 0.00131660817874335
32.7032703270327 0.109572283956935 0.00131553419992438
32.7232723272327 0.109542500660744 0.00131446174909079
32.7432743274327 0.109512743540384 0.00131339082305503
32.7632763276328 0.109483012554562 0.00131232141863845
32.7832783278328 0.109453307662081 0.00131125353267129
32.8032803280328 0.109423628821838 0.00131018716199263
32.8232823282328 0.10939397599283 0.00130912230345037
32.8432843284328 0.109364349134147 0.00130805895390119
32.8632863286329 0.109334748204974 0.00130699711021052
32.8832883288329 0.109305173164593 0.00130593676925252
32.9032903290329 0.10927562397238 0.00130487792791004
32.9232923292329 0.109246100587804 0.0013038205830746
32.9432943294329 0.109216602970432 0.00130276473164635
32.963296329633 0.109187131079922 0.00130171037053406
32.983298329833 0.109157684876026 0.00130065749665505
33.003300330033 0.109128264318591 0.00129960610693521
33.023302330233 0.109098869367557 0.00129855619830894
33.043304330433 0.109069499982955 0.00129750776771914
33.0633063306331 0.109040156124911 0.00129646081211714
33.0833083308331 0.109010837753642 0.00129541532846274
33.1033103310331 0.108981544829459 0.00129437131372412
33.1233123312331 0.108952277312762 0.00129332876487784
33.1433143314331 0.108923035164046 0.00129228767890879
33.1633163316332 0.108893818343895 0.00129124805281022
33.1833183318332 0.108864626812986 0.00129020988358363
33.2033203320332 0.108835460532084 0.00128917316823879
33.2233223322332 0.108806319462048 0.00128813790379372
33.2433243324332 0.108777203563826 0.00128710408727462
33.2633263326333 0.108748112798455 0.00128607171571589
33.2833283328333 0.108719047127064 0.00128504078616007
33.3033303330333 0.10869000651087 0.00128401129565783
33.3233323332333 0.10866099091118 0.00128298324126792
33.3433343334333 0.10863200028939 0.00128195662005717

33.3633363336334 0.108603034606985 0.00128093142910046
33.3833383338334 0.108574093825538 0.00127990766548065
33.4033403340334 0.108545177906711 0.00127888532628863
33.4233423342334 0.108516286812255 0.00127786440862323
33.4433443344334 0.108487420504006 0.00127684490959119
33.4633463346335 0.108458578943891 0.0012758268263072
33.4833483348335 0.108429762093922 0.0012748101558938
33.5033503350335 0.108400969916199 0.00127379489548139
33.5233523352335 0.108372202372909 0.0012727810422082
33.5433543354335 0.108343459426325 0.00127176859322025
33.5633563356336 0.108314741038807 0.00127075754567136
33.5833583358336 0.108286047172802 0.00126974789672308
33.6033603360336 0.10825737779084 0.00126873964354467
33.6233623362336 0.108228732855541 0.00126773278331311
33.6433643364336 0.108200112329605 0.00126672731321304
33.6633663366337 0.108171516175823 0.00126572323043675
33.6833683368337 0.108142944357066 0.00126472053218415
33.7033703370337 0.108114396836292 0.00126371921566273
33.7233723372337 0.108085873576545 0.00126271927808756
33.7433743374337 0.108057374540949 0.00126172071668127
33.7633763376338 0.108028899692717 0.00126072352867397
33.7833783378338 0.108000448995141 0.0012597277113033
33.8033803380338 0.1079720224116 0.00125873326181434
33.8233823382338 0.107943619905555 0.00125774017745964
33.8433843384338 0.10791524144055 0.00125674845549915
33.8633863386339 0.107886886980211 0.00125575809320022
33.8833883388339 0.107858556488249 0.00125476908783757
33.9033903390339 0.107830249928455 0.00125378143669325
33.9233923392339 0.107801967264703 0.00125279513705667
33.9433943394339 0.10777370846095 0.00125181018622449
33.963396339634 0.107745473481232 0.00125082658150067
33.983398339834 0.107717262289668 0.00124984432019641
34.003400340034 0.10768907485046 0.00124886339963013
34.023402340234 0.107660911127888 0.00124788381712746
34.043404340434 0.107632771086314 0.00124690557002121
34.0634063406341 0.107604654690181 0.00124592865565131
34.0834083408341 0.107576561904012 0.00124495307136486
34.1034103410341 0.107548492692409 0.00124397881451603
34.1234123412341 0.107520447020056 0.00124300588246611
34.1434143414341 0.107492424851714 0.00124203427258341
34.1634163416342 0.107464426152226 0.0012410639822433
34.1834183418342 0.107436450886514 0.00124009500882814
34.2034203420342 0.107408499019576 0.00123912734972731
34.2234223422342 0.107380570516491 0.00123816100233713

34.2434243424342 0.107352665342418 0.00123719596406087
34.2634263426343 0.107324783462592 0.00123623223230873
34.2834283428343 0.107296924842327 0.00123526980449778
34.3034303430343 0.107269089447014 0.001234308678052
34.3234323432343 0.107241277242122 0.00123334885040219
34.3434343434343 0.1072134881932 0.001232390318986
34.3634363436344 0.10718572226587 0.00123143308124788
34.3834383438344 0.107157979425834 0.00123047713463906
34.4034403440344 0.107130259638869 0.00122952247661754
34.4234423442344 0.107102562870831 0.00122856910464806
34.4434443444344 0.107074889087651 0.00122761701620207
34.4634463446345 0.107047238255335 0.00122666620875773
34.4834483448345 0.107019610339966 0.00122571667979985
34.5034503450345 0.106992005307704 0.00122476842681993
34.5234523452345 0.106964423124782 0.00122382144731606
34.5434543454345 0.106936863757511 0.00122287573879297
34.5634563456346 0.106909327172275 0.00122193129876196
34.5834583458346 0.106881813335534 0.0012209881247409
34.6034603460346 0.106854322213824 0.0012200462142542
34.6234623462346 0.106826853773751 0.00121910556483281
34.6434643464346 0.106799407982001 0.00121816617401417
34.6634663466347 0.106771984805331 0.00121722803934219
34.6834683468347 0.106744584210571 0.00121629115836726
34.7034703470347 0.106717206164628 0.0012153555286462
34.7234723472347 0.106689850634479 0.00121442114774224
34.7434743474347 0.106662517587176 0.00121348801322502
34.7634763476348 0.106635206989844 0.00121255612267055
34.7834783478348 0.106607918809681 0.00121162547366119
34.8034803480348 0.106580653013958 0.00121069606378564
34.8234823482348 0.106553409570017 0.00120976789063893
34.8434843484348 0.106526188445273 0.00120884095182234
34.8634863486349 0.106498989607215 0.00120791524494348
34.8834883488349 0.106471813023401 0.00120699076761617
34.9034903490349 0.106444658661462 0.00120606751746048
34.9234923492349 0.1064175264891 0.00120514549210268
34.9434943494349 0.10639041647409 0.00120422468917526
34.963496349635 0.106363328584276 0.00120330510631686
34.983498349835 0.106336262787573 0.00120238674117227
35.003500350035 0.106309219051969 0.00120146959139242
35.023502350235 0.10628219734552 0.00120055365463436
35.043504350435 0.106255197636353 0.00119963892856123
35.0635063506351 0.106228219892666 0.00119872541084223
35.0835083508351 0.106201264082726 0.00119781309915263
35.1035103510351 0.10617433017487 0.00119690199117374

35.1235123512351 0.106147418137505 0.00119599208459285
35.1435143514351 0.106120527939107 0.00119508337710329
35.1635163516352 0.106093659548222 0.00119417586640433
35.1835183518352 0.106066812933462 0.00119326955020124
35.2035203520352 0.106039988063512 0.00119236442620518
35.2235223522352 0.106013184907122 0.00119146049213325
35.2435243524352 0.105986403433114 0.00119055774570847
35.2635263526353 0.105959643610374 0.00118965618465971
35.2835283528353 0.10593290540786 0.00118875580672172
35.3035303530353 0.105906188794596 0.00118785660963509
35.3235323532353 0.105879493739673 0.00118695859114623
35.3435343534353 0.105852820212251 0.00118606174900737
35.3635363536354 0.105826168181557 0.0011851660809765
35.3835383538354 0.105799537616884 0.00118427158481742
35.4035403540354 0.105772928487593 0.00118337825829965
35.4235423542354 0.105746340763111 0.00118248609919845
35.4435443544354 0.105719774412934 0.00118159510529479
35.4635463546355 0.10569322940662 0.00118070527437535
35.4835483548355 0.105666705713797 0.00117981660423248
35.5035503550355 0.105640203304158 0.00117892909266418
35.5235523552355 0.105613722147461 0.00117804273747412
35.5435543554355 0.105587262213531 0.00117715753647156
35.5635563556356 0.105560823472257 0.00117627348747138
35.5835583558356 0.105534405893594 0.00117539058829405
35.6035603560356 0.105508009447564 0.00117450883676562
35.6235623562356 0.10548163410425 0.00117362823071767
35.6435643564356 0.105455279833803 0.00117274876798734
35.6635663566357 0.105428946606438 0.00117187044641726
35.6835683568357 0.105402634392433 0.00117099326385559
35.7035703570357 0.105376343162133 0.00117011721815595
35.7235723572357 0.105350072885944 0.00116924230717743
35.7435743574357 0.105323823534339 0.00116836852878457
35.7635763576358 0.105297595077851 0.00116749588084734
35.7835783578358 0.10527138748708 0.00116662436124114
35.8035803580358 0.105245200732688 0.00116575396784674
35.8235823582358 0.1052190347854 0.00116488469855031
35.8435843584358 0.105192889616005 0.00116401655124335
35.8635863586359 0.105166765195354 0.00116314952382276
35.8835883588359 0.105140661494361 0.00116228361419071
35.9035903590359 0.105114578484002 0.00116141882025473
35.9235923592359 0.105088516135317 0.00116055513992761
35.9435943594359 0.105062474419407 0.00115969257112744
35.963596359636 0.105036453307434 0.00115883111177756
35.983598359836 0.105010452770624 0.00115797075980657

36.003600360036 0.104984472780264 0.00115711151314828
36.023602360236 0.104958513307702 0.00115625336974172
36.043604360436 0.104932574324348 0.00115539632753113
36.0636063606361 0.104906655801673 0.0011545403844659
36.0836083608361 0.104880757711208 0.0011536855385006
36.1036103610361 0.104854880024546 0.00115283178759497
36.1236123612361 0.104829022713342 0.00115197912971383
36.1436143614361 0.104803185749309 0.00115112756282717
36.1636163616362 0.104777369104222 0.00115027708491003
36.1836183618362 0.104751572749916 0.00114942769394257
36.2036203620362 0.104725796658285 0.00114857938790999
36.2236223622362 0.104700040801284 0.00114773216480257
36.2436243624362 0.104674305150928 0.00114688602261561
36.2636263626363 0.104648589679291 0.00114604095934941
36.2836283628363 0.104622894358506 0.00114519697300931
36.3036303630363 0.104597219160766 0.00114435406160562
36.3236323632363 0.104571564058322 0.00114351222315363
36.3436343634363 0.104545929023487 0.00114267145567357
36.3636363636364 0.104520314028629 0.00114183175719063
36.3836383638364 0.104494719046177 0.00114099312573493
36.4036403640364 0.104469144048617 0.00114015555934148
36.4236423642364 0.104443589008494 0.00113931905605021
36.4436443644364 0.104418053898413 0.00113848361390592
36.4636463646365 0.104392538691033 0.00113764923095827
36.4836483648365 0.104367043359074 0.00113681590526177
36.5036503650365 0.104341567875313 0.00113598363487579
36.5236523652365 0.104316112212584 0.00113515241786449
36.5436543654365 0.104290676343778 0.00113432225229685
36.5636563656366 0.104265260241846 0.00113349313624664
36.5836583658366 0.104239863879792 0.0011326650677924
36.6036603660366 0.10421448723068 0.00113183804501745
36.6236623662366 0.10418913026763 0.00113101206600982
36.6436643664366 0.104163792963817 0.00113018712886231
36.6636663666367 0.104138475292475 0.00112936323167242
36.6836683668367 0.104113177226893 0.00112854037254235
36.7036703670367 0.104087898740417 0.001127718549579
36.7236723672367 0.104062639806447 0.00112689776089392
36.7436743674367 0.104037400398441 0.00112607800460334
36.7636763676368 0.104012180489912 0.00112525927882813
36.7836783678368 0.103986980054428 0.00112444158169379
36.8036803680368 0.103961799065614 0.00112362491133043
36.8236823682368 0.103936637497148 0.00112280926587278
36.8436843684368 0.103911495322766 0.00112199464346014
36.8636863686369 0.103886372516256 0.00112118104223638

36.8836883688369 0.103861269051463 0.00112036846034995
36.9036903690369 0.103836184902286 0.00111955689595383
36.9236923692369 0.103811120042678 0.00111874634720553
36.9436943694369 0.103786074446647 0.0011179368122671
36.963696369637 0.103761048088255 0.00111712828930506
36.983698369837 0.103736040941619 0.00111632077649044
37.003700370037 0.103711052980909 0.00111551427199876
37.023702370237 0.103686084180349 0.00111470877400998
37.043704370437 0.103661134514217 0.0011139042807085
37.0637063706371 0.103636203956844 0.00111310079028319
37.0837083708371 0.103611292482615 0.00111229830092732
37.1037103710371 0.103586400065967 0.00111149681083857
37.1237123712371 0.103561526681393 0.00111069631821902
37.1437143714371 0.103536672303436 0.00110989682127511
37.1637163716372 0.103511836906692 0.0011090983182177
37.1837183718372 0.103487020465813 0.00110830080726193
37.2037203720372 0.103462222955499 0.00110750428662736
37.2237223722372 0.103437444350506 0.00110670875453781
37.2437243724372 0.10341268462564 0.00110591420922147
37.2637263726373 0.103387943755761 0.0011051206489108
37.2837283728373 0.10336322171578 0.00110432807184255
37.3037303730373 0.103338518480659 0.00110353647625776
37.3237323732373 0.103313834025413 0.00110274586040172
37.3437343734373 0.103289168325109 0.00110195622252397
37.3637363736374 0.103264521354864 0.00110116756087831
37.3837383738374 0.103239893089848 0.00110037987372273
37.4037403740374 0.10321528350528 0.00109959315931946
37.4237423742374 0.103190692576432 0.00109880741593491
37.4437443744374 0.103166120278626 0.00109802264183969
37.4637463746375 0.103141566587235 0.00109723883530856
37.4837483748375 0.103117031477683 0.00109645599462046
37.5037503750375 0.103092514925445 0.00109567411805849
37.5237523752375 0.103068016906043 0.00109489320390986
37.5437543754375 0.103043537395055 0.0010941132504659
37.5637563756376 0.103019076368103 0.00109333425602208
37.5837583758376 0.102994633800864 0.00109255621887794
37.6037603760376 0.102970209669063 0.00109177913733713
37.6237623762376 0.102945803948472 0.00109100300970735
37.6437643764376 0.102921416614918 0.00109022783430037
37.6637663766377 0.102897047644273 0.00108945360943202
37.6837683768377 0.102872697012461 0.00108868033342215
37.7037703770377 0.102848364695453 0.00108790800459464
37.7237723772377 0.10282405066927 0.0010871366212774
37.7437743774377 0.102799754909984 0.0010863661818023

37.7637763776378 0.102775477393712 0.00108559668450523
37.7837783778378 0.102751218096623 0.00108482812772606
37.8037803780378 0.102726976994933 0.0010840605098086
37.8237823782378 0.102702754064906 0.00108329382910064
37.8437843784378 0.102678549282856 0.0010825280839539
37.8637863786379 0.102654362625143 0.00108176327272401
37.8837883788379 0.102630194068177 0.00108099939377054
37.9037903790379 0.102606043588415 0.00108023644545696
37.9237923792379 0.102581911162362 0.00107947442615063
37.9437943794379 0.102557796766571 0.0010787133342228
37.963796379638 0.102533700377642 0.00107795316804857
37.983798379838 0.102509621972222 0.00107719392600694
38.003800380038 0.102485561527007 0.00107643560648071
38.023802380238 0.102461519018738 0.00107567820785654
38.043804380438 0.102437494424205 0.00107492172852492
38.0638063806381 0.102413487720245 0.00107416616688014
38.0838083808381 0.102389498883739 0.0010734115213203
38.1038103810381 0.102365527891617 0.00107265779024728
38.1238123812381 0.102341574720857 0.00107190497206675
38.1438143814381 0.10231763934848 0.00107115306518815
38.1638163816382 0.102293721751556 0.00107040206802467
38.1838183818382 0.102269821907199 0.00106965197899323
38.2038203820382 0.102245939792572 0.00106890279651452
38.2238223822382 0.10222207538488 0.00106815451901291
38.2438243824382 0.102198228661379 0.00106740714491654
38.2638263826383 0.102174399599365 0.00106666067265719
38.2838283828383 0.102150588176185 0.00106591510067037
38.3038303830383 0.102126794369227 0.00106517042739525
38.3238323832383 0.102103018155927 0.00106442665127468
38.3438343834383 0.102079259513766 0.00106368377075517
38.3638363836384 0.10205551842027 0.00106294178428685
38.3838383838384 0.102031794853008 0.00106220069032352
38.4038403840384 0.102008088789598 0.00106146048732259
38.4238423842384 0.101984400207699 0.00106072117374508
38.4438443844384 0.101960729085016 0.00105998274805562
38.4638463846385 0.1019370753993 0.00105924520872244
38.4838483848385 0.101913439128343 0.00105850855421733
38.5038503850385 0.101889820249986 0.00105777278301568
38.5238523852385 0.10186621874211 0.00105703789359643
38.5438543854385 0.101842634582642 0.00105630388444205
38.5638563856386 0.101819067749552 0.00105557075403859
38.5838583858386 0.101795518220857 0.00105483850087561
38.6038603860386 0.101771985974612 0.00105410712344617
38.6238623862386 0.101748470988922 0.00105337662024687

38.6438643864386 0.101724973241931 0.00105264698977781
38.6638663866387 0.101701492711829 0.00105191823054256
38.6838683868387 0.101678029376846 0.00105119034104816
38.7038703870387 0.10165458321526 0.00105046331980515
38.7238723872387 0.101631154205388 0.00104973716532752
38.7438743874387 0.101607742325592 0.00104901187613268
38.7638763876388 0.101584347554277 0.00104828745074151
38.7838783878388 0.101560969869889 0.0010475638876783
38.8038803880388 0.101537609250917 0.00104684118547077
38.8238823882388 0.101514265675896 0.00104611934265004
38.8438843884388 0.101490939123398 0.00104539835775062
38.8638863886389 0.101467629572041 0.00104467822931044
38.8838883888389 0.101444337000484 0.00104395895587077
38.9038903890389 0.101421061387429 0.00104324053597627
38.9238923892389 0.101397802711618 0.00104252296817495
38.9438943894389 0.101374560951837 0.00104180625101816
38.963896389639 0.101351336086913 0.00104109038306062
38.983898389839 0.101328128095714 0.00104037536286035
39.003900390039 0.10130493695715 0.0010396611889787
39.023902390239 0.101281762650173 0.00103894785998033
39.043904390439 0.101258605153776 0.00103823537443319
39.0639063906391 0.101235464446993 0.00103752373090854
39.0839083908391 0.101212340508898 0.00103681292798091
39.1039103910391 0.101189233318609 0.00103610296422809
39.1239123912391 0.101166142855282 0.00103539383823116
39.1439143914391 0.101143069098115 0.00103468554857442
39.1639163916392 0.101120012026348 0.00103397809384544
39.1839183918392 0.101096971619259 0.00103327147263501
39.2039203920392 0.101073947856167 0.00103256568353714
39.2239223922392 0.101050940716435 0.00103186072514908
39.2439243924392 0.10102795017946 0.00103115659607125
39.2639263926393 0.101004976224685 0.0010304532949073
39.2839283928393 0.10098201883159 0.00102975082026404
39.3039303930393 0.100959077979696 0.00102904917075147
39.3239323932393 0.100936153648563 0.00102834834498277
39.3439343934393 0.100913245817792 0.00102764834157425
39.3639363936394 0.100890354467022 0.00102694915914541
39.3839383938394 0.100867479575934 0.00102625079631885
39.4039403940394 0.100844621124245 0.00102555325172033
39.4239423942394 0.100821779091716 0.00102485652397873
39.4439443944394 0.100798953458143 0.00102416061172603
39.4639463946395 0.100776144203363 0.00102346551359734
39.4839483948395 0.100753351307252 0.00102277122823084
39.5039503950395 0.100730574749725 0.00102207775426781

39.5239523952395 0.100707814510736 0.00102138509035262
39.5439543954395 0.100685070570278 0.00102069323513269
39.5639563956396 0.100662342908381 0.00102000218725851
39.5839583958396 0.100639631505115 0.00101931194538362
39.6039603960396 0.100616936340589 0.0010186225081646
39.6239623962396 0.10059425739495 0.00101793387426109
39.6439643964396 0.100571594648381 0.00101724604233571
39.6639663966397 0.100548948081107 0.00101655901105414
39.6839683968397 0.100526317673388 0.00101587277908505
39.7039703970397 0.100503703405524 0.00101518734510011
39.7239723972397 0.100481105257851 0.00101450270777398
39.7439743974397 0.100458523210745 0.00101381886578432
39.7639763976398 0.100435957244617 0.00101313581781174
39.7839783978398 0.100413407339919 0.00101245356253983
39.8039803980398 0.100390873477138 0.00101177209865514
39.8239823982398 0.100368355636798 0.00101109142484716
39.8439843984398 0.100345853799463 0.00101041153980832
39.8639863986399 0.100323367945731 0.001009732442234
39.8839883988399 0.10030089805624 0.00100905413082249
39.9039903990399 0.100278444111664 0.00100837660427499
39.9239923992399 0.100256006092712 0.00100769986129562
39.9439943994399 0.100233583980132 0.00100702390059139
39.96399639964 0.10021117775471 0.00100634872087221
39.98399839984 0.100188787397265 0.00100567432085087
40.00400040004 0.100166412888655 0.00100500069924302
40.02400240024 0.100144054209774 0.0010043278547672
40.04400440044 0.100121711341553 0.00100365578614479
40.0640064006401 0.100099384264959 0.00100298449210003
40.0840084008401 0.100077072960993 0.00100231397136
40.1040104010401 0.100054777410695 0.00100164422265462
40.1240124012401 0.10003249759514 0.00100097524471661
40.1440144014401 0.100010233495438 0.00100030703628155
40.1640164016402 0.0999879850927374 9.99639596087787E-4
40.1840184018402 0.0999657523682196 9.98972922876503E-4
40.2040204020402 0.0999435353031027 9.98307015391654E-4
40.2240224022402 0.0999213338786405 9.97641872379995E-4
40.2440244024402 0.0998991480761222 9.96977492591054E-4
40.2640264026403 0.0998769778768721 9.96313874777124E-4
40.2840284028403 0.0998548232622501 9.95651017693273E-4
40.3040304030403 0.0998326842136514 9.94988920097316E-4
40.3240324032403 0.0998105607125058 9.94327580749813E-4
40.3440344034403 0.0997884527402786 9.93666998414074E-4
40.3640364036404 0.0997663602784699 9.93007171856128E-4
40.3840384038404 0.0997442833086145 9.92348099844735E-4

40.4040404040404 0.0997222218122822 9.91689781151373E-4
40.4240424042404 0.0997001757710775 9.91032214550229E-4
40.4440444044404 0.0996781451666389 9.9037539881818E-4
40.4640464046405 0.0996561299806403 9.89719332734815E-4
40.4840484048405 0.0996341301947894 9.89064015082396E-4
40.5040504050405 0.0996121457908282 9.88409444645864E-4
40.5240524052405 0.0995901767505333 9.87755620212837E-4
40.5440544054405 0.0995682230557153 9.87102540573595E-4
40.5640564056406 0.0995462846882189 9.86450204521072E-4
40.5840584058406 0.0995243616299226 9.85798610850852E-4
40.6040604060406 0.0995024538627389 9.85147758361158E-4
40.6240624062406 0.0994805613686142 9.84497645852849E-4
40.6440644064406 0.0994586841295285 9.83848272129412E-4
40.6640664066407 0.0994368221274957 9.83199635996953E-4
40.6840684068407 0.0994149753445628 9.82551736264186E-4
40.7040704070407 0.0993931437628107 9.81904571742432E-4
40.7240724072407 0.0993713273643534 9.8125814124561E-4
40.7440744074407 0.0993495261313385 9.80612443590232E-4
40.7640764076408 0.0993277400459465 9.79967477595384E-4
40.7840784078408 0.0993059690903911 9.79323242082734E-4
40.8040804080408 0.0992842132469195 9.78679735876523E-4
40.8240824082408 0.0992624724978113 9.78036957803546E-4
40.8440844084408 0.0992407468253792 9.77394906693155E-4
40.8640864086409 0.0992190362119686 9.76753581377243E-4
40.8840884088409 0.099197340639958 9.76112980690257E-4
40.9040904090409 0.0991756600917583 9.75473103469166E-4
40.9240924092409 0.0991539945498127 9.74833948553467E-4
40.9440944094409 0.0991323439965973 9.74195514785178E-4
40.964096409641 0.0991107084146205 9.73557801008829E-4
40.984098409841 0.0990890877864229 9.72920806071455E-4
41.004100410041 0.0990674820945773 9.72284528822587E-4
41.024102410241 0.0990458913216889 9.71648968114248E-4
41.044104410441 0.0990243154503947 9.71014122800946E-4
41.0641064106411 0.0990027544633641 9.70379991739674E-4
41.0841084108411 0.0989812083432978 9.69746573789879E-4
41.1041104110411 0.0989596770729289 9.69113867813485E-4
41.1241124112411 0.098938160635022 9.6848187267487E-4
41.1441144114411 0.0989166590123736 9.67850587240861E-4
41.1641164116412 0.0988951721878113 9.67220010380729E-4
41.1841184118412 0.098873700144195 9.66590140966185E-4
41.2041204120412 0.0988522428644151 9.6596097787136E-4
41.2241224122412 0.0988308003313943 9.65332519972823E-4
41.2441244124412 0.0988093725280859 9.64704766149546E-4
41.2641264126413 0.0987879594374748 9.64077715282921E-4

41.2841284128413 0.0987665610425769 9.63451366256738E-4
41.3041304130413 0.0987451773264392 9.62825717957188E-4
41.3241324132413 0.0987238082721395 9.62200769272847E-4
41.3441344134413 0.0987024538627868 9.61576519094679E-4
41.3641364136414 0.0986811140815207 9.60952966316022E-4
41.3841384138414 0.0986597889115115 9.60330109832588E-4
41.4041404140414 0.0986384783359605 9.59707948542448E-4
41.4241424142414 0.098617182338099 3 9.59086481346041E-4
41.4441444144414 0.09859590090119 9.58465707146142E-4
41.4641464146415 0.0985746340085255 9.57845624847888E-4
41.4841484148415 0.0985533816434286 9.57226233358738E-4
41.5041504150415 0.0985321437892527 9.56607531588494E-4
41.5241524152415 0.0985109204293813 9.5598951844928E-4
41.5441544154415 0.0984897115472281 9.55372192855535E-4
41.5641564156416 0.098468517126237 9.54755553724024E-4
41.5841584158416 0.0984473371498815 9.54139599973803E-4
41.6041604160416 0.0984261716016653 9.53524330526235E-4
41.6241624162416 0.0984050204651221 9.52909744304982E-4
41.6441644164416 0.0983838837238152 9.52295840235988E-4
41.6641664166417 0.0983627613613373 9.51682617247477E-4
41.6841684168417 0.0983416533613113 9.51070074269953E-4
41.7041704170417 0.0983205597073892 9.50458210236186E-4
41.7241724172417 0.0982994803832527 9.4984702408121E-4
41.7441744174417 0.098278415372613 9.4923651474232E-4
41.7641764176418 0.0982573646592103 9.48626681159056E-4
41.7841784178418 0.0982363282268142 9.48017522273199E-4
41.8041804180418 0.0982153060592239 9.47409037028783E-4
41.8241824182418 0.098194298140267 9.46801224372061E-4
41.8441844184418 0.0981733044538006 9.46194083251515E-4
41.8641864186419 0.0981523249837109 9.45587612617856E-4
41.8841884188419 0.0981313597139126 9.44981811423997E-4
41.9041904190419 0.0981104086283496 9.44376678625069E-4
41.9241924192419 0.0980894717109941 9.43772213178398E-4
41.9441944194419 0.0980685489458475 9.43168414043511E-4
41.964196419642 0.0980476403169395 9.42565280182126E-4
41.984198419842 0.0980267458083286 9.41962810558145E-4
42.004200420042 0.0980058654041015 9.41361004137646E-4
42.024202420242 0.0979849990883735 9.40759859888882E-4
42.0442044204421 0.0979641468452881 9.40159376782272E-4
42.0642064206421 0.0979433086590173 9.39559553790401E-4
42.0842084208421 0.0979224845137612 9.38960389888002E-4
42.1042104210421 0.0979016743937477 9.38361884051958E-4
42.1242124212421 0.0978808782832332 9.37764035261302E-4
42.1442144214421 0.0978600961665021 9.37166842497202E-4

42.1642164216422 0.0978393280278664 9.36570304742957E-4
42.1842184218422 0.0978185738516663 9.35974420983992E-4
42.2042204220422 0.0977978336222694 9.35379190207855E-4
42.2242224222422 0.0977771073240715 9.34784611404209E-4
42.2442244224422 0.0977563949414955 9.34190683564822E-4
42.2642264226423 0.0977356964589924 9.33597405683572E-4
42.2842284228423 0.0977150118610406 9.33004776756435E-4
42.3042304230423 0.0976943411321455 9.32412795781469E-4
42.3242324232423 0.0976736842568405 9.31821461758833E-4
42.3442344234423 0.097653041219686 9.31230773690758E-4
42.3642364236424 0.0976324120052696 9.30640730581553E-4
42.3842384238424 0.0976117965982064 9.30051331437603E-4
42.4042404240424 0.0975911949831383 9.29462575267349E-4
42.4242424242424 0.0975706071447343 9.28874461081292E-4
42.4442444244424 0.0975500330676904 9.28286987891993E-4
42.4642464246425 0.0975294727367298 9.2770015471406E-4
42.4842484248425 0.097508926136602 9.27113960564134E-4
42.5042504250425 0.0974883932520838 9.26528404460907E-4
42.5242524252425 0.0974678740679785 9.25943485425093E-4
42.5442544254425 0.0974473685691161 9.25359202479436E-4
42.5642564256426 0.0974268767403532 9.24775554648703E-4
42.5842584258426 0.0974063985665727 9.24192540959672E-4
42.6042604260426 0.0973859340326844 9.23610160441133E-4
42.6242624262426 0.0973654831236241 9.23028412123885E-4
42.6442644264426 0.0973450458243543 9.22447295040722E-4
42.6642664266427 0.0973246221198633 9.21866808226431E-4
42.6842684268427 0.0973042119951661 9.21286950717794E-4
42.7042704270427 0.0972838154353036 9.20707721553571E-4
42.7242724272427 0.0972634324253426 9.20129119774505E-4
42.7442744274427 0.0972430629503764 9.19551144423309E-4
42.7642764276428 0.0972227069955237 9.18973794544663E-4
42.7842784278428 0.0972023645459295 9.18397069185215E-4
42.8042804280428 0.0971820355867646 9.17820967393569E-4
42.8242824282428 0.0971617201032253 9.17245488220279E-4
42.8442844284428 0.0971414180805336 9.16670630717842E-4
42.8642864286429 0.0971211295039376 9.16096393940708E-4
42.8842884288429 0.0971008543587106 9.15522776945261E-4
42.9042904290429 0.0970805926301514 9.1494977878981E-4
42.9242924292429 0.0970603443035844 9.14377398534593E-4
42.9442944294429 0.0970401093643592 9.13805635241777E-4
42.964296429643 0.097019887797851 9.13234487975436E-4
42.984298429843 0.0969996795894602 9.12663955801567E-4
43.004300430043 0.0969794847246121 9.1209403778806E-4
43.024302430243 0.0969593031887576 9.11524733004718E-4

43.044304430443 0.0969391349673724 9.10956040523233E-4
43.0643064306431 0.0969189800459574 9.10387959417194E-4
43.0843084308431 0.0968988384100382 9.09820488762073E-4
43.1043104310431 0.0968787100451655 9.09253627635224E-4
43.1243124312431 0.0968585949369149 9.0868737511588E-4
43.1443144314431 0.0968384930708868 9.08121730285148E-4
43.1643164316432 0.0968184044327059 9.0755669222599E-4
43.1843184318432 0.0967983290080222 9.06992260023243E-4
43.2043204320432 0.0967782667825098 9.06428432763598E-4
43.2243224322432 0.0967582177418679 9.05865209535599E-4
43.2443244324433 0.0967381818718194 9.05302589429629E-4
43.2643264326433 0.0967181591581122 9.0474057153792E-4
43.2843284328433 0.0966981495865185 9.04179154954545E-4
43.3043304330433 0.0966781531428347 9.03618338775406E-4
43.3243324332433 0.0966581698128817 9.03058122098237E-4
43.3443344334433 0.096638199582504 9.02498504022584E-4
43.3643364336434 0.096618242437571 9.01939483649826E-4
43.3843384338434 0.0965982983639757 9.01381060083152E-4
43.4043404340434 0.096578367347635 9.00823232427551E-4
43.4243424342434 0.0965584493744902 9.00265999789828E-4
43.4443444344434 0.0965385444305064 8.99709361278582E-4
43.4643464346435 0.0965186525016723 8.99153316004212E-4
43.4843484348435 0.0964987735740005 8.985978630789E-4
43.5043504350435 0.0964789076335274 8.98043001616617E-4
43.5243524352435 0.0964590546663131 8.97488730733117E-4
43.5443544354435 0.0964392146584412 8.96935049545933E-4
43.5643564356436 0.0964193875960191 8.96381957174363E-4
43.5843584358436 0.0963995734651772 8.95829452739475E-4
43.6043604360436 0.09637977225207 8.95277535364101E-4
43.6243624362436 0.0963599839428749 8.94726204172832E-4
43.6443644364436 0.096340208523793 8.94175458292012E-4
43.6643664366437 0.0963204459810483 8.93625296849729E-4
43.6843684368437 0.0963006963008883 8.93075718975823E-4
43.7043704370437 0.0962809594695836 8.9252672380187E-4
43.7243724372437 0.0962612354734279 8.91978310461183E-4
43.7443744374437 0.0962415242987381 8.91430478088807E-4
43.7643764376438 0.0962218259318537 8.90883225821508E-4
43.7843784378438 0.0962021403591378 8.90336552797784E-4
43.8043804380438 0.0961824675669758 8.8979045815784E-4
43.8243824382438 0.0961628075417762 8.89244941043602E-4
43.8443844384438 0.0961431602699702 8.88700000598701E-4
43.8643864386439 0.0961235257380119 8.88155635968473E-4
43.8843884388439 0.0961039039323778 8.87611846299955E-4
43.9043904390439 0.0960842948395675 8.87068630741883E-4

43.9243924392439 0.0960646984461025 8.86525988444675E-4
43.9443944394439 0.0960451147385274 8.85983918560444E-4
43.964396439644 0.0960255437034091 8.85442420242988E-4
43.984398439844 0.0960059853273366 8.8490149264777E-4
44.004400440044 0.0959864395969216 8.8436113493194E-4
44.024402440244 0.0959669064987981 8.83821346254315E-4
44.044404440444 0.0959473860196222 8.83282125775376E-4
44.0644064406441 0.0959278781460723 8.82743472657263E-4
44.0844084408441 0.0959083828648487 8.82205386063774E-4
44.1044104410441 0.095888900162674 3 8.81667865160365E-4
44.1244124412441 0.0958694300262935 8.81130909114133E-4
44.1444144414441 0.095849972442473 8.80594517093822E-4
44.1644164416442 0.0958305273980014 8.8005868826982E-4
44.1844184418442 0.095811094879688 9 8.79523421814143E-4
44.2044204420442 0.095791674874368 8.78988716900446E-4
44.2244224422442 0.0957722673688928 8.7845457270401E-4
44.2444244424442 0.0957528723501388 8.77920988401734E-4
44.2644264426443 0.0957334898050037 8.77387963172142E-4
44.2844284428443 0.0957141197204063 8.7685549619537E-4
44.3044304430443 0.0956947620832876 8.76323586653171E-4
44.3244324432443 0.0956754168806094 8.75792233728894E-4
44.3444344434443 0.0956560840993556 8.75261436607502E-4
44.3644364436444 0.0956367637265311 8.74731194475549E-4
44.3844384438444 0.0956174557491625 8.74201506521188E-4
44.4044404440444 0.0955981601542974 8.73672371934159E-4
44.4244424442444 0.0955788769290048 8.73143789905791E-4
44.4444444444445 0.0955596060603751 8.72615759628997E-4
44.4644464446445 0.0955403475355195 8.72088280298267E-4
44.4844484448445 0.0955211013415707 8.71561351109662E-4
44.5044504450445 0.0955018674656822 8.71034971260819E-4
44.5244524452445 0.0954826458950286 8.70509139950939E-4
44.5444544454445 0.0954634366168056 8.69983856380786E-4
44.5644564456446 0.0954442396182295 8.69459119752682E-4
44.5844584458446 0.0954250548865378 8.68934929270504E-4
44.6044604460446 0.0954058824089887 8.68411284139681E-4
44.6244624462446 0.0953867221728612 8.6788818356719E-4
44.6444644464446 0.0953675741654549 8.67365626761545E-4
44.6644664466447 0.0953484383740903 8.66843612932808E-4
44.6844684468447 0.0953293147861082 8.66322141292564E-4
44.7044704470447 0.0953102033888703 8.65801211053941E-4
44.7244724472447 0.0952911041697586 8.65280821431589E-4
44.7444744474447 0.0952720171161758 8.64760971641684E-4
44.7644764476448 0.095252942215545 8.64241660901919E-4
44.7844784478448 0.0952338794553094 8.63722888431504E-4

44.8044804480448 0.0952148288229328 8.63204653451161E-4
44.8244824482448 0.0951957903058993 8.62686955183124E-4
44.8444844484448 0.095176763891713 8.62169792851124E-4
44.8644864486449 0.0951577495678985 8.61653165680398E-4
44.8844884488449 0.0951387473220006 8.61137072897683E-4
44.9044904490449 0.0951197571415837 8.60621513731201E-4
44.9244924492449 0.0951007790142326 8.60106487410668E-4
44.9444944494449 0.0950818129275522 8.59591993167287E-4
44.964496449645 0.0950628588691672 8.59078030233741E-4
44.984498449845 0.0950439168267223 8.58564597844194E-4
45.004500450045 0.0950249867878819 8.5805169523428E-4
45.024502450245 0.0950060687403305 8.57539321641109E-4
45.044504450445 0.094987162671772 8.57027476303255E-4
45.0645064506451 0.0949682685699303 8.56516158460754E-4
45.0845084508451 0.094949386422549 8.56005367355108E-4
45.1045104510451 0.0949305162173911 8.5549510222927E-4
45.1245124512451 0.0949116579422395 8.54985362327649E-4
45.1445144514451 0.0948928115848965 8.54476146896101E-4
45.1645164516452 0.0948739771331837 8.53967455181927E-4
45.1845184518452 0.0948551545749424 8.5345928643387E-4
45.2045204520452 0.0948363438980334 8.52951639902118E-4
45.2245224522452 0.0948175450903365 8.52444514838281E-4
45.2445244524452 0.0947987581397511 8.51937910495411E-4
45.2645264526453 0.0947799830341958 8.51431826127981E-4
45.2845284528453 0.0947612197616084 8.50926260991892E-4
45.3045304530453 0.0947424683099459 8.50421214344466E-4
45.3245324532453 0.0947237286671845 8.49916685444438E-4
45.3445344534453 0.0947050008213194 8.49412673551958E-4
45.3645364536454 0.0946862847603648 8.48909177928586E-4
45.3845384538454 0.0946675804723542 8.48406197837292E-4
45.4045404540454 0.0946488879453396 8.47903732542444E-4
45.4245424542454 0.0946302071673923 8.47401781309812E-4
45.4445444544454 0.0946115381266023 8.4690034340656E-4
45.4645464546455 0.0945928808110786 8.46399418101248E-4
45.4845484548455 0.0945742352089487 8.45899004663823E-4
45.5045504550455 0.0945556013083589 8.45399102365615E-4
45.5245524552455 0.0945369790974746 8.44899710479344E-4
45.5445544554455 0.0945183685644793 8.44400828279101E-4
45.5645564556456 0.0944997696975754 8.43902455040355E-4
45.5845584558456 0.0944811824849839 8.43404590039949E-4
45.6045604560456 0.0944626069149443 8.42907232556095E-4
45.6245624562456 0.0944440429757143 8.42410381868366E-4
45.6445644564457 0.0944254906555706 8.41914037257701E-4
45.6645664566457 0.0944069499428078 8.41418198006397E-4

45.6845684568457 0.0943884208257389 8.40922863398103E-4
45.7045704570457 0.0943699032926956 8.40428032717825E-4
45.7245724572457 0.0943513973320275 8.39933705251916E-4
45.7445744574457 0.0943329029321026 8.39439880288072E-4
45.7645764576458 0.0943144200813069 8.38946557115331E-4
45.7845784578458 0.0942959487680448 8.38453735024076E-4
45.8045804580458 0.0942774889807386 8.37961413306017E-4
45.8245824582458 0.0942590407078289 8.37469591254204E-4
45.8445844584458 0.0942406039377738 8.36978268163006E-4
45.8645864586459 0.09422217865905 8.36487443328126E-4
45.8845884588459 0.094203764860152 8.35997116046592E-4
45.9045904590459 0.0941853625295918 8.35507285616741E-4
45.9245924592459 0.0941669716558996 8.35017951338231E-4
45.9445944594459 0.0941485922276234 8.34529112512036E-4
45.964596459646 0.0941302242333289 8.34040768440437E-4
45.984598459846 0.0941118676615994 8.33552918427017E-4
46.004600460046 0.094093522501036 8.33065561776667E-4
46.024602460246 0.0940751887402579 8.32578697795582E-4
46.044604460446 0.094056866367901 8.32092325791246E-4
46.0646064606461 0.0940385553726195 8.31606445072438E-4
46.0846084608461 0.0940202557430849 8.31121054949231E-4
46.1046104610461 0.0940019674679859 8.30636154732981E-4
46.1246124612461 0.0939836905360293 8.30151743736336E-4
46.1446144614461 0.0939654249359386 8.29667821273215E-4
46.1646164616462 0.0939471706564553 8.29184386658823E-4
46.1846184618462 0.0939289276863376 8.28701439209634E-4
46.2046204620462 0.0939106960143616 8.28218978243403E-4
46.2246224622462 0.0938924756293203 8.27737003079142E-4
46.2446244624462 0.0938742665200237 8.27255513037133E-4
46.2646264626463 0.0938560686752997 8.26774507438929E-4
46.2846284628463 0.0938378820839926 8.26293985607329E-4
46.3046304630463 0.0938197067349641 8.25813946866397E-4
46.3246324632463 0.093801542617093 8.2533439054145E-4
46.3446344634463 0.093783389719275 8.24855315959051E-4
46.3646364636464 0.0937652480304227 8.24376722447015E-4
46.3846384638464 0.093747117539466 8.23898609334402E-4
46.4046404640464 0.0937289982353512 8.23420975951508E-4
46.4246424642464 0.0937108901070419 8.2294382162987E-4
46.4446444644464 0.0936927931435181 8.2246714570226E-4
46.4646464646465 0.0936747073337771 8.21990947502688E-4
46.4846484648465 0.0936566326668325 8.21515226366387E-4
46.5046504650465 0.0936385691317147 8.21039981629817E-4
46.5246524652465 0.0936205167174711 8.20565212630667E-4
46.5446544654465 0.0936024754131652 8.20090918707837E-4

46.5646564656466 0.0935844452078776 8.19617099201455E-4
46.5846584658466 0.0935664260907049 8.19143753452856E-4
46.6046604660466 0.0935484180507609 8.18670880804592E-4
46.6246624662466 0.0935304210771752 8.18198480600421E-4
46.6446644664466 0.0935124351590944 8.1772655218531E-4
46.6646664666467 0.0934944602856811 8.17255094905422E-4
46.6846684668467 0.0934764964461144 8.16784108108129E-4
46.7046704670467 0.09345854362959 8.16313591141998E-4
46.7246724672467 0.0934406018253196 8.1584354335679E-4
46.7446744674468 0.0934226710225309 8.15373964103451E-4
46.7646764676468 0.0934047512104686 8.1490485273413E-4
46.7846784678468 0.0933868423783929 8.14436208602148E-4
46.8046804680468 0.0933689445155803 8.13968031062015E-4
46.8246824682468 0.0933510576113236 8.13500319469426E-4
46.8446844684469 0.0933331816549315 8.13033073181244E-4
46.8646864686469 0.0933153166357288 8.12566291555514E-4
46.8846884688469 0.0932974625430564 8.1209997395145E-4
46.9046904690469 0.0932796193662709 8.11634119729437E-4
46.9246924692469 0.0932617870947451 8.11168728251021E-4
46.9446944694469 0.0932439657178676 8.10703798878919E-4
46.964696469647 0.0932261552250429 8.10239330977007E-4
46.984698469847 0.0932083556056911 8.09775323910312E-4
47.004700470047 0.0931905668492484 8.09311777045027E-4
47.024702470247 0.0931727889451665 8.08848689748485E-4
47.044704470447 0.0931550218829129 8.08386061389181E-4
47.0647064706471 0.0931372656519712 8.07923891336756E-4
47.0847084708471 0.0931195202418396 8.0746217896198E-4
47.1047104710471 0.0931017856420331 8.07000923636787E-4
47.1247124712471 0.0930840618420816 8.06540124734234E-4
47.1447144714471 0.0930663488315305 8.06079781628519E-4
47.1647164716472 0.093048646599941 8.05619893694972E-4
47.1847184718472 0.0930309551368898 8.05160460310061E-4
47.2047204720472 0.0930132744319687 8.04701480851373E-4
47.2247224722472 0.0929956044747851 8.04242954697623E-4
47.2447244724472 0.0929779452549619 8.03784881228654E-4
47.2647264726473 0.0929602967621369 8.03327259825421E-4
47.2847284728473 0.0929426589859639 8.02870089870006E-4
47.3047304730473 0.0929250319161112 8.02413370745596E-4
47.3247324732473 0.0929074155422629 8.01957101836499E-4
47.3447344734473 0.0928898098541179 8.01501282528125E-4
47.3647364736474 0.0928722148413907 8.01045912207E-4
47.3847384738474 0.0928546304938104 8.00590990260746E-4
47.4047404740474 0.0928370568011216 8.00136516078091E-4
47.4247424742474 0.0928194937530837 7.99682489048862E-4

47.4447444744474 0.0928019413394713 7.99228908563979E-4
47.4647464746475 0.0927843995500741 7.98775774015467E-4
47.4847484748475 0.0927668683746964 7.98323084796427E-4
47.5047504750475 0.0927493478031578 7.97870840301063E-4
47.5247524752475 0.0927318378252923 7.97419039924655E-4
47.5447544754475 0.0927143384309495 7.96967683063577E-4
47.5647564756476 0.0926968496099931 7.96516769115272E-4
47.5847584758476 0.092679371352302 7.96066297478274E-4
47.6047604760476 0.0926619036477699 7.95616267552188E-4
47.6247624762476 0.0926444464863048 7.95166678737687E-4
47.6447644764476 0.0926269998578299 7.94717530436528E-4
47.6647664766477 0.0926095637522828 7.94268822051525E-4
47.6847684768477 0.0925921381596156 7.93820552986562E-4
47.7047704770477 0.0925747230697955 7.93372722646593E-4
47.7247724772477 0.0925573184728037 7.92925330437628E-4
47.7447744774477 0.0925399243586362 7.92478375766729E-4
47.7647764776478 0.0925225407173035 7.9203185804203E-4
47.7847784778478 0.0925051675388307 7.91585776672707E-4
47.8047804780478 0.0924878048132569 7.91140131068991E-4
47.8247824782478 0.0924704525306361 7.9069492064216E-4
47.8447844784478 0.0924531106810362 7.90250144804541E-4
47.8647864786479 0.09243577925454 7.89805802969508E-4
47.8847884788479 0.0924184582412441 7.89361894551472E-4
47.9047904790479 0.0924011476312596 7.88918418965878E-4
47.9247924792479 0.0923838474147119 7.88475375629224E-4
47.944794479448 0.0923665575817406 7.8803276395903E-4
47.964796479648 0.0923492781224993 7.87590583373851E-4
47.984798479848 0.0923320090271558 7.87148833293268E-4
48.004800480048 0.0923147502858923 7.86707513137899E-4
48.024802480248 0.0922975018889048 7.86266622329378E-4
48.0448044804481 0.0922802638264034 7.85826160290366E-4
48.0648064806481 0.0922630360886123 7.85386126444541E-4
48.0848084808481 0.0922458186657697 7.84946520216601E-4
48.1048104810481 0.0922286115481277 7.84507341032261E-4
48.1248124812481 0.0922114147259524 7.84068588318249E-4
48.1448144814481 0.0921942281895238 7.83630261502298E-4
48.1648164816482 0.0921770519291357 7.83192360013157E-4
48.1848184818482 0.0921598859350956 7.82754883280573E-4
48.2048204820482 0.0921427301977254 7.82317830735308E-4
48.2248224822482 0.0921255847073601 7.81881201809116E-4
48.2448244824482 0.0921084494543488 7.81444995934755E-4
48.2648264826483 0.0920913244290543 7.81009212545978E-4
48.2848284828483 0.092074209621853 7.8057385107753E-4
48.3048304830483 0.0920571050231351 7.80138910965157E-4

48.3248324832483 0.0920400106233042 7.79704391645585E-4
48.3448344834483 0.0920229264127778 7.79270292556535E-4
48.3648364836484 0.0920058523819867 7.78836613136709E-4
48.3848384838484 0.0919887885213755 7.78403352825798E-4
48.4048404840484 0.091971734821402 7.77970511064467E-4
48.4248424842484 0.0919546912725377 7.77538087294361E-4
48.4448444844484 0.0919376578652677 7.77106080958108E-4
48.4648464846485 0.0919206345900902 7.76674491499305E-4
48.4848484848485 0.0919036214375168 7.76243318362518E-4
48.5048504850485 0.0918866183980728 7.75812560993292E-4
48.5248524852485 0.0918696254622966 7.75382218838133E-4
48.5448544854485 0.0918526426207399 7.74952291344513E-4
48.5648564856486 0.0918356698639677 7.74522777960868E-4
48.5848584858486 0.0918187071825584 7.74093678136597E-4
48.6048604860486 0.0918017545671033 7.73664991322054E-4
48.6248624862486 0.0917848120082073 7.73236716968551E-4
48.6448644864486 0.0917678794964882 7.72808854528361E-4
48.6648664866487 0.0917509570225768 7.72381403454696E-4
48.6848684868487 0.0917340445771174 7.7195436320173E-4
48.7048704870487 0.091717142150767 7.71527733224578E-4
48.7248724872487 0.091700249734196 7.71101512979308E-4
48.7448744874487 0.0916833673180875 7.70675701922922E-4
48.7648764876488 0.0916664948931379 7.70250299513372E-4
48.7848784878488 0.0916496324500563 7.69825305209549E-4
48.8048804880488 0.0916327799795649 7.69400718471273E-4
48.8248824882488 0.0916159374723986 7.68976538759306E-4
48.8448844884488 0.0915991049193053 7.68552765535341E-4
48.8648864886489 0.0915822823110461 7.68129398262005E-4
48.8848884888489 0.0915654696383942 7.67706436402848E-4
48.9048904890489 0.0915486668921364 7.67283879422356E-4
48.9248924892489 0.0915318740630715 7.66861726785929E-4
48.9448944894489 0.0915150911420118 7.66439977959896E-4
48.964896489649 0.0914983181197815 7.66018632411502E-4
48.984898489849 0.0914815549872184 7.65597689608919E-4
49.004900490049 0.091464801735172 7.65177149021222E-4
49.024902490249 0.0914480583545051 7.64757010118412E-4
49.044904490449 0.091431324836093 7.64337272371397E-4
49.0649064906491 0.0914146011708235 7.63917935251998E-4
49.0849084908491 0.0913978873495969 7.6349899823294E-4
49.1049104910491 0.091381183363326 7.63080460787856E-4
49.1249124912491 0.0913644892029362 7.62662322391284E-4
49.1449144914492 0.0913478048593654 7.62244582518663E-4
49.1649164916492 0.0913311303235638 7.61827240646331E-4
49.1849184918492 0.0913144655864942 7.61410296251528E-4

49.2049204920492 0.0912978106391315 7.6099374881238E-4
49.2249224922492 0.0912811654724634 7.60577597807922E-4
49.2449244924493 0.0912645300774893 7.60161842718065E-4
49.2649264926493 0.0912479044452218 7.59746483023625E-4
49.2849284928493 0.0912312885666851 7.59331518206296E-4
49.3049304930493 0.0912146824329157 7.58916947748657E-4
49.3249324932493 0.0911980860349624 7.58502771134176E-4
49.3449344934493 0.0911814993638866 7.58088987847203E-4
49.3649364936494 0.0911649224107614 7.57675597372965E-4
49.3849384938494 0.0911483551666722 7.5726259919757E-4
49.4049404940494 0.0911317976227165 7.56849992807998E-4
49.4249424942494 0.091115249770004 7.56437777692105E-4
49.4449444944494 0.0910987115996564 7.56025953338621E-4
49.4649464946495 0.0910821831028074 7.55614519237146E-4
49.4849484948495 0.0910656642706029 7.55203474878144E-4
49.5049504950495 0.0910491550942007 7.54792819752954E-4
49.5249524952495 0.0910326555647704 7.54382553353767E-4
49.5449544954495 0.0910161656734938 7.53972675173648E-4
49.5649564956496 0.0909996854115646 7.53563184706517E-4
49.5849584958496 0.0909832147701882 7.53154081447153E-4
49.6049604960496 0.0909667537405821 7.52745364891192E-4
49.6249624962496 0.0909503023139756 7.52337034535127E-4
49.6449644964496 0.0909338604816096 7.51929089876303E-4
49.6649664966497 0.0909174282347369 7.51521530412909E-4
49.6849684968497 0.0909010055646224 7.51114355643998E-4
49.7049704970497 0.0908845924625424 7.50707565069459E-4
49.7249724972497 0.0908681889197849 7.50301158190027E-4
49.7449744974497 0.0908517949276498 7.49895134507285E-4
49.7649764976498 0.0908354104774485 7.49489493523655E-4
49.7849784978498 0.0908190355605041 7.490842347424E-4
49.8049804980498 0.0908026701681514 7.48679357667619E-4
49.8249824982498 0.0907863142917366 7.48274861804247E-4
49.8449844984498 0.0907699679226177 7.47870746658058E-4
49.8649864986499 0.0907536310521642 7.47467011735657E-4
49.8849884988499 0.0907373036717571 7.47063656544473E-4
49.9049904990499 0.0907209857727886 7.4666068059277E-4
49.9249924992499 0.090704677346663 7.46258083389639E-4
49.9449944994499 0.0906883783847957 7.45855864444998E-4
49.96499649965 0.0906720888786133 7.4545402326958E-4
49.98499849985 0.0906558088195542 7.45052559374947E-4
50.00500050005 0.0906395381990678 7.44651472273473E-4
50.02500250025 0.0906232770086153 7.44250761478364E-4
50.04500450045 0.0906070252396689 7.43850426503628E-4
50.0650065006501 0.0905907828837123 7.43450466864094E-4

50.0850085008501 0.0905745499322402 7.43050882075402E-4
50.1050105010501 0.0905583263767589 7.42651671654004E-4
50.1250125012501 0.0905421122087858 7.42252835117159E-4
50.1450145014501 0.0905259074198495 7.41854371982937E-4
50.1650165016502 0.0905097120014897 7.41456281770205E-4
50.1850185018502 0.0904935259452573 7.41058563998644E-4
50.2050205020502 0.0904773492427147 7.40661218188734E-4
50.2250225022502 0.0904611818854348 7.40264243861747E-4
50.2450245024502 0.090445023865002 7.39867640539766E-4
50.2650265026503 0.0904288751730118 7.39471407745663E-4
50.2850285028503 0.0904127358010705 7.39075545003105E-4
50.3050305030503 0.0903966057407956 7.38680051836555E-4
50.3250325032503 0.0903804849838157 7.38284927771269E-4
50.3450345034504 0.0903643735217698 7.37890172333283E-4
50.3650365036504 0.090348271346309 7.37495785049438E-4
50.3850385038504 0.0903321784490941 7.37101765447345E-4
50.4050405040504 0.0903160948217975 7.36708113055408E-4
50.4250425042504 0.0903000204561024 7.36314827402812E-4
50.4450445044505 0.0902839553437026 7.35921908019521E-4
50.4650465046505 0.0902678994763031 7.35529354436284E-4
50.4850485048505 0.0902518528456196 7.35137166184624E-4
50.5050505050505 0.0902358154433785 7.34745342796841E-4
50.5250525052505 0.0902197872613172 7.34353883806012E-4
50.5450545054505 0.0902037682911834 7.33962788745979E-4
50.5650565056506 0.0901877585247361 7.33572057151363E-4
50.5850585058506 0.0901717579537445 7.33181688557549E-4
50.6050605060506 0.090155766569989 7.32791682500699E-4
50.6250625062506 0.0901397843652602 7.32402038517731E-4
50.6450645064506 0.0901238113313596 7.3201275614633E-4
50.6650665066507 0.0901078474600991 7.31623834924945E-4
50.6850685068507 0.0900918927433016 7.31235274392789E-4
50.7050705070507 0.0900759471728001 7.30847074089828E-4
50.7250725072507 0.0900600107404385 7.30459233556794E-4
50.7450745074507 0.0900440834380712 7.30071752335168E-4
50.7650765076508 0.0900281652575629 7.29684629967189E-4
50.7850785078508 0.0900122561907889 7.2929786599585E-4
50.8050805080508 0.0899963562296351 7.2891145996489E-4
50.8250825082508 0.0899804653659976 7.28525411418807E-4
50.8450845084508 0.0899645835917833 7.28139719902842E-4
50.8650865086509 0.089948710898909 7.27754384962979E-4
50.8850885088509 0.0899328472793022 7.27369406145951E-4
50.9050905090509 0.0899169927249007 7.26984782999234E-4
50.9250925092509 0.0899011472276527 7.26600515071047E-4
50.9450945094509 0.0898853107795167 7.26216601910347E-4

50.965096509651 0.0898694833724614 7.25833043066832E-4
50.985098509851 0.0898536649984658 7.25449838090932E-4
51.005100510051 0.0898378556495192 7.2506698653382E-4
51.025102510251 0.0898220553176213 7.24684487947398E-4
51.045104510451 0.0898062639947816 7.24302341884298E-4
51.0651065106511 0.08979048167302 7.23920547897887E-4
51.0851085108511 0.0897747083443668 7.23539105542262E-4
51.1051105110511 0.0897589440008622 7.23158014372245E-4
51.1251125112511 0.0897431886345565 7.22777273943383E-4
51.1451145114511 0.0897274422375103 7.22396883811953E-4
51.1651165116512 0.0897117048017942 7.22016843534949E-4
51.1851185118512 0.0896959763194886 7.21637152670087E-4
51.2051205120512 0.0896802567826844 7.21257810775806E-4
51.2251225122512 0.0896645461834822 7.20878817411262E-4
51.2451245124512 0.0896488445139928 7.20500172136326E-4
51.2651265126513 0.089633151766337 7.20121874511592E-4
51.2851285128513 0.0896174679326453 7.19743924098355E-4
51.3051305130513 0.0896017930050584 7.19366320458631E-4
51.3251325132513 0.089586126975727 7.1898906315515E-4
51.3451345134513 0.0895704698368112 7.18612151751339E-4
51.3651365136514 0.0895548215804815 7.18235585811343E-4
51.3851385138514 0.0895391821989182 7.17859364900013E-4
51.4051405140514 0.0895235516843112 7.17483488582896E-4
51.4251425142514 0.0895079300288604 7.17107956426254E-4
51.4451445144514 0.0894923172247756 7.16732767997045E-4
51.4651465146515 0.0894767132642761 7.16357922862924E-4
51.4851485148515 0.0894611181395913 7.15983420592252E-4
51.5051505150515 0.0894455318429599 7.15609260754079E-4
51.5251525152515 0.0894299543666308 7.1523544291816E-4
51.5451545154516 0.0894143857028624 7.14861966654939E-4
51.5651565156516 0.0893988258439227 7.14488831535551E-4
51.5851585158516 0.0893832747820894 7.14116037131829E-4
51.6051605160516 0.08936773250965 7.13743583016291E-4
51.6251625162516 0.0893521990189017 7.13371468762148E-4
51.6451645164517 0.0893366743021506 7.12999693943287E-4
51.6651665166517 0.0893211583517136 7.126282581343E-4
51.6851685168517 0.089305651159916 7.12257160910445E-4
51.7051705170517 0.0892901527190933 7.11886401847672E-4
51.7251725172517 0.0892746630215904 7.1151598052261E-4
51.7451745174518 0.0892591820597616 7.11145896512565E-4
51.7651765176518 0.089243709825971 7.10776149395533E-4
51.7851785178518 0.0892282463125917 7.10406738750172E-4
51.8051805180518 0.0892127915120066 7.10037664155826E-4
51.8251825182518 0.089197345416608 7.09668925192507E-4

51.8451845184518 0.0891819080187974 7.09300521440905E-4
51.8651865186519 0.0891664793109862 7.08932452482383E-4
51.8851885188519 0.0891510592855945 7.08564717898963E-4
51.9051905190519 0.0891356479350522 7.08197317273346E-4
51.9251925192519 0.0891202452517985 7.078302501889E-4
51.9451945194519 0.0891048512282819 7.07463516229653E-4
51.965196519652 0.0890894658569601 7.07097114980303E-4
51.985198519852 0.0890740891303 7.06731046026204E-4
52.005200520052 0.0890587210407783 7.06365308953382E-4
52.025202520252 0.0890433615808801 7.05999903348512E-4
52.045204520452 0.0890280107431006 7.05634828798939E-4
52.0652065206521 0.0890126685199438 7.05270084892661E-4
52.0852085208521 0.0889973349039226 7.04905671218326E-4
52.1052105210521 0.0889820098875597 7.04541587365246E-4
52.1252125212521 0.0889666934633866 7.04177832923384E-4
52.1452145214521 0.0889513856239438 7.03814407483351E-4
52.1652165216522 0.0889360863617811 7.03451310636412E-4
52.1852185218522 0.0889207956694577 7.03088541974485E-4
52.2052205220522 0.0889055135395414 7.02726101090129E-4
52.2252225222522 0.0888902399646091 7.02363987576552E-4
52.2452245224522 0.0888749749372472 7.02002201027612E-4
52.2652265226523 0.0888597184500508 7.01640741037807E-4
52.2852285228523 0.0888444704956238 7.01279607202276E-4
52.3052305230523 0.0888292310665796 7.00918799116804E-4
52.3252325232523 0.0888140001555402 7.00558316377813E-4
52.3452345234523 0.0887987777551369 7.00198158582367E-4
52.3652365236524 0.0887835638580093 6.99838325328161E-4
52.3852385238524 0.0887683584568068 6.99478816213534E-4
52.4052405240524 0.088753161544187 6.99119630837454E-4
52.4252425242524 0.0887379731128167 6.98760768799523E-4
52.4452445244524 0.0887227931553716 6.98402229699982E-4
52.4652465246525 0.0887076216645361 6.98044013139693E-4
52.4852485248525 0.0886924586330034 6.97686118720152E-4
52.5052505250525 0.088677304053476 6.97328546043491E-4
52.5252525252525 0.0886621579186645 6.96971294712455E-4
52.5452545254525 0.0886470202212888 6.96614364330423E-4
52.5652565256526 0.0886318909540772 6.96257754501396E-4
52.5852585258526 0.0886167701097671 6.95901464830001E-4
52.6052605260526 0.0886016576811043 6.95545494921483E-4
52.6252625262526 0.0885865536608439 6.95189844381715E-4
52.6452645264527 0.0885714580417487 6.94834512817177E-4
52.6652665266527 0.0885563708165912 6.94479499834977E-4
52.6852685268527 0.0885412919781521 6.94124805042839E-4
52.7052705270527 0.0885262215192206 6.93770428049099E-4

52.7252725272527 0.0885111594325949 6.93416368462709E-4
52.7452745274528 0.0884961057110814 6.9306262589323E-4
52.7652765276528 0.0884810603474956 6.92709199950845E-4
52.7852785278528 0.0884660233346613 6.92356090246341E-4
52.8052805280528 0.0884509946654106 6.92003296391108E-4
52.8252825282528 0.0884359743325845 6.91650817997152E-4
52.8452845284529 0.0884209623290328 6.91298654677093E-4
52.8652865286529 0.0884059586476131 6.9094680604414E-4
52.8852885288529 0.0883909632811919 6.90595271712114E-4
52.9052905290529 0.0883759762226444 6.90244051295444E-4
52.9252925292529 0.0883609974648539 6.89893144409158E-4
52.945294529453 0.0883460270007121 6.89542550668877E-4
52.965296529653 0.0883310648231195 6.8919226969083E-4
52.985298529853 0.0883161109249847 6.88842301091843E-4
53.005300530053 0.0883011652992249 6.88492644489336E-4
53.025302530253 0.0882862279387657 6.88143299501333E-4
53.045304530453 0.0882712988365406 6.87794265746434E-4
53.0653065306531 0.088256377985492 6.87445542843853E-4
53.0853085308531 0.0882414653785707 6.8709713041339E-4
53.1053105310531 0.0882265610087352 6.86749028075428E-4
53.1253125312531 0.088211664868953 6.8640123545095E-4
53.1453145314531 0.0881967769521994 6.86053752161523E-4
53.1653165316532 0.088181897251458 6.85706577829299E-4
53.1853185318532 0.088167025759721 6.85359712077021E-4
53.2053205320532 0.0881521624699885 6.85013154528013E-4
53.2253225322532 0.088137307375269 6.84666904806188E-4
53.2453245324532 0.0881224604685792 6.8432096253604E-4
53.2653265326533 0.0881076217429439 6.83975327342637E-4
53.2853285328533 0.0880927911913963 6.83629998851643E-4
53.3053305330533 0.0880779688069773 6.83284976689283E-4
53.3253325332533 0.0880631545827364 6.82940260482373E-4
53.3453345334533 0.088048348511731 6.82595849858301E-4
53.3653365336534 0.0880335505870268 6.82251744445033E-4
53.3853385338534 0.0880187608016974 6.81907943871105E-4
53.4053405340534 0.0880039791488245 6.8156444776563E-4
53.4253425342534 0.0879892056214979 6.81221255758294E-4
53.4453445344534 0.0879744402128157 6.80878367479355E-4
53.4653465346535 0.0879596829158836 6.80535782559634E-4
53.4853485348535 0.0879449337238158 6.80193500630531E-4
53.5053505350535 0.0879301926297338 6.79851521324001E-4
53.5253525352535 0.087915459626768 6.79509844272581E-4
53.5453545354535 0.087900734708056 6.79168469109358E-4
53.5653565356536 0.0878860178667438 6.78827395467994E-4
53.5853585358536 0.0878713090959853 6.78486622982712E-4

53.6053605360536 0.0878566083889421 6.78146151288294E-4
53.6253625362536 0.0878419157387838 6.77805980020083E-4
53.6453645364536 0.0878272311386881 6.77466108813983E-4
53.6653665366537 0.0878125545818406 6.77126537306462E-4
53.6853685368537 0.0877978860614343 6.76787265134538E-4
53.7053705370537 0.0877832255706707 6.76448291935789E-4
53.7253725372537 0.0877685731027585 6.76109617348344E-4
53.7453745374537 0.0877539286509147 6.75771241010891E-4
53.7653765376538 0.087739292208364 6.75433162562674E-4
53.7853785378538 0.0877246637683388 6.75095381643478E-4
53.8053805380538 0.0877100433240793 6.74757897893653E-4
53.8253825382538 0.0876954308688336 6.74420710954086E-4
53.8453845384539 0.0876808263958574 6.74083820466222E-4
53.8653865386539 0.0876662298984141 6.73747226072046E-4
53.8853885388539 0.0876516413697751 6.734109274141E-4
53.9053905390539 0.0876370608032191 6.73074924135459E-4
53.9253925392539 0.0876224881920329 6.72739215879752E-4
53.945394539454 0.087607923529511 6.72403802291152E-4
53.965396539654 0.0875933668089548 6.72068683014359E-4
53.985398539854 0.0875788180236742 6.71733857694632E-4
54.005400540054 0.0875642771669867 6.71399325977767E-4
54.025402540254 0.0875497442322169 6.71065087510091E-4
54.0454045404541 0.0875352192126974 6.70731141938477E-4
54.0654065406541 0.0875207021017681 6.70397488910326E-4
54.0854085408541 0.0875061928927769 6.70064128073587E-4
54.1054105410541 0.0874916915790789 6.69731059076734E-4
54.1254125412541 0.087477198154037 6.69398281568778E-4
54.1454145414542 0.0874627126110212 6.69065795199263E-4
54.1654165416542 0.0874482349434098 6.68733599618267E-4
54.1854185418542 0.0874337651445876 6.6840169447639E-4
54.2054205420542 0.0874193032079481 6.68070079424778E-4
54.2254225422542 0.0874048491268911 6.67738754115086E-4
54.2454245424542 0.0873904028948246 6.67407718199509E-4
54.2654265426543 0.0873759645051639 6.67076971330766E-4
54.2854285428543 0.0873615339513316 6.667465131621E-4
54.3054305430543 0.0873471112267578 6.6641634334728E-4
54.3254325432543 0.0873326963248802 6.66086461540598E-4
54.3454345434543 0.0873182892391434 6.65756867396864E-4
54.3654365436544 0.0873038899630001 6.6542756057142E-4
54.3854385438544 0.0872894984899097 6.65098540720115E-4
54.4054405440544 0.0872751148133395 6.64769807499329E-4
54.4254425442544 0.0872607389267637 6.64441360565953E-4
54.4454445444544 0.087246370823664 6.64113199577397E-4
54.4654465446545 0.0872320104975296 6.6378532419159E-4

54.4854485448545 0.0872176579418567 6.6345773406697E-4
54.5054505450545 0.087203313150149 6.63130428862497E-4
54.5254525452545 0.0871889761159175 6.62803408237642E-4
54.5454545454545 0.0871746468326803 6.62476671852383E-4
54.5654565456546 0.0871603252939628 6.62150219367216E-4
54.5854585458546 0.0871460114932975 6.61824050443139E-4
54.6054605460546 0.0871317054242246 6.61498164741672E-4
54.6254625462546 0.0871174070802912 6.61172561924834E-4
54.6454645464546 0.0871031164550514 6.60847241655152E-4
54.6654665466547 0.0870888335420666 6.60522203595656E-4
54.6854685468547 0.0870745583349059 6.60197447409898E-4
54.7054705470547 0.0870602908271445 6.59872972761909E-4
54.7254725472547 0.0870460310123659 6.59548779316246E-4
54.7454745474547 0.0870317788841598 6.59224866737953E-4
54.7654765476548 0.0870175344361238 6.58901234692588E-4
54.7854785478548 0.0870032976618619 6.58577882846197E-4
54.8054805480548 0.0869890685549854 6.58254810865332E-4
54.8254825482548 0.0869748471091133 6.5793201841705E-4
54.8454845484548 0.0869606333178708 6.57609505168892E-4
54.8654865486549 0.0869464271748906 6.57287270788906E-4
54.8854885488549 0.0869322286738122 6.56965314945627E-4
54.9054905490549 0.0869180378082825 6.56643637308093E-4
54.9254925492549 0.0869038545719551 6.56322237545832E-4
54.9454945494549 0.0868896789584907 6.56001115328866E-4
54.965496549655 0.086875510961557 6.55680270327704E-4
54.985498549855 0.0868613505748287 6.55359702213354E-4
55.005500550055 0.0868471977919874 6.5503941065731E-4
55.025502550255 0.0868330526067218 6.54719395331552E-4
55.0455045504551 0.0868189150127273 6.54399655908555E-4
55.0655065506551 0.0868047850037065 6.54080192061272E-4
55.0855085508551 0.0867906625733686 6.5376100346315E-4
55.1055105510551 0.08677654771543 6.53442089788119E-4
55.1255125512551 0.086762440423614 6.53123450710594E-4
55.1455145514552 0.0867483406916504 6.52805085905468E-4
55.1655165516552 0.0867342485132762 6.52486995048123E-4
55.1855185518552 0.0867201638822353 6.52169177814421E-4
55.2055205520552 0.0867060867922782 6.51851633880704E-4
55.2255225522552 0.0866920172371622 6.51534362923792E-4
55.2455245524553 0.0866779552106518 6.51217364620986E-4
55.2655265526553 0.0866639007065182 6.50900638650068E-4
55.2855285528553 0.0866498537185388 6.50584184689287E-4
55.3055305530553 0.0866358142404986 6.5026800241738E-4
55.3255325532553 0.0866217822661889 6.49952091513552E-4
55.3455345534554 0.0866077577894077 6.4963645165748E-4

55.3655365536554 0.0865937408039603 6.49321082529324E-4
55.3855385538554 0.0865797313036579 6.49005983809703E-4
55.4055405540554 0.0865657292823192 6.48691155179726E-4
55.4255425542554 0.0865517347337692 6.48376596320956E-4
55.4455445544554 0.0865377476518395 6.48062306915428E-4
55.4655465546555 0.0865237680303687 6.47748286645655E-4
55.4855485548555 0.0865097958632019 6.4743453519461E-4
55.5055505550555 0.0864958311441909 6.47121052245737E-4
55.5255525552555 0.0864818738671941 6.46807837482945E-4
55.5455545554555 0.0864679240260765 6.46494890590603E-4
55.5655565556556 0.08645398161471 6.46182211253557E-4
55.5855585558556 0.0864400466269728 6.45869799157105E-4
55.6055605560556 0.0864261190567497 6.4555765398701E-4
55.6255625562556 0.0864121988979324 6.45245775429502E-4
55.6455645564556 0.0863982861444191 6.44934163171271E-4
55.6655665566557 0.086384380790114 6.44622816899457E-4
55.6855685568557 0.0863704828289287 6.44311736301672E-4
55.7055705570557 0.0863565922547809 6.44000921065982E-4
55.7255725572557 0.0863427090615948 6.43690370880906E-4
55.7455745574557 0.0863288332433011 6.43380085435422E-4
55.7655765576558 0.0863149647938374 6.43070064418969E-4
55.7855785578558 0.0863011037071473 6.42760307521437E-4
55.8055805580558 0.0862872499771812 6.42450814433166E-4
55.8255825582558 0.0862734035978958 6.42141584844956E-4
55.8455845584558 0.0862595645632543 6.41832618448052E-4
55.8655865586559 0.0862457328672266 6.41523914934161E-4
55.8855885588559 0.0862319085037889 6.41215473995434E-4
55.9055905590559 0.0862180914669235 6.40907295324466E-4
55.9255925592559 0.0862042817506194 6.4059937861431E-4
55.9455945594559 0.0861904793488723 6.40291723558468E-4
55.965596559656 0.0861766842556836 6.39984329850878E-4
55.985598559856 0.0861628964650619 6.39677197185943E-4
56.005600560056 0.0861491159710214 6.39370325258488E-4
56.025602560256 0.0861353427675832 6.39063713763802E-4
56.045604560456 0.0861215768487745 6.38757362397608E-4
56.0656065606561 0.086107818208629 6.38451270856079E-4
56.0856085608561 0.0860940668411867 6.38145438835824E-4
56.1056105610561 0.0860803227404937 6.37839866033897E-4
56.1256125612561 0.0860665859006027 6.37534552147792E-4
56.1456145614561 0.0860528563155725 6.37229496875437E-4
56.1656165616562 0.0860391339794683 6.3692469991521E-4
56.1856185618562 0.0860254188863616 6.36620160965918E-4
56.2056205620562 0.08601171103033 6.36315879726811E-4
56.2256225622562 0.0859980104054574 6.36011855897571E-4

56.2456245624563 0.0859843170058343 6.3570808917832E-4
56.2656265626563 0.0859706308255569 6.35404579269613E-4
56.2856285628563 0.0859569518587279 6.35101325872437E-4
56.3056305630563 0.0859432800994561 6.34798328688212E-4
56.3256325632563 0.0859296155418566 6.34495587418795E-4
56.3456345634564 0.0859159581800509 6.34193101766476E-4
56.3656365636564 0.0859023080081662 6.33890871433965E-4
56.3856385638564 0.0858886650203362 6.33588896124415E-4
56.4056405640564 0.0858750292107005 6.33287175541396E-4
56.4256425642564 0.0858614005734054 6.32985709388922E-4
56.4456445644565 0.0858477791026026 6.3268449737142E-4
56.4656465646565 0.0858341647924505 6.32383539193748E-4
56.4856485648565 0.0858205576371132 6.32082834561194E-4
56.5056505650565 0.0858069576307612 6.31782383179468E-4
56.5256525652565 0.0857933647675712 6.31482184754709E-4
56.5456545654566 0.0857797790417255 6.31182238993469E-4
56.5656565656566 0.085766200447413 6.30882545602738E-4
56.5856585658566 0.0857526289788282 6.30583104289914E-4
56.6056605660566 0.0857390646301721 6.30283914762826E-4
56.6256625662566 0.0857255073956514 6.29984976729721E-4
56.6456645664566 0.0857119572694791 6.29686289899264E-4
56.6656665666567 0.0856984142458739 6.29387853980539E-4
56.6856685668567 0.0856848783190609 6.29089668683053E-4
56.7056705670567 0.0856713494832708 6.28791733716723E-4
56.7256725672567 0.0856578277327408 6.28494048791891E-4
56.7456745674567 0.0856443130617134 6.28196613619307E-4
56.7656765676568 0.0856308054644378 6.27899427910143E-4
56.7856785678568 0.0856173049351687 6.27602491375985E-4
56.8056805680568 0.0856038114681668 6.27305803728823E-4
56.8256825682568 0.0855903250576989 6.27009364681073E-4
56.8456845684568 0.0855768456980376 6.26713173945558E-4
56.8656865686569 0.0855633733834616 6.2641723123551E-4
56.8856885688569 0.0855499081082552 6.26121536264572E-4
56.9056905690569 0.085536449866709 6.25826088746805E-4
56.9256925692569 0.0855229986531191 6.25530888396666E-4
56.9456945694569 0.0855095544617879 6.25235934929033E-4
56.965696569657 0.0854961172870231 6.24941228059178E-4
56.985698569857 0.0854826871231389 6.24646767502797E-4
57.005700570057 0.0854692639644551 6.24352552975982E-4
57.025702570257 0.085455847805297 6.24058584195226E-4
57.045704570457 0.0854424386399963 6.23764860877439E-4
57.0657065706571 0.0854290364628902 6.23471382739922E-4
57.0857085708571 0.0854156412683218 6.23178149500392E-4
57.1057105710571 0.0854022530506399 6.22885160876959E-4

57.1257125712571 0.0853888718041993 6.22592416588138E-4
57.1457145714571 0.0853754975233605 6.22299916352848E-4
57.1657165716572 0.0853621302024897 6.22007659890405E-4
57.1857185718572 0.0853487698359587 6.21715646920518E-4
57.2057205720572 0.0853354164181457 6.21423877163313E-4
57.2257225722572 0.085322069943434 6.21132350339299E-4
57.2457245724572 0.0853087304062127 6.20841066169383E-4
57.2657265726573 0.0852953978008772 6.20550024374878E-4
57.2857285728573 0.0852820721218278 6.20259224677485E-4
57.3057305730573 0.0852687533634712 6.19968666799301E-4
57.3257325732573 0.0852554415202193 6.19678350462821E-4
57.3457345734573 0.0852421365864901 6.19388275390934E-4
57.3657365736574 0.0852288385567072 6.19098441306921E-4
57.3857385738574 0.0852155474252995 6.18808847934449E-4
57.4057405740574 0.085202263186702 6.1851949499759E-4
57.4257425742574 0.085188985835355 6.18230382220792E-4
57.4457445744575 0.085175715365705 6.17941509328909E-4
57.4657465746575 0.0851624517722036 6.17652876047173E-4
57.4857485748575 0.085149195049308 6.17364482101205E-4
57.5057505750575 0.0851359451914815 6.17076327217024E-4
57.5257525752575 0.0851227021931926 6.16788411121027E-4
57.5457545754576 0.0851094660489153 6.16500733539997E-4
57.5657565756576 0.0850962367531299 6.16213294201116E-4
57.5857585758576 0.0850830143003216 6.15926092831938E-4
57.6057605760576 0.0850697986849812 6.15639129160404E-4
57.6257625762576 0.0850565899016053 6.15352402914846E-4
57.6457645764577 0.0850433879446963 6.15065913823975E-4
57.6657665766577 0.0850301928087614 6.14779661616881E-4
57.6857685768577 0.085017004488314 6.14493646023041E-4
57.7057705770577 0.0850038229778727 6.14207866772313E-4
57.7257725772577 0.0849906482719619 6.13922323594935E-4
57.7457745774578 0.0849774803651111 6.13637016221521E-4
57.7657765776578 0.0849643192518556 6.1335194438307E-4
57.7857785778578 0.0849511649267362 6.13067107810959E-4
57.8057805780578 0.0849380173842989 6.12782506236936E-4
57.8257825782578 0.0849248766190956 6.12498139393139E-4
57.8457845784578 0.0849117426256834 6.12214007012071E-4
57.8657865786579 0.0848986153986249 6.11930108826615E-4
57.8857885788579 0.0848854949324879 6.11646444570026E-4
57.9057905790579 0.0848723812218461 6.11363013975942E-4
57.9257925792579 0.0848592742612785 6.1107981677837E-4
57.9457945794579 0.0848461740453692 6.10796852711684E-4
57.965796579658 0.0848330805687081 6.1051412151064E-4
57.985798579858 0.0848199938258903 6.10231622910365E-4

58.005800580058 0.0848069138115164 6.09949356646353E-4
58.025802580258 0.0847938405201923 6.09667322454467E-4
58.045804580458 0.0847807739465293 6.09385520070947E-4
58.0658065806581 0.084767714085144 6.09103949232395E-4
58.0858085808581 0.0847546609306585 6.08822609675787E-4
58.1058105810581 0.0847416144777003 6.08541501138465E-4
58.1258125812581 0.084728574720902 6.08260623358137E-4
58.1458145814581 0.0847155416549016 6.0797997607288E-4
58.1658165816582 0.0847025152743431 6.07699559021142E-4
58.1858185818582 0.0846894955738744 6.07419371941718E-4
58.2058205820582 0.0846764825481498 6.07139414573785E-4
58.2258225822582 0.0846634761918289 6.06859686656885E-4
58.2458245824582 0.0846504764995761 6.0658018793091E-4
58.2658265826583 0.0846374834660614 6.06300918136128E-4
58.2858285828583 0.0846244970859598 6.06021877013157E-4
58.3058305830583 0.084611517353952 6.0574306430299E-4
58.3258325832583 0.0845985442647237 6.05464479746971E-4
58.3458345834583 0.0845855778129657 6.05186123086804E-4
58.3658365836584 0.0845726179933744 6.04907994064563E-4
58.3858385838584 0.0845596648006511 6.04630092422666E-4
58.4058405840584 0.0845467182295026 6.04352417903902E-4
58.4258425842584 0.0845337782746406 6.0407497025141E-4
58.4458445844584 0.0845208449307825 6.03797749208691E-4
58.4658465846585 0.0845079181926504 6.03520754519599E-4
58.4858485848585 0.0844949980549719 6.03243985928346E-4
58.5058505850585 0.0844820845124796 6.02967443179498E-4
58.5258525852585 0.0844691775599115 6.02691126017975E-4
58.5458545854585 0.0844562771920104 6.02415034189051E-4
58.5658565856586 0.0844433834035248 6.02139167438359E-4
58.5858585858586 0.084430496189208 6.01863525511878E-4
58.6058605860586 0.0844176155438182 6.01588108155935E-4
58.6258625862586 0.0844047414621195 6.01312915117224E-4
58.6458645864587 0.0843918739388805 6.01037946142777E-4
58.6658665866587 0.084379012968875 6.00763200979978E-4
58.6858685868587 0.084366158546882 6.00488679376563E-4
58.7058705870587 0.0843533106676856 6.00214381080616E-4
58.7258725872587 0.0843404693260752 5.99940305840571E-4
58.7458745874588 0.0843276345168451 5.9966645340521E-4
58.7658765876588 0.0843148062347945 5.99392823523659E-4
58.7858785878588 0.0843019844747281 5.99119415945394E-4
58.8058805880588 0.0842891692314551 5.98846230420233E-4
58.8258825882588 0.0842763604997905 5.98573266698346E-4
58.8458845884589 0.0842635582745536 5.98300524530238E-4
58.8658865886589 0.0842507625505694 5.98028003666773E-4

58.8858885888589 0.0842379733226673 5.9775570385914E-4
58.9058905890589 0.0842251905856823 5.97483624858887E-4
58.9258925892589 0.0842124143344542 5.97211766417898E-4
58.945894589459 0.0841996445638275 5.96940128288394E-4
58.965896589659 0.0841868812686522 5.96668710222946E-4
58.985898589859 0.0841741244437833 5.96397511974464E-4
59.005900590059 0.0841613740840802 5.96126533296191E-4
59.025902590259 0.0841486301844079 5.95855773941718E-4
59.045904590459 0.0841358927396361 5.9558523366497E-4
59.0659065906591 0.0841231617446393 5.95314912220208E-4
59.0859085908591 0.0841104371942976 5.95044809362043E-4
59.1059105910591 0.0840977190834953 5.94774924845408E-4
59.1259125912591 0.084085007407122 5.94505258425579E-4
59.1459145914591 0.0840723021600723 5.94235809858171E-4
59.1659165916592 0.0840596033372457 5.93966578899129E-4
59.1859185918592 0.0840469109335463 5.93697565304735E-4
59.2059205920592 0.0840342249438837 5.93428768831609E-4
59.2259225922592 0.0840215453631719 5.93160189236697E-4
59.2459245924592 0.0840088721863301 5.92891826277288E-4
59.2659265926593 0.083996205408282 5.92623679710988E-4
59.2859285928593 0.0839835450239567 5.92355749295753E-4
59.3059305930593 0.0839708910282879 5.92088034789857E-4
59.3259325932593 0.0839582434162143 5.91820535951914E-4
59.3459345934593 0.0839456021826791 5.91553252540859E-4
59.3659365936594 0.0839329673226311 5.91286184315969E-4
59.3859385938594 0.0839203388310229 5.91019331036836E-4
59.4059405940594 0.0839077167028132 5.90752692463394E-4
59.4259425942594 0.0838951009329642 5.90486268355891E-4
59.4459445944594 0.083882491516444 5.90220058474915E-4
59.4659465946595 0.0838698884482249 5.89954062581372E-4
59.4859485948595 0.0838572917232843 5.896882804365E-4
59.5059505950595 0.0838447013366045 5.89422711801864E-4
59.5259525952595 0.0838321172831722 5.89157356439345E-4
59.5459545954595 0.0838195395579791 5.88892214111158E-4
59.5659565956596 0.083806968156022 5.88627284579841E-4
59.5859585958596 0.0837944030723018 5.88362567608246E-4
59.6059605960596 0.0837818443018247 5.88098062959559E-4
59.6259625962596 0.0837692918396018 5.87833770397289E-4
59.6459645964596 0.0837567456806481 5.87569689685251E-4
59.6659665966597 0.0837442058199844 5.87305820587605E-4
59.6859685968597 0.0837316722526356 5.87042162868812E-4
59.7059705970597 0.0837191449736315 5.86778716293666E-4
59.7259725972597 0.0837066239780066 5.8651548062727E-4
59.7459745974598 0.0836941092608002 5.86252455635056E-4

59.7659765976598 0.0836816008170563 5.85989641082767E-4
59.7859785978598 0.0836690986418236 5.85727036736472E-4
59.8059805980598 0.0836566027301552 5.85464642362544E-4
59.8259825982598 0.0836441130771095 5.85202457727692E-4
59.8459845984599 0.0836316296777491 5.84940482598926E-4
59.8659865986599 0.0836191525271414 5.84678716743576E-4
59.8859885988599 0.0836066816203586 5.84417159929291E-4
59.9059905990599 0.0835942169524776 5.84155811924034E-4
59.9259925992599 0.0835817585185794 5.83894672496075E-4
59.94599459946 0.0835693063137506 5.8363374141401E-4
59.96599659966 0.0835568603330815 5.83373018446736E-4
59.98599859986 0.083544420571668 5.83112503363477E-4
60.00600060006 0.0835319870246095 5.82852195933749E-4
60.02600260026 0.083519559687011 5.82592095927401E-4
60.0460046004601 0.0835071385539817 5.82332203114579E-4
60.0660066006601 0.0834947236206353 5.82072517265741E-4
60.0860086008601 0.0834823148820906 5.81813038151669E-4
60.1060106010601 0.0834699123334704 5.81553765543433E-4
60.1260126012601 0.0834575159699024 5.81294699212427E-4
60.1460146014602 0.0834451257865191 5.81035838930352E-4
60.1660166016602 0.0834327417784569 5.80777184469211E-4
60.1860186018602 0.0834203639408574 5.80518735601316E-4
60.2060206020602 0.0834079922688666 5.8026049209929E-4
60.2260226022602 0.0833956267576353 5.80002453736066E-4
60.2460246024602 0.0833832674023181 5.7974462028487E-4
60.2660266026603 0.0833709141980749 5.79486991519245E-4
60.2860286028603 0.0833585671400698 5.79229567213032E-4
60.3060306030603 0.0833462262234715 5.78972347140379E-4
60.3260326032603 0.0833338914434532 5.78715331075739E-4
60.3460346034603 0.0833215627951927 5.78458518793868E-4
60.3660366036604 0.0833092402738724 5.78201910069828E-4
60.3860386038604 0.0832969238746788 5.7794550467897E-4
60.4060406040604 0.0832846135928034 5.77689302396967E-4
60.4260426042604 0.0832723094234419 5.77433302999779E-4
60.4460446044604 0.0832600113617945 5.77177506263668E-4
60.4660466046605 0.0832477194030659 5.76921911965201E-4
60.4860486048605 0.0832354335424654 5.76666519881245E-4
60.5060506050605 0.0832231537752069 5.76411329788965E-4
60.5260526052605 0.0832108800965082 5.76156341465822E-4
60.5460546054605 0.0831986125015921 5.75901554689578E-4
60.5660566056606 0.0831863509856856 5.75646969238294E-4
60.5860586058606 0.0831740955440204 5.7539258489033E-4
60.6060606060606 0.083161846171832 5.75138401424332E-4
60.6260626062606 0.0831496028643612 5.74884418619256E-4

60.6460646064606 0.0831373656168526 5.74630636254348E-4
60.6660666066607 0.0831251344245555 5.74377054109149E-4
60.6860686068607 0.0831129092827236 5.74123671963497E-4
60.7060706070607 0.0831006901866146 5.7387048959752E-4
60.7260726072607 0.0830884771314914 5.73617506791646E-4
60.7460746074607 0.0830762701126204 5.7336472332659E-4
60.7660766076608 0.0830640691252734 5.7311213898337E-4
60.7860786078608 0.0830518741647255 5.72859753543284E-4
60.8060806080608 0.0830396852262568 5.72607566787929E-4
60.8260826082608 0.0830275023051519 5.72355578499196E-4
60.8460846084608 0.0830153253966992 5.72103788459259E-4
60.8660866086609 0.0830031544961919 5.71852196450591E-4
60.8860886088609 0.0829909895989275 5.71600802255947E-4
60.9060906090609 0.0829788307002076 5.71349605658378E-4
60.9260926092609 0.0829666777953388 5.71098606441229E-4
60.946094609461 0.082954530879631 5.70847804388115E-4
60.966096609661 0.0829423899483992 5.70597199282956E-4
60.986098609861 0.0829302549969626 5.70346790909957E-4
61.006100610061 0.0829181260206445 5.70096579053605E-4
61.026102610261 0.0829060030147725 5.69846563498674E-4
61.0461046104611 0.0828938859746788 5.69596744030229E-4
61.0661066106611 0.0828817748956998 5.69347120433621E-4
61.0861086108611 0.0828696697731762 5.69097692494486E-4
61.1061106110611 0.0828575706024527 5.68848459998736E-4
61.1261126112611 0.0828454773788786 5.68599422732579E-4
61.1461146114612 0.0828333900978072 5.68350580482499E-4
61.1661166116612 0.0828213087545965 5.68101933035269E-4
61.1861186118612 0.0828092333446085 5.67853480177944E-4
61.2061206120612 0.0827971638632093 5.67605221697859E-4
61.2261226122612 0.0827851003057695 5.67357157382632E-4
61.2461246124613 0.0827730426676638 5.67109287020163E-4
61.2661266126613 0.0827609909442712 5.66861610398633E-4
61.2861286128613 0.082748945130975 5.66614127306505E-4
61.3061306130613 0.0827369052231626 5.66366837532519E-4
61.3261326132613 0.0827248712162259 5.66119740865702E-4
61.3461346134614 0.0827128431055605 5.65872837095349E-4
61.3661366136614 0.0827008208865669 5.65626126011047E-4
61.3861386138614 0.082688804554649 5.6537960740265E-4
61.4061406140614 0.0826767941052156 5.65133281060296E-4
61.4261426142614 0.0826647895336793 5.64887146774397E-4
61.4461446144614 0.082652790835457 5.64641204335647E-4
61.4661466146615 0.0826407980059699 5.64395453535013E-4
61.4861486148615 0.0826288110406432 5.64149894163739E-4
61.5061506150615 0.0826168299349063 5.63904526013346E-4

61.5261526152615 0.0826048546841929 5.63659348875627E-4
61.5461546154615 0.0825928852839406 5.6341436254265E-4
61.5661566156616 0.0825809217295917 5.63169566806766E-4
61.5861586158616 0.0825689640165919 5.62924961460588E-4
61.6061606160616 0.0825570121403916 5.6268054629701E-4
61.6261626162616 0.082545066096445 5.62436321109193E-4
61.6461646164616 0.0825331258802108 5.6219228569058E-4
61.6661666166617 0.0825211914871517 5.6194843983488E-4
61.6861686168617 0.0825092629127339 5.61704783336064E-4
61.7061706170617 0.0824973401524289 5.61461315988398E-4
61.7261726172617 0.0824854232017113 5.61218037586401E-4
61.7461746174617 0.0824735120560604 5.60974947924865E-4
61.7661766176618 0.082461606710959 5.60732046798851E-4
61.7861786178618 0.0824497071618949 5.604893340037E-4
61.8061806180618 0.0824378134043591 5.60246809335008E-4
61.8261826182618 0.0824259254338471 5.6000447258865E-4
61.8461846184618 0.0824140432458586 5.59762323560766E-4
61.8661866186619 0.082402166835897 5.59520362047759E-4
61.8861886188619 0.08239029619947 5.59278587846305E-4
61.9061906190619 0.0823784313320894 5.59037000753346E-4
61.9261926192619 0.082366572229271 5.58795600566092E-4
61.9461946194619 0.0823547188865346 5.58554387082016E-4
61.966196619662 0.0823428712994041 5.58313360098856E-4
61.986198619862 0.0823310294634074 5.58072519414619E-4
62.006200620062 0.0823191933740764 5.57831864827572E-4
62.026202620262 0.0823073630269474 5.57591396136254E-4
62.046204620462 0.08229553841756 5.57351113139458E-4
62.0662066206621 0.0822837195414586 5.57111015636251E-4
62.0862086208621 0.0822719063941911 5.56871103425955E-4
62.1062106210621 0.0822600989713096 5.56631376308155E-4
62.1262126212621 0.0822482972683702 5.56391834082707E-4
62.1462146214622 0.0822365012809328 5.56152476549716E-4
62.1662166216622 0.0822247110045618 5.55913303509562E-4
62.1862186218622 0.0822129264348249 5.55674314762872E-4
62.2062206220622 0.0822011475672944 5.55435510110544E-4
62.2262226222622 0.0821893743975464 5.55196889353737E-4
62.2462246224623 0.0821776069211605 5.54958452293859E-4
62.2662266226623 0.0821658451337211 5.5472019873259E-4
62.2862286228623 0.0821540890308158 5.54482128471854E-4
62.3062306230623 0.0821423386080368 5.54244241313854E-4
62.3262326232623 0.0821305938609795 5.54006537061031E-4
62.3462346234624 0.082118854785244 5.53769015516094E-4
62.3662366236624 0.0821071213764342 5.53531676482012E-4
62.3862386238624 0.0820953936301576 5.53294519762005E-4

62.4062406240624 0.0820836715420256 5.53057545159548E-4
62.4262426242624 0.0820719551076539 5.52820752478375E-4
62.4462446244625 0.0820602443226621 5.52584141522479E-4
62.4662466246625 0.0820485391826735 5.52347712096105E-4
62.4862486248625 0.0820368396833153 5.52111464003752E-4
62.5062506250625 0.0820251458202187 5.51875397050172E-4
62.5262526252625 0.0820134575890189 5.51639511040376E-4
62.5462546254626 0.0820017749853549 5.51403805779625E-4
62.5662566256626 0.0819900980048694 5.51168281073433E-4
62.5862586258626 0.0819784266432095 5.50932936727574E-4
62.6062606260626 0.0819667608960257 5.50697772548062E-4
62.6262626262626 0.0819551007589726 5.50462788341175E-4
62.6462646264627 0.0819434462277085 5.50227983913431E-4
62.6662666266627 0.0819317972978957 5.4999335907161E-4
62.6862686268627 0.0819201539652004 5.49758913622735E-4
62.7062706270627 0.0819085162252929 5.4952464737409E-4
62.7262726272627 0.0818968840738467 5.49290560133195E-4
62.7462746274627 0.0818852575065395 5.49056651707826E-4
62.7662766276628 0.0818736365190531 5.48822921906013E-4
62.7862786278628 0.0818620211070729 5.48589370536033E-4
62.8062806280628 0.0818504112662879 5.483559974064E-4
62.8262826282628 0.0818388069923914 5.48122802325895E-4
62.8462846284628 0.0818272082810802 5.4788978510353E-4
62.8662866286629 0.0818156151280551 5.47656945548578E-4
62.8862886288629 0.0818040275290204 5.47424283470544E-4
62.9062906290629 0.0817924454796848 5.47191798679195E-4
62.9262926292629 0.0817808689757602 5.46959490984536E-4
62.9462946294629 0.0817692980129626 5.46727360196813E-4
62.966296629663 0.0817577325870119 5.46495406126533E-4
62.986298629863 0.0817461726936314 5.46263628584428E-4
63.006300630063 0.0817346183285485 5.46032027381491E-4
63.026302630263 0.0817230694874945 5.45800602328952E-4
63.046304630463 0.0817115261662041 5.45569353238284E-4
63.0663066306631 0.0816999883604162 5.45338279921208E-4
63.0863086308631 0.0816884560658729 5.45107382189683E-4
63.1063106310631 0.0816769292783207 5.44876659855915E-4
63.1263126312631 0.0816654079935095 5.44646112732352E-4
63.1463146314631 0.0816538922071928 5.44415740631675E-4
63.1663166316632 0.0816423819151283 5.44185543366822E-4
63.1863186318632 0.0816308771130773 5.43955520750962E-4
63.2063206320632 0.0816193777968045 5.43725672597505E-4
63.2263226322632 0.0816078839620788 5.43495998720107E-4
63.2463246324632 0.0815963956046725 5.43266498932658E-4
63.2663266326633 0.0815849127203618 5.43037173049289E-4

63.2863286328633 0.0815734353049267 5.42808020884376E-4
63.3063306330633 0.0815619633541506 5.42579042252526E-4
63.3263326332633 0.0815504968638209 5.42350236968589E-4
63.3463346334634 0.0815390358297286 5.4212160484765E-4
63.3663366336634 0.0815275802476684 5.41893145705036E-4
63.3863386338634 0.0815161301134391 5.41664859356315E-4
63.4063406340634 0.0815046854228423 5.41436745617279E-4
63.4263426342634 0.0814932461716841 5.41208804303969E-4
63.4463446344635 0.0814818123557739 5.40981035232655E-4
63.4663466346635 0.081470383970925 5.40753438219847E-4
63.4863486348635 0.0814589610129542 5.4052601308229E-4
63.5063506350635 0.0814475434776821 5.40298759636963E-4
63.5263526352635 0.0814361313609329 5.40071677701083E-4
63.5463546354636 0.0814247246585345 5.39844767092098E-4
63.5663566356636 0.0814133233663183 5.39618027627689E-4
63.5863586358636 0.0814019274801197 5.39391459125775E-4
63.6063606360636 0.0813905369957774 5.39165061404507E-4
63.6263626362636 0.081379151909134 5.38938834282269E-4
63.6463646364637 0.0813677722160356 5.38712777577677E-4
63.6663666366637 0.0813563979123319 5.38486891109578E-4
63.6863686368637 0.0813450289938766 5.3826117469706E-4
63.7063706370637 0.0813336654565265 5.38035628159427E-4
63.7263726372637 0.0813223072961423 5.37810251316227E-4
63.7463746374638 0.0813109545085884 5.37585043987236E-4
63.7663766376638 0.0812996070897325 5.37360005992456E-4
63.7863786378638 0.0812882650354465 5.37135137152127E-4
63.8063806380638 0.0812769283416052 5.36910437286709E-4
63.8263826382638 0.0812655970040876 5.36685906216904E-4
63.8463846384639 0.0812542710187758 5.36461543763629E-4
63.8663866386639 0.081242950381556 5.36237349748046E-4
63.8863886388639 0.0812316350883176 5.36013323991529E-4
63.9063906390639 0.0812203251349536 5.35789466315688E-4
63.9263926392639 0.0812090205173608 5.35565776542364E-4
63.9463946394639 0.0811977212314397 5.35342254493622E-4
63.966396639664 0.0811864272730939 5.35118899991752E-4
63.986398639864 0.0811751386382311 5.34895712859278E-4
64.006400640064 0.081163855322762 5.34672692918937E-4
64.026402640264 0.0811525773226013 5.34449839993703E-4
64.046404640464 0.0811413046336672 5.34227153906779E-4
64.0664066406641 0.0811300372518813 5.3400463448158E-4
64.0864086408641 0.0811187751731687 5.33782281541752E-4
64.1064106410641 0.0811075183934584 5.33560094911175E-4
64.1264126412641 0.0810962669086827 5.3333807441394E-4
64.1464146414641 0.0810850207147774 5.33116219874369E-4

64.1664166416642 0.0810737798076818 5.32894531117004E-4
64.1864186418642 0.0810625441833389 5.32673007966612E-4
64.2064206420642 0.0810513138376954 5.32451650248189E-4
64.2264226422642 0.081040088766701 5.32230457786942E-4
64.2464246424642 0.0810288689663092 5.32009430408307E-4
64.2664266426643 0.0810176544324769 5.31788567937938E-4
64.2864286428643 0.0810064451611647 5.31567870201717E-4
64.3064306430643 0.0809952411483369 5.31347337025747E-4
64.3264326432643 0.0809840423899608 5.31126968236346E-4
64.3464346434643 0.0809728488820074 5.30906763660056E-4
64.3664366436644 0.0809616606204512 5.30686723123635E-4
64.3864386438644 0.0809504776012702 5.30466846454068E-4
64.4064406440644 0.080939299820446 5.30247133478554E-4
64.4264426442644 0.0809281272739635 5.30027584024517E-4
64.4464446444644 0.0809169599578114 5.29808197919598E-4
64.4664466446645 0.0809057978679809 5.29588974991642E-4
64.4864486448645 0.0808946410004683 5.29369915068742E-4
64.5064506450645 0.0808834893512719 5.29151017979183E-4
64.5264526452645 0.0808723429163943 5.28932283551478E-4
64.5464546454646 0.080861201691841 5.28713711614355E-4
64.5664566456646 0.0808500656736214 5.28495301996762E-4
64.5864586458646 0.0808389348577482 5.28277054527861E-4
64.6064606460646 0.0808278092402376 5.28058969037032E-4
64.6264626462646 0.0808166888171091 5.27841045353869E-4
64.6464646464647 0.0808055735843858 5.27623283308185E-4
64.6664666466647 0.080794463538094 5.27405682730001E-4
64.6864686468647 0.0807833586742638 5.27188243449562E-4
64.7064706470647 0.0807722589889281 5.26970965297318E-4
64.7264726472647 0.0807611644781242 5.26753848103947E-4
64.7464746474648 0.0807500751378917 5.26536891700325E-4
64.7664766476648 0.0807389909642745 5.26320095917553E-4
64.7864786478648 0.0807279119533196 5.26103460586944E-4
64.8064806480648 0.0807168381010771 5.25886985540017E-4
64.8264826482648 0.0807057694036015 5.25670670608519E-4
64.8464846484649 0.0806947058569491 5.25454515624386E-4
64.8664866486649 0.0806836474571811 5.25238520419789E-4
64.8864886488649 0.0806725942003611 5.25022684827094E-4
64.9064906490649 0.0806615460825571 5.24807008678896E-4
64.9264926492649 0.080650503099839 5.24591491807978E-4
64.946494649465 0.0806394652482815 5.24376134047354E-4
64.966496649665 0.0806284325239622 5.24160935230242E-4
64.986498649865 0.0806174049229614 5.2394589519006E-4
65.006500650065 0.0806063824413641 5.23731013760458E-4
65.026502650265 0.0805953650752576 5.23516290775277E-4

65.0465046504651 0.0805843528207328 5.2330172606857E-4
65.0665066506651 0.0805733456738841 5.23087319474605E-4
65.0865086508651 0.0805623436308096 5.22873070827859E-4
65.1065106510651 0.0805513466876098 5.22658979963007E-4
65.1265126512651 0.0805403548403894 5.22445046714943E-4
65.1465146514652 0.0805293680852562 5.22231270918764E-4
65.1665166516652 0.0805183864183213 5.22017652409776E-4
65.1865186518652 0.0805074098356989 5.21804191023488E-4
65.2065206520652 0.0804964383335069 5.21590886595621E-4
65.2265226522652 0.080485471907867 5.21377738962107E-4
65.2465246524653 0.0804745105549025 5.21164747959062E-4
65.2665266526653 0.0804635542707421 5.20951913422838E-4
65.2865286528653 0.0804526030515163 5.2073923518997E-4
65.3065306530653 0.0804416568933599 5.20526713097211E-4
65.3265326532653 0.0804307157924105 5.20314346981513E-4
65.3465346534654 0.080419779744809 5.20102136680032E-4
65.3665366536654 0.0804088487466998 5.1989008203013E-4
65.3865386538654 0.0803979227942303 5.19678182869373E-4
65.4065406540654 0.0803870018835518 5.19466439035534E-4
65.4265426542654 0.0803760860108184 5.19254850366585E-4
65.4465446544655 0.0803651751721872 5.19043416700696E-4
65.4665466546655 0.0803542693638196 5.18832137876259E-4
65.4865486548655 0.0803433685818795 5.18621013731848E-4
65.5065506550655 0.0803324728225339 5.18410044106244E-4
65.5265526552655 0.0803215820819538 5.1819922883844E-4
65.5465546554656 0.0803106963563132 5.17988567767622E-4
65.5665566556656 0.080299815641789 5.17778060733177E-4
65.5865586558656 0.0802889399345615 5.17567707574692E-4
65.6065606560656 0.080278069230815 5.17357508131965E-4
65.6265626562656 0.080267203526736 5.17147462244982E-4
65.6465646564657 0.0802563428185147 5.16937569753935E-4
65.6665666566657 0.080245487102345 5.16727830499218E-4
65.6865686568657 0.0802346363744233 5.1651824432142E-4
65.7065706570657 0.0802237906309495 5.16308811061329E-4
65.7265726572657 0.0802129498681272 5.16099530559938E-4
65.7465746574657 0.0802021140821623 5.15890402658427E-4
65.7665766576658 0.0801912832692652 5.15681427198194E-4
65.7865786578658 0.080180457425648 5.15472604020809E-4
65.8065806580658 0.0801696365475276 5.15263932968068E-4
65.8265826582658 0.0801588206311229 5.15055413881938E-4
65.8465846584658 0.0801480096726569 5.14847046604607E-4
65.8665866586659 0.080137203668355 5.1463883097844E-4
65.8865886588659 0.0801264026144464 5.14430766846009E-4
65.9065906590659 0.0801156065071635 5.14222854050084E-4

65.9265926592659 0.0801048153427416 5.14015092433625E-4
65.9465946594659 0.0800940291174194 5.1380748183979E-4
65.966596659666 0.0800832478274387 5.13600022111932E-4
65.986598659866 0.0800724714690449 5.13392713093608E-4
66.006600660066 0.0800617000384855 5.13185554628547E-4
66.026602660266 0.080050933532013 5.12978546560707E-4
66.046604660466 0.080040171945881 5.127716887342E-4
66.0666066606661 0.0800294152763481 5.12564980993372E-4
66.0866086608661 0.0800186635196749 5.12358423182731E-4
66.1066106610661 0.0800079166721258 5.12152015146999E-4
66.1266126612661 0.0799971747299681 5.11945756731082E-4
66.1466146614661 0.0799864376894722 5.11739647780076E-4
66.1666166616662 0.0799757055469121 5.1153368813928E-4
66.1866186618662 0.079964978298564 5.11327877654172E-4
66.2066206620662 0.0799542559407087 5.11122216170438E-4
66.2266226622662 0.0799435384696293 5.10916703533947E-4
66.2466246624662 0.0799328258816118 5.10711339590755E-4
66.2666266626663 0.0799221181729459 5.10506124187119E-4
66.2866286628663 0.0799114153399242 5.10301057169479E-4
66.3066306630663 0.0799007173788425 5.10096138384471E-4
66.3266326632663 0.0798900242859996 5.09891367678915E-4
66.3466346634663 0.0798793360576978 5.0968674489983E-4
66.3666366636664 0.0798686526902424 5.09482269894422E-4
66.3866386638664 0.0798579741799415 5.09277942510083E-4
66.4066406640664 0.0798473005231068 5.09073762594395E-4
66.4266426642664 0.0798366317160527 5.08869729995132E-4
66.4466446644664 0.079825967755097 5.08665844560253E-4
66.4666466646665 0.0798153086365606 5.08462106137909E-4
66.4866486648665 0.0798046543567676 5.08258514576443E-4
66.5066506650665 0.0797940049120447 5.0805506972437E-4
66.5266526652665 0.0797833602987224 5.07851771430412E-4
66.5466546654665 0.0797727205131341 5.07648619543469E-4
66.5666566656666 0.0797620855516161 5.0744561391263E-4
66.5866586658666 0.0797514554105077 5.07242754387164E-4
66.6066606660666 0.0797408300861519 5.07040040816539E-4
66.6266626662666 0.079730209574894 5.06837473050398E-4
66.6466646664666 0.0797195938730833 5.06635050938582E-4
66.6666666666667 0.0797089829770711 5.06432774331102E-4
66.6866686668667 0.0796983768832129 5.0623064307817E-4
66.7066706670667 0.0796877755878665 5.06028657030174E-4
66.7266726672667 0.0796771790873931 5.0582681603769E-4
66.7466746674667 0.079666587378157 5.05625119951479E-4
66.7666766676668 0.0796560004565252 5.0542356862248E-4
66.7866786678668 0.0796454183188684 5.05222161901831E-4

66.8066806680668 0.0796348409615601 5.05020899640842E-4
66.8266826682668 0.0796242683809765 5.04819781691008E-4
66.8466846684669 0.0796137005734975 5.04618807904012E-4
66.8666866686669 0.0796031375355055 5.04417978131714E-4
66.8866886688669 0.0795925792633858 5.04217292226154E-4
66.9066906690669 0.0795820257535279 5.04016750039574E-4
66.9266926692669 0.0795714770023233 5.03816351424378E-4
66.946694669467 0.0795609330061665 5.03616096233157E-4
66.966696669667 0.0795503937614557 5.03415984318688E-4
66.986698669867 0.0795398592645919 5.03216015533929E-4
67.006700670067 0.0795293295119789 5.03016189732018E-4
67.026702670267 0.0795188045000236 5.02816506766272E-4
67.0467046704671 0.0795082842251361 5.02616966490189E-4
67.0667066706671 0.0794977686837296 5.02417568757452E-4
67.0867086708671 0.07948725787222 5.02218313421921E-4
67.1067106710671 0.0794767517870265 5.02019200337636E-4
67.1267126712671 0.079466250424571 5.01820229358815E-4
67.1467146714672 0.0794557537812792 5.01621400339866E-4
67.1667166716672 0.0794452618535787 5.01422713135357E-4
67.1867186718672 0.0794347746379008 5.01224167600051E-4
67.2067206720672 0.0794242921306797 5.01025763588884E-4
67.2267226722672 0.0794138143283528 5.00827500956975E-4
67.2467246724673 0.07940334122736 5.00629379559612E-4
67.2667266726673 0.0793928728241446 5.00431399252269E-4
67.2867286728673 0.0793824091151528 5.00233559890595E-4
67.3067306730673 0.0793719500968336 5.00035861330414E-4
67.3267326732673 0.0793614957656397 4.99838303427737E-4
67.3467346734674 0.0793510461180254 4.99640886038733E-4
67.3667366736674 0.0793406011504496 4.99443609019771E-4
67.3867386738674 0.0793301608593732 4.99246472227379E-4
67.4067406740674 0.0793197252412603 4.99049475518269E-4
67.4267426742674 0.0793092942925776 4.98852618749321E-4
67.4467446744675 0.079298868009796 4.98655901777611E-4
67.4667466746675 0.0792884463893879 4.98459324460363E-4
67.4867486748675 0.0792780294278292 4.98262886654991E-4
67.5067506750675 0.0792676171215993 4.98066588219088E-4
67.5267526752675 0.07925720946718 4.97870429010414E-4
67.5467546754676 0.0792468064610561 4.97674408886906E-4
67.5667566756676 0.0792364080997153 4.97478527706671E-4
67.5867586758676 0.0792260143796489 4.97282785328E-4
67.6067606760676 0.07921562529735 4.97087181609344E-4
67.6267626762676 0.079205240849316 4.96891716409346E-4
67.6467646764677 0.0791948610320458 4.96696389586796E-4
67.6667666766677 0.0791844858420428 4.96501201000689E-4

67.6867686768677 0.0791741152758117 4.96306150510161E-4
67.7067706770677 0.0791637493298615 4.96111237974544E-4
67.7267726772677 0.0791533880007037 4.95916463253335E-4
67.7467746774678 0.0791430312848523 4.95721826206197E-4
67.7667766776678 0.0791326791788246 4.95527326692971E-4
67.7867786778678 0.079122331679141 4.9533296457367E-4
67.8067806780678 0.0791119887823243 4.9513873970847E-4
67.8267826782678 0.0791016504849011 4.94944651957736E-4
67.8467846784679 0.0790913167834 4.94750701181986E-4
67.8667866786679 0.0790809876743526 4.94556887241908E-4
67.8867886788679 0.0790706631542942 4.94363209998378E-4
67.9067906790679 0.0790603432197624 4.94169669312427E-4
67.9267926792679 0.0790500278672974 4.93976265045258E-4
67.946794679468 0.0790397170934431 4.93782997058246E-4
67.966796679668 0.0790294108947459 4.93589865212941E-4
67.986798679868 0.0790191092677551 4.93396869371053E-4
68.006800680068 0.0790088122090228 4.9320400939446E-4
68.026802680268 0.0789985197151041 4.93011285145216E-4
68.0468046804681 0.078988231782557 4.92818696485539E-4
68.0668066806681 0.0789779484079427 4.92626243277821E-4
68.0868086808681 0.0789676695878247 4.92433925384615E-4
68.1068106810681 0.0789573953187695 4.92241742668638E-4
68.1268126812681 0.0789471255973471 4.9204969499279E-4
68.1468146814681 0.0789368604201293 4.91857782220119E-4
68.1668166816682 0.0789265997836921 4.91666004213863E-4
68.1868186818682 0.0789163436846132 4.91474360837402E-4
68.2068206820682 0.0789060921194737 4.91282851954297E-4
68.2268226822682 0.0788958450848576 4.91091477428276E-4
68.2468246824682 0.0788856025773515 4.90900237123223E-4
68.2668266826683 0.0788753645935456 4.90709130903206E-4
68.2868286828683 0.0788651311300316 4.90518158632434E-4
68.3068306830683 0.0788549021834055 4.90327320175304E-4
68.3268326832683 0.0788446777502647 4.90136615396357E-4
68.3468346834683 0.0788344578272114 4.89946044160326E-4
68.3668366836684 0.0788242424108486 4.89755606332081E-4
68.3868386838684 0.0788140314977835 4.89565301776678E-4
68.4068406840684 0.0788038250846254 4.89375130359317E-4
68.4268426842684 0.0787936231679871 4.89185091945385E-4
68.4468446844684 0.0787834257444836 4.88995186400412E-4
68.4668466846685 0.0787732328107334 4.88805413590107E-4
68.4868486848685 0.0787630443633568 4.88615773380326E-4
68.5068506850685 0.0787528603989782 4.88426265637108E-4
68.5268526852685 0.078742680914224 4.88236890226641E-4
68.5468546854685 0.0787325059057235 4.88047647015274E-4

68.5668566856686 0.0787223353701091 4.8785853586953E-4
68.5868586858686 0.0787121693040162 4.87669556656091E-4
68.6068606860686 0.0787020077040822 4.87480709241789E-4
68.6268626862686 0.078691850566948 4.8729199349363E-4
68.6468646864686 0.0786816978892573 4.8710340927878E-4
68.6668666866687 0.0786715496676562 4.86914956464564E-4
68.6868686868687 0.078661405898794 4.86726634918465E-4
68.7068706870687 0.0786512665793229 4.86538444508138E-4
68.7268726872687 0.078641131705897 4.8635038510138E-4
68.7468746874687 0.0786310012751747 4.86162456566173E-4
68.7668766876688 0.0786208752838157 4.85974658770631E-4
68.7868786878688 0.0786107537284837 4.85786991583056E-4
68.8068806880688 0.0786006366058443 4.85599454871887E-4
68.8268826882688 0.0785905239125665 4.85412048505739E-4
68.8468846884688 0.0785804156453215 4.85224772353371E-4
68.8668866886689 0.0785703118007842 4.85037626283718E-4
68.8868886888689 0.078560212375631 4.84850610165856E-4
68.9068906890689 0.0785501173665424 4.84663723869037E-4
68.9268926892689 0.0785400267702009 4.84476967262659E-4
68.9468946894689 0.0785299405832917 4.84290340216281E-4
68.966896689669 0.0785198588025036 4.8410384259963E-4
68.986898689869 0.078509781424527 4.83917474282571E-4
69.006900690069 0.078499708446056 4.83731235135143E-4
69.026902690269 0.0784896398637871 4.8354512502754E-4
69.046904690469 0.0784795756744196 4.83359143830106E-4
69.0669066906691 0.0784695158746554 4.83173291413345E-4
69.0869086908691 0.0784594604611995 4.82987567647921E-4
69.1069106910691 0.0784494094307599 4.8280197240466E-4
69.1269126912691 0.0784393627800459 4.82616505554518E-4
69.1469146914691 0.0784293205057714 4.82431166968641E-4
69.1669166916692 0.0784192826046522 4.82245956518314E-4
69.1869186918692 0.0784092490734067 4.82060874074976E-4
69.2069206920692 0.0783992199087561 4.8187591951022E-4
69.2269226922692 0.0783891951074247 4.81691092695805E-4
69.2469246924693 0.0783791746661394 4.8150639350364E-4
69.2669266926693 0.0783691585816298 4.81321821805786E-4
69.2869286928693 0.0783591468506279 4.81137377474455E-4
69.3069306930693 0.0783491394698691 4.80953060382027E-4
69.3269326932693 0.0783391364360906 4.80768870401015E-4
69.3469346934694 0.0783291377460334 4.80584807404111E-4
69.3669366936694 0.0783191433964406 4.80400871264142E-4
69.3869386938694 0.078309153384058 4.80217061854094E-4
69.4069406940694 0.0782991677056346 4.80033379047111E-4
69.4269426942694 0.0782891863579215 4.79849822716485E-4

69.4469446944695 0.0782792093376729 4.79666392735659E-4
69.4669466946695 0.0782692366416455 4.79483088978233E-4
69.4869486948695 0.0782592682665991 4.79299911317959E-4
69.5069506950695 0.0782493042092958 4.79116859628742E-4
69.5269526952695 0.0782393444665004 4.78933933784631E-4
69.5469546954696 0.0782293890349809 4.78751133659841E-4
69.5669566956696 0.0782194379115073 4.78568459128723E-4
69.5869586958696 0.0782094910928531 4.78385910065797E-4
69.6069606960696 0.0781995485757936 4.78203486345716E-4
69.6269626962696 0.0781896103571074 4.78021187843293E-4
69.6469646964697 0.0781796764335758 4.77839014433494E-4
69.6669666966697 0.0781697468019826 4.77656965991435E-4
69.6869686968697 0.0781598214591144 4.77475042392378E-4
69.7069706970697 0.0781499004017605 4.7729324351174E-4
69.7269726972697 0.0781399836267123 4.77111569225075E-4
69.7469746974698 0.078130071130765 4.7693001940811E-4
69.7669766976698 0.0781201629107155 4.76748593936703E-4
69.7869786978698 0.078110258963364 4.7656729268687E-4
69.8069806980698 0.0781003592855131 4.7638611553477E-4
69.8269826982698 0.0780904638739677 4.76205062356713E-4
69.8469846984699 0.0780805727255366 4.76024133029169E-4
69.8669866986699 0.0780706858370297 4.75843327428736E-4
69.8869886988699 0.0780608032052605 4.75662645432173E-4
69.9069906990699 0.0780509248270455 4.75482086916393E-4
69.9269926992699 0.0780410506992024 4.75301651758437E-4
69.94699469947 0.0780311808185533 4.75121339835517E-4
69.96699669967 0.0780213151819219 4.74941151024976E-4
69.98699869987 0.0780114537861348 4.74761085204312E-4
70.00700070007 0.0780015966280212 4.74581142251165E-4
70.02700270027 0.0779917437044133 4.74401322043331E-4
70.0470047004701 0.0779818950121454 4.74221624458739E-4
70.0670067006701 0.0779720505480548 4.7404204937548E-4
70.0870087008701 0.0779622103089816 4.73862596671781E-4
70.1070107010701 0.0779523742917679 4.73683266226015E-4
70.1270127012701 0.0779425424932593 4.7350405791671E-4
70.1470147014702 0.0779327149103032 4.73324971622527E-4
70.1670167016702 0.0779228915397504 4.73146007222286E-4
70.1870187018702 0.0779130723784538 4.72967164594943E-4
70.2070207020702 0.0779032574232691 4.72788443619601E-4
70.2270227022702 0.0778934466710545 4.72609844175508E-4
70.2470247024703 0.077883640118671 4.72431366142059E-4
70.2670267026703 0.0778738377629825 4.72253009398796E-4
70.2870287028703 0.077864039600855 4.72074773825398E-4
70.3070307030703 0.0778542456291574 4.71896659301694E-4

70.3270327032703 0.0778444558447607 4.71718665707649E-4
70.3470347034704 0.0778346702445398 4.7154079292339E-4
70.3670367036704 0.0778248888253707 4.71363040829164E-4
70.3870387038704 0.077815111584133 4.7118540930538E-4
70.4070407040704 0.0778053385177086 4.71007898232581E-4
70.4270427042704 0.0777955696229816 4.70830507491449E-4
70.4470447044705 0.0777858048968399 4.70653236962828E-4
70.4670467046705 0.0777760443361728 4.70476086527685E-4
70.4870487048705 0.0777662879378726 4.70299056067135E-4
70.5070507050705 0.0777565356988341 4.70122145462437E-4
70.5270527052705 0.0777467876159553 4.69945354594998E-4
70.5470547054705 0.0777370436861359 4.69768683346352E-4
70.5670567056706 0.0777273039062789 4.6959213159819E-4
70.5870587058706 0.0777175682732896 4.69415699232337E-4
70.6070607060706 0.0777078367840756 4.69239386130756E-4
70.6270627062706 0.0776981094355477 4.69063192175561E-4
70.6470647064706 0.0776883862246189 4.68887117248998E-4
70.6670667066707 0.0776786671482049 4.6871116123346E-4
70.6870687068707 0.077668952203224 4.68535324011479E-4
70.7070707070707 0.0776592413865967 4.68359605465718E-4
70.7270727072707 0.0776495346952468 4.68184005478999E-4
70.7470747074707 0.0776398321261003 4.68008523934272E-4
70.7670767076708 0.077630133676085 4.67833160714616E-4
70.7870787078708 0.0776204393421333 4.67657915703281E-4
70.8070807080708 0.0776107491211778 4.67482788783624E-4
70.8270827082708 0.0776010630101551 4.67307779839158E-4
70.8470847084708 0.0775913810060042 4.67132888753534E-4
70.8670867086709 0.0775817031056665 4.66958115410542E-4
70.8870887088709 0.0775720293060858 4.66783459694104E-4
70.9070907090709 0.0775623596042086 4.66608921488288E-4
70.9270927092709 0.0775526939969842 4.66434500677299E-4
70.9470947094709 0.0775430324813638 4.66260197145471E-4
70.967096709671 0.0775333750543021 4.66086010777296E-4
70.987098709871 0.0775237217127553 4.65911941457379E-4
71.007100710071 0.0775140724536832 4.65737989070485E-4
71.027102710271 0.0775044272740471 4.65564153501498E-4
71.047104710471 0.077494786170812 4.6539043463546E-4
71.0671067106711 0.0774851491409443 4.65216832357527E-4
71.0871087108711 0.0774755161814138 4.65043346553012E-4
71.1071107110711 0.0774658872891921 4.64869977107346E-4
71.1271127112711 0.0774562624612538 4.64696723906107E-4
71.1471147114711 0.0774466416945763 4.64523586835016E-4
71.1671167116712 0.0774370249861392 4.64350565779923E-4
71.1871187118712 0.0774274123329244 4.64177660626807E-4

71.2071207120712 0.0774178037319164 4.6400487126179E-4
71.2271227122712 0.0774081991801029 4.63832197571137E-4
71.2471247124712 0.0773985986744732 4.63659639441233E-4
71.2671267126713 0.07738900221202 4.63487196758614E-4
71.2871287128713 0.0773794097897374 4.63314869409932E-4
71.3071307130713 0.0773698214046231 4.63142657281992E-4
71.3271327132713 0.0773602370536769 4.62970560261727E-4
71.3471347134713 0.0773506567339007 4.62798578236196E-4
71.3671367136714 0.0773410804423 4.62626711092614E-4
71.3871387138714 0.0773315081758818 4.62454958718312E-4
71.4071407140714 0.077321939931656 4.62283321000759E-4
71.4271427142714 0.0773123757066348 4.62111797827556E-4
71.4471447144714 0.0773028154978333 4.61940389086445E-4
71.4671467146715 0.0772932593022685 4.61769094665292E-4
71.4871487148715 0.0772837071169608 4.61597914452107E-4
71.5071507150715 0.0772741589389321 4.61426848335025E-4
71.5271527152715 0.0772646147652075 4.61255896202316E-4
71.5471547154715 0.0772550745928145 4.61085057942387E-4
71.5671567156716 0.0772455384187826 4.60914333443768E-4
71.5871587158716 0.0772360062401446 4.60743722595134E-4
71.6071607160716 0.0772264780539347 4.60573225285278E-4
71.6271627162716 0.0772169538571908 4.6040284140314E-4
71.6471647164717 0.0772074336469525 4.60232570837781E-4
71.6671667166717 0.0771979174202622 4.60062413478399E-4
71.6871687168717 0.0771884051741647 4.59892369214322E-4
71.7071707170717 0.077178896905707 4.59722437935009E-4
71.7271727172717 0.077169392611939 4.59552619530048E-4
71.7471747174718 0.077159892289913 4.59382913889167E-4
71.7671767176718 0.0771503959366837 4.59213320902214E-4
71.7871787178718 0.0771409035493082 4.59043840459176E-4
71.8071807180718 0.0771314151248464 4.58874472450167E-4
71.8271827182718 0.0771219306603601 4.5870521676543E-4
71.8471847184719 0.0771124501529138 4.58536073295335E-4
71.8671867186719 0.0771029735995747 4.58367041930393E-4
71.8871887188719 0.0770935009974125 4.58198122561237E-4
71.9071907190719 0.077084032343499 4.58029315078633E-4
71.9271927192719 0.0770745676349084 4.57860619373469E-4
71.947194719472 0.0770651068687181 4.57692035336776E-4
71.967196719672 0.077055650042007 4.575235628597E-4
71.987198719872 0.0770461971518574 4.5735520183353E-4
72.007200720072 0.0770367481953526 4.57186952149661E-4
72.027202720272 0.0770273031695806 4.57018813699652E-4
72.0472047204721 0.0770178620716297 4.56850786375158E-4
72.0672067206721 0.0770084248985916 4.56682870067977E-4

72.0872087208721 0.0769989916475606 4.56515064670034E-4
72.1072107210721 0.0769895623156329 4.56347370073379E-4
72.1272127212721 0.0769801368999078 4.56179786170197E-4
72.1472147214722 0.0769707153974865 4.56012312852793E-4
72.1672167216722 0.0769612978054727 4.55844950013601E-4
72.1872187218722 0.0769518841209727 4.55677697545182E-4
72.2072207220722 0.0769424743410952 4.55510555340227E-4
72.2272227222722 0.0769330684629517 4.55343523291561E-4
72.2472247224723 0.0769236664836549 4.55176601292111E-4
72.2672267226723 0.076914268400322 4.55009789234967E-4
72.2872287228723 0.0769048742100701 4.54843087013306E-4
72.3072307230723 0.0768954839100208 4.54676494520464E-4
72.3272327232723 0.0768860974972969 4.54510011649883E-4
72.3472347234724 0.0768767149690249 4.54343638295149E-4
72.3672367236724 0.0768673363223323 4.54177374349957E-4
72.3872387238724 0.0768579615543493 4.54011219708127E-4
72.4072407240724 0.0768485906622096 4.53845174263624E-4
72.4272427242724 0.0768392236430481 4.53679237910519E-4
72.4472447244725 0.0768298604940025 4.53513410543014E-4
72.4672467246725 0.0768205012122134 4.53347692055445E-4
72.4872487248725 0.0768111457948227 4.53182082342252E-4
72.5072507250725 0.0768017942389761 4.53016581298026E-4
72.5272527252725 0.0767924465418207 4.52851188817464E-4
72.5472547254726 0.0767831027005061 4.5268590479539E-4
72.5672567256726 0.0767737627121848 4.5252072912676E-4
72.5872587258726 0.0767644265740112 4.52355661706643E-4
72.6072607260726 0.0767550942831425 4.52190702430248E-4
72.6272627262726 0.0767457658367382 4.52025851192891E-4
72.6472647264727 0.0767364412319596 4.51861107890016E-4
72.6672667266727 0.0767271204659714 4.51696472417201E-4
72.6872687268727 0.0767178035359398 4.5153194467013E-4
72.7072707270727 0.0767084904390342 4.51367524544627E-4
72.7272727272727 0.0766991811724255 4.51203211936628E-4
72.7472747274728 0.0766898757332879 4.51039006742196E-4
72.7672767276728 0.0766805741187973 4.50874908857518E-4
72.7872787278728 0.0766712763261321 4.50710918178896E-4
72.8072807280728 0.0766619823524731 4.50547034602758E-4
72.8272827282728 0.0766526921950042 4.50383258025667E-4
72.8472847284729 0.0766434058509105 4.50219588344286E-4
72.8672867286729 0.0766341233173801 4.50056025455414E-4
72.8872887288729 0.0766248445916036 4.49892569255972E-4
72.9072907290729 0.0766155696707736 4.49729219642994E-4
72.9272927292729 0.0766062985520853 4.49565976513641E-4
72.947294729473 0.0765970312327363 4.49402839765197E-4

72.967296729673 0.0765877677099266 4.49239809295066E-4
72.987298729873 0.076578507980858 4.49076885000765E-4
73.007300730073 0.0765692520427356 4.48914066779945E-4
73.027302730273 0.0765599998927662 4.48751354530368E-4
73.047304730473 0.076550751528159 4.48588748149918E-4
73.0673067306731 0.076541506946126 4.48426247536607E-4
73.0873087308731 0.0765322661438809 4.4826385258855E-4
73.1073107310731 0.0765230291186405 4.48101563204004E-4
73.1273127312731 0.076513795867623 4.47939379281325E-4
73.1473147314731 0.0765045663880503 4.47777300719008E-4
73.1673167316732 0.0764953406771453 4.47615327415651E-4
73.1873187318732 0.0764861187321339 4.4745345926998E-4
73.2073207320732 0.0764769005502447 4.47291696180844E-4
73.2273227322732 0.0764676861287074 4.47130038047193E-4
73.2473247324732 0.0764584754647557 4.46968484768122E-4
73.2673267326733 0.0764492685556244 4.46807036242825E-4
73.2873287328733 0.0764400653985511 4.46645692370624E-4
73.3073307330733 0.0764308659907754 4.46484453050947E-4
73.3273327332733 0.0764216703295398 4.46323318183358E-4
73.3473347334733 0.0764124784120889 4.46162287667531E-4
73.3673367336734 0.0764032902356697 4.46001361403258E-4
73.3873387338734 0.0763941057975311 4.45840539290446E-4
73.4073407340734 0.0763849250949249 4.45679821229126E-4
73.4273427342734 0.0763757481251048 4.45519207119438E-4
73.4473447344734 0.0763665748853268 4.45358696861644E-4
73.4673467346735 0.0763574053728501 4.45198290356132E-4
73.4873487348735 0.076348239584935 4.45037987503391E-4
73.5073507350735 0.0763390775188447 4.44877788204035E-4
73.5273527352735 0.0763299191718448 4.44717692358795E-4
73.5473547354735 0.0763207645412035 4.44557699868526E-4
73.5673567356736 0.0763116136241902 4.44397810634179E-4
73.5873587358736 0.0763024664180782 4.44238024556849E-4
73.6073607360736 0.0762933229201414 4.44078341537716E-4
73.6273627362736 0.0762841831276572 4.43918761478098E-4
73.6473647364736 0.0762750470379053 4.43759284279429E-4
73.6673667366737 0.0762659146481675 4.43599909843256E-4
73.6873687368737 0.0762567859557271 4.43440638071224E-4
73.7073707370737 0.0762476609578708 4.43281468865118E-4
73.7273727372737 0.0762385396518875 4.43122402126829E-4
73.7473747374737 0.0762294220350675 4.42963437758356E-4
73.7673767376738 0.0762203081047045 4.42804575661827E-4
73.7873787378738 0.0762111978580937 4.4264581573947E-4
73.8073807380738 0.0762020912925334 4.42487157893645E-4
73.8273827382738 0.0761929884053234 4.42328602026811E-4

73.8473847384738 0.076183889193766 4.42170148041547E-4
73.8673867386739 0.0761747936551661 4.42011795840548E-4
73.8873887388739 0.0761657017868305 4.4185354532662E-4
73.9073907390739 0.0761566135860685 4.41695396402682E-4
73.9273927392739 0.0761475290501921 4.4153734897178E-4
73.947394739474 0.0761384481765147 4.41379402937056E-4
73.967396739674 0.0761293709623526 4.41221558201773E-4
73.987398739874 0.0761202974050244 4.41063814669311E-4
74.007400740074 0.0761112275018503 4.40906172243154E-4
74.027402740274 0.0761021612501537 4.40748630826908E-4
74.0474047404741 0.0760930986472602 4.40591190324296E-4
74.0674067406741 0.0760840396904964 4.4043385063913E-4
74.0874087408741 0.0760749843771931 4.40276611675368E-4
74.1074107410741 0.0760659327046817 4.40119473337054E-4
74.1274127412741 0.0760568846702968 4.39962435528357E-4
74.1474147414742 0.0760478402713753 4.39805498153559E-4
74.1674167416742 0.0760387995052557 4.39648661117046E-4
74.1874187418742 0.0760297623692792 4.3949192432332E-4
74.2074207420742 0.0760207288607897 4.39335287677003E-4
74.2274227422742 0.0760116989771329 4.39178751082824E-4
74.2474247424743 0.076002672715656 4.39022314445603E-4
74.2674267426743 0.0759936500737097 4.38865977670303E-4
74.2874287428743 0.0759846310486467 4.38709740661984E-4
74.3074307430743 0.0759756156378217 4.38553603325819E-4
74.3274327432743 0.0759666038385916 4.38397565567089E-4
74.3474347434744 0.0759575956483153 4.3824162729118E-4
74.3674367436744 0.075948591064355 4.38085788403611E-4
74.3874387438744 0.0759395900840739 4.37930048809987E-4
74.4074407440744 0.0759305927048385 4.37774408416039E-4
74.4274427442744 0.0759215989240168 4.376188671276E-4
74.4474447444745 0.075912608738979 4.37463424850612E-4
74.4674467446745 0.0759036221470986 4.37308081491141E-4
74.4874487448745 0.0758946391457501 4.37152836955347E-4
74.5074507450745 0.0758856597323107 4.36997691149503E-4
74.5274527452745 0.0758766839041605 4.36842643980006E-4
74.5474547454746 0.0758677116586804 4.36687695353337E-4
74.5674567456746 0.0758587429932549 4.36532845176109E-4
74.5874587458746 0.0758497779052701 4.36378093355032E-4
74.6074607460746 0.0758408163921146 4.36223439796933E-4
74.6274627462746 0.0758318584511785 4.36068884408734E-4
74.6474647464747 0.0758229040798552 4.35914427097482E-4
74.6674667466747 0.0758139532755401 4.35760067770332E-4
74.6874687468747 0.0758050060356296 4.35605806334524E-4
74.7074707470747 0.0757960623575245 4.35451642697444E-4

74.7274727472747 0.0757871222386259 4.35297576766555E-4
74.7474747474748 0.0757781856763378 4.35143608449437E-4
74.7674767476748 0.0757692526680668 4.34989737653787E-4
74.7874787478748 0.0757603232112214 4.34835964287403E-4
74.8074807480748 0.075751397303212 4.34682288258184E-4
74.8274827482748 0.0757424749414516 4.34528709474147E-4
74.8474847484749 0.0757335561233555 4.34375227843413E-4
74.8674867486749 0.0757246408463412 4.34221843274215E-4
74.8874887488749 0.075715729107828 4.3406855567488E-4
74.9074907490749 0.0757068209052377 4.33915364953854E-4
74.9274927492749 0.0756979162359944 4.33762271019686E-4
74.947494749475 0.0756890150975242 4.3360927378103E-4
74.967496749675 0.0756801174872556 4.33456373146651E-4
74.987498749875 0.0756712234026194 4.33303569025419E-4
75.007500750075 0.0756623328410482 4.33150861326308E-4
75.027502750275 0.0756534457999774 4.32998249958404E-4
75.0475047504751 0.0756445622768436 4.32845734830884E-4
75.0675067506751 0.0756356822690867 4.3269331585305E-4
75.0875087508751 0.0756268057741482 4.32540992934301E-4
75.1075107510751 0.0756179327894719 4.3238876598414E-4
75.1275127512751 0.075609063312504 4.3223663491218E-4
75.1475147514752 0.0756001973406928 4.32084599628139E-4
75.1675167516752 0.0755913348714882 4.31932660041829E-4
75.1875187518752 0.0755824759023434 4.31780816063189E-4
75.2075207520752 0.0755736204307129 4.31629067602244E-4
75.2275227522752 0.0755647684540535 4.31477414569129E-4
75.2475247524753 0.075555919969825 4.31325856874094E-4
75.2675267526753 0.0755470749754884 4.3117439442748E-4
75.2875287528753 0.0755382334685068 4.3102302713973E-4
75.3075307530753 0.0755293954463466 4.30871754921411E-4
75.3275327532753 0.0755205609064757 4.3072057768318E-4
75.3475347534754 0.0755117298463637 4.30569495335794E-4
75.3675367536754 0.075502902263483 4.30418507790122E-4
75.3875387538754 0.0754940781553085 4.30267614957142E-4
75.4075407540754 0.0754852575193168 4.3011681674793E-4
75.4275427542754 0.075476440352986 4.29966113073651E-4
75.4475447544754 0.0754676266537979 4.29815503845603E-4
75.4675467546755 0.0754588164192349 4.29664988975159E-4
75.4875487548755 0.0754500096467828 4.29514568373816E-4
75.5075507550755 0.0754412063339288 4.29364241953158E-4
75.5275527552755 0.0754324064781626 4.29214009624882E-4
75.5475547554755 0.0754236100769764 4.29063871300793E-4
75.5675567556756 0.0754148171278639 4.28913826892788E-4
75.5875587558756 0.0754060276283211 4.28763876312863E-4

75.6075607560756 0.0753972415758466 4.28614019473133E-4
75.6275627562756 0.0753884589679405 4.28464256285796E-4
75.6475647564756 0.0753796798021057 4.28314586663169E-4
75.6675667566757 0.0753709040758468 4.28165010517659E-4
75.6875687568757 0.0753621317866711 4.28015527761788E-4
75.7075707570757 0.0753533629320871 4.27866138308159E-4
75.7275727572757 0.0753445975096066 4.27716842069502E-4
75.7475747574757 0.0753358355167427 4.27567638958626E-4
75.7675767576758 0.0753270769510109 4.27418528888457E-4
75.7875787578758 0.0753183218099291 4.27269511772016E-4
75.8075807580758 0.075309570091017 4.27120587522423E-4
75.8275827582758 0.0753008217917965 4.26971756052902E-4
75.8475847584758 0.075292076909792 4.26823017276784E-4
75.8675867586759 0.0752833354425295 4.26674371107487E-4
75.8875887588759 0.0752745973875378 4.26525817458545E-4
75.9075907590759 0.0752658627423469 4.26377356243577E-4
75.9275927592759 0.0752571315044899 4.26228987376316E-4
75.9475947594759 0.0752484036715018 4.26080710770592E-4
75.967596759676 0.0752396792409187 4.25932526340319E-4
75.987598759876 0.0752309582102805 4.25784433999537E-4
76.007600760076 0.0752222405771281 4.25636433662371E-4
76.027602760276 0.0752135263390051 4.2548852524305E-4
76.047604760476 0.0752048154934569 4.25340708655901E-4
76.0676067606761 0.0751961080380307 4.25192983815342E-4
76.0876087608761 0.075187403970277 4.25045350635915E-4
76.1076107610761 0.0751787032877471 4.24897809032235E-4
76.1276127612761 0.0751700059879953 4.2475035891903E-4
76.1476147614761 0.0751613120685776 4.24603000211123E-4
76.1676167616762 0.0751526215270523 4.24455732823437E-4
76.1876187618762 0.0751439343609796 4.24308556670995E-4
76.2076207620762 0.0751352505679221 4.24161471668913E-4
76.2276227622762 0.0751265701454445 4.24014477732416E-4
76.2476247624762 0.0751178930911136 4.23867574776823E-4
76.2676267626763 0.0751092194024976 4.23720762717536E-4
76.2876287628763 0.075100549077168 4.23574041470079E-4
76.3076307630763 0.0750918821126978 4.23427410950066E-4
76.3276327632763 0.0750832185066626 4.23280871073213E-4
76.3476347634764 0.0750745582566386 4.23134421755308E-4
76.3676367636764 0.0750659013602061 4.22988062912275E-4
76.3876387638764 0.0750572478149461 4.22841794460106E-4
76.4076407640764 0.0750485976184425 4.22695616314908E-4
76.4276427642764 0.0750399507682804 4.22549528392871E-4
76.4476447644765 0.0750313072620486 4.22403530610304E-4
76.4676467646765 0.0750226670973366 4.22257622883595E-4

76.4876487648765 0.075014030271736 4.22111805129224E-4
76.5076507650765 0.0750053967828413 4.21966077263788E-4
76.5276527652765 0.0749967666282484 4.21820439203964E-4
76.5476547654766 0.0749881398055558 4.21674890866534E-4
76.5676567656766 0.0749795163123641 4.21529432168378E-4
76.5876587658766 0.0749708961462754 4.21384063026464E-4
76.6076607660766 0.0749622793048942 4.21238783357857E-4
76.6276627662766 0.0749536657858277 4.21093593079735E-4
76.6476647664767 0.0749450555866841 4.20948492109349E-4
76.6676667666767 0.0749364487050747 4.20803480364062E-4
76.6876687668767 0.0749278451386121 4.20658557761322E-4
76.7076707670767 0.0749192448849113 4.20513724218681E-4
76.7276727672767 0.0749106479415896 4.20368979653784E-4
76.7476747674768 0.074902054306266 4.20224323984368E-4
76.7676767676768 0.0748934639765618 4.20079757128269E-4
76.7876787678768 0.0748848769501003 4.19935279003419E-4
76.8076807680768 0.0748762932245074 4.19790889527851E-4
76.8276827682768 0.0748677127974098 4.1964658861967E-4
76.8476847684769 0.0748591356664373 4.19502376197098E-4
76.8676867686769 0.074850561829222 4.19358252178452E-4
76.8876887688769 0.0748419912833968 4.19214216482124E-4
76.9076907690769 0.0748334240265984 4.19070269026627E-4
76.9276927692769 0.0748248600564642 4.18926409730551E-4
76.947694769477 0.074816299370634 4.18782638512579E-4
76.967696769677 0.0748077419667497 4.18638955291492E-4
76.987698769877 0.0747991878424558 4.18495359986175E-4
77.007700770077 0.0747906369953982 4.18351852515596E-4
77.027702770277 0.0747820894232247 4.1820843279881E-4
77.0477047704771 0.0747735451235863 4.18065100754989E-4
77.0677067706771 0.0747650040941346 4.17921856303373E-4
77.0877087708771 0.0747564663325245 4.17778699363318E-4
77.1077107710771 0.0747479318364121 4.17635629854252E-4
77.1277127712771 0.0747394006034559 4.17492647695713E-4
77.1477147714772 0.0747308726313167 4.17349752807326E-4
77.1677167716772 0.0747223479176571 4.17206945108812E-4
77.1877187718772 0.0747138264601407 4.17064224519964E-4
77.2077207720772 0.0747053082564355 4.16921590960707E-4
77.2277227722772 0.0746967933042096 4.16779044351025E-4
77.2477247724773 0.0746882816011342 4.16636584611016E-4
77.2677267726773 0.0746797731448818 4.16494211660856E-4
77.2877287728773 0.074671267933127 4.16351925420814E-4
77.3077307730773 0.0746627659635473 4.16209725811267E-4
77.3277327732773 0.0746542672338213 4.16067612752663E-4
77.3477347734774 0.0746457717416303 4.1592558616556E-4

77.3677367736774 0.0746372794846572 4.15783645970597E-4
77.3877387738774 0.0746287904605872 4.1564179208851E-4
77.4077407740774 0.0746203046671071 4.15500024440118E-4
77.4277427742774 0.0746118221019062 4.15358342946341E-4
77.4477447744775 0.074603342762676 4.15216747528194E-4
77.4677467746775 0.0745948666471095 4.15075238106772E-4
77.4877487748775 0.0745863937529019 4.14933814603264E-4
77.5077507750775 0.0745779240777506 4.14792476938956E-4
77.5277527752775 0.0745694576193548 4.14651225035218E-4
77.5477547754776 0.0745609943754163 4.14510058813523E-4
77.5677567756776 0.0745525343436377 4.14368978195411E-4
77.5877587758776 0.0745440775217249 4.14227983102537E-4
77.6077607760776 0.0745356239073855 4.14087073456639E-4
77.6277627762776 0.0745271734983289 4.13946249179544E-4
77.6477647764777 0.0745187262922663 4.13805510193165E-4
77.6677667766777 0.0745102822869114 4.13664856419509E-4
77.6877687768777 0.0745018414799795 4.13524287780674E-4
77.7077707770777 0.0744934038691883 4.13383804198849E-4
77.7277727772777 0.0744849694522575 4.13243405596312E-4
77.7477747774778 0.0744765382269087 4.13103091895433E-4
77.7677767776778 0.0744681101908653 4.12962863018664E-4
77.7877787778778 0.0744596853418525 4.12822718888545E-4
77.8077807780778 0.0744512636775985 4.12682659427722E-4
77.8277827782778 0.0744428451958331 4.12542684558923E-4
77.8477847784778 0.0744344298942877 4.12402794204959E-4
77.8677867786779 0.0744260177706955 4.12262988288728E-4
77.8877887788779 0.0744176088227924 4.12123266733224E-4
77.9077907790779 0.0744092030483165 4.11983629461536E-4
77.9277927792779 0.074400800445007 4.11844076396829E-4
77.9477947794779 0.0743924010106055 4.11704607462361E-4
77.967796779678 0.0743840047428561 4.11565222581486E-4
77.987798779878 0.0743756116395041 4.11425921677634E-4
78.007800780078 0.0743672216982976 4.11286704674337E-4
78.027802780278 0.0743588349169856 4.11147571495198E-4
78.047804780478 0.07435045129332 4.11008522063921E-4
78.0678067806781 0.074342070825055 4.10869556304305E-4
78.0878087808781 0.0743336935099454 4.10730674140213E-4
78.1078107810781 0.0743253193457495 4.10591875495622E-4
78.1278127812781 0.0743169483302265 4.10453160294576E-4
78.1478147814781 0.0743085804611387 4.10314528461225E-4
78.1678167816782 0.0743002157362486 4.10175979919783E-4
78.1878187818782 0.0742918541533224 4.10037514594572E-4
78.2078207820782 0.0742834957101282 4.09899132410005E-4
78.2278227822782 0.0742751404044349 4.0976083329056E-4

78.2478247824782 0.074266788234014 4.09622617160812E-4
78.2678267826783 0.0742584391966395 4.09484483945435E-4
78.2878287828783 0.0742500932900866 4.09346433569173E-4
78.3078307830783 0.074241750512133 4.09208465956867E-4
78.3278327832783 0.0742334108605582 4.09070581033442E-4
78.3478347834783 0.0742250743331431 4.089327787239E-4
78.3678367836784 0.0742167409276719 4.08795058953352E-4
78.3878387838784 0.0742084106419298 4.08657421646977E-4
78.4078407840784 0.0742000834737039 4.0851986673004E-4
78.4278427842784 0.0741917594207835 4.08382394127898E-4
78.4478447844784 0.0741834384809605 4.08245003766003E-4
78.4678467846785 0.0741751206520276 4.08107695569871E-4
78.4878487848785 0.0741668059317801 4.07970469465119E-4
78.5078507850785 0.0741584943180158 4.07833325377455E-4
78.5278527852785 0.0741501858085337 4.07696263232662E-4
78.5478547854785 0.0741418804011344 4.07559282956602E-4
78.5678567856786 0.0741335780936215 4.07422384475238E-4
78.5878587858786 0.0741252788837999 4.07285567714609E-4
78.6078607860786 0.0741169827694772 4.07148832600851E-4
78.6278627862786 0.0741086897484618 4.07012179060167E-4
78.6478647864787 0.0741003998185646 4.06875607018855E-4
78.6678667866787 0.0740921129775989 4.06739116403298E-4
78.6878687868787 0.0740838292233795 4.06602707139964E-4
78.7078707870787 0.0740755485537235 4.06466379155411E-4
78.7278727872787 0.0740672709664491 4.06330132376263E-4
78.7478747874788 0.0740589964593775 4.06193966729248E-4
78.7678767876788 0.0740507250303308 4.06057882141164E-4
78.7878787878788 0.0740424566771346 4.05921878538912E-4
78.8078807880788 0.0740341913976146 4.05785955849456E-4
78.8278827882788 0.0740259291896001 4.05650113999861E-4
78.8478847884789 0.0740176700509212 4.05514352917266E-4
78.8678867886789 0.07400941397941 4.0537867252889E-4
78.8878887888789 0.0740011609729014 4.05243072762051E-4
78.9078907890789 0.0739929110292321 4.05107553544147E-4
78.9278927892789 0.0739846641462391 4.04972114802636E-4
78.947894789479 0.0739764203217636 4.04836756465095E-4
78.967896789679 0.0739681795536475 4.04701478459161E-4
78.987898789879 0.0739599418397347 4.0456628071256E-4
79.007900790079 0.0739517071778716 4.04431163153107E-4
79.027902790279 0.073943475565906 4.04296125708694E-4
79.0479047904791 0.0739352470016874 4.04161168307291E-4
79.0679067906791 0.0739270214830681 4.04026290876962E-4
79.0879087908791 0.0739187990079017 4.03891493345851E-4
79.1079107910791 0.0739105795740439 4.03756775642179E-4

79.1279127912791 0.0739023631793523 4.03622137694255E-4
79.1479147914792 0.0738941498216864 4.03487579430467E-4
79.1679167916792 0.0738859394989075 4.03353100779285E-4
79.1879187918792 0.0738777322088794 4.03218701669268E-4
79.2079207920792 0.0738695279494673 4.03084382029054E-4
79.2279227922792 0.0738613267185383 4.02950141787358E-4
79.2479247924793 0.0738531285139614 4.02815980872975E-4
79.2679267926793 0.0738449333336079 4.02681899214797E-4
79.2879287928793 0.0738367411753509 4.02547896741783E-4
79.3079307930793 0.0738285520370653 4.02413973382985E-4
79.3279327932793 0.0738203659166278 4.02280129067525E-4
79.3479347934794 0.0738121828119169 4.02146363724607E-4
79.3679367936794 0.0738040027208137 4.0201267728353E-4
79.3879387938794 0.0737958256412007 4.01879069673665E-4
79.4079407940794 0.0737876515709623 4.01745540824463E-4
79.4279427942794 0.073779480507985 4.0161209066546E-4
79.4479447944795 0.073771312450157 4.01478719126269E-4
79.4679467946795 0.0737631473953684 4.01345426136584E-4
79.4879487948795 0.0737549853415116 4.01212211626186E-4
79.5079507950795 0.0737468262864806 4.01079075524932E-4
79.5279527952795 0.073738670228171 4.00946017762755E-4
79.5479547954796 0.0737305171644815 4.00813038269688E-4
79.5679567956796 0.0737223670933107 4.00680136975809E-4
79.5879587958796 0.0737142200125611 4.00547313811316E-4
79.6079607960796 0.0737060759201355 4.00414568706453E-4
79.6279627962796 0.0736979348139406 4.00281901591581E-4
79.6479647964797 0.0736897966918824 4.00149312397097E-4
79.6679667966797 0.0736816615518705 4.00016801053509E-4
79.6879687968797 0.0736735293918162 3.99884367491399E-4
79.7079707970797 0.0736654002096329 3.9975201164143E-4
79.7279727972797 0.0736572740032349 3.99619733434334E-4
79.7479747974798 0.0736491507705396 3.99487532800938E-4
79.7679767976798 0.0736410305094651 3.99355409672129E-4
79.7879787978798 0.0736329132179322 3.99223363978889E-4
79.8079807980798 0.0736247988938634 3.99091395652276E-4
79.8279827982798 0.0736166875351832 3.98959504623427E-4
79.8479847984799 0.0736085791398179 3.98827690823557E-4
79.8679867986799 0.0736004737056955 3.9869595418396E-4
79.8879887988799 0.0735923712307459 3.98564294636004E-4
79.9079907990799 0.0735842717129012 3.98432712111148E-4
79.9279927992799 0.0735761751500949 3.98301206540914E-4
79.94799479948 0.0735680815402631 3.98169777856919E-4
79.96799679968 0.0735599908813429 3.98038425990845E-4
79.98799879988 0.073551903171274 3.9790715087446E-4

80.00800080008 0.0735438184079978 3.97775952439611E-4
80.02800280028 0.0735357365894573 3.9764483061822E-4
80.0480048004801 0.0735276577135969 3.97513785342275E-4
80.0680068006801 0.0735195817783645 3.9738281654387E-4
80.0880088008801 0.0735115087817088 3.97251924155163E-4
80.1080108010801 0.0735034387215799 3.97121108108375E-4
80.1280128012801 0.0734953715959306 3.96990368335828E-4
80.1480148014802 0.0734873074027153 3.96859704769907E-4
80.1680168016802 0.0734792461398903 3.96729117343086E-4
80.1880188018802 0.0734711878054138 3.96598605987906E-4
80.2080208020802 0.0734631323972454 3.96468170636985E-4
80.2280228022802 0.0734550799133476 3.96337811223034E-4
80.2480248024802 0.0734470303516834 3.96207527678818E-4
80.2680268026803 0.073438983710219 3.96077319937198E-4
80.2880288028803 0.0734309399869212 3.959471879311E-4
80.3080308030803 0.0734228991797597 3.95817131593536E-4
80.3280328032803 0.0734148612867056 3.9568715085759E-4
80.3480348034803 0.0734068263057321 3.95557245656427E-4
80.3680368036804 0.0733987942348138 3.9542741592328E-4
80.3880388038804 0.0733907650719273 3.95297661591464E-4
80.4080408040804 0.0733827388150511 3.95167982594368E-4
80.4280428042804 0.0733747154621662 3.95038378865469E-4
80.4480448044804 0.0733666950112542 3.949088503383E-4
80.4680468046805 0.0733586774602998 3.94779396946491E-4
80.4880488048805 0.0733506628072884 3.9465001862373E-4
80.5080508050805 0.0733426510502082 3.94520715303794E-4
80.5280528052805 0.0733346421870486 3.94391486920528E-4
80.5480548054805 0.0733266362158013 3.94262333407858E-4
80.5680568056806 0.0733186331344597 3.94133254699785E-4
80.5880588058806 0.0733106329410188 3.94004250730384E-4
80.6080608060806 0.0733026356334756 3.93875321433802E-4
80.6280628062806 0.0732946412098291 3.9374646674427E-4
80.6480648064806 0.0732866496680797 3.93617686596084E-4
80.6680668066807 0.0732786610062305 3.9348898092363E-4
80.6880688068807 0.0732706752222853 3.93360349661351E-4
80.7080708070807 0.0732626923142506 3.93231792743779E-4
80.7280728072807 0.0732547122801343 3.93103310105517E-4
80.7480748074807 0.0732467351179462 3.92974901681236E-4
80.7680768076808 0.0732387608256986 3.92846567405701E-4
80.7880788078808 0.0732307894014045 3.9271830721373E-4
80.8080808080808 0.0732228208430789 3.9259012104022E-4
80.8280828082808 0.0732148551487399 3.92462008820159E-4
80.8480848084808 0.0732068923164064 3.92333970488595E-4
80.8680868086809 0.0731989323440979 3.92206005980636E-4

80.8880888088809 0.0731909752298385 3.92078115231501E-4
80.9080908090809 0.0731830209716524 3.91950298176459E-4
80.9280928092809 0.0731750695675659 3.91822554750859E-4
80.9480948094809 0.0731671210156066 3.91694884890113E-4
80.968096809681 0.0731591753138053 3.9156728852973E-4
80.988098809881 0.0731512324601929 3.91439765605267E-4
81.008100810081 0.0731432924528034 3.91312316052372E-4
81.028102810281 0.0731353552896724 3.91184939806767E-4
81.0481048104811 0.0731274209688368 3.91057636804236E-4
81.0681068106811 0.0731194894883358 3.90930406980648E-4
81.0881088108811 0.0731115608462103 3.90803250271937E-4
81.1081108110811 0.0731036350405025 3.90676166614111E-4
81.1281128112811 0.0730957120692575 3.90549155943262E-4
81.1481148114812 0.0730877919305211 3.90422218195542E-4
81.1681168116812 0.0730798746223416 3.90295353307181E-4
81.1881188118812 0.0730719601427693 3.90168561214491E-4
81.2081208120812 0.0730640484898552 3.90041841853837E-4
81.2281228122812 0.0730561396616527 3.89915195161667E-4
81.2481248124813 0.0730482336562177 3.89788621074511E-4
81.2681268126813 0.0730403304716071 3.89662119528962E-4
81.2881288128813 0.0730324301058793 3.89535690461676E-4
81.3081308130813 0.073024532557096 3.89409333809411E-4
81.3281328132813 0.0730166378233189 3.89283049508964E-4
81.3481348134814 0.0730087459026127 3.89156837497225E-4
81.3681368136814 0.0730008567930432 3.8903069771115E-4
81.3881388138814 0.0729929704926785 3.88904630087763E-4
81.4081408140814 0.0729850869995881 3.88778634564168E-4
81.4281428142814 0.0729772063118437 3.88652711077539E-4
81.4481448144815 0.0729693284275185 3.88526859565117E-4
81.4681468146815 0.0729614533446873 3.88401079964217E-4
81.4881488148815 0.0729535810614276 3.88275372212234E-4
81.5081508150815 0.0729457115758172 3.88149736246616E-4
81.5281528152815 0.0729378448859372 3.88024172004903E-4
81.5481548154816 0.0729299809898693 3.87898679424691E-4
81.5681568156816 0.0729221198856978 3.87773258443657E-4
81.5881588158816 0.0729142615715086 3.87647908999547E-4
81.6081608160816 0.0729064060453889 3.87522631030172E-4
81.6281628162816 0.0728985533054286 3.87397424473429E-4
81.6481648164817 0.0728907033497181 3.87272289267262E-4
81.6681668166817 0.0728828561763505 3.87147225349705E-4
81.6881688168817 0.0728750117834211 3.87022232658868E-4
81.7081708170817 0.0728671701690251 3.86897311132902E-4
81.7281728172817 0.0728593313312623 3.8677246071007E-4
81.7481748174818 0.0728514952682316 3.8664768132867E-4

81.7681768176818 0.072843661978035 3.86522972927085E-4
81.7881788178818 0.0728358314587765 3.86398335443776E-4
81.8081808180818 0.0728280037085608 3.86273768817257E-4
81.8281828182818 0.0728201787254951 3.86149272986123E-4
81.8481848184819 0.0728123565076888 3.86024847889043E-4
81.8681868186819 0.0728045370532521 3.85900493464747E-4
81.8881888188819 0.0727967203602971 3.85776209652034E-4
81.9081908190819 0.0727889064269386 3.85651996389786E-4
81.9281928192819 0.0727810952512921 3.85527853616939E-4
81.948194819482 0.0727732868314754 3.8540378127251E-4
81.968196819682 0.0727654811656081 3.85279779295583E-4
81.988198819882 0.0727576782518114 3.85155847625309E-4
82.008200820082 0.0727498780882079 3.85031986200906E-4
82.028202820282 0.0727420806729226 3.84908194961668E-4
82.0482048204821 0.0727342860040821 3.8478447384696E-4
82.0682068206821 0.0727264940798143 3.84660822796202E-4
82.0882088208821 0.072718704898249 3.84537241748893E-4
82.1082108210821 0.0727109184575192 3.8441373064462E-4
82.1282128212821 0.0727031347557571 3.84290289422999E-4
82.1482148214822 0.0726953537910984 3.84166918023744E-4
82.1682168216822 0.0726875755616801 3.84043616386629E-4
82.1882188218822 0.0726798000656414 3.83920384451504E-4
82.2082208220822 0.0726720273011219 3.83797222158269E-4
82.2282228222822 0.0726642572662641 3.83674129446908E-4
82.2482248224823 0.0726564899592127 3.8355110625748E-4
82.2682268226823 0.0726487253781128 3.83428152530093E-4
82.2882288228823 0.0726409635211119 3.83305268204936E-4
82.3082308230823 0.0726332043863596 3.83182453222267E-4
82.3282328232823 0.0726254479720061 3.83059707522396E-4
82.3482348234824 0.0726176942762051 3.8293703104573E-4
82.3682368236824 0.0726099432971106 3.82814423732722E-4
82.3882388238824 0.0726021950328783 3.8269188552389E-4
82.4082408240824 0.0725944494816671 3.82569416359841E-4
82.4282428242824 0.072586706641636 3.82447016181233E-4
82.4482448244825 0.0725789665109464 3.82324684928791E-4
82.4682468246825 0.0725712290877617 3.82202422543319E-4
82.4882488248825 0.0725634943702463 3.82080228965675E-4
82.5082508250825 0.0725557623565676 3.81958104136804E-4
82.5282528252825 0.0725480330448932 3.81836047997695E-4
82.5482548254826 0.0725403064333932 3.81714060489416E-4
82.5682568256826 0.0725325825202395 3.81592141553103E-4
82.5882588258826 0.072524861303606 3.81470291129965E-4
82.6082608260826 0.0725171427816675 3.81348509161262E-4
82.6282628262826 0.0725094269526006 3.81226795588326E-4

82.6482648264827 0.072501713814585 3.81105150352575E-4
82.6682668266827 0.0724940033658002 3.80983573395464E-4
82.6882688268827 0.0724862956044287 3.80862064658535E-4
82.7082708270827 0.0724785905286542 3.80740624083391E-4
82.7282728272827 0.0724708881366625 3.80619251611702E-4
82.7482748274827 0.0724631884266405 3.804979471852E-4
82.7682768276828 0.0724554913967773 3.80376710745688E-4
82.7882788278828 0.0724477970452639 3.80255542235039E-4
82.8082808280828 0.0724401053702921 3.8013444159518E-4
82.8282828282828 0.0724324163700571 3.80013408768127E-4
82.8482848284828 0.0724247300427535 3.79892443695929E-4
82.8682868286829 0.0724170463865798 3.79771546320732E-4
82.8882888288829 0.0724093653997348 3.79650716584729E-4
82.9082908290829 0.0724016870804194 3.79529954430186E-4
82.9282928292829 0.072394011426837 3.79409259799443E-4
82.9482948294829 0.0723863384371909 3.79288632634878E-4
82.968296829683 0.0723786681096882 3.79168072878972E-4
82.988298829883 0.0723710004425359 3.79047580474238E-4
83.008300830083 0.072363335433944 3.7892715536328E-4
83.028302830283 0.0723556730821237 3.78806797488752E-4
83.048304830483 0.0723480133852877 3.78686506793379E-4
83.0683068306831 0.0723403563416507 3.7856628321995E-4
83.0883088308831 0.0723327019494286 3.78446126711313E-4
83.1083108310831 0.0723250502068402 3.783260372104E-4
83.1283128312831 0.0723174011121048 3.7820601466019E-4
83.1483148314831 0.0723097546634434 3.78086059003727E-4
83.1683168316832 0.0723021108590799 3.77966170184136E-4
83.1883188318832 0.0722944696972382 3.77846348144583E-4
83.2083208320832 0.0722868311761455 3.77726592828325E-4
83.2283228322832 0.0722791952940298 3.77606904178665E-4
83.2483248324832 0.07227156204912 3.77487282138963E-4
83.2683268326833 0.0722639314396493 3.77367726652683E-4
83.2883288328833 0.0722563034638496 3.77248237663305E-4
83.3083308330833 0.0722486781199566 3.77128815114407E-4
83.3283328332833 0.0722410554062064 3.77009458949614E-4
83.3483348334833 0.0722334353208375 3.76890169112621E-4
83.3683368336834 0.0722258178620899 3.7677094554719E-4
83.3883388338834 0.0722182030282051 3.76651788197139E-4
83.4083408340834 0.0722105908174269 3.76532697006363E-4
83.4283428342834 0.0722029812279994 3.76413671918801E-4
83.4483448344835 0.0721953742581699 3.76294712878472E-4
83.4683468346835 0.0721877699061865 3.76175819829454E-4
83.4883488348835 0.0721801681702998 3.76056992715895E-4
83.5083508350835 0.072172569048761 3.75938231481994E-4

83.5283528352835 0.0721649725398237 3.75819536072022E-4
83.5483548354836 0.0721573786417429 3.75700906430311E-4
83.5683568356836 0.072149787352775 3.75582342501251E-4
83.5883588358836 0.0721421986711791 3.75463844229311E-4
83.6083608360836 0.0721346125952143 3.75345411558998E-4
83.6283628362836 0.0721270291231431 3.7522704443491E-4
83.6483648364837 0.072119448253229 3.75108742801694E-4
83.6683668366837 0.0721118699837364 3.74990506604054E-4
83.6883688368837 0.0721042943129328 3.74872335786773E-4
83.7083708370837 0.0720967212390863 3.74754230294684E-4
83.7283728372837 0.0720891507604666 3.74636190072682E-4
83.7483748374838 0.072081582875346 3.74518215065736E-4
83.7683768376838 0.0720740175819971 3.7440030521886E-4
83.7883788378838 0.0720664548786958 3.74282460477153E-4
83.8083808380838 0.0720588947637188 3.74164680785766E-4
83.8283828382838 0.0720513372353436 3.74046966089898E-4
83.8483848384839 0.0720437822918509 3.73929316334832E-4
83.8683868386839 0.0720362299315224 3.73811731465906E-4
83.8883888388839 0.0720286801526414 3.7369421142852E-4
83.9083908390839 0.0720211329534925 3.73576756168126E-4
83.9283928392839 0.0720135883323624 3.73459365630249E-4
83.948394839484 0.07200604628754 3.73342039760483E-4
83.968396839684 0.0719985068173144 3.73224778504463E-4
83.988398839884 0.0719909699199777 3.73107581807903E-4
84.008400840084 0.071983435593823 3.72990449616573E-4
84.028402840284 0.0719759038371453 3.72873381876307E-4
84.0484048404841 0.0719683746482407 3.7275637853299E-4
84.0684068406841 0.0719608480254079 3.72639439532585E-4
84.0884088408841 0.0719533239669461 3.72522564821101E-4
84.1084108410841 0.0719458024711575 3.72405754344623E-4
84.1284128412841 0.0719382835363448 3.72289008049289E-4
84.1484148414842 0.0719307671608126 3.72172325881292E-4
84.1684168416842 0.0719232533428675 3.72055707786901E-4
84.1884188418842 0.0719157420808174 3.71939153712432E-4
84.2084208420842 0.0719082333729718 3.71822663604268E-4
84.2284228422842 0.0719007272176421 3.71706237408857E-4
84.2484248424843 0.0718932236131412 3.715898750727E-4
84.2684268426843 0.071885722557784 3.7147357654237E-4
84.2884288428843 0.0718782240498859 3.71357341764481E-4
84.3084308430843 0.0718707280877652 3.71241170685727E-4
84.3284328432843 0.0718632346697413 3.71125063252856E-4
84.3484348434844 0.0718557437941356 3.71009019412679E-4
84.3684368436844 0.0718482554592699 3.70893039112052E-4
84.3884388438844 0.0718407696634695 3.7077712229792E-4

84.4084408440844 0.0718332864050596 3.70661268917257E-4
84.4284428442844 0.0718258056823685 3.70545478917126E-4
84.4484448444845 0.0718183274937244 3.70429752244619E-4
84.4684468446845 0.071810851837459 3.70314088846921E-4
84.4884488448845 0.0718033787119039 3.70198488671246E-4
84.5084508450845 0.0717959081153939 3.70082951664895E-4
84.5284528452845 0.0717884400462647 3.6996747777522E-4
84.5484548454846 0.071780974502853 3.69852066949617E-4
84.5684568456846 0.0717735114834984 3.69736719135566E-4
84.5884588458846 0.0717660509865403 3.69621434280579E-4
84.6084608460846 0.071758593010322 3.6950621233226E-4
84.6284628462846 0.0717511375531864 3.69391053238243E-4
84.6484648464847 0.0717436846134796 3.69275956946246E-4
84.6684668466847 0.0717362341895481 3.69160923404028E-4
84.6884688468847 0.0717287862797405 3.69045952559413E-4
84.7084708470847 0.0717213408824072 3.68931044360289E-4
84.7284728472847 0.0717138979958995 3.68816198754592E-4
84.7484748474848 0.0717064576185718 3.68701415690339E-4
84.7684768476848 0.0716990197487778 3.6858669511557E-4
84.7884788478848 0.0716915843848751 3.68472036978422E-4
84.8084808480848 0.0716841515252216 3.68357441227068E-4
84.8284828482848 0.0716767211681768 3.68242907809743E-4
84.8484848484849 0.0716692933121028 3.68128436674752E-4
84.8684868486849 0.071661867955362 3.6801402777044E-4
84.8884888488849 0.0716544450963191 3.67899681045223E-4
84.9084908490849 0.0716470247333408 3.6778539644758E-4
84.9284928492849 0.0716396068647944 3.67671173926036E-4
84.948494849485 0.0716321914890492 3.67557013429175E-4
84.968496849685 0.0716247786044769 3.67442914905655E-4
84.988498849885 0.0716173682094493 3.67328878304172E-4
85.008500850085 0.0716099603023409 3.67214903573492E-4
85.028502850285 0.0716025548815277 3.6710099066244E-4
85.0485048504851 0.071595151945387 3.66987139519896E-4
85.0685068506851 0.0715877514922975 3.66873350094792E-4
85.0885088508851 0.0715803535206394 3.6675962233612E-4
85.1085108510851 0.0715729580287959 3.66645956192948E-4
85.1285128512851 0.0715655650151497 3.66532351614371E-4
85.1485148514851 0.0715581744780863 3.66418808549562E-4
85.1685168516852 0.0715507864159929 3.6630532694775E-4
85.1885188518852 0.0715434008272579 3.66191906758221E-4
85.2085208520852 0.0715360177102715 3.66078547930316E-4
85.2285228522852 0.0715286370634249 3.65965250413427E-4
85.2485248524852 0.0715212588851113 3.65852014157011E-4
85.2685268526853 0.0715138831737258 3.65738839110583E-4

85.2885288528853 0.0715065099276649 3.6562572522372E-4
85.3085308530853 0.0714991391453261 3.6551267244604E-4
85.3285328532853 0.0714917708251083 3.65399680727216E-4
85.3485348534853 0.0714844049654142 3.65286750017018E-4
85.3685368536854 0.071477041564645 3.65173880265221E-4
85.3885388538854 0.0714696806212058 3.65061071421691E-4
85.4085408540854 0.0714623221335022 3.6494832343634E-4
85.4285428542854 0.071454966099941 3.64835636259125E-4
85.4485448544854 0.0714476125189322 3.64723009840092E-4
85.4685468546855 0.0714402613888853 3.64610444129303E-4
85.4885488548855 0.0714329127082125 3.64497939076901E-4
85.5085508550855 0.071425566475328 3.64385494633087E-4
85.5285528552855 0.0714182226886465 3.64273110748108E-4
85.5485548554855 0.0714108813465849 3.6416078737227E-4
85.5685568556856 0.0714035424475614 3.64048524455937E-4
85.5885588558856 0.0713962059899968 3.63936321949542E-4
85.6085608560856 0.0713888719723106 3.63824179803532E-4
85.6285628562856 0.0713815403929278 3.63712097968469E-4
85.6485648564856 0.0713742112502714 3.63600076394919E-4
85.6685668566857 0.0713668845427683 3.63488115033535E-4
85.6885688568857 0.0713595602688459 3.63376213835014E-4
85.7085708570857 0.0713522384269334 3.63264372750112E-4
85.7285728572857 0.0713449190154622 3.63152591729649E-4
85.7485748574858 0.0713376020328644 3.63040870724487E-4
85.7685768576858 0.0713302874775727 3.62929209685533E-4
85.7885788578858 0.0713229753480233 3.62817608563776E-4
85.8085808580858 0.0713156656426541 3.62706067310261E-4
85.8285828582858 0.0713083583599023 3.6259458587606E-4
85.8485848584859 0.0713010534982085 3.62483164212322E-4
85.8685868586859 0.0712937510560139 3.62371802270243E-4
85.8885888588859 0.0712864510317623 3.62260500001086E-4
85.9085908590859 0.0712791534238979 3.62149257356152E-4
85.9285928592859 0.0712718582308668 3.62038074286805E-4
85.948594859486 0.0712645654511176 3.61926950744477E-4
85.968596859686 0.071257275083099 3.61815886680629E-4
85.988598859886 0.0712499871252613 3.61704882046789E-4
86.008600860086 0.0712427015760583 3.61593936794556E-4
86.028602860286 0.0712354184339422 3.61483050875545E-4
86.0486048604861 0.0712281376973702 3.61372224241477E-4
86.0686068606861 0.071220859364798 3.61261456844079E-4
86.0886088608861 0.0712135834346843 3.61150748635155E-4
86.1086108610861 0.0712063099054901 3.61040099566578E-4
86.1286128612861 0.0711990387756757 3.60929509590239E-4
86.1486148614862 0.0711917700437049 3.60818978658114E-4

86.1686168616862 0.0711845037080426 3.60708506722228E-4
86.1886188618862 0.0711772397671544 3.60598093734642E-4
86.2086208620862 0.0711699782195081 3.6048773964749E-4
86.2286228622862 0.0711627190635733 3.60377444412959E-4
86.2486248624863 0.0711554622978203 3.60267207983278E-4
86.2686268626863 0.0711482079207221 3.60157030310749E-4
86.2886288628863 0.0711409559307514 3.60046911347697E-4
86.3086308630863 0.0711337063263839 3.59936851046528E-4
86.3286328632863 0.0711264591060973 3.59826849359706E-4
86.3486348634864 0.0711192142683687 3.59716906239717E-4
86.3686368636864 0.0711119718116786 3.59607021639131E-4
86.3886388638864 0.0711047317345083 3.59497195510558E-4
86.4086408640864 0.0710974940353403 3.59387427806656E-4
86.4286428642864 0.0710902587126599 3.5927771848016E-4
86.4486448644865 0.071083025764953 3.59168067483842E-4
86.4686468646865 0.0710757951907062 3.59058474770515E-4
86.4886488648865 0.0710685669884087 3.58948940293063E-4
86.5086508650865 0.0710613411565507 3.58839464004412E-4
86.5286528652865 0.071054117693625 3.58730045857562E-4
86.5486548654866 0.0710468965981243 3.58620685805538E-4
86.5686568656866 0.0710396778685441 3.58511383801442E-4
86.5886588658866 0.0710324615033806 3.58402139798411E-4
86.6086608660866 0.0710252475011323 3.58292953749652E-4
86.6286628662866 0.0710180358602981 3.58183825608405E-4
86.6486648664867 0.0710108265793792 3.58074755327978E-4
86.6686668666867 0.0710036196568775 3.57965742861714E-4
86.6886688668867 0.0709964150912981 3.57856788163039E-4
86.7086708670867 0.0709892128811463 3.57747891185412E-4
86.7286728672867 0.0709820130249284 3.57639051882334E-4
86.7486748674868 0.0709748155211536 3.57530270207381E-4
86.7686768676868 0.0709676203683313 3.57421546114163E-4
86.7886788678868 0.0709604275649731 3.5731287955635E-4
86.8086808680868 0.070953237109592 3.57204270487668E-4
86.8286828682868 0.070946049000703 3.57095718861896E-4
86.8486848684869 0.0709388632368214 3.56987224632851E-4
86.8686868686869 0.070931679816465 3.56878787754419E-4
86.8886888688869 0.0709244987381526 3.56770408180526E-4
86.9086908690869 0.0709173200004049 3.56662085865161E-4
86.9286928692869 0.0709101436017431 3.56553820762347E-4
86.948694869487 0.0709029695406913 3.56445612826178E-4
86.968696869687 0.0708957978157748 3.56337462010803E-4
86.988698869887 0.0708886284255187 3.56229368270387E-4
87.008700870087 0.0708814613684516 3.56121331559186E-4
87.028702870287 0.0708742966431026 3.56013351831489E-4

87.0487048704871 0.0708671342480028 3.55905429041641E-4
87.0687068706871 0.0708599741816842 3.55797563144038E-4
87.0887088708871 0.070852816442681 3.55689754093131E-4
87.1087108710871 0.0708456610295281 3.55582001843412E-4
87.1287128712871 0.0708385079407622 3.55474306349432E-4
87.1487148714872 0.0708313571749218 3.55366667565795E-4
87.1687168716872 0.0708242087305461 3.55259085447145E-4
87.1887188718872 0.0708170626061769 3.55151559948197E-4
87.2087208720872 0.0708099188003561 3.55044091023691E-4
87.2287228722872 0.0708027773116282 3.54936678628441E-4
87.2487248724873 0.0707956381385385 3.54829322717295E-4
87.2687268726873 0.0707885012796346 3.54722023245171E-4
87.2887288728873 0.0707813667334647 3.54614780167021E-4
87.3087308730873 0.0707742344985782 3.54507593437844E-4
87.3287328732873 0.0707671045735271 3.54400463012707E-4
87.3487348734874 0.0707599769568641 3.54293388846717E-4
87.3687368736874 0.070752851647144 3.54186370895039E-4
87.3887388738874 0.0707457286429223 3.54079409112878E-4
87.4087408740874 0.0707386079427557 3.53972503455488E-4
87.4287428742874 0.0707314895452039 3.53865653878191E-4
87.4487448744875 0.0707243734488264 3.53758860336341E-4
87.4687468746875 0.0707172596521857 3.53652122785358E-4
87.4887488748875 0.0707101481538436 3.53545441180687E-4
87.5087508750875 0.0707030389523658 3.53438815477854E-4
87.5287528752875 0.0706959320463182 3.53332245632419E-4
87.5487548754875 0.0706888274342676 3.53225731599985E-4
87.5687568756876 0.0706817251147833 3.53119273336216E-4
87.5887588758876 0.0706746250864358 3.53012870796828E-4
87.6087608760876 0.0706675273477967 3.52906523937577E-4
87.6287628762876 0.0706604318974396 3.52800232714278E-4
87.6487648764876 0.0706533387339391 3.5269399708279E-4
87.6687668766877 0.070646247855871 3.52587816999018E-4
87.6887688768877 0.0706391592618136 3.5248169241893E-4
87.7087708770877 0.0706320729503452 3.52375623298524E-4
87.7287728772877 0.0706249889200469 3.52269609593868E-4
87.7487748774877 0.0706179071694998 3.52163651261057E-4
87.7687768776878 0.0706108276972885 3.52057748256266E-4
87.7887788778878 0.0706037505019975 3.51951900535696E-4
87.8087808780878 0.0705966755822122 3.5184610805559E-4
87.8287828782878 0.0705896029365211 3.51740370772264E-4
87.8487848784878 0.0705825325635133 3.51634688642071E-4
87.8687868786879 0.0705754644617789 3.51529061621406E-4
87.8887888788879 0.0705683986299106 3.51423489666733E-4
87.9087908790879 0.0705613350665015 3.51317972734545E-4

87.9287928792879 0.0705542737701464 3.51212510781392E-4
87.9487948794879 0.0705472147394414 3.51107103763866E-4
87.968796879688 0.0705401579729844 3.5100175163862E-4
87.988798879888 0.0705331034693749 3.50896454362351E-4
88.008800880088 0.0705260512272136 3.50791211891808E-4
88.028802880288 0.0705190012451015 3.50686024183765E-4
88.048804880488 0.0705119535216432 3.50580891195082E-4
88.0688068806881 0.0705049080554433 3.50475812882644E-4
88.0888088808881 0.0704978648451075 3.50370789203382E-4
88.1088108810881 0.0704908238892444 3.50265820114291E-4
88.1288128812881 0.0704837851864624 3.50160905572399E-4
88.1488148814882 0.0704767487353723 3.50056045534789E-4
88.1688168816882 0.0704697145345862 3.49951239958594E-4
88.1888188818882 0.0704626825827177 3.49846488800998E-4
88.2088208820882 0.0704556528783812 3.49741792019218E-4
88.2288228822882 0.0704486254201932 3.49637149570536E-4
88.2488248824883 0.0704416002067712 3.49532561412271E-4
88.2688268826883 0.0704345772367341 3.49428027501792E-4
88.2888288828883 0.0704275565087028 3.49323547796522E-4
88.3088308830883 0.0704205380212992 3.49219122253929E-4
88.3288328832883 0.0704135217731461 3.49114750831518E-4
88.3488348834884 0.0704065077628684 3.49010433486855E-4
88.3688368836884 0.0703994959890927 3.48906170177555E-4
88.3888388838884 0.0703924864504459 3.48801960861266E-4
88.4088408840884 0.0703854791455572 3.48697805495694E-4
88.4288428842884 0.0703784740730573 3.48593704038597E-4
88.4488448844885 0.0703714712315775 3.48489656447766E-4
88.4688468846885 0.070364470619751 3.4838566268105E-4
88.4888488848885 0.0703574722362119 3.48281722696335E-4
88.5088508850885 0.0703504760795967 3.48177836451566E-4
88.5288528852885 0.0703434821485432 3.48074003904742E-4
88.5488548854886 0.0703364904416893 3.47970225013882E-4
88.5688568856886 0.0703295009576753 3.4786649973707E-4
88.5888588858886 0.0703225136951433 3.47762828032443E-4
88.6088608860886 0.070315528652736 3.47659209858171E-4
88.6288628862886 0.0703085458290971 3.47555645172471E-4
88.6488648864887 0.0703015652228726 3.47452133933612E-4
88.6688668866887 0.0702945868327109 3.4734867609993E-4
88.6888688868887 0.0702876106572587 3.47245271629761E-4
88.7088708870887 0.0702806366951672 3.4714192048153E-4
88.7288728872887 0.0702736649450866 3.47038622613681E-4
88.7488748874888 0.0702666954056703 3.46935377984723E-4
88.7688768876888 0.070259728075572 3.46832186553201E-4
88.7888788878888 0.0702527629534477 3.46729048277715E-4

88.8088808880888 0.0702458000379539 3.46625963116904E-4
88.8288828882888 0.0702388393277484 3.46522931029447E-4
88.8488848884889 0.0702318808214919 3.46419951974093E-4
88.8688868886889 0.0702249245178443 3.46317025909609E-4
88.8888888888889 0.0702179704154685 3.46214152794825E-4
88.9088908890889 0.070211018513028 3.46111332588612E-4
88.9288928892889 0.0702040688091882 3.4600856524989E-4
88.948894889489 0.070197121302615 3.45905850737614E-4
88.968896889689 0.0701901759919773 3.45803189010808E-4
88.988898889889 0.0701832328759433 3.45700580028513E-4
89.008900890089 0.0701762919531845 3.45598023749843E-4
89.028902890289 0.070169353222372 3.45495520133931E-4
89.0489048904891 0.0701624166821801 3.45393069139982E-4
89.0689068906891 0.0701554823312828 3.45290670727227E-4
89.0889088908891 0.0701485501683567 3.45188324854952E-4
89.1089108910891 0.070141620192079 3.45086031482485E-4
89.1289128912891 0.0701346924011288 3.44983790569203E-4
89.1489148914892 0.0701277667941863 3.44881602074525E-4
89.1689168916892 0.0701208433699324 3.44779465957908E-4
89.1889188918892 0.070113922127051 3.44677382178876E-4
89.2089208920892 0.0701070030642257 3.44575350696974E-4
89.2289228922892 0.0701000861801424 3.44473371471807E-4
89.2489248924893 0.0700931714734886 3.44371444463027E-4
89.2689268926893 0.0700862589429524 3.44269569630319E-4
89.2889288928893 0.0700793485872228 3.44167746933411E-4
89.3089308930893 0.0700724404049918 3.44065976332097E-4
89.3289328932893 0.0700655343949518 3.43964257786199E-4
89.3489348934894 0.0700586305557962 3.43862591255584E-4
89.3689368936894 0.0700517288862201 3.43760976700166E-4
89.3889388938894 0.0700448293849208 3.43659414079916E-4
89.4089408940894 0.0700379320505953 3.43557903354826E-4
89.4289428942894 0.0700310368819433 3.43456444484952E-4
89.4489448944895 0.0700241438776651 3.43355037430386E-4
89.4689468946895 0.070017253036463 3.43253682151268E-4
89.4889488948895 0.0700103643570403 3.43152378607784E-4
89.5089508950895 0.0700034778381012 3.43051126760154E-4
89.5289528952895 0.0699965934783517 3.42949926568653E-4
89.5489548954896 0.0699897112764994 3.42848777993597E-4
89.5689568956896 0.0699828312312528 3.42747680995345E-4
89.5889588958896 0.069975953341322 3.42646635534305E-4
89.6089608960896 0.0699690776054187 3.42545641570929E-4
89.6289628962896 0.0699622040222548 3.42444699065699E-4
89.6489648964897 0.0699553325905443 3.42343807979151E-4
89.6689668966897 0.0699484633090034 3.42242968271875E-4

89.6889688968897 0.0699415961763483 3.42142179904494E-4
89.7089708970897 0.069934731191297 3.42041442837672E-4
89.7289728972897 0.0699278683525685 3.41940757032116E-4
89.7489748974898 0.0699210076588847 3.418401224486E-4
89.7689768976898 0.0699141491089667 3.4173953904791E-4
89.7889788978898 0.0699072927015374 3.41639006790884E-4
89.8089808980898 0.0699004384353232 3.4153852563843E-4
89.8289828982898 0.0698935863090483 3.41438095551456E-4
89.8489848984899 0.0698867363214407 3.41337716490942E-4
89.8689868986899 0.0698798884712291 3.41237388417907E-4
89.8889888988899 0.0698730427571439 3.41137111293418E-4
89.9089908990899 0.0698661991779159 3.41036885078571E-4
89.9289928992899 0.069859357732278 3.40936709734517E-4
89.9489948994899 0.0698525184189647 3.40836585222453E-4
89.96899689969 0.06984568123671 3.40736511503594E-4
89.98899889989 0.0698388461842512 3.40636488539229E-4
90.00900090009 0.0698320132603268 3.40536516290682E-4
90.02900290029 0.0698251824636757 3.40436594719316E-4
90.04900490049 0.0698183537930384 3.40336723786531E-4
90.0690069006901 0.0698115272471563 3.40236903453771E-4
90.0890089008901 0.0698047028247732 3.40137133682537E-4
90.1090109010901 0.069797880524633 3.40037414434351E-4
90.1290129012901 0.0697910603454823 3.39937745670804E-4
90.1490149014901 0.0697842422860683 3.39838127353517E-4
90.1690169016902 0.0697774263451388 3.39738559444144E-4
90.1890189018902 0.0697706125214443 3.39639041904396E-4
90.2090209020902 0.0697638008137352 3.39539574696016E-4
90.2290229022902 0.069756991220764 3.39440157780793E-4
90.2490249024902 0.0697501837412845 3.39340791120563E-4
90.2690269026903 0.0697433783740516 3.39241474677199E-4
90.2890289028903 0.0697365751178218 3.3914220841262E-4
90.3090309030903 0.0697297739713528 3.39042992288789E-4
90.3290329032903 0.0697229749334038 3.38943826267712E-4
90.3490349034903 0.0697161780027337 3.38844710311414E-4
90.3690369036904 0.0697093831781051 3.38745644381995E-4
90.3890389038904 0.0697025904582811 3.3864662844159E-4
90.4090409040904 0.0696957998420251 3.38547662452358E-4
90.4290429042904 0.0696890113281024 3.38448746376512E-4
90.4490449044904 0.0696822249152802 3.38349880176311E-4
90.4690469046905 0.0696754406023257 3.38251063814039E-4
90.4890489048905 0.0696686583880095 3.38152297252056E-4
90.5090509050905 0.069661878271101 3.38053580452724E-4
90.5290529052905 0.0696551002503731 3.37954913378478E-4
90.5490549054906 0.0696483243245982 3.37856295991771E-4

90.5690569056906 0.0696415504925504 3.37757728255101E-4
90.5890589058906 0.0696347787530064 3.37659210131032E-4
90.6090609060906 0.0696280091047426 3.3756074158214E-4
90.6290629062906 0.0696212415465379 3.37462322571065E-4
90.6490649064907 0.069614476077171 3.37363953060461E-4
90.6690669066907 0.069607712695424 3.37265633013062E-4
90.6890689068907 0.0696009514000776 3.37167362391599E-4
90.7090709070907 0.0695941921899166 3.37069141158884E-4
90.7290729072907 0.0695874350637244 3.36970969277738E-4
90.7490749074908 0.0695806800202889 3.36872846711061E-4
90.7690769076908 0.0695739270583951 3.36774773421743E-4
90.7890789078908 0.0695671761768333 3.36676749372765E-4
90.8090809080908 0.0695604273743925 3.36578774527116E-4
90.8290829082908 0.0695536806498644 3.36480848847846E-4
90.8490849084909 0.0695469360020405 3.36382972298026E-4
90.8690869086909 0.0695401934297157 3.36285144840795E-4
90.8890889088909 0.0695334529316835 3.36187366439296E-4
90.9090909090909 0.0695267145067417 3.36089637056757E-4
90.9290929092909 0.0695199781536863 3.35991956656404E-4
90.949094909491 0.0695132438713166 3.35894325201531E-4
90.969096909691 0.0695065116584335 3.35796742655475E-4
90.989098909891 0.0694997815138367 3.35699208981583E-4
91.009100910091 0.0694930534363291 3.35601724143266E-4
91.029102910291 0.0694863274247159 3.35504288103994E-4
91.0491049104911 0.0694796034778008 3.35406900827235E-4
91.0691069106911 0.06947288159439 3.35309562276516E-4
91.0891089108911 0.0694661617732925 3.35212272415428E-4
91.1091109110911 0.0694594440133158 3.3511503120756E-4
91.1291129112911 0.0694527283132704 3.35017838616568E-4
91.1491149114912 0.0694460146719681 3.34920694606148E-4
91.1691169116912 0.0694393030882209 3.34823599140021E-4
91.1891189118912 0.069432593560843 3.34726552181963E-4
91.2091209120912 0.0694258860886497 3.34629553695785E-4
91.2291229122912 0.0694191806704574 3.34532603645337E-4
91.2491249124913 0.0694124773050827 3.34435701994497E-4
91.2691269126913 0.0694057759913461 3.34338848707215E-4
91.2891289128913 0.0693990767280666 3.34242043747447E-4
91.3091309130913 0.0693923795140663 3.34145287079213E-4
91.3291329132913 0.0693856843481673 3.34048578666551E-4
91.3491349134914 0.0693789912291936 3.33951918473556E-4
91.3691369136914 0.0693723001559708 3.3385530646436E-4
91.3891389138914 0.0693656111273251 3.33758742603131E-4
91.4091409140914 0.0693589241420829 3.33662226854058E-4
91.4291429142914 0.0693522391990754 3.33565759181422E-4

91.4491449144915 0.0693455562971304 3.33469339549478E-4
91.4691469146915 0.069338875435081 3.33372967922575E-4
91.4891489148915 0.0693321966117592 3.33276644265072E-4
91.5091509150915 0.0693255198259985 3.33180368541369E-4
91.5291529152915 0.069318845076634 3.33084140715909E-4
91.5491549154916 0.0693121723625023 3.32987960753182E-4
91.5691569156916 0.0693055016824408 3.32891828617704E-4
91.5891589158916 0.0692988330352886 3.32795744274047E-4
91.6091609160916 0.069292166419885 3.32699707686799E-4
91.6291629162916 0.0692855018350722 3.32603718820612E-4
91.6491649164917 0.0692788392796916 3.32507777640151E-4
91.6691669166917 0.0692721787525875 3.32411884110142E-4
91.6891689168917 0.0692655202526048 3.3231603819534E-4
91.7091709170917 0.0692588637785897 3.32220239860541E-4
91.7291729172917 0.0692522093293893 3.32124489070575E-4
91.7491749174918 0.0692455569038528 3.32028785790325E-4
91.7691769176918 0.069238906500829 3.31933129984684E-4
91.7891789178918 0.0692322581191698 3.31837521618617E-4
91.8091809180918 0.0692256117577273 3.31741960657109E-4
91.8291829182918 0.069218967415355 3.31646447065187E-4
91.8491849184919 0.0692123250909074 3.31550980807914E-4
91.8691869186919 0.0692056847832411 3.31455561850403E-4
91.8891889188919 0.0691990464912121 3.31360190157778E-4
91.9091909190919 0.06919241021368 3.3126486569524E-4
91.9291929192919 0.0691857759495031 3.3116958842799E-4
91.949194919492 0.0691791436975432 3.31074358321297E-4
91.969196919692 0.0691725134566622 3.30979175340456E-4
91.989198919892 0.0691658852257229 3.30884039450794E-4
92.009200920092 0.0691592590035899 3.30788950617685E-4
92.029202920292 0.0691526347891297 3.30693908806553E-4
92.0492049204921 0.0691460125812079 3.30598913982824E-4
92.0692069206921 0.0691393923786933 3.30503966111997E-4
92.0892089208921 0.0691327741804545 3.30409065159585E-4
92.1092109210921 0.0691261579853627 3.3031421109116E-4
92.1292129212921 0.0691195437922891 3.30219403872315E-4
92.1492149214922 0.0691129316001071 3.30124643468693E-4
92.1692169216922 0.0691063214076905 3.30029929845965E-4
92.1892189218922 0.0690997132139141 3.29935262969837E-4
92.2092209220922 0.0690931070176554 3.29840642806073E-4
92.2292229222922 0.0690865028177908 3.29746069320443E-4
92.2492249224923 0.0690799006132008 3.29651542478794E-4
92.2692269226923 0.0690733004027637 3.29557062246965E-4
92.2892289228923 0.0690667021853626 3.29462628590881E-4
92.3092309230923 0.0690601059598788 3.29368241476462E-4

92.3292329232923 0.0690535117251966 3.29273900869694E-4
92.3492349234924 0.0690469194802001 3.29179606736576E-4
92.3692369236924 0.0690403292237757 3.29085359043164E-4
92.3892389238924 0.0690337409548106 3.28991157755543E-4
92.4092409240924 0.069027154672194 4 3.28897002839853E-4
92.4292429242924 0.0690205703748146 3.28802894262227E-4
92.4492449244924 0.0690139880615643 3.28708831988894E-4
92.4692469246925 0.0690074077313329 3.28614815986052E-4
92.4892489248925 0.0690008293830157 3.28520846220005E-4
92.5092509250925 0.0689942530155066 3.28426922657052E-4
92.5292529252925 0.0689876786277011 3.2833304526354E-4
92.5492549254925 0.0689811062184948 3.28239214005838E-4
92.5692569256926 0.0689745357867877 3.28145428850392E-4
92.5892589258926 0.068967967331477 3.28051689763631E-4
92.6092609260926 0.0689614008514643 3.27957996712069E-4
92.6292629262926 0.06895483634565 3.2786434966222E-4
92.6492649264926 0.0689482738129378 3.27770748580667E-4
92.6692669266927 0.0689417132522303 3.27677193433994E-4
92.6892689268927 0.0689351546624341 3.27583684188863E-4
92.7092709270927 0.0689285980424538 3.27490220811928E-4
92.7292729272927 0.0689220433911969 3.27396803269907E-4
92.7492749274927 0.0689154907075731 3.27303431529562E-4
92.7692769276928 0.0689089399904911 3.27210105557665E-4
92.7892789278928 0.0689023912388616 3.27116825321037E-4
92.8092809280928 0.0688958444515974 3.27023590786548E-4
92.8292829282928 0.0688892996276109 3.26930401921077E-4
92.8492849284929 0.0688827567658166 3.2683725869156E-4
92.8692869286929 0.0688762158651302 3.26744161064964E-4
92.8892889288929 0.0688696769244688 3.26651109008299E-4
92.9092909290929 0.0688631399427493 3.26558102488588E-4
92.9292929292929 0.0688566049188911 3.26465141472912E-4
92.949294929493 0.0688500718518148 3.2637222592839E-4
92.969296929693 0.0688435407404406 3.26279355822148E-4
92.989298929893 0.0688370115836924 3.26186531121392E-4
93.009300930093 0.0688304843804925 3.26093751793319E-4
93.029302930293 0.0688239591297667 3.26001017805197E-4
93.0493049304931 0.068817435830441 3.25908329124317E-4
93.0693069306931 0.0688109144814411 3.25815685717985E-4
93.0893089308931 0.0688043950816964 3.25723087553576E-4
93.1093109310931 0.0687978776301362 3.25630534598485E-4
93.1293129312931 0.0687913621256909 3.25538026820143E-4
93.1493149314932 0.0687848485672922 3.25445564186017E-4
93.1693169316932 0.0687783369538731 3.25353146663612E-4
93.1893189318932 0.068771827284367 3.25260774220455E-4

93.2093209320932 0.06876531955771 3.25168446824136E-4
93.2293229322932 0.068758813772838 3.25076164442258E-4
93.2493249324933 0.0687523099286877 3.24983927042454E-4
93.2693269326933 0.0687458080241986 3.24891734592417E-4
93.2893289328933 0.0687393080583103 3.24799587059858E-4
93.3093309330933 0.0687328100299628 3.24707484412517E-4
93.3293329332933 0.0687263139380989 3.24615426618188E-4
93.3493349334934 0.0687198197816622 3.245234136447E-4
93.3693369336934 0.0687133275595958 3.24431445459888E-4
93.3893389338934 0.0687068372708462 3.24339522031659E-4
93.4093409340934 0.0687003489143588 3.24247643327922E-4
93.4293429342934 0.0686938624890824 3.2415580931665E-4
93.4493449344935 0.0686873779939652 3.2406401996583E-4
93.4693469346935 0.0686808954279572 3.23972275243494E-4
93.4893489348935 0.0686744147900095 3.23880575117704E-4
93.5093509350935 0.0686679360790744 3.23788919556559E-4
93.5293529352935 0.0686614592941053 3.23697308528196E-4
93.5493549354936 0.0686549844340559 3.23605742000771E-4
93.5693569356936 0.0686485114978835 3.2351421994251E-4
93.5893589358936 0.0686420404845429 3.23422742321623E-4
93.6093609360936 0.0686355713929932 3.23331309106399E-4
93.6293629362936 0.0686291042221929 3.23239920265138E-4
93.6493649364937 0.0686226389711021 3.23148575766182E-4
93.6693669366937 0.0686161756386818 3.230572755779E-4
93.6893689368937 0.0686097142238951 3.22966019668714E-4
93.7093709370937 0.0686032547257051 3.22874808007061E-4
93.7293729372937 0.068596797143076 3.22783640561413E-4
93.7493749374938 0.0685903414749733 3.22692517300282E-4
93.7693769376938 0.0685838877203647 3.22601438192223E-4
93.7893789378938 0.0685774358782175 3.2251040320581E-4
93.8093809380938 0.068570985947501 3.22419412309657E-4
93.8293829382938 0.0685645379271852 3.22328465472415E-4
93.8493849384939 0.0685580918162414 3.22237562662764E-4
93.8693869386939 0.0685516476136421 3.22146703849422E-4
93.8893889388939 0.0685452053183597 3.22055889001124E-4
93.9093909390939 0.0685387649293708 3.21965118086682E-4
93.9293929392939 0.0685323264456498 3.21874391074895E-4
93.949394939494 0.0685258898661735 3.21783707934614E-4
93.969396939694 0.0685194551899201 3.21693068634729E-4
93.989398939894 0.0685130224158687 3.21602473144159E-4
94.009400940094 0.0685065915429991 3.21511921431853E-4
94.029402940294 0.0685001625702926 3.21421413466797E-4
94.0494049404941 0.068493735496731 3.21330949218005E-4
94.0694069406941 0.0684873103212994 3.21240528654551E-4

94.0894089408941 0.0684808870429802 3.21150151745493E-4
94.1094109410941 0.0684744656607612 3.21059818459982E-4
94.1294129412941 0.0684680461736274 3.20969528767145E-4
94.1494149414942 0.068461628580567 3.20879282636177E-4
94.1694169416942 0.0684552128805686 3.20789080036293E-4
94.1894189418942 0.0684487990726234 3.20698920936762E-4
94.2094209420942 0.0684423871557216 3.20608805306857E-4
94.2294229422942 0.0684359771288554 3.205187331159E-4
94.2494249424943 0.0684295689910187 3.20428704333252E-4
94.2694269426943 0.0684231627412046 3.20338718928279E-4
94.2894289428943 0.06841675837841 3.20248776870423E-4
94.3094309430943 0.0684103559016303 3.20158878129118E-4
94.3294329432943 0.0684039553098634 3.20069022673852E-4
94.3494349434944 0.0683975566021087 3.19979210474155E-4
94.3694369436944 0.0683911597773654 3.19889441499565E-4
94.3894389438944 0.068384764834634 3.19799715719665E-4
94.4094409440944 0.0683783717729169 3.19710033104076E-4
94.4294429442944 0.0683719805912174 3.19620393622448E-4
94.4494449444945 0.0683655912885392 3.19530797244459E-4
94.4694469446945 0.0683592038638876 3.19441243939825E-4
94.4894489448945 0.0683528183162692 3.19351733678296E-4
94.5094509450945 0.0683464346446905 3.19262266429641E-4
94.5294529452945 0.0683400528481611 3.19172842163687E-4
94.5494549454946 0.0683336729256899 3.19083460850273E-4
94.5694569456946 0.0683272948762872 3.18994122459269E-4
94.5894589458946 0.0683209186989644 3.18904826960584E-4
94.6094609460946 0.0683145443927355 3.18815574324176E-4
94.6294629462946 0.0683081719566127 3.18726364519997E-4
94.6494649464947 0.0683018013896125 3.18637197518076E-4
94.6694669466947 0.0682954326907499 3.18548073288439E-4
94.6894689468947 0.068289065859041 3.18458991801148E-4
94.7094709470947 0.0682827008935051 3.18369953026319E-4
94.7294729472947 0.0682763377931618 3.18280956934095E-4
94.7494749474948 0.0682699765570301 3.18192003494629E-4
94.7694769476948 0.0682636171841313 3.18103092678121E-4
94.7894789478948 0.0682572596734883 3.18014224454808E-4
94.8094809480948 0.0682509040241244 3.17925398794954E-4
94.8294829482948 0.0682445502350633 3.17836615668844E-4
94.8494849484948 0.0682381983053313 3.17747875046818E-4
94.8694869486949 0.0682318482339544 3.17659176899225E-4
94.8894889488949 0.0682255000199612 3.17570521196472E-4
94.9094909490949 0.0682191536623789 3.17481907908962E-4
94.9294929492949 0.0682128091602384 3.17393337007164E-4
94.9494949494949 0.0682064665125694 3.17304808461549E-4

94.969496949695 0.0682001257184045 3.17216322242649E-4
94.989498949895 0.0681937867767768 3.17127878321014E-4
95.009500950095 0.0681874496867197 3.17039476667218E-4
95.029502950295 0.068181114447267 3.16951117251858E-4
95.049504950495 0.0681747810574569 3.16862800045609E-4
95.0695069506951 0.0681684495163251 3.16774525019125E-4
95.0895089508951 0.0681621198229105 3.16686292143127E-4
95.1095109510951 0.0681557919762515 3.16598101388341E-4
95.1295129512951 0.0681494659753884 3.1650995272554E-4
95.1495149514951 0.0681431418193628 3.16421846125528E-4
95.1695169516952 0.0681368195072167 3.16333781559134E-4
95.1895189518952 0.0681304990379933 3.16245758997221E-4
95.2095209520952 0.068124180410738 3.16157778410695E-4
95.2295229522952 0.0681178636244941 3.16069839770456E-4
95.2495249524953 0.0681115486783098 3.15981943047482E-4
95.2695269526953 0.0681052355712316 3.15894088212752E-4
95.2895289528953 0.0680989243023087 3.15806275237289E-4
95.3095309530953 0.0680926148705896 3.15718504092132E-4
95.3295329532953 0.0680863072751267 3.15630774748387E-4
95.3495349534954 0.0680800015149693 3.15543087177133E-4
95.3695369536954 0.068073697589172 3.15455441349539E-4
95.3895389538954 0.068067395496787 3.1536783723676E-4
95.4095409540954 0.0680610952368697 3.15280274810008E-4
95.4295429542954 0.0680547968084763 3.15192754040526E-4
95.4495449544955 0.0680485002106619 3.15105274899556E-4
95.4695469546955 0.0680422054424853 3.15017837358409E-4
95.4895489548955 0.0680359125030053 3.14930441388412E-4
95.5095509550955 0.0680296213912816 3.1484308696092E-4
95.5295529552955 0.0680233321063751 3.14755774047323E-4
95.5495549554956 0.0680170446473481 3.14668502619045E-4
95.5695569556956 0.0680107590132624 3.1458127264752E-4
95.5895589558956 0.0680044752031828 3.1449408410424E-4
95.6095609560956 0.0679981932161731 3.14406936960699E-4
95.6295629562956 0.0679919130513002 3.14319831188451E-4
95.6495649564957 0.0679856347076309 3.14232766759063E-4
95.6695669566957 0.067979358184233 3.14145743644135E-4
95.6895689568957 0.0679730834801746 3.14058761815285E-4
95.7095709570957 0.0679668105945274 3.13971821244199E-4
95.7295729572957 0.0679605395263613 3.1388492190255E-4
95.7495749574958 0.0679542702747476 3.13798063762054E-4
95.7695769576958 0.0679480028387617 3.13711246794492E-4
95.7895789578958 0.0679417372174751 3.13624470971611E-4
95.8095809580958 0.0679354734099633 3.1353773626523E-4
95.8295829582958 0.0679292114153039 3.13451042647212E-4

95.8495849584959 0.0679229512325716 3.13364390089395E-4
95.8695869586959 0.0679166928608468 3.13277778563713E-4
95.8895889588959 0.0679104362992064 3.13191208042068E-4
95.9095909590959 0.0679041815467319 3.13104678496441E-4
95.9295929592959 0.0678979286025036 3.13018189898811E-4
95.949594959496 0.067891677465604 3.12931742221204E-4
95.969596959696 0.0678854281351153 3.12845335435662E-4
95.989598959896 0.0678791806101225 3.12758969514273E-4
96.009600960096 0.0678729348897098 3.12672644429139E-4
96.029602960296 0.0678666909729638 3.12586360152404E-4
96.0496049604961 0.0678604488589715 3.12500116656237E-4
96.0696069606961 0.0678542085468205 3.1241391391283E-4
96.0896089608961 0.0678479700355998 3.12327751894411E-4
96.1096109610961 0.0678417333243994 3.12241630573235E-4
96.1296129612961 0.0678354984123112 3.12155549921601E-4
96.1496149614962 0.0678292652984264 3.12069509911814E-4
96.1696169616962 0.0678230339818371 3.11983510516207E-4
96.1896189618962 0.0678168044616389 3.11897551707178E-4
96.2096209620962 0.067810576736925 3.11811633457104E-4
96.2296229622962 0.0678043508067931 3.11725755738443E-4
96.2496249624963 0.0677981266703385 3.11639918523634E-4
96.2696269626963 0.0677919043266607 3.11554121785192E-4
96.2896289628963 0.0677856837748564 3.11468365495604E-4
96.3096309630963 0.0677794650140272 3.11382649627444E-4
96.3296329632963 0.067773248043274 3.11296974153291E-4
96.3496349634964 0.0677670328616963 3.11211339045722E-4
96.3696369636964 0.0677608194683994 3.11125744277403E-4
96.3896389638964 0.0677546078624862 3.11040189820992E-4
96.4096409640964 0.0677483980430613 3.10954675649177E-4
96.4296429642964 0.0677421900092303 3.1086920173468E-4
96.4496449644965 0.0677359837601 3.10783768050253E-4
96.4696469646965 0.067729779294778 3.10698374568677E-4
96.4896489648965 0.0677235766123731 3.10613021262762E-4
96.5096509650965 0.0677173757119947 3.10527708105341E-4
96.5296529652965 0.0677111765927529 3.10442435069276E-4
96.5496549654966 0.0677049792537594 3.10357202127464E-4
96.5696569656966 0.0676987836941271 3.10272009252831E-4
96.5896589658966 0.0676925899129698 3.10186856418337E-4
96.6096609660966 0.067686397909401 3.10101743596945E-4
96.6296629662966 0.0676802076825363 3.10016670761669E-4
96.6496649664967 0.0676740192314919 3.09931637885544E-4
96.6696669666967 0.0676678325553853 3.09846644941638E-4
96.6896689668967 0.0676616476533351 3.09761691903047E-4
96.7096709670967 0.0676554645244597 3.09676778742884E-4

96.7296729672967 0.0676492831678801 3.09591905434309E-4
96.7496749674968 0.0676431035827167 3.09507071950491E-4
96.7696769676968 0.0676369257680924 3.09422278264647E-4
96.7896789678968 0.0676307497231294 3.09337524350001E-4
96.8096809680968 0.0676245754469521 3.0925281017982E-4
96.8296829682968 0.0676184029386848 3.09168135727387E-4
96.8496849684969 0.0676122321974542 3.09083500966031E-4
96.8696869686969 0.0676060632223867 3.08998905869093E-4
96.8896889688969 0.0675998960126108 3.08914350409959E-4
96.9096909690969 0.0675937305672546 3.08829834562019E-4
96.9296929692969 0.0675875668854474 3.08745358298703E-4
96.949694969497 0.0675814049663204 3.08660921593474E-4
96.969696969697 0.067575244809005 3.08576524419816E-4
96.989698969897 0.0675690864126341 3.0849216675125E-4
97.009700970097 0.0675629297763405 3.08407848561308E-4
97.029702970297 0.0675567748992589 3.08323569823563E-4
97.0497049704971 0.0675506217805243 3.08239330511608E-4
97.0697069706971 0.0675444704192734 3.08155130599073E-4
97.0897089708971 0.0675383208146436 3.08070970059613E-4
97.1097109710971 0.0675321729657734 3.07986848866911E-4
97.1297129712971 0.0675260268718001 3.07902766994655E-4
97.1497149714972 0.0675198825318654 3.07818724416597E-4
97.1697169716972 0.0675137399451097 3.07734721106495E-4
97.1897189718972 0.0675075991106749 3.07650757038139E-4
97.2097209720972 0.0675014600277042 3.07566832185351E-4
97.2297229722972 0.0674953226953408 3.07482946521965E-4
97.2497249724972 0.0674891871127292 3.07399100021856E-4
97.2697269726973 0.067483053279016 3.07315292658932E-4
97.2897289728973 0.0674769211933473 3.07231524407115E-4
97.3097309730973 0.0674707908548694 3.07147795240343E-4
97.3297329732973 0.067464662262733 3.07064105132628E-4
97.3497349734973 0.0674585354160851 3.06980454057945E-4
97.3697369736974 0.0674524103140776 3.06896841990355E-4
97.3897389738974 0.0674462869558608 3.06813268903906E-4
97.4097409740974 0.0674401653405868 3.06729734772685E-4
97.4297429742974 0.0674340454674097 3.06646239570829E-4
97.4497449744974 0.0674279273354818 3.06562783272454E-4
97.4697469746975 0.0674218109439583 3.06479365851737E-4
97.4897489748975 0.0674156962919964 3.06395987282894E-4
97.5097509750975 0.067409583378752 3.06312647540138E-4
97.5297529752975 0.0674034722033809 3.06229346597697E-4
97.5497549754975 0.0673973627650441 3.0614608442988E-4
97.5697569756976 0.0673912550629002 3.0606286101098E-4
97.5897589758976 0.0673851490961092 3.05979676315325E-4

97.6097609760976 0.0673790448638331 3.05896530317283E-4
97.6297629762976 0.0673729423652328 3.0581342299122E-4
97.6497649764977 0.0673668415994721 3.05730354311556E-4
97.6697669766977 0.067360742565715 3.05647324252731E-4
97.6897689768977 0.0673546452631264 3.0556433278921E-4
97.7097709770977 0.0673485496908721 3.0548137989548E-4
97.7297729772977 0.0673424558481186 3.05398465546057E-4
97.7497749774978 0.0673363637340332 3.05315589715483E-4
97.7697769776978 0.0673302733477849 3.05232752378332E-4
97.7897789778978 0.0673241846885441 3.05149953509213E-4
97.8097809780978 0.0673180977554785 3.05067193082716E-4
97.8297829782978 0.0673120125477613 3.04984471073517E-4
97.8497849784979 0.0673059290645639 3.04901787456278E-4
97.8697869786979 0.0672998473050595 3.04819142205706E-4
97.8897889788979 0.0672937672684222 3.04736535296532E-4
97.9097909790979 0.067287688953826 3.04653966703498E-4
97.9297929792979 0.0672816123604473 3.04571436401391E-4
97.949794979498 0.0672755374874627 3.04488944365022E-4
97.969796979698 0.0672694643340489 3.04406490569211E-4
97.989798979898 0.067263392899385 3.04324074988824E-4
98.009800980098 0.0672573231826505 3.04241697598751E-4
98.029802980298 0.0672512551830244 3.04159358373886E-4
98.0498049804981 0.0672451888996882 3.04077057289171E-4
98.0698069806981 0.0672391243318242 3.03994794319572E-4
98.0898089808981 0.0672330614786143 3.03912569440067E-4
98.1098109810981 0.0672270003392437 3.03830382625688E-4
98.1298129812981 0.0672209409128954 3.03748233851453E-4
98.1498149814982 0.0672148831987561 3.03666123092445E-4
98.1698169816982 0.0672088271960113 3.03584050323743E-4
98.1898189818982 0.0672027729038479 3.03502015520461E-4
98.2098209820982 0.0671967203214553 3.03420018657755E-4
98.2298229822982 0.0671906694480215 3.03338059710783E-4
98.2498249824983 0.0671846202827362 3.03256138654734E-4
98.2698269826983 0.0671785728247903 3.03174255464831E-4
98.2898289828983 0.0671725270733759 3.03092410116325E-4
98.3098309830983 0.0671664830276845 3.03010602584471E-4
98.3298329832983 0.0671604406869107 3.02928832844582E-4
98.3498349834984 0.0671544000502472 3.02847100871958E-4
98.3698369836984 0.0671483611168911 3.02765406641974E-4
98.3898389838984 0.0671423238860358 3.02683750129965E-4
98.4098409840984 0.06713628835688 3.02602131311354E-4
98.4298429842984 0.0671302545286209 3.02520550161555E-4
98.4498449844985 0.0671242224004571 3.02439006656017E-4
98.4698469846985 0.0671181919715874 3.02357500770203E-4

98.4898489848985 0.0671121632412129 3.02276032479623E-4
98.5098509850985 0.0671061362085344 3.02194601759793E-4
98.5298529852985 0.0671001108727538 3.02113208586261E-4
98.5498549854986 0.0670940872330743 3.02031852934603E-4
98.5698569856986 0.0670880652886996 3.01950534780417E-4
98.5898589858986 0.067082045038836 3.01869254099317E-4
98.6098609860986 0.0670760264826822 3.01788010866957E-4
98.6298629862986 0.067070009619452 3.01706805059012E-4
98.6498649864987 0.0670639944483498 3.01625636651176E-4
98.6698669866987 0.0670579809685839 3.01544505619176E-4
98.6898689868987 0.0670519691793634 3.01463411938762E-4
98.7098709870987 0.0670459590798968 3.01382355585689E-4
98.7298729872987 0.067039950669396 3.01301336535773E-4
98.7498749874988 0.0670339439470725 3.01220354764836E-4
98.7698769876988 0.0670279389121371 3.01139410248705E-4
98.7898789878988 0.0670219355638041 3.01058502963266E-4
98.8098809880988 0.0670159339012876 3.00977632884417E-4
98.8298829882988 0.0670099339238017 3.00896799988068E-4
98.8498849884989 0.0670039356305619 3.00816004250165E-4
98.8698869886989 0.0669979390207855 3.00735245646684E-4
98.8898889888989 0.0669919440936899 3.00654524153623E-4
98.9098909890989 0.0669859508484927 3.00573839746989E-4
98.9298929892989 0.0669799592844131 3.00493192402828E-4
98.949894989499 0.0669739694006711 3.00412582097211E-4
98.969896989699 0.0669679811964874 3.0033200880623E-4
98.989898989899 0.0669619946710836 3.00251472505997E-4
99.009900990099 0.0669560098236827 3.00170973172666E-4
99.029902990299 0.0669500266535068 3.00090510782383E-4
99.0499049904991 0.0669440451597796 3.00010085311333E-4
99.0699069906991 0.0669380653417282 2.99929696735759E-4
99.0899089908991 0.0669320871985757 2.99849345031863E-4
99.1099109910991 0.0669261107295501 2.99769030175925E-4
99.1299129912991 0.06692013593388 2.99688752144247E-4
99.1499149914992 0.0669141628107913 2.99608510913109E-4
99.1699169916992 0.066908191359514 2.99528306458856E-4
99.1899189918992 0.0669022215792784 2.99448138757852E-4
99.2099209920992 0.0668962534693137 2.99368007786457E-4
99.2299229922992 0.0668902870288538 2.9928791352111E-4
99.2499249924993 0.0668843222571292 2.99207855938215E-4
99.2699269926993 0.0668783591533736 2.99127835014233E-4
99.2899289928993 0.0668723977168217 2.99047850725651E-4
99.3099309930993 0.066866437946708 2.98967903048965E-4
99.3299329932993 0.0668604798422665 2.98887991960678E-4
99.3499349934994 0.0668545234027358 2.98808117437368E-4

99.3699369936994 0.0668485686273527 2.98728279455601E-4
99.3899389938994 0.0668426155153552 2.98648477991972E-4
99.4099409940994 0.0668366640659821 2.98568713023098E-4
99.4299429942994 0.0668307142784735 2.98488984525629E-4
99.4499449944995 0.0668247661520689 2.98409292476217E-4
99.4699469946995 0.0668188196860099 2.98329636851557E-4
99.4899489948995 0.0668128748795399 2.98250017628378E-4
99.5099509950995 0.0668069317319012 2.9817043478341E-4
99.5299529952995 0.0668009902423371 2.98090888293408E-4
99.5499549954996 0.0667950504100933 2.9801137813517E-4
99.5699569956996 0.066789112234414 2.97931904285489E-4
99.5899589958996 0.0667831757145463 2.97852466721208E-4
99.6099609960996 0.0667772408497368 2.97773065419176E-4
99.6299629962996 0.066771307639233 2.97693700356266E-4
99.6499649964996 0.0667653760822844 2.97614371509392E-4
99.6699669966997 0.0667594461781405 2.97535078855481E-4
99.6899689968997 0.0667535179260507 2.97455822371466E-4
99.7099709970997 0.0667475913252665 2.97376602034328E-4
99.7299729972997 0.0667416663750401 2.97297417821064E-4
99.7499749974997 0.0667357430746234 2.97218269708681E-4
99.7699769976998 0.0667298214232702 2.97139157674228E-4
99.7899789978998 0.066723901420235 2.97060081694768E-4
99.8099809980998 0.0667179830647725 2.96981041747385E-4
99.8299829982998 0.0667120663561392 2.96902037809199E-4
99.8499849984998 0.0667061512935902 2.9682306985732E-4
99.8699869986999 0.0667002378763852 2.96744137868937E-4
99.8899889988999 0.0666943261037801 2.96665241821195E-4
99.9099909990999 0.0666884159750349 2.9658638169131E-4
99.9299929992999 0.0666825074894101 2.96507557456516E-4
99.9499949995 0.0666766006461651 2.96428769094046E-4
99.9699969997 0.0666706954445622 2.96350016581179E-4
99.9899989999 0.0666647918838625 2.96271299895196E-4
100.0100010001 0.06665888996333 2.96192619013427E-4
100.0300030003 0.0666529896822279 2.96113973913202E-4
100.0500050005 0.0666470910398211 2.96035364571887E-4
100.0700070007 0.0666411940353746 2.95956790966863E-4
100.0900090009 0.0666352986681544 2.95878253075534E-4
100.1100110011 0.066629404937427 2.95799750875326E-4
100.1300130013 0.0666235128424612 2.95721284343703E-4
100.1500150015 0.0666176223825249 2.95642853458132E-4
100.1700170017 0.0666117335568863 2.95564458196099E-4
100.1900190019 0.0666058463648171 2.95486098535144E-4
100.2100210021 0.0665999608055863 2.95407774452787E-4
100.2300230023 0.0665940768784665 2.95329485926602E-4

100.2500250025 0.0665881945827293 2.95251232934168E-4
100.2700270027 0.0665823139176485 2.95173015453105E-4
100.2900290029 0.0665764348824987 2.95094833461052E-4
100.3100310031 0.0665705574765527 2.95016686935636E-4
100.3300330033 0.0665646816990864 2.94938575854543E-4
100.3500350035 0.0665588075493761 2.9486050019547E-4
100.3700370037 0.0665529350266991 2.94782459936142E-4
100.3900390039 0.0665470641303325 2.94704455054294E-4
100.4100410041 0.0665411948595552 2.94626485527695E-4
100.4300430043 0.0665353272136464 2.94548551334131E-4
100.4500450045 0.0665294611918856 2.94470652451405E-4
100.4700470047 0.0665235967935535 2.94392788857344E-4
100.490049004901 0.0665177340179329 2.94314960529819E-4
100.510051005101 0.0665118728643041 2.94237167446679E-4
100.530053005301 0.0665060133319521 2.94159409585846E-4
100.550055005501 0.066500155420159 2.94081686925213E-4
100.570057005701 0.0664942991282096 2.94003999442727E-4
100.590059005901 0.0664884444553899 2.93926347116358E-4
100.610061006101 0.0664825914009853 2.93848729924081E-4
100.630063006301 0.0664767399642834 2.93771147843911E-4
100.650065006501 0.0664708901445725 2.93693600853884E-4
100.670067006701 0.0664650419411371 2.93616088932003E-4
100.690069006901 0.0664591953532698 2.93538612056381E-4
100.710071007101 0.06645335038026 2.93461170205106E-4
100.730073007301 0.066447507021397 2.93383763356277E-4
100.750075007501 0.066441665275973 2.9330639148804E-4
100.770077007701 0.0664358251432793 2.93229054578543E-4
100.790079007901 0.0664299866226097 2.93151752605977E-4
100.810081008101 0.0664241497132572 2.93074485548535E-4
100.830083008301 0.0664183144145154 2.92997253384431E-4
100.850085008501 0.0664124807256815 2.92920056091934E-4
100.870087008701 0.0664066486460479 2.92842893649267E-4
100.890089008901 0.0664008181749136 2.92765766034748E-4
100.910091009101 0.0663949893115755 2.9268867322668E-4
100.930093009301 0.0663891620553313 2.92611615203388E-4
100.950095009501 0.0663833364054792 2.92534591943214E-4
100.970097009701 0.066377512361319 2.92457603424535E-4
100.990099009901 0.0663716899221524 2.92380649625764E-4
101.010101010101 0.0663658690872777 2.92303730525277E-4
101.030103010301 0.0663600498559982 2.92226846101535E-4
101.050105010501 0.0663542322276159 2.92149996332985E-4
101.070107010701 0.0663484162014337 2.92073181198105E-4
101.090109010901 0.0663426017767558 2.91996400675396E-4
101.110111011101 0.0663367889528871 2.91919654743381E-4

101.130113011301 0.0663309777291319 2.91842943380588E-4
101.150115011501 0.0663251681047968 2.91766266565588E-4
101.170117011701 0.0663193600791879 2.91689624276952E-4
101.190119011901 0.0663135536516143 2.91613016493306E-4
101.210121012101 0.0663077488213835 2.91536443193265E-4
101.230123012301 0.0663019455878032 2.91459904355458E-4
101.250125012501 0.0662961439501852 2.91383399958579E-4
101.270127012701 0.0662903439078381 2.91306929981293E-4
101.290129012901 0.0662845454600744 2.91230494402326E-4
101.310131013101 0.066278748606204 2.91154093200381E-4
101.330133013301 0.0662729533455411 2.91077726354229E-4
101.350135013501 0.0662671596773989 2.91001393842641E-4
101.370137013701 0.0662613676010904 2.90925095644394E-4
101.390139013901 0.066255577115931 2.90848831738311E-4
101.410141014101 0.0662497882212358 2.90772602103217E-4
101.430143014301 0.0662440009163214 2.90696406717975E-4
101.450145014501 0.0662382152005038 2.90620245561445E-4
101.470147014701 0.0662324310731018 2.90544118612539E-4
101.490149014901 0.0662266485334326 2.90468025850158E-4
101.510151015102 0.0662208675808151 2.90391967253236E-4
101.530153015302 0.0662150882145696 2.90315942800737E-4
101.550155015502 0.0662093104340156 2.90239952471628E-4
101.570157015702 0.0662035342384754 2.90163996244918E-4
101.590159015902 0.066197759627272 2.90088074099615E-4
101.610161016102 0.0661919865997214 2.90012186014751E-4
101.630163016302 0.0661862151551548 2.89936331969409E-4
101.650165016502 0.0661804452928921 2.89860511942641E-4
101.670167016702 0.0661746770122584 2.89784725913554E-4
101.690169016902 0.0661689103125794 2.89708973861268E-4
101.710171017102 0.0661631451931809 2.89633255764922E-4
101.730173017302 0.0661573816533893 2.8955757160367E-4
101.750175017502 0.0661516196925313 2.89481921356683E-4
101.770177017702 0.0661458593099378 2.89406305003197E-4
101.790179017902 0.0661401005049344 2.89330722522391E-4
101.810181018102 0.0661343432768522 2.89255173893522E-4
101.830183018302 0.0661285876250214 2.89179659095854E-4
101.850185018502 0.066122833548772 2.89104178108654E-4
101.870187018702 0.0661170810474362 2.8902873091123E-4
101.890189018902 0.0661113301203464 2.88953317482904E-4
101.910191019102 0.0661055807668347 2.88877937803005E-4
101.930193019302 0.066099832986236 2.88802591850908E-4
101.950195019502 0.0660940867778832 2.88727279605975E-4
101.970197019702 0.0660883421411127 2.88652001047624E-4
101.990199019902 0.0660825990752595 2.88576756155264E-4

102.010201020102 0.0660768575796594 2.88501544908329E-4
102.030203020302 0.0660711176536509 2.88426367286293E-4
102.050205020502 0.06606537929657 2.88351223268617E-4
102.070207020702 0.0660596425077555 2.882761128348E-4
102.090209020902 0.0660539072865479 2.8820103596438E-4
102.110211021102 0.0660481736322863 2.88125992636885E-4
102.130213021302 0.0660424415443102 2.88050982831862E-4
102.150215021502 0.0660367110219616 2.87976006528897E-4
102.170217021702 0.0660309820645819 2.87901063707585E-4
102.190219021902 0.0660252546715146 2.8782615434755E-4
102.210221022102 0.0660195288421003 2.87751278428397E-4
102.230223022302 0.0660138045756868 2.87676435929835E-4
102.250225022502 0.0660080818716146 2.87601626831484E-4
102.270227022702 0.0660023607292321 2.8752685111308E-4
102.290229022902 0.0659966411478826 2.87452108754306E-4
102.310231023102 0.0659909231269146 2.87377399734918E-4
102.330233023302 0.0659852066656743 2.87302724034656E-4
102.350235023502 0.0659794917635093 2.87228081633291E-4
102.370237023702 0.0659737784197686 2.87153472510618E-4
102.390239023902 0.0659680666338008 2.8707889664644E-4
102.410241024102 0.0659623564049567 2.870043540206E-4
102.430243024302 0.0659566477325864 2.86929844612942E-4
102.450245024502 0.0659509406160405 2.86855368403324E-4
102.470247024702 0.0659452350546718 2.8678092537165E-4
102.490249024903 0.0659395310478322 2.86706515497814E-4
102.510251025103 0.0659338285948756 2.86632138761754E-4
102.530253025303 0.0659281276951544 2.86557795143397E-4
102.550255025503 0.0659224283480244 2.86483484622722E-4
102.570257025703 0.0659167305528392 2.86409207179694E-4
102.590259025903 0.0659110343089558 2.86334962794328E-4
102.610261026103 0.0659053396157309 2.86260751446652E-4
102.630263026303 0.0658996464725213 2.861865731167E-4
102.650265026503 0.0658939548786852 2.86112427784535E-4
102.670267026703 0.0658882648335786 2.86038315430208E-4
102.690269026903 0.0658825763365624 2.85964236033837E-4
102.710271027103 0.0658768893869967 2.85890189575541E-4
102.730273027303 0.0658712039842417 2.85816176035456E-4
102.750275027503 0.0658655201276575 2.85742195393723E-4
102.770277027703 0.065859837816607 2.85668247630528E-4
102.790279027903 0.0658541570504506 2.85594332726039E-4
102.810281028103 0.0658484778285525 2.85520450660482E-4
102.830283028303 0.065842800150277 2.85446601414094E-4
102.850285028503 0.0658371240149863 2.85372784967098E-4
102.870287028703 0.0658314494220473 2.85299001299781E-4

102.890289028903 0.065825776370824 2.85225250392413E-4
102.910291029103 0.0658201048606834 2.85151532225305E-4
102.930293029303 0.0658144348909923 2.85077846778779E-4
102.950295029503 0.065808766461117 2.85004194033161E-4
102.970297029703 0.0658030995704276 2.84930573968836E-4
102.990299029903 0.0657974342182908 2.84856986566156E-4
103.010301030103 0.0657917704040765 2.84783431805522E-4
103.030303030303 0.0657861081271555 2.84709909667359E-4
103.050305030503 0.065780447386897 2.84636420132082E-4
103.070307030703 0.065774788182672 2.8456296318014E-4
103.090309030903 0.065769130513856 2.84489538792048E-4
103.110311031103 0.0657634743798169 2.84416146948233E-4
103.130313031303 0.0657578197799298 2.84342787629229E-4
103.150315031503 0.0657521667135686 2.8426946081556E-4
103.170317031703 0.065746515180107 2.84196166487759E-4
103.190319031903 0.0657408651789214 2.84122904626408E-4
103.210321032103 0.0657352167093857 2.84049675212062E-4
103.230323032303 0.0657295697708765 2.83976478225321E-4
103.250325032503 0.0657239243627714 2.8390331364681E-4
103.270327032703 0.0657182804844476 2.8383018145716E-4
103.290329032903 0.065712638135283 2.83757081637019E-4
103.310331033103 0.0657069973146564 2.83684014167057E-4
103.330333033303 0.0657013580219474 2.83610979027967E-4
103.350335033503 0.065695720256535 2.83537976200438E-4
103.370337033703 0.0656900840178018 2.83465005665223E-4
103.390339033903 0.0656844493051264 2.83392067403027E-4
103.410341034103 0.0656788161178923 2.83319161394634E-4
103.430343034303 0.0656731844554821 2.83246287620822E-4
103.450345034503 0.0656675543172785 2.83173446062383E-4
103.470347034703 0.065661925702664 2.83100636700114E-4
103.490349034904 0.0656562986110241 2.83027859514863E-4
103.510351035104 0.0656506730417436 2.82955114487478E-4
103.530353035304 0.0656450489942082 2.82882401598829E-4
103.550355035504 0.0656394264678029 2.82809720829784E-4
103.570357035704 0.0656338054619156 2.82737072161263E-4
103.590359035904 0.0656281859759328 2.82664455574173E-4
103.610361036104 0.0656225680092429 2.82591871049461E-4
103.630363036304 0.0656169515612349 2.82519318568086E-4
103.650365036504 0.0656113366312963 2.82446798111003E-4
103.670367036704 0.0656057232188179 2.82374309659214E-4
103.690369036904 0.0656001113231902 2.82301853193731E-4
103.710371037104 0.0655945009438033 2.82229428695567E-4
103.730373037304 0.0655888920800503 2.82157036145787E-4
103.750375037504 0.0655832847313225 2.82084675525442E-4

103.770377037704 0.0655776788970114 2.82012346815595E-4
103.790379037904 0.0655720745765121 2.81940049997362E-4
103.810381038104 0.0655664717692176 2.81867785051847E-4
103.830383038304 0.0655608704745228 2.81795551960185E-4
103.850385038504 0.065555270691822 2.81723350703513E-4
103.870387038704 0.0655496724205121 2.81651181263014E-4
103.890389038904 0.0655440756599889 2.81579043619864E-4
103.910391039104 0.0655384804096491 2.81506937755259E-4
103.930393039304 0.0655328866688903 2.81434863650421E-4
103.950395039504 0.0655272944371104 2.81362821286581E-4
103.970397039704 0.0655217037137083 2.81290810644994E-4
103.990399039904 0.0655161144980835 2.81218831706933E-4
104.010401040104 0.065510526789636 2.81146884453687E-4
104.030403040304 0.0655049405877648 2.81074968866543E-4
104.050405040504 0.0654993558918716 2.81003084926829E-4
104.070407040704 0.0654937727013593 2.80931232615901E-4
104.090409040904 0.0654881910156275 2.80859411915081E-4
104.110411041104 0.0654826108340815 2.80787622805774E-4
104.130413041304 0.0654770321561238 2.80715865269358E-4
104.150415041504 0.0654714549811577 2.80644139287233E-4
104.170417041704 0.0654658793085882 2.80572444840829E-4
104.190419041904 0.0654603051378198 2.80500781911577E-4
104.210421042104 0.065454732468259 2.80429150480947E-4
104.230423042304 0.0654491612993114 2.80357550530407E-4
104.250425042504 0.0654435916303839 2.80285982041446E-4
104.270427042704 0.065438023460884 2.80214444995576E-4
104.290429042904 0.0654324567902199 2.80142939374324E-4
104.310431043104 0.0654268916177996 2.80071465159227E-4
104.330433043304 0.0654213279430323 2.80000022331844E-4
104.350435043504 0.0654157657653284 2.79928610873761E-4
104.370437043704 0.0654102050840973 2.79857230766558E-4
104.390439043904 0.0654046458987489 2.79785881991833E-4
104.410441044104 0.0653990882086967 2.7971456453124E-4
104.430443044304 0.0653935320133508 2.79643278366398E-4
104.450445044504 0.0653879773121254 2.79572023478987E-4
104.470447044704 0.0653824241044331 2.79500799850676E-4
104.490449044904 0.0653768723896857 2.79429607463138E-4
104.510451045105 0.0653713221672986 2.79358446298093E-4
104.530453045305 0.0653657734366876 2.79287316337282E-4
104.550455045505 0.0653602261972668 2.79216217562431E-4
104.570457045705 0.0653546804484519 2.79145149955297E-4
104.590459045905 0.0653491361896603 2.79074113497668E-4
104.610461046105 0.0653435934203087 2.79003108171332E-4
104.630463046305 0.065338052139814 2.78932133958088E-4

104.650465046505 0.0653325123475949 2.78861190839768E-4
104.670467046705 0.0653269740430697 2.78790278798209E-4
104.690469046905 0.0653214372256585 2.78719397815281E-4
104.710471047105 0.0653159018947791 2.78648547872831E-4
104.730473047305 0.065310368049854 2.78577728952776E-4
104.750475047505 0.0653048356903032 2.78506941037014E-4
104.770477047705 0.0652993048155477 2.78436184107461E-4
104.790479047905 0.0652937754250091 2.78365458146051E-4
104.810481048105 0.0652882475181124 2.78294763134771E-4
104.830483048305 0.0652827210942776 2.78224099055554E-4
104.850485048505 0.0652771961529293 2.78153465890399E-4
104.870487048705 0.0652716726934925 2.78082863621322E-4
104.890489048905 0.0652661507153921 2.78012292230345E-4
104.910491049105 0.065260630218051 2.77941751699474E-4
104.930493049305 0.0652551112008978 2.77871242010797E-4
104.950495049505 0.0652495936633569 2.77800763146358E-4
104.970497049705 0.0652440776048556 2.77730315088247E-4
104.990499049905 0.0652385630248231 2.77659897818586E-4
105.010501050105 0.0652330499226849 2.7758951131946E-4
105.030503050305 0.0652275382978704 2.77519155573016E-4
105.050505050505 0.0652220281498085 2.77448830561399E-4
105.070507050705 0.0652165194779297 2.77378536266783E-4
105.090509050905 0.065211012281663 2.77308272671338E-4
105.110511051105 0.065205506560439 2.77238039757256E-4
105.130513051305 0.0652000023136892 2.77167837506758E-4
105.150515051505 0.0651944995408456 2.7709766590207E-4
105.170517051705 0.06518899824134 2.77027524925435E-4
105.190519051905 0.0651834984146052 2.7695741455911E-4
105.210521052105 0.0651780000600742 2.76887334785368E-4
105.230523052305 0.0651725031771829 2.76817285586527E-4
105.250525052505 0.0651670077653627 2.76747266944855E-4
105.270527052705 0.0651615138240499 2.76677278842693E-4
105.290529052905 0.0651560213526797 2.76607321262376E-4
105.310531053105 0.0651505303506888 2.76537394186269E-4
105.330533053305 0.0651450408175119 2.76467497596719E-4
105.350535053505 0.0651395527525874 2.76397631476128E-4
105.370537053705 0.0651340661553532 2.76327795806903E-4
105.390539053905 0.065128581025246 2.76257990571444E-4
105.410541054105 0.0651230973617042 2.7618821575218E-4
105.430543054305 0.0651176151641686 2.7611847133158E-4
105.450545054505 0.0651121344320779 2.76048757292097E-4
105.470547054705 0.0651066551648709 2.75979073616193E-4
105.490549054906 0.0651011773619896 2.7590942028638E-4
105.510551055106 0.0650957010228753 2.75839797285168E-4

105.530553055306 0.0650902261469691 2.75770204595074E-4
105.550555055506 0.0650847527337138 2.75700642198648E-4
105.570557055706 0.0650792807825513 2.75631110078436E-4
105.590559055906 0.0650738102929234 2.75561608216991E-4
105.610561056106 0.0650683412642774 2.75492136596944E-4
105.630563056306 0.0650628736960542 2.75422695200855E-4
105.650565056506 0.0650574075877013 2.75353284011377E-4
105.670567056706 0.0650519429386614 2.7528390301111E-4
105.690569056906 0.0650464797483824 2.75214552182728E-4
105.710571057106 0.065041018016308 2.7514523150886E-4
105.730573057306 0.0650355577418882 2.75075940972227E-4
105.750575057506 0.0650300989245674 2.75006680555486E-4
105.770577057706 0.0650246415637947 2.7493745024136E-4
105.790579057906 0.065019185659018 2.7486825001257E-4
105.810581058106 0.0650137312096858 2.7479907985185E-4
105.830583058306 0.0650082782152492 2.74729939741976E-4
105.850585058506 0.0650028266751548 2.74660829665677E-4
105.870587058706 0.0649973765888554 2.7459174960577E-4
105.890589058906 0.0649919279558011 2.74522699545043E-4
105.910591059106 0.0649864807754427 2.74453679466305E-4
105.930593059306 0.0649810350472317 2.7438468935238E-4
105.950595059506 0.0649755907706221 2.74315729186135E-4
105.970597059706 0.0649701479450649 2.74246798950406E-4
105.990599059906 0.0649647065700142 2.74177898628076E-4
106.010601060106 0.064959266644923 2.74109028202025E-4
106.030603060306 0.0649538281692456 2.74040187655158E-4
106.050605060506 0.0649483911424387 2.73971376970417E-4
106.070607060706 0.0649429555639544 2.73902596130693E-4
106.090609060906 0.0649375214332507 2.73833845118967E-4
106.110611061106 0.064932088749783 2.73765123918189E-4
106.130613061306 0.0649266575130073 2.73696432511328E-4
106.150615061506 0.0649212277223824 2.73627770881395E-4
106.170617061706 0.0649157993773644 2.73559139011379E-4
106.190619061906 0.0649103724774123 2.73490536884312E-4
106.210621062106 0.0649049470219838 2.73421964483217E-4
106.230623062306 0.0648995230105394 2.73353421791164E-4
106.250625062506 0.0648941004425383 2.73284908791212E-4
106.270627062706 0.0648886793174392 2.73216425466424E-4
106.290629062906 0.0648832596347053 2.73147971799929E-4
106.310631063106 0.0648778413937942 2.73079547774791E-4
106.330633063306 0.0648724245941714 2.73011153374185E-4
106.350635063506 0.0648670092352942 2.72942788581189E-4
106.370637063706 0.0648615953166296 2.72874453379014E-4
106.390639063906 0.0648561828376377 2.7280614775079E-4

106.410641064106 0.0648507717977826 2.72737871679709E-4
106.430643064306 0.0648453621965289 2.72669625148974E-4
106.450645064506 0.0648399540333395 2.72601408141779E-4
106.470647064706 0.0648345473076808 2.72533220641366E-4
106.490649064906 0.0648291420190174 2.72465062630968E-4
106.510651065107 0.0648237381668152 2.72396934093837E-4
106.530653065307 0.0648183357505394 2.7232883501323E-4
106.550655065507 0.0648129347696592 2.72260765372461E-4
106.570657065707 0.0648075352236392 2.72192725154791E-4
106.590659065907 0.0648021371119481 2.72124714343547E-4
106.610661066107 0.0647967404340547 2.7205673292206E-4
106.630663066307 0.0647913451894275 2.7198878087367E-4
106.650665066507 0.0647859513775344 2.71920858181715E-4
106.670667066707 0.0647805589978447 2.7185296482956E-4
106.690669066907 0.0647751680498299 2.71785100800607E-4
106.710671067107 0.0647697785329591 2.71717266078233E-4
106.730673067307 0.064764390446705 2.71649460645874E-4
106.750675067507 0.0647590037905366 2.71581684486922E-4
106.770677067707 0.0647536185639276 2.71513937584838E-4
106.790679067907 0.0647482347663491 2.71446219923061E-4
106.810681068107 0.0647428523972746 2.71378531485069E-4
106.830683068307 0.0647374714561761 2.71310872254323E-4
106.850685068507 0.06473209194253 2.71243242214355E-4
106.870687068707 0.0647267138558068 2.71175641348628E-4
106.890689068907 0.0647213371954838 2.71108069640698E-4
106.910691069107 0.0647159619610347 2.71040527074083E-4
106.930693069307 0.0647105881519354 2.70973013632342E-4
106.950695069507 0.064705215767661 2.70905529299027E-4
106.970697069707 0.0646998448076898 2.70838074057742E-4
106.990699069907 0.0646944752714961 2.70770647892044E-4
107.010701070107 0.0646891071585588 2.70703250785562E-4
107.030703070307 0.0646837404683553 2.70635882721911E-4
107.050705070507 0.0646783752003642 2.70568543684728E-4
107.070707070707 0.0646730113540631 2.70501233657651E-4
107.090709070907 0.0646676489289315 2.70433952624343E-4
107.110711071107 0.0646622879244485 2.70366700568475E-4
107.130713071307 0.0646569283400949 2.70299477473746E-4
107.150715071507 0.0646515701753501 2.70232283323845E-4
107.170717071707 0.0646462134296969 2.70165118102513E-4
107.190719071907 0.0646408581026138 2.70097981793445E-4
107.210721072107 0.0646355041935841 2.70030874380403E-4
107.230723072307 0.0646301517020895 2.69963795847139E-4
107.250725072507 0.064624800627613 2.69896746177428E-4
107.270727072707 0.0646194509696374 2.6982972535505E-4

107.290729072907 0.0646141027276457 2.69762733363798E-4
107.310731073107 0.0646087559011227 2.69695770187492E-4
107.330733073307 0.0646034104895515 2.69628835809943E-4
107.350735073507 0.0645980664924187 2.69561930215011E-4
107.370737073707 0.0645927239092072 2.69495053386519E-4
107.390739073907 0.0645873827394045 2.69428205308354E-4
107.410741074107 0.0645820429824954 2.69361385964379E-4
107.430743074307 0.0645767046379666 2.69294595338489E-4
107.450745074507 0.0645713677053056 2.69227833414595E-4
107.470747074707 0.0645660321839999 2.69161100176617E-4
107.490749074908 0.0645606980735353 2.69094395608465E-4
107.510751075108 0.064555365373403 2.69027719694117E-4
107.530753075308 0.0645500340830898 2.68961072417511E-4
107.550755075508 0.0645447042020845 2.68894453762616E-4
107.570757075708 0.0645393757298773 2.68827863713425E-4
107.590759075908 0.0645340486659585 2.68761302253939E-4
107.610761076108 0.0645287230098173 2.68694769368159E-4
107.630763076308 0.0645233987609445 2.68628265040109E-4
107.650765076508 0.0645180759188325 2.68561789253841E-4
107.670767076708 0.0645127544829723 2.68495341993399E-4
107.690769076908 0.0645074344528557 2.68428923242846E-4
107.710771077108 0.0645021158279745 2.68362532986249E-4
107.730773077308 0.064496798607822 2.68296171207707E-4
107.750775077508 0.0644914827918919 2.68229837891325E-4
107.770777077708 0.0644861683796779 2.68163533021223E-4
107.790779077908 0.0644808553706726 2.68097256581512E-4
107.810781078108 0.0644755437643724 2.68031008556357E-4
107.830783078308 0.064470233560271 2.67964788929899E-4
107.850785078508 0.0644649247578644 2.67898597686314E-4
107.870787078708 0.0644596173566485 2.67832434809787E-4
107.890789078908 0.0644543113561196 2.67766300284514E-4
107.910791079108 0.0644490067557714 2.67700194094666E-4
107.930793079308 0.0644437035551055 2.67634116224522E-4
107.950795079508 0.0644384017536148 2.67568066658259E-4
107.970797079708 0.0644331013508002 2.67502045380162E-4
107.990799079908 0.0644278023461594 2.67436052374484E-4
108.010801080108 0.0644225047391894 2.67370087625478E-4
108.030803080308 0.0644172085293905 2.67304151117444E-4
108.050805080508 0.0644119137162617 2.67238242834674E-4
108.070807080708 0.064406620299303 2.67172362761482E-4
108.090809080908 0.0644013282780154 2.671065108822E-4
108.110811081108 0.0643960376518973 2.67040687181135E-4
108.130813081308 0.0643907484204514 2.66974891642659E-4
108.150815081508 0.0643854605831794 2.66909124251136E-4

108.170817081708 0.0643801741395815 2.6684338499092E-4
108.190819081908 0.0643748890891612 2.66777673846413E-4
108.210821082108 0.0643696054314198 2.66711990801998E-4
108.230823082308 0.0643643231658617 2.66646335842104E-4
108.250825082508 0.0643590422919907 2.66580708951163E-4
108.270827082708 0.0643537628093088 2.66515110113587E-4
108.290829082908 0.0643484847173211 2.66449539313838E-4
108.310831083108 0.0643432080155324 2.66383996536381E-4
108.330833083308 0.0643379327034463 2.66318481765675E-4
108.350835083508 0.06433265878057 2.66252994986228E-4
108.370837083708 0.0643273862464076 2.66187536182522E-4
108.390839083908 0.0643221151004674 2.66122105339092E-4
108.410841084108 0.0643168453422537 2.66056702440439E-4
108.430843084308 0.064311576971274 2.65991327471106E-4
108.450845084508 0.0643063099870364 2.65925980415652E-4
108.470847084708 0.0643010443890476 2.65860661258627E-4
108.490849084908 0.0642957801768168 2.65795369984619E-4
108.510851085109 0.0642905173498512 2.65730106578201E-4
108.530853085309 0.0642852559076599 2.65664871023973E-4
108.550855085509 0.064279995849752 2.65599663306545E-4
108.570857085709 0.0642747371756387 2.65534483410562E-4
108.590859085909 0.0642694798848282 2.65469331320638E-4
108.610861086109 0.0642642239768317 2.65404207021429E-4
108.630863086309 0.0642589694511587 2.65339110497584E-4
108.650865086509 0.0642537163073227 2.65274041733802E-4
108.670867086709 0.0642484645448323 2.65209000714733E-4
108.690869086909 0.0642432141632015 2.65143987425102E-4
108.710871087109 0.0642379651619417 2.65079001849606E-4
108.730873087309 0.0642327175405659 2.65014043972971E-4
108.750875087509 0.0642274712985861 2.6494911377992E-4
108.770877087709 0.0642222264355178 2.64884211255223E-4
108.790879087909 0.0642169829508721 2.64819336383607E-4
108.810881088109 0.0642117408441649 2.64754489149863E-4
108.830883088309 0.0642065001149105 2.64689669538771E-4
108.850885088509 0.0642012607626225 2.6462487753511E-4
108.870887088709 0.0641960227868172 2.64560113123699E-4
108.890889088909 0.0641907861870107 2.64495376289362E-4
108.910891089109 0.0641855509627187 2.64430667016926E-4
108.930893089309 0.0641803171134563 2.64365985291219E-4
108.950895089509 0.0641750846387403 2.64301331097094E-4
108.970897089709 0.0641698535380898 2.64236704419445E-4
108.990899089909 0.0641646238110205 2.64172105243127E-4
109.010901090109 0.0641593954570502 2.64107533553031E-4
109.030903090309 0.0641541684756977 2.64042989334066E-4

109.050905090509 0.0641489428664821 2.63978472571153E-4
109.070907090709 0.0641437186289199 2.63913983249187E-4
109.090909090909 0.0641384957625322 2.63849521353131E-4
109.110911091109 0.0641332742668387 2.63785086867934E-4
109.130913091309 0.0641280541413597 2.63720679778561E-4
109.150915091509 0.0641228353856132 2.63656300069957E-4
109.170917091709 0.0641176179991212 2.63591947727122E-4
109.190919091909 0.0641124019814062 2.63527622735071E-4
109.210921092109 0.0641071873319873 2.63463325078783E-4
109.230923092309 0.0641019740503885 2.63399054743307E-4
109.250925092509 0.0640967621361296 2.63334811713646E-4
109.270927092709 0.064091551588734 2.63270595974853E-4
109.290929092909 0.0640863424077252 2.63206407511994E-4
109.310931093109 0.0640811345926242 2.63142246310108E-4
109.330933093309 0.064075928142957 2.63078112354303E-4
109.350935093509 0.0640707230582458 2.6301400562965E-4
109.370937093709 0.0640655193380176 2.62949926121282E-4
109.390939093909 0.0640603169817917 2.6288587381425E-4
109.410941094109 0.0640551159890979 2.62821848693737E-4
109.430943094309 0.0640499163594592 2.62757850744853E-4
109.450945094509 0.0640447180924014 2.62693879952747E-4
109.470947094709 0.0640395211874502 2.62629936302574E-4
109.49094909491 0.0640343256441327 2.62566019779517E-4
109.51095109511 0.0640291314619746 2.62502130368745E-4
109.53095309531 0.0640239386405045 2.62438268055472E-4
109.55095509551 0.0640187471792468 2.62374432824865E-4
109.57095709571 0.0640135570777327 2.62310624662187E-4
109.59095909591 0.064008368335487 2.62246843552626E-4
109.61096109611 0.0640031809520397 2.6218308948144E-4
109.63096309631 0.0639979949269194 2.62119362433879E-4
109.65096509651 0.0639928102596555 2.62055662395209E-4
109.67096709671 0.0639876269497752 2.61991989350679E-4
109.69096909691 0.0639824449968105 2.619283432856E-4
109.71097109711 0.0639772644002909 2.61864724185261E-4
109.73097309731 0.0639720851597462 2.61801132034964E-4
109.75097509751 0.0639669072747064 2.61737566820017E-4
109.77097709771 0.0639617307447036 2.61674028525764E-4
109.79097909791 0.0639565555692689 2.61610517137542E-4
109.81098109811 0.0639513817479353 2.61547032640719E-4
109.83098309831 0.0639462092802324 2.6148357502063E-4
109.85098509851 0.0639410381656936 2.61420144262659E-4
109.87098709871 0.0639358684038514 2.61356740352194E-4
109.89098909891 0.0639306999942396 2.61293363274637E-4
109.91099109911 0.0639255329363902 2.61230013015381E-4

109.93099309931 0.0639203672298376 2.61166689559854E-4
109.95099509951 0.0639152028741152 2.6110339289348E-4
109.97099709971 0.0639100398687596 2.61040123001727E-4
109.99099909991 0.0639048782133018 2.60976879870002E-4
110.01100110011 0.0638997179072798 2.60913663483807E-4
110.03100310031 0.0638945589502279 2.60850473828602E-4
110.05100510051 0.0638894013416798 2.6078731088985E-4
110.07100710071 0.0638842450811738 2.60724174653077E-4
110.09100910091 0.0638790901682463 2.60661065103791E-4
110.11101110111 0.0638739366024321 2.60597982227488E-4
110.13101310131 0.063868784383269 2.6053492600971E-4
110.15101510151 0.0638636335102939 2.60471896435991E-4
110.17101710171 0.0638584839830445 2.60408893491886E-4
110.19101910191 0.0638533358010581 2.6034591716295E-4
110.21102110211 0.0638481889638736 2.60282967434765E-4
110.23102310231 0.0638430434710306 2.60220044292926E-4
110.25102510251 0.0638378993220648 2.60157147722992E-4
110.27102710271 0.0638327565165174 2.60094277710593E-4
110.29102910291 0.0638276150539277 2.60031434241344E-4
110.31103110311 0.0638224749338352 2.59968617300868E-4
110.33103310331 0.0638173361557787 2.59905826874789E-4
110.35103510351 0.0638121987193005 2.59843062948779E-4
110.37103710371 0.0638070626239394 2.59780325508474E-4
110.39103910391 0.0638019278692378 2.59717614539561E-4
110.41104110411 0.0637967944547365 2.59654930027715E-4
110.43104310431 0.0637916623799769 2.59592271958624E-4
110.45104510451 0.063786531644501 2.59529640317994E-4
110.47104710471 0.0637814022478511 2.59467035091536E-4
110.491049104911 0.0637762741895687 2.59404456264963E-4
110.511051105111 0.0637711474691983 2.5934190382403E-4
110.531053105311 0.0637660220862816 2.59279377754461E-4
110.551055105511 0.0637608980403632 2.59216878042028E-4
110.571057105711 0.0637557753309845 2.59154404672469E-4
110.591059105911 0.063750653957692 2.59091957631588E-4
110.611061106111 0.0637455339200299 2.59029536905173E-4
110.631063106311 0.0637404152175408 2.58967142478996E-4
110.651065106511 0.0637352978497712 2.58904774338883E-4
110.671067106711 0.0637301818162658 2.58842432470647E-4
110.691069106911 0.063725067116569 2.58780116860103E-4
110.711071107111 0.0637199537502289 2.58717827493119E-4
110.731073107311 0.0637148417167908 2.58655564355534E-4
110.751075107511 0.0637097310157994 2.58593327433187E-4
110.771077107711 0.0637046216468017 2.58531116711954E-4
110.791079107911 0.0636995136093471 2.58468932177745E-4

110.811081108111 0.0636944069029812 2.58406773816429E-4
110.831083108311 0.0636893015272513 2.58344641613907E-4
110.851085108511 0.0636841974817068 2.58282535556107E-4
110.871087108711 0.0636790947658929 2.58220455628917E-4
110.891089108911 0.0636739933793605 2.58158401818301E-4
110.911091109111 0.0636688933216579 2.58096374110195E-4
110.931093109311 0.0636637945923332 2.58034372490542E-4
110.951095109511 0.0636586971909361 2.5797239694531E-4
110.971097109711 0.0636536011170167 2.5791044746048E-4
110.991099109911 0.0636485063701244 2.57848524022027E-4
111.011101110111 0.0636434129498095 2.57786626615952E-4
111.031103110311 0.0636383208556222 2.57724755228252E-4
111.051105110511 0.0636332300871141 2.57662909844955E-4
111.071107110711 0.063628140643834 2.57601090452058E-4
111.091109110911 0.0636230525253347 2.57539297035615E-4
111.111111111111 0.0636179657311695 2.57477529581691E-4
111.131113111311 0.063612880260887 2.57415788076304E-4
111.151115111511 0.063607796114041 2.57354072505537E-4
111.171117111711 0.0636027132901832 2.57292382855457E-4
111.191119111911 0.0635976317888673 2.57230719112164E-4
111.211121112111 0.0635925516096448 2.57169081261735E-4
111.231123112311 0.0635874727520716 2.57107469290308E-4
111.251125112511 0.0635823952156982 2.5704588318396E-4
111.271127112711 0.0635773190000784 2.5698432292882E-4
111.291129112911 0.0635722441047697 2.56922788511064E-4
111.311131113111 0.0635671705293233 2.56861279916799E-4
111.331133113311 0.0635620982732936 2.5679979713218E-4
111.351135113511 0.0635570273362383 2.56738340143403E-4
111.371137113711 0.0635519577177093 2.5667690893661E-4
111.391139113911 0.0635468894172648 2.56615503498015E-4
111.411141114111 0.0635418224344585 2.56554123813791E-4
111.431143114311 0.0635367567688477 2.56492769870159E-4
111.451145114511 0.0635316924199873 2.5643144165332E-4
111.471147114711 0.0635266293874346 2.56370139149508E-4
111.491149114911 0.0635215676707462 2.56308862344954E-4
111.511151115112 0.0635165072694801 2.56247611225916E-4
111.531153115312 0.0635114481831915 2.56186385778623E-4
111.551155115512 0.0635063904114397 2.56125185989361E-4
111.571157115712 0.0635013339537827 2.56064011844405E-4
111.591159115912 0.0634962788097764 2.56002863330014E-4
111.611161116112 0.0634912249789825 2.5594174043252E-4
111.631163116312 0.0634861724609561 2.55880643138186E-4
111.651165116512 0.0634811212552588 2.55819571433357E-4
111.671167116712 0.0634760713614489 2.55758525304347E-4

111.691169116912 0.0634710227790835 2.55697504737459E-4
111.711171117112 0.063465975507726 2.55636509719083E-4
111.731173117312 0.0634609295469346 2.55575540235548E-4
111.751175117512 0.0634558848962709 2.55514596273233E-4
111.771177117712 0.0634508415552932 2.55453677818485E-4
111.791179117912 0.0634457995235624 2.55392784857688E-4
111.811181118112 0.0634407588006393 2.55331917377229E-4
111.831183118312 0.0634357193860875 2.55271075363536E-4
111.851185118512 0.0634306812794659 2.55210258802992E-4
111.871187118712 0.0634256444803377 2.55149467682032E-4
111.891189118912 0.0634206089882638 2.55088701987072E-4
111.911191119112 0.0634155748028069 2.55027961704556E-4
111.931193119312 0.063410541923529 2.54967246820928E-4
111.951195119512 0.0634055103499936 2.54906557322653E-4
111.971197119712 0.0634004800817652 2.54845893196217E-4
111.991199119912 0.0633954511184027 2.54785254428046E-4
112.011201120112 0.063390423459473 2.54724641004666E-4
112.031203120312 0.0633853971045393 2.54664052912566E-4
112.051205120512 0.0633803720531646 2.54603490138241E-4
112.071207120712 0.0633753483049135 2.54542952668208E-4
112.091209120912 0.0633703258593507 2.54482440488995E-4
112.111211121112 0.0633653047160412 2.54421953587137E-4
112.131213121312 0.0633602848745496 2.54361491949176E-4
112.151215121512 0.0633552663344411 2.54301055561661E-4
112.171217121712 0.0633502490952807 2.54240644411148E-4
112.191219121912 0.0633452331566346 2.54180258484215E-4
112.211221122112 0.0633402185180685 2.5411989776744E-4
112.231223122312 0.0633352051791492 2.54059562247418E-4
112.251225122512 0.0633301931394419 2.53999251910734E-4
112.271227122712 0.0633251823985134 2.53938966743999E-4
112.291229122912 0.063320172955932 2.53878706733846E-4
112.311231123112 0.0633151648112632 2.53818471866882E-4
112.331233123312 0.0633101579640758 2.53758262129761E-4
112.351235123512 0.063305152413936 2.5369807750911E-4
112.371237123712 0.0633001481604126 2.53637917991595E-4
112.391239123912 0.0632951452030737 2.53577783563881E-4
112.411241124112 0.0632901435414864 2.53517674212623E-4
112.431243124312 0.0632851431752201 2.53457589924517E-4
112.451245124512 0.0632801441038429 2.53397530686243E-4
112.471247124712 0.0632751463269254 2.53337496484522E-4
112.491249124913 0.0632701498440356 2.53277487306047E-4
112.511251125113 0.0632651546547437 2.53217503137545E-4
112.531253125313 0.0632601607586182 2.5315754396573E-4
112.551255125513 0.0632551681552293 2.53097609777341E-4

112.571257125713 0.0632501768441483 2.53037700559138E-4
112.591259125913 0.0632451868249444 2.52977816297861E-4
112.611261126113 0.063240198097189 2.52917956980283E-4
112.631263126313 0.0632352106604519 2.52858122593164E-4
112.651265126513 0.0632302245143051 2.52798313123296E-4
112.671267126713 0.0632252396583208 2.52738528557483E-4
112.691269126913 0.0632202560920673 2.52678768882485E-4
112.711271127113 0.0632152738151196 2.52619034085149E-4
112.731273127313 0.0632102928270484 2.52559324152277E-4
112.751275127513 0.0632053131274254 2.52499639070693E-4
112.771277127713 0.0632003347158233 2.52439978827232E-4
112.791279127913 0.0631953575918154 2.52380343408748E-4
112.811281128113 0.0631903817549724 2.52320732802071E-4
112.831283128313 0.0631854072048715 2.52261146994108E-4
112.851285128513 0.0631804339410822 2.52201585971688E-4
112.871287128713 0.0631754619631801 2.52142049721715E-4
112.891289128913 0.0631704912707374 2.52082538231058E-4
112.911291129113 0.0631655218633289 2.52023051486629E-4
112.931293129313 0.0631605537405284 2.51963589475326E-4
112.951295129513 0.0631555869019104 2.51904152184068E-4
112.971297129713 0.0631506213470502 2.51844739599787E-4
112.991299129913 0.0631456570755219 2.51785351709407E-4
113.011301130113 0.0631406940869003 2.51725988499867E-4
113.031303130313 0.0631357323807611 2.51666649958124E-4
113.051305130513 0.0631307719566799 2.51607336071139E-4
113.071307130713 0.063125812814231 2.51548046825861E-4
113.091309130913 0.063120854952992 2.5148878220929E-4
113.111311131113 0.063115898372539 2.5142954220841E-4
113.131313131313 0.0631109430724468 2.51370326810198E-4
113.151315131513 0.0631059890522917 2.51311136001654E-4
113.171317131713 0.0631010363116522 2.51251969769811E-4
113.191319131913 0.0630960848501038 2.51192828101671E-4
113.211321132113 0.0630911346672256 2.51133710984287E-4
113.231323132313 0.0630861857625925 2.51074618404667E-4
113.251325132513 0.0630812381357833 2.51015550349873E-4
113.271327132713 0.0630762917863759 2.5095650680696E-4
113.291329132913 0.0630713467139479 2.50897487762987E-4
113.311331133113 0.063066402918077 2.50838493205019E-4
113.331333133313 0.0630614603983412 2.50779523120134E-4
113.351335133513 0.0630565191543212 2.50720577495446E-4
113.371337133713 0.0630515791855936 2.50661656318026E-4
113.391339133913 0.0630466404917375 2.5060275957498E-4
113.411341134113 0.0630417030723337 2.50543887253445E-4
113.431343134313 0.0630367669269599 2.50485039340521E-4

113.451345134513 0.0630318320551969 2.50426215823355E-4
113.471347134713 0.0630268984566231 2.5036741668907E-4
113.491349134913 0.0630219661308199 2.50308641924833E-4
113.511351135114 0.0630170350773673 2.50249891517795E-4
113.531353135314 0.0630121052958433 2.50191165455097E-4
113.551355135514 0.063007176785832 2.50132463723954E-4
113.571357135714 0.0630022495469123 2.50073786311523E-4
113.591359135914 0.0629973235786636 2.50015133204979E-4
113.611361136114 0.0629923988806705 2.4995650439156E-4
113.631363136314 0.0629874754525109 2.49897899858434E-4
113.651365136514 0.0629825532937682 2.49839319592835E-4
113.671367136714 0.0629776324040254 2.49780763582007E-4
113.691369136914 0.0629727127828616 2.49722231813145E-4
113.711371137114 0.0629677944298598 2.496637242735E-4
113.731373137314 0.0629628773446033 2.49605240950335E-4
113.751375137514 0.0629579615266736 2.49546781830894E-4
113.771377137714 0.0629530469756535 2.49488346902442E-4
113.791379137914 0.0629481336911265 2.49429936152262E-4
113.811381138114 0.0629432216726754 2.49371549567633E-4
113.831383138314 0.0629383109198822 2.4931318713583E-4
113.851385138514 0.062933401432332 2.4925484884417E-4
113.871387138714 0.0629284932096067 2.49196534679939E-4
113.891389138914 0.0629235862512921 2.49138244630478E-4
113.911391139114 0.0629186805569709 2.49079978683093E-4
113.931393139314 0.062913776126227 2.4902173682511E-4
113.951395139514 0.062908872958645 2.48963519043873E-4
113.971397139714 0.0629039710538095 2.48905325326731E-4
113.991399139914 0.062899070411307 2.48847155661059E-4
114.011401140114 0.0628941710307202 2.48789010034195E-4
114.031403140314 0.0628892729116341 2.487308884335509E-4
114.051405140514 0.0628843760536346 2.48672790846391E-4
114.071407140714 0.0628794804563075 2.4861471726023E-4
114.091409140914 0.0628745861192373 2.4855666766241E-4
114.111411141114 0.0628696930420115 2.48498642040355E-4
114.131413141314 0.0628648012242122 2.48440640381431E-4
114.151415141514 0.0628599106654312 2.48382662673127E-4
114.171417141714 0.0628550213652503 2.48324708902814E-4
114.191419141914 0.0628501333232586 2.48266779057962E-4
114.211421142114 0.0628452465390406 2.48208873125988E-4
114.231423142314 0.0628403610121854 2.4815099109437E-4
114.251425142514 0.0628354767422781 2.48093132950546E-4
114.271427142714 0.0628305937289071 2.48035298681999E-4
114.291429142914 0.0628257119716606 2.47977488276213E-4
114.311431143114 0.0628208314701239 2.47919701720648E-4

114.331433143314 0.0628159522238858 2.47861939002806E-4
114.351435143514 0.0628110742325331 2.47804200110175E-4
114.371437143714 0.0628061974956554 2.47746485030281E-4
114.391439143914 0.0628013220128412 2.47688793750643E-4
114.411441144114 0.0627964477836772 2.47631126258764E-4
114.431443144314 0.0627915748077516 2.47573482542171E-4
114.451445144514 0.0627867030846556 2.47515862588433E-4
114.471447144714 0.0627818326139771 2.47458266385084E-4
114.491449144915 0.0627769633953034 2.47400693919659E-4
114.511451145115 0.0627720954282281 2.4734314517977E-4
114.531453145315 0.0627672287123348 2.47285620152923E-4
114.551455145515 0.062762363247216 2.47228118826728E-4
114.571457145715 0.0627574990324624 2.47170641188783E-4
114.591459145915 0.0627526360676608 2.47113187226646E-4
114.611461146115 0.062747774352405 2.47055756927962E-4
114.631463146315 0.0627429138862819 2.46998350280302E-4
114.651465146515 0.0627380546688845 2.46940967271312E-4
114.671467146715 0.0627331966998022 2.46883607888607E-4
114.691469146915 0.0627283399786239 2.46826272119795E-4
114.711471147115 0.062723484504944 2.46768959952559E-4
114.731473147315 0.0627186302783516 2.46711671374524E-4
114.751475147515 0.0627137772984374 2.46654406373341E-4
114.771477147715 0.0627089255647924 2.46597164936669E-4
114.791479147915 0.0627040750770104 2.46539947052204E-4
114.811481148115 0.0626992258346808 2.464827527076E-4
114.831483148315 0.0626943778373958 2.46425581890549E-4
114.851485148515 0.0626895310847474 2.46368434588741E-4
114.871487148715 0.0626846855763286 2.46311310789892E-4
114.891489148915 0.0626798413117304 2.46254210481693E-4
114.911491149115 0.0626749982905467 2.46197133651879E-4
114.931493149315 0.0626701565123696 2.4614008028817E-4
114.951495149515 0.0626653159767894 2.46083050378266E-4
114.971497149715 0.0626604766834022 2.46026043909948E-4
114.991499149915 0.0626556386317988 2.45969060870932E-4
115.011501150115 0.0626508018215751 2.45912101249007E-4
115.031503150315 0.0626459662523218 2.45855165031902E-4
115.051505150515 0.0626411319236331 2.45798252207397E-4
115.071507150715 0.0626362988351017 2.45741362763257E-4
115.091509150915 0.0626314669863226 2.45684496687279E-4
115.111511151115 0.0626266363768912 2.45627653967273E-4
115.131513151315 0.0626218070063988 2.45570834591006E-4
115.151515151515 0.062616978874439 2.45514038546277E-4
115.171517151715 0.0626121519806105 2.45457265820951E-4
115.191519151915 0.0626073263245028 2.4540051640279E-4

115.211521152115 0.0626025019057143 2.45343790279667E-4
115.231523152315 0.0625976787238375 2.45287087439391E-4
115.251525152515 0.0625928567784687 2.45230407869821E-4
115.271527152715 0.0625880360692019 2.45173751558797E-4
115.291529152915 0.0625832165956346 2.45117118494203E-4
115.311531153115 0.0625783983573578 2.45060508663854E-4
115.331533153315 0.0625735813539709 2.45003922055667E-4
115.351535153515 0.0625687655850667 2.44947358657489E-4
115.371537153715 0.0625639510502444 2.44890818457249E-4
115.391539153915 0.0625591377490979 2.44834301442819E-4
115.411541154115 0.0625543256812225 2.44777807602089E-4
115.431543154315 0.0625495148462151 2.44721336922979E-4
115.451545154515 0.0625447052436731 2.44664889393417E-4
115.471547154715 0.0625398968731915 2.44608465001311E-4
115.491549154916 0.0625350897343685 2.4455206373461E-4
115.511551155116 0.0625302838267993 2.44495685581234E-4
115.531553155316 0.0625254791500797 2.44439330529116E-4
115.551555155516 0.0625206757038097 2.44382998566245E-4
115.571557155716 0.0625158734875849 2.44326689680562E-4
115.591559155916 0.0625110725010026 2.44270403860034E-4
115.611561156116 0.0625062727436597 2.4421414109263E-4
115.631563156316 0.0625014742151562 2.4415790136636E-4
115.651565156516 0.062496676915086 2.4410168466917E-4
115.671567156716 0.0624918808430504 2.44045490989095E-4
115.691569156916 0.0624870859986449 2.4398932031411E-4
115.711571157116 0.0624822923814695 2.43933172632247E-4
115.731573157316 0.0624774999911212 2.4387704793151E-4
115.751575157516 0.062472708827198 2.43820946199921E-4
115.771577157716 0.0624679188892991 2.43764867425518E-4
115.791579157916 0.0624631301770235 2.43708811596345E-4
115.811581158116 0.0624583426899682 2.43652778700428E-4
115.831583158316 0.062453556427733 2.4359676872583E-4
115.851585158516 0.0624487713899193 2.43540781660641E-4
115.871587158716 0.0624439875761216 2.43484817492873E-4
115.891589158916 0.0624392049859427 2.43428876210641E-4
115.911591159116 0.0624344236189812 2.43372957802018E-4
115.931593159316 0.0624296434748345 2.43317062255068E-4
115.951595159516 0.0624248645531051 2.43261189557919E-4
115.971597159716 0.0624200868533908 2.43205339698652E-4
115.991599159916 0.0624153103752918 2.43149512665379E-4
116.011601160116 0.062410535118409 2.43093708446226E-4
116.031603160316 0.0624057610823421 2.43037927029313E-4
116.051605160516 0.0624009882666905 2.4298216840276E-4
116.071607160716 0.0623962166710559 2.4292643255472E-4

116.091609160916 0.0623914462950373 2.42870719473318E-4
116.111611161116 0.0623866771382355 2.4281502914671E-4
116.131613161316 0.0623819092002526 2.42759361563066E-4
116.151615161516 0.0623771424806882 2.42703716710542E-4
116.171617161716 0.0623723769791421 2.42648094577291E-4
116.191619161916 0.0623676126952181 2.42592495151528E-4
116.211621162116 0.0623628496285149 2.42536918421404E-4
116.231623162316 0.0623580877786354 2.42481364375134E-4
116.251625162516 0.0623533271451802 2.42425833000907E-4
116.271627162716 0.0623485677277526 2.42370324286949E-4
116.291629162916 0.0623438095259486 2.42314838221405E-4
116.311631163116 0.0623390525393776 2.42259374792587E-4
116.331633163316 0.0623342967676381 2.4220393398868E-4
116.351635163516 0.0623295422103301 2.42148515797895E-4
116.371637163716 0.0623247888670577 2.42093120208494E-4
116.391639163916 0.0623200367374218 2.42037747208709E-4
116.411641164116 0.062315285821026 2.41982396786808E-4
116.431643164316 0.062310536117474 2.41927068931067E-4
116.451645164516 0.0623057876263654 2.41871763629719E-4
116.471647164716 0.0623010403473037 2.41816480871046E-4
116.491649164917 0.062296294279892 2.41761220643333E-4
116.511651165117 0.062291549423734 2.41705982934878E-4
116.531653165317 0.0622868057784325 2.41650767733971E-4
116.551655165517 0.0622820633435884 2.41595575028887E-4
116.571657165717 0.0622773221188059 2.41540404807946E-4
116.591659165917 0.0622725821036895 2.41485257059475E-4
116.611661166117 0.0622678432978428 2.41430131771799E-4
116.631663166317 0.0622631057008674 2.41375028933221E-4
116.651665166517 0.0622583693123675 2.41319948532081E-4
116.671667166717 0.0622536341319481 2.41264890556736E-4
116.691669166917 0.0622489001592109 2.41209854995507E-4
116.711671167117 0.0622441673937622 2.41154841836776E-4
116.731673167317 0.0622394358352034 2.41099851068874E-4
116.751675167517 0.0622347054831412 2.41044882680196E-4
116.771677167717 0.062229976337178 2.40989936659095E-4
116.791679167917 0.0622252483969182 2.40935012993951E-4
116.811681168117 0.0622205216619668 2.40880111673158E-4
116.831683168317 0.0622157961319276 2.408252326851E-4
116.851685168517 0.0622110718064067 2.40770376018194E-4
116.871687168717 0.0622063486850083 2.40715541660841E-4
116.891689168917 0.0622016267673361 2.40660729601439E-4
116.911691169117 0.0621969060529966 2.40605939828426E-4
116.931693169317 0.0621921865415926 2.40551172330203E-4
116.951695169517 0.0621874682327311 2.40496427095223E-4

116.971697169717 0.0621827511260178 2.4044170411193E-4
116.991699169917 0.0621780352210562 2.40387003368749E-4
117.011701170117 0.0621733205174516 2.4033232485413E-4
117.031703170317 0.0621686070148108 2.40277668556546E-4
117.051705170517 0.0621638947127399 2.40223034464468E-4
117.071707170717 0.0621591836108425 2.40168422566341E-4
117.091709170917 0.0621544737087256 2.40113832850658E-4
117.111711171117 0.0621497650059955 2.40059265305909E-4
117.131713171317 0.0621450575022576 2.40004719920575E-4
117.151715171517 0.0621403511971178 2.39950196683152E-4
117.171717171717 0.0621356460901824 2.39895695582144E-4
117.191719171917 0.0621309421810577 2.39841216606063E-4
117.211721172117 0.0621262394693496 2.39786759743417E-4
117.231723172317 0.0621215379546648 2.39732324982733E-4
117.251725172517 0.0621168376366103 2.39677912312544E-4
117.271727172717 0.0621121385147934 2.39623521721394E-4
117.291729172917 0.0621074405888188 2.395691531978E-4
117.311731173117 0.0621027438582934 2.39514806730312E-4
117.331733173317 0.0620980483228259 2.39460482307505E-4
117.351735173517 0.062093353982021 2.39406179917911E-4
117.371737173717 0.0620886608354881 2.39351899550127E-4
117.391739173917 0.0620839688828332 2.39297641192709E-4
117.411741174117 0.0620792781236622 2.39243404834223E-4
117.431743174317 0.0620745885575846 2.39189190463279E-4
117.451745174517 0.0620699001842065 2.39134998068454E-4
117.471747174717 0.0620652130031354 2.39080827638346E-4
117.491749174918 0.0620605270139791 2.39026679161562E-4
117.511751175118 0.0620558422163462 2.38972552626722E-4
117.531753175318 0.0620511586098429 2.38918448022426E-4
117.551755175518 0.0620464761940778 2.38864365337306E-4
117.571757175718 0.0620417949686579 2.38810304559981E-4
117.591759175918 0.0620371149331921 2.38756265679095E-4
117.611761176118 0.0620324360872884 2.38702248683291E-4
117.631763176318 0.0620277584305548 2.38648253561214E-4
117.651765176518 0.0620230819625994 2.38594280301515E-4
117.671767176718 0.0620184066830282 2.38540328892829E-4
117.691769176918 0.0620137325914535 2.38486399323869E-4
117.711771177118 0.0620090596874823 2.38432491583286E-4
117.731773177318 0.0620043879707241 2.38378605659773E-4
117.751775177518 0.0619997174407849 2.38324741541981E-4
117.771777177718 0.0619950480972739 2.38270899218611E-4
117.791779177918 0.0619903799398023 2.38217078678383E-4
117.811781178118 0.0619857129679753 2.38163279909962E-4
117.831783178318 0.0619810471814055 2.38109502902096E-4

117.851785178518 0.0619763825797 2.3805574764348E-4
117.871787178718 0.061971719162467 2.38002014122824E-4
117.891789178918 0.0619670569293168 2.37948302328868E-4
117.911791179118 0.0619623958798589 2.3789461225035E-4
117.931793179318 0.0619577360137022 2.37840943876005E-4
117.951795179518 0.0619530773304556 2.37787297194569E-4
117.971797179718 0.0619484198297286 2.37733672194797E-4
117.991799179918 0.0619437635111301 2.37680068865441E-4
118.011801180118 0.0619391083742735 2.37626487195304E-4
118.031803180318 0.0619344544187634 2.375729271731E-4
118.051805180518 0.0619298016442116 2.37519388787627E-4
118.071807180718 0.061925150050229 2.37465872027679E-4
118.091809180918 0.0619204996364227 2.37412376882013E-4
118.111811181118 0.0619158504024039 2.37358903339434E-4
118.131813181318 0.0619112023477836 2.37305451388754E-4
118.151815181518 0.0619065554721715 2.37252021018775E-4
118.171817181718 0.0619019097751767 2.37198612218294E-4
118.191819181918 0.0618972652564103 2.37145224976139E-4
118.211821182118 0.061892621915483 2.37091859281139E-4
118.231823182318 0.061887979752002 2.37038515122085E-4
118.251825182518 0.0618833387655827 2.36985192487871E-4
118.271827182718 0.0618786989558314 2.36931891367288E-4
118.291829182918 0.0618740603223608 2.36878611749207E-4
118.311831183118 0.0618694228647812 2.36825353622479E-4
118.331833183318 0.0618647865827022 2.36772116975948E-4
118.351835183518 0.0618601514757349 2.36718901798484E-4
118.371837183718 0.0618555175434898 2.36665708078952E-4
118.391839183918 0.0618508847855788 2.3661253580624E-4
118.411841184118 0.0618462532016134 2.36559384969237E-4
118.431843184318 0.0618416227912027 2.3650625555681E-4
118.451845184518 0.0618369935539574 2.36453147557846E-4
118.471847184718 0.06183236548949 2.36400060961261E-4
118.491849184918 0.0618277385974135 2.36346995755982E-4
118.511851185119 0.0618231128773338 2.36293951930857E-4
118.531853185319 0.061818488328867 2.36240929474859E-4
118.551855185519 0.0618138649516212 2.36187928376873E-4
118.571857185719 0.0618092427452102 2.36134948625854E-4
118.591859185919 0.0618046217092449 2.36081990210728E-4
118.611861186119 0.061800001843336 2.36029053120429E-4
118.631863186319 0.0617953831470945 2.35976137343891E-4
118.651865186519 0.0617907656201345 2.35923242870097E-4
118.671867186719 0.0617861492620657 2.35870369687979E-4
118.691869186919 0.0617815340724983 2.35817517786484E-4
118.711871187119 0.0617769200510478 2.35764687154618E-4

118.731873187319 0.0617723071973235 2.35711877781331E-4
118.751875187519 0.0617676955109393 2.35659089655622E-4
118.771877187719 0.0617630849915037 2.35606322766437E-4
118.791879187919 0.0617584756386333 2.3555357710282E-4
118.811881188119 0.0617538674519365 2.35500852653725E-4
118.831883188319 0.0617492604310255 2.35448149408156E-4
118.851885188519 0.0617446545755153 2.35395467355152E-4
118.871887188719 0.0617400498850167 2.35342806483712E-4
118.891889188919 0.0617354463591407 2.35290166782838E-4
118.911891189119 0.061730843997501 2.35237548241572E-4
118.931893189319 0.0617262427997091 2.35184950848934E-4
118.951895189519 0.0617216427653791 2.3513237459398E-4
118.971897189719 0.0617170438941202 2.35079819465713E-4
118.991899189919 0.0617124461855484 2.3502728545322E-4
119.011901190119 0.0617078496392747 2.34974772545536E-4
119.031903190319 0.061703254254912 2.34922280731721E-4
119.051905190519 0.0616986600320725 2.34869810000836E-4
119.071907190719 0.0616940669703693 2.34817360341954E-4
119.091909190919 0.0616894750694151 2.34764931744149E-4
119.111911191119 0.0616848843288228 2.34712524196502E-4
119.131913191319 0.0616802947482059 2.34660137688107E-4
119.151915191519 0.0616757063271754 2.34607772208033E-4
119.171917191719 0.0616711190653468 2.34555427745406E-4
119.191919191919 0.0616665329623301 2.34503104289298E-4
119.211921192119 0.0616619480177408 2.3445080182884E-4
119.231923192319 0.0616573642311905 2.34398520353133E-4
119.251925192519 0.0616527816022961 2.34346259851336E-4
119.271927192719 0.0616482001306645 2.34294020312503E-4
119.291929192919 0.0616436198159128 2.3424180172581E-4
119.311931193119 0.0616390406576535 2.34189604080382E-4
119.331933193319 0.0616344626555005 2.34137427365369E-4
119.351935193519 0.0616298858090664 2.34085271569907E-4
119.371937193719 0.0616253101179638 2.34033136683138E-4
119.391939193919 0.0616207355818081 2.33981022694246E-4
119.411941194119 0.0616161622002123 2.33928929592384E-4
119.431943194319 0.0616115899727905 2.33876857366729E-4
119.451945194519 0.0616070188991529 2.33824806006417E-4
119.471947194719 0.0616024489789172 2.33772775500673E-4
119.49194919492 0.0615978802116933 2.33720765838639E-4
119.51195119512 0.0615933125970981 2.33668777009542E-4
119.53195319532 0.0615887461347435 2.33616809002556E-4
119.55195519552 0.0615841808242449 2.33564861806901E-4
119.57195719572 0.0615796166652143 2.33512935411765E-4
119.59195919592 0.0615750536572662 2.33461029806362E-4

119.61196119612 0.0615704918000144 2.33409144979912E-4
119.63196319632 0.0615659310930731 2.33357280921641E-4
119.65196519652 0.0615613715360557 2.33305437620767E-4
119.67196719672 0.0615568131285762 2.33253615066526E-4
119.69196919692 0.0615522558702499 2.33201813248169E-4
119.71197119712 0.0615476997606891 2.33150032154921E-4
119.73197319732 0.0615431447995096 2.3309827177605E-4
119.75197519752 0.0615385909863224 2.33046532100776E-4
119.77197719772 0.0615340383207467 2.32994813118413E-4
119.79197919792 0.0615294868023928 2.32943114818182E-4
119.81198119812 0.0615249364308765 2.32891437189374E-4
119.83198319832 0.0615203872058094 2.32839780221232E-4
119.85198519852 0.0615158391268099 2.32788143903083E-4
119.87198719872 0.0615112921934904 2.32736528224193E-4
119.89198919892 0.0615067464054649 2.32684933173846E-4
119.91199119912 0.0615022017623477 2.32633358741341E-4
119.93199319932 0.0614976582637523 2.32581804915966E-4
119.95199519952 0.0614931159092965 2.32530271687069E-4
119.97199719972 0.0614885746985928 2.32478759043938E-4
119.99199919992 0.061484034631255 2.32427266975882E-4
120.01200120012 0.0614794957068971 2.32375795472218E-4
120.03200320032 0.0614749579251358 2.32324344522298E-4
120.05200520052 0.0614704212855851 2.32272914115448E-4
120.07200720072 0.0614658857878572 2.3222150424098E-4
120.09200920092 0.0614613514315727 2.32170114888302E-4
120.11201120112 0.0614568182163386 2.32118746046679E-4
120.13201320132 0.0614522861417746 2.32067397705519E-4
120.15201520152 0.0614477552074956 2.32016069854182E-4
120.17201720172 0.0614432254131139 2.31964762482006E-4
120.19201920192 0.0614386967582458 2.31913475578377E-4
120.21202120212 0.0614341692425052 2.31862209132659E-4
120.23202320232 0.0614296428655067 2.31810963134228E-4
120.25202520252 0.0614251176268679 2.31759737572499E-4
120.27202720272 0.0614205935262 2.31708532436822E-4
120.29202920292 0.0614160705631208 2.31657347716623E-4
120.31203120312 0.0614115487372444 2.31606183401293E-4
120.33203320332 0.0614070280481844 2.31555039480222E-4
120.35203520352 0.0614025084955567 2.3150391594283E-4
120.37203720372 0.0613979900789768 2.31452812778538E-4
120.39203920392 0.061393472798059 2.31401729976757E-4
120.41204120412 0.0613889566524183 2.31350667526914E-4
120.43204320432 0.0613844416416717 2.31299625418459E-4
120.45204520452 0.0613799277654323 2.31248603640806E-4
120.47204720472 0.0613754150233165 2.3119760218341E-4

120.49204920492 0.0613709034149364 2.31146621035682E-4
120.512051205121 0.0613663929399116 2.31095660187123E-4
120.532053205321 0.0613618835978548 2.31044719627161E-4
120.552055205521 0.0613573753883799 2.30993799345241E-4
120.572057205721 0.0613528683111064 2.30942899330879E-4
120.592059205921 0.0613483623656456 2.308920195735E-4
120.612061206121 0.0613438575516143 2.30841160062597E-4
120.632063206321 0.0613393538686285 2.30790320787659E-4
120.652065206521 0.0613348513162995 2.30739501738127E-4
120.672067206721 0.0613303498942477 2.3068870290355E-4
120.692069206921 0.0613258496020868 2.30637924273404E-4
120.712071207121 0.0613213504394329 2.30587165837199E-4
120.732073207321 0.0613168524058987 2.30536427584409E-4
120.752075207521 0.0613123555011018 2.30485709504573E-4
120.772077207721 0.0613078597246556 2.30435011587181E-4
120.792079207921 0.0613033650761784 2.30384333821787E-4
120.812081208121 0.0612988715552834 2.30333676197893E-4
120.832083208321 0.0612943791615876 2.30283038705046E-4
120.852085208521 0.061289887894706 2.30232421332774E-4
120.872087208721 0.0612853977542524 2.30181824070601E-4
120.892089208921 0.0612809087398443 2.30131246908095E-4
120.912091209121 0.061276420851097 2.30080689834801E-4
120.932093209321 0.0612719340876251 2.30030152840268E-4
120.952095209521 0.0612674484490438 2.29979635914049E-4
120.972097209721 0.0612629639349709 2.29929139045735E-4
120.992099209921 0.0612584805450211 2.29878662224887E-4
121.012101210121 0.0612539982788082 2.29828205441058E-4
121.032103210321 0.0612495171359479 2.29777768683832E-4
121.052105210521 0.0612450371160601 2.29727351942837E-4
121.072107210721 0.0612405582187543 2.29676955207599E-4
121.092109210921 0.0612360804436518 2.29626578467772E-4
121.112111211121 0.0612316037903621 2.29576221712887E-4
121.132113211321 0.061227128258507 2.29525884932619E-4
121.152115211521 0.0612226538476977 2.29475568116524E-4
121.172117211721 0.0612181805575551 2.29425271254275E-4
121.192119211921 0.0612137083876879 2.29374994335414E-4
121.212121212121 0.061209237337717 2.2932473734962E-4
121.232123212321 0.0612047674072547 2.29274500286485E-4
121.252125212521 0.0612002985959202 2.2922428313568E-4
121.272127212721 0.0611958309033273 2.29174085886821E-4
121.292129212921 0.0611913643290902 2.29123908529535E-4
121.312131213121 0.0611868988728271 2.29073751053495E-4
121.332133213321 0.0611824345341524 2.29023613448338E-4
121.352135213521 0.0611779713126841 2.28973495703747E-4

121.372137213721 0.0611735092080334 2.28923397809334E-4
121.392139213921 0.0611690482198202 2.28873319754809E-4
121.412141214121 0.0611645883476587 2.28823261529827E-4
121.432143214321 0.061160129591165 2.28773223124068E-4
121.452145214521 0.0611556719499561 2.28723204527225E-4
121.472147214721 0.0611512154236451 2.28673205728954E-4
121.492149214922 0.0611467600118472 2.28623226718938E-4
121.512151215122 0.0611423057141822 2.28573267486918E-4
121.532153215322 0.0611378525302647 2.2852332802258E-4
121.552155215522 0.0611334004597071 2.28473408315588E-4
121.572157215722 0.0611289495021297 2.28423508355703E-4
121.592159215922 0.0611244996571447 2.28373628132599E-4
121.612161216122 0.0611200509243706 2.28323767636023E-4
121.632163216322 0.0611156033034217 2.28273926855682E-4
121.652165216522 0.061111156793915 2.28224105781316E-4
121.672167216722 0.0611067113954644 2.28174304402638E-4
121.692169216922 0.0611022671076868 2.28124522709396E-4
121.712171217122 0.0610978239301991 2.28074760691343E-4
121.732173217322 0.0610933818626154 2.28025018338206E-4
121.752175217522 0.0610889409045507 2.27975295639729E-4
121.772177217722 0.0610845010556255 2.27925592585719E-4
121.792179217922 0.0610800623154499 2.27875909165872E-4
121.812181218122 0.0610756246836414 2.27826245369971E-4
121.832183218322 0.0610711881598203 2.27776601187838E-4
121.852185218522 0.0610667527435977 2.27726976609193E-4
121.872187218722 0.0610623184345885 2.27677371623805E-4
121.892189218922 0.0610578852324121 2.27627786221496E-4
121.912191219122 0.0610534531366825 2.27578220392036E-4
121.932193219322 0.0610490221470146 2.27528674125207E-4
121.952195219522 0.0610445922630276 2.27479147410843E-4
121.972197219722 0.0610401634843341 2.27429640238711E-4
121.992199219922 0.0610357358105491 2.27380152598608E-4
122.012201220122 0.0610313092412943 2.27330684480412E-4
122.032203220322 0.0610268837761779 2.27281235873851E-4
122.052205220522 0.0610224594148207 2.27231806768805E-4
122.072207220722 0.0610180361568373 2.27182397155082E-4
122.092209220922 0.0610136140018413 2.2713300702249E-4
122.112211221122 0.0610091929494517 2.27083636360895E-4
122.132213221322 0.0610047729992826 2.27034285160119E-4
122.152215221522 0.0610003541509507 2.26984953410016E-4
122.172217221722 0.0609959364040722 2.26935641100436E-4
122.192219221922 0.0609915197582604 2.26886348221205E-4
122.212221222122 0.0609871042131348 2.2683707476222E-4
122.232223222322 0.060982689768307 2.26787820713299E-4

122.252225222522 0.0609782764233956 2.26738586064335E-4
122.272227222722 0.0609738641780141 2.26689370805176E-4
122.292229222922 0.0609694530317801 2.26640174925717E-4
122.312231223122 0.06096504298431 2.26590998415845E-4
122.332233223322 0.0609606340352171 2.26541841265415E-4
122.352235223522 0.0609562261841203 2.26492703464351E-4
122.372237223722 0.0609518194306312 2.264435850025E-4
122.392239223922 0.0609474137743702 2.26394485869814E-4
122.412241224122 0.0609430092149476 2.2634540605613E-4
122.432243224322 0.0609386057519843 2.26296345551415E-4
122.452245224522 0.0609342033850928 2.26247304345544E-4
122.472247224722 0.0609298021138904 2.2619828242845E-4
122.492249224923 0.0609254019379909 2.26149279790031E-4
122.512251225123 0.0609210028570106 2.26100296420218E-4
122.532253225323 0.0609166048705668 2.26051332308958E-4
122.552255225523 0.0609122079782739 2.26002387446171E-4
122.572257225723 0.0609078121797484 2.25953461821803E-4
122.592259225923 0.0609034174746054 2.25904555425791E-4
122.612261226123 0.060899023862458 2.25855668248052E-4
122.632263226323 0.0608946313429255 2.25806800278579E-4
122.652265226523 0.0608902399156214 2.25757951507303E-4
122.672267226723 0.0608858495801632 2.25709121924204E-4
122.692269226923 0.0608814603361629 2.25660311519206E-4
122.712271227123 0.060877072183241 2.25611520282335E-4
122.732273227323 0.0608726851210075 2.255627482035E-4
122.752275227523 0.0608682991490842 2.25513995272747E-4
122.772277227723 0.0608639142670801 2.25465261479986E-4
122.792279227923 0.0608595304746146 2.25416546815245E-4
122.812281228123 0.0608551477713039 2.25367851268525E-4
122.832283228323 0.0608507661567606 2.25319174829785E-4
122.852285228523 0.0608463856306009 2.25270517489034E-4
122.872287228723 0.0608420061924415 2.25221879236286E-4
122.892289228923 0.0608376278418972 2.25173260061546E-4
122.912291229123 0.0608332505785835 2.25124659954824E-4
122.932293229323 0.0608288744021154 2.25076078906134E-4
122.952295229523 0.0608244993121084 2.25027516905494E-4
122.972297229723 0.060820125308179 2.24978973942944E-4
122.992299229923 0.0608157523899409 2.24930450008495E-4
123.012301230123 0.0608113805570106 2.24881945092192E-4
123.032303230323 0.0608070098090023 2.24833459184061E-4
123.052305230523 0.060802640145532 2.24784992274152E-4
123.072307230723 0.060798271566215 2.24736544352511E-4
123.092309230923 0.060793904070667 2.24688115409193E-4
123.112311231123 0.0607895376585031 2.24639705434253E-4

123.132313231323 0.060785172329338 2.24591314417742E-4
123.152315231523 0.0607808080827863 2.24542942349719E-4
123.172317231723 0.0607764449184658 2.24494589220279E-4
123.192319231923 0.0607720828359898 2.24446255019474E-4
123.212321232123 0.0607677218349735 2.24397939737379E-4
123.232323232323 0.0607633619150306 2.24349643364062E-4
123.252325232523 0.0607590030757789 2.24301365889635E-4
123.272327232723 0.0607546453168331 2.24253107304182E-4
123.292329232923 0.0607502886378073 2.24204867597787E-4
123.312331233123 0.0607459330383165 2.24156646760546E-4
123.332333233323 0.0607415785179786 2.24108444782593E-4
123.352335233523 0.0607372250764037 2.24060261653978E-4
123.372337233723 0.060732872713209 2.24012097364839E-4
123.392339233923 0.0607285214280131 2.23963951905326E-4
123.412341234123 0.0607241712204249 2.23915825265494E-4
123.432343234323 0.0607198220900637 2.23867717435512E-4
123.452345234523 0.0607154740365431 2.23819628405495E-4
123.472347234723 0.0607111270594787 2.2377155816558E-4
123.492349234923 0.0607067811584833 2.23723506705879E-4
123.512351235124 0.0607024363331735 2.23675474016553E-4
123.532353235324 0.0606980925831626 2.23627460087727E-4
123.552355235524 0.0606937499080684 2.23579464909583E-4
123.572357235724 0.0606894083075034 2.23531488472249E-4
123.592359235924 0.0606850677810834 2.23483530765891E-4
123.612361236124 0.0606807283284218 2.23435591780657E-4
123.632363236324 0.0606763899491373 2.23387671506752E-4
123.652365236524 0.0606720526428374 2.23339769934269E-4
123.672367236724 0.060667716409144 2.2329188705346E-4
123.692369236924 0.0606633812476682 2.23244022854463E-4
123.712371237124 0.0606590471580256 2.2319617732747E-4
123.732373237324 0.0606547141398299 2.23148350462655E-4
123.752375237524 0.0606503821926965 2.2310054225022E-4
123.772377237724 0.0606460513162397 2.23052752680353E-4
123.792379237924 0.0606417215100743 2.23004981743258E-4
123.812381238124 0.060637392773815 2.22957229429136E-4
123.832383238324 0.0606330651070765 2.22909495728198E-4
123.852385238524 0.0606287385094733 2.22861780630653E-4
123.872387238724 0.0606244129806197 2.22814084126716E-4
123.892389238924 0.0606200885201288 2.22766406206591E-4
123.912391239124 0.0606157651276152 2.22718746860501E-4
123.932393239324 0.060611442802697 2.22671106078716E-4
123.952395239524 0.0606071215449845 2.22623483851421E-4
123.972397239724 0.0606028013540938 2.22575880168873E-4
123.992399239924 0.0605984822296388 2.22528295021305E-4

124.012401240124 0.0605941641712323 2.22480728398945E-4
124.032403240324 0.0605898471784921 2.2243318029208E-4
124.052405240524 0.0605855312510298 2.22385650690933E-4
124.072407240724 0.0605812163884609 2.22338139585772E-4
124.092409240924 0.0605769025903969 2.2229064696683E-4
124.112411241124 0.0605725898564559 2.22243172824413E-4
124.132413241324 0.0605682781862501 2.22195717148768E-4
124.152415241524 0.060563967579393 2.22148279930161E-4
124.172417241724 0.0605596580355004 2.22100861158887E-4
124.192419241924 0.060555349554184 2.220534608252E-4
124.212421242124 0.0605510421350608 2.22006078919418E-4
124.232423242324 0.0605467357777421 2.21958715431799E-4
124.252425242524 0.0605424304818436 2.21911370352655E-4
124.272427242724 0.0605381262469771 2.21864043672259E-4
124.292429242924 0.0605338230727598 2.21816735380946E-4
124.312431243124 0.060529520958802 2.21769445468984E-4
124.332433243324 0.0605252199047213 2.21722173926722E-4
124.352435243524 0.0605209199101285 2.21674920744438E-4
124.372437243724 0.0605166209746391 2.21627685912469E-4
124.392439243924 0.0605123230978654 2.21580469421118E-4
124.412441244124 0.0605080262794215 2.21533271260715E-4
124.432443244324 0.0605037305189229 2.21486091421608E-4
124.452445244524 0.0604994358159828 2.21438929894125E-4
124.472447244724 0.0604951421702126 2.21391786668577E-4
124.492449244925 0.0604908495812278 2.21344661735326E-4
124.512451245125 0.0604865580486419 2.21297555084715E-4
124.532453245325 0.0604822675720688 2.21250466707098E-4
124.552455245525 0.0604779781511192 2.21203396592794E-4
124.572457245725 0.0604736897854097 2.21156344732196E-4
124.592459245925 0.0604694024745532 2.21109311115659E-4
124.612461246125 0.060465116218164 2.21062295733555E-4
124.632463246325 0.0604608310158526 2.21015298576222E-4
124.652465246525 0.0604565468672356 2.20968319634066E-4
124.672467246725 0.0604522637719229 2.20921358897429E-4
124.692469246925 0.0604479817295315 2.20874416356731E-4
124.712471247125 0.0604437007396717 2.20827492002322E-4
124.732473247325 0.0604394208019588 2.20780585824614E-4
124.752475247525 0.0604351419160059 2.20733697813999E-4
124.772477247725 0.060430864081425 2.20686827960859E-4
124.792479247925 0.0604265872978276 2.20639976255576E-4
124.812481248125 0.0604223115648318 2.20593142688611E-4
124.832483248325 0.0604180368820473 2.20546327250333E-4
124.852485248525 0.0604137632490868 2.20499529931149E-4
124.872487248725 0.0604094906655655 2.20452750721502E-4

124.892489248925 0.0604052191310939 2.20405989611783E-4
124.912491249125 0.060400948645288 2.20359246592449E-4
124.932493249325 0.0603966792077582 2.20312521653901E-4
124.952495249525 0.0603924108181177 2.20265814786573E-4
124.972497249725 0.0603881434759801 2.20219125980909E-4
124.992499249925 0.0603838771809581 2.20172455227348E-4
125.012501250125 0.0603796119326633 2.2012580251632E-4
125.032503250325 0.060375347730711 2.200791678383E-4
125.052505250525 0.0603710845747115 2.20032551183712E-4
125.072507250725 0.060366822464279 2.19985952543028E-4
125.092509250925 0.0603625613990249 2.19939371906695E-4
125.112511251125 0.060358301378563 2.19892809265186E-4
125.132513251325 0.0603540424025041 2.1984626460895E-4
125.152515251525 0.0603497844704638 2.19799737928491E-4
125.172517251725 0.0603455275820514 2.19753229214247E-4
125.192519251925 0.0603412717368823 2.1970673845673E-4
125.212521252125 0.0603370169345669 2.19660265646399E-4
125.232523252325 0.0603327631747184 2.19613810773749E-4
125.252525252525 0.06032851045695 2.19567373829278E-4
125.272527252725 0.0603242587808694 2.1952095480343E-4
125.292529252925 0.060320008146096 2.19474553686782E-4
125.312531253125 0.0603157585522366 2.19428170469773E-4
125.332533253325 0.060311509998906 2.19381805142936E-4
125.352535253525 0.060307262485716 2.1933545769678E-4
125.372537253725 0.0603030160122773 2.19289128121797E-4
125.392539253925 0.0602987705782058 2.1924281640855E-4
125.412541254125 0.0602945261831101 2.19196522547518E-4
125.432543254325 0.0602902828266026 2.19150246529231E-4
125.452545254525 0.0602860405082957 2.19103988344221E-4
125.472547254725 0.0602817992278003 2.19057747983008E-4
125.492549254925 0.0602775589847325 2.19011525436176E-4
125.512551255126 0.0602733197786994 2.18965320694209E-4
125.532553255326 0.0602690816093151 2.18919133747679E-4
125.552555255526 0.0602648444761931 2.18872964587147E-4
125.572557255726 0.0602606083789422 2.18826813203135E-4
125.592559255926 0.060256373317174 2.18780679586196E-4
125.612561256126 0.0602521392905015 2.18734563726902E-4
125.632563256326 0.0602479062985377 2.18688465615831E-4
125.652565256526 0.0602436743408909 2.18642385243512E-4
125.672567256726 0.0602394434171756 2.18596322600547E-4
125.692569256926 0.0602352135270033 2.1855027767751E-4
125.712571257126 0.060230984669983 2.18504250464952E-4
125.732573257326 0.06022675684573 2.18458240953495E-4
125.752575257526 0.0602225300538518 2.1841224913368E-4

125.772577257726 0.0602183042939603 2.18366274996105E-4
125.792579257926 0.0602140795656695 2.18320318531389E-4
125.812581258126 0.0602098558685908 2.1827437973013E-4
125.832583258326 0.060205633202333 2.18228458582901E-4
125.852585258526 0.0602014115665081 2.18182555080316E-4
125.872587258726 0.0601971909607268 2.18136669212976E-4
125.892589258926 0.0601929713846022 2.18090800971513E-4
125.912591259126 0.0601887528377433 2.18044950346521E-4
125.932593259326 0.0601845353197641 2.1799911732865E-4
125.952595259526 0.0601803188302714 2.17953301908476E-4
125.972597259726 0.0601761033688799 2.17907504076666E-4
125.992599259926 0.0601718889352008 2.17861723823854E-4
126.012601260126 0.0601676755288403 2.17815961140622E-4
126.032603260326 0.0601634631494139 2.17770216017657E-4
126.052605260526 0.0601592517965323 2.17724488445601E-4
126.072607260726 0.0601550414698049 2.17678778415083E-4
126.092609260926 0.0601508321688411 2.17633085916736E-4
126.112611261126 0.0601466238932526 2.17587410941225E-4
126.132613261326 0.0601424166426539 2.17541753479247E-4
126.152615261526 0.060138210416651 2.17496113521411E-4
126.172617261726 0.0601340052148547 2.17450491058386E-4
126.192619261926 0.060129801036878 2.17404886080862E-4
126.212621262126 0.0601255978823312 2.1735929857951E-4
126.232623262326 0.0601213957508234 2.17313728544991E-4
126.252625262526 0.0601171946419657 2.17268175967988E-4
126.272627262726 0.0601129945553669 2.17222640839169E-4
126.292629262926 0.0601087954906402 2.17177123149249E-4
126.312631263126 0.0601045974473947 2.17131622888904E-4
126.332633263326 0.0601004004252392 2.17086140048815E-4
126.352635263526 0.0600962044237883 2.17040674619722E-4
126.372637263726 0.0600920094426461 2.16995226592262E-4
126.392639263926 0.0600878154814277 2.16949795957194E-4
126.412641264126 0.0600836225397409 2.16904382705198E-4
126.432643264326 0.0600794306171962 2.16858986826995E-4
126.452645264526 0.0600752397134047 2.16813608313309E-4
126.472647264726 0.0600710498279752 2.16768247154848E-4
126.492649264927 0.0600668609605171 2.16722903342327E-4
126.512651265127 0.0600626731106416 2.16677576866488E-4
126.532653265327 0.0600584862779586 2.16632267718061E-4
126.552655265527 0.060054300462076 2.16586975887761E-4
126.572657265727 0.0600501156626081 2.16541701366372E-4
126.592659265927 0.0600459318791597 2.16496444144583E-4
126.612661266127 0.0600417491113423 2.16451204213159E-4
126.632663266327 0.0600375673587668 2.16405981562861E-4

126.652665266527 0.0600333866210414 2.16360776184426E-4
126.672667266727 0.060029206897775 2.16315588068603E-4
126.692669266927 0.0600250281885796 2.16270417206178E-4
126.712671267127 0.0600208504930612 2.16225263587876E-4
126.732673267327 0.0600166738108325 2.16180127204501E-4
126.752675267527 0.0600124981415036 2.16135008046828E-4
126.772677267727 0.0600083234846806 2.16089906105599E-4
126.792679267927 0.0600041498399739 2.160448213716E-4
126.812681268127 0.0599999772069929 2.15999753835617E-4
126.832683268327 0.0599958055853479 2.15954703488444E-4
126.852685268527 0.0599916349746475 2.15909670320865E-4
126.872687268727 0.059987465374498 2.1586465432364E-4
126.892689268927 0.0599832967845153 2.15819655487638E-4
126.912691269127 0.0599791292043024 2.15774673803596E-4
126.932693269327 0.0599749626334703 2.15729709262335E-4
126.952695269527 0.0599707970716289 2.15684761854673E-4
126.972697269727 0.059966632518384 2.15639831571385E-4
126.992699269927 0.0599624689733499 2.15594918403346E-4
127.012701270127 0.0599583064361287 2.155500223413E-4
127.032703270327 0.0599541449063353 2.15505143376133E-4
127.052705270527 0.0599499843835759 2.15460281498642E-4
127.072707270727 0.0599458248674586 2.15415436699649E-4
127.092709270927 0.0599416663575935 2.15370608970002E-4
127.112711271127 0.0599375088535871 2.15325798300512E-4
127.132713271327 0.0599333523550506 2.15281004682048E-4
127.152715271527 0.0599291968615908 2.15236228105432E-4
127.172717271727 0.0599250423728162 2.15191468561511E-4
127.192719271927 0.059920888888338 2.15146726041166E-4
127.212721272127 0.0599167364077604 2.15102000535203E-4
127.232723272327 0.0599125849306951 2.15057292034515E-4
127.252725272527 0.0599084344567498 2.15012600529957E-4
127.272727272727 0.0599042849855303 2.14967926012367E-4
127.292729272927 0.0599001365166474 2.14923268472637E-4
127.312731273127 0.0598959890497104 2.14878627901653E-4
127.332733273327 0.0598918425843224 2.14834004290236E-4
127.352735273527 0.0598876971200979 2.14789397629331E-4
127.372737273727 0.0598835526566416 2.14744807909784E-4
127.392739273927 0.0598794091935595 2.14700235122462E-4
127.412741274127 0.059875266730464 2.14655679258298E-4
127.432743274327 0.0598711252669613 2.14611140308168E-4
127.452745274527 0.059866984802658 2.14566618262953E-4
127.472747274727 0.0598628453371625 2.1452211311356E-4
127.492749274928 0.0598587068700842 2.1447762485091E-4
127.512751275128 0.0598545694010279 2.14433153465877E-4

127.532753275328 0.0598504329296055 2.14388698949416E-4
127.552755275528 0.0598462974554213 2.14344261292406E-4
127.572757275728 0.0598421629780845 2.14299840485782E-4
127.592759275928 0.0598380294972023 2.14255436520459E-4
127.612761276128 0.0598338970123805 2.14211049387343E-4
127.632763276328 0.0598297655232289 2.1416667907739E-4
127.652765276528 0.0598256350293552 2.14122325581531E-4
127.672767276728 0.0598215055303664 2.14077988890698E-4
127.692769276928 0.0598173770258676 2.14033668995806E-4
127.712771277128 0.0598132495154681 2.13989365887819E-4
127.732773277328 0.0598091229987769 2.13945079557699E-4
127.752775277528 0.0598049974753967 2.13900809996349E-4
127.772777277728 0.059800872944939 2.13856557194769E-4
127.792779277928 0.0597967494070084 2.13812321143885E-4
127.812781278128 0.0597926268612128 2.13768101834667E-4
127.832783278328 0.0597885053071596 2.13723899258083E-4
127.852785278528 0.0597843847444552 2.13679713405093E-4
127.872787278728 0.0597802651727072 2.13635544266673E-4
127.892789278928 0.0597761465915227 2.135913918338E-4
127.912791279128 0.0597720290005083 2.13547256097449E-4
127.932793279328 0.0597679123992697 2.13503137048588E-4
127.952795279528 0.0597637967874158 2.13459034678225E-4
127.972797279728 0.0597596821645513 2.13414948977325E-4
127.992799279928 0.0597555685302842 2.13370879936895E-4
128.012801280128 0.0597514558842199 2.13326827547915E-4
128.032803280328 0.0597473442259657 2.13282791801391E-4
128.052805280528 0.05974323355513 2.13238772688345E-4
128.072807280728 0.0597391238713162 2.1319477019975E-4
128.092809280928 0.0597350151741343 2.13150784326651E-4
128.112811281128 0.0597309074631882 2.13106815060035E-4
128.132813281328 0.0597268007380842 2.13062862390916E-4
128.152815281528 0.0597226949984275 2.130189263103E-4
128.172817281728 0.0597185902438266 2.12975006809237E-4
128.192819281928 0.0597144864738913 2.12931103878788E-4
128.212821282128 0.0597103836882205 2.12887217509906E-4
128.232823282328 0.0597062818864241 2.12843347693665E-4
128.252825282528 0.0597021810681079 2.12799494421098E-4
128.272827282728 0.0596980812328802 2.12755657683267E-4
128.292829282928 0.0596939823803419 2.12711837471164E-4
128.312831283128 0.0596898845101025 2.12668033775872E-4
128.332833283328 0.0596857876217673 2.12624246588436E-4
128.352835283528 0.0596816917149422 2.12580475899909E-4
128.372837283728 0.0596775967892345 2.12536721701364E-4
128.392839283928 0.0596735028442479 2.12492983983841E-4

128.412841284128 0.0596694098795885 2.12449262738407E-4
128.432843284328 0.0596653178948643 2.12405557956155E-4
128.452845284528 0.0596612268896782 2.12361869628126E-4
128.472847284728 0.059657136863635 2.12318197745388E-4
128.492849284929 0.0596530478163444 2.12274542299061E-4
128.512851285129 0.0596489597474072 2.12230903280177E-4
128.532853285329 0.0596448726564342 2.12187280679879E-4
128.552855285529 0.0596407865430249 2.1214367448919E-4
128.572857285729 0.0596367014067902 2.12100084699263E-4
128.592859285929 0.0596326172473322 2.12056511301158E-4
128.612861286129 0.0596285340642573 2.12012954285986E-4
128.632863286329 0.0596244518571716 2.11969413644857E-4
128.652865286529 0.0596203706256788 2.11925889368862E-4
128.672867286729 0.0596162903693847 2.11882381449117E-4
128.692869286929 0.0596122110878953 2.11838889876743E-4
128.712871287129 0.0596081327808153 2.11795414642852E-4
128.732873287329 0.0596040554477461 2.11751955738526E-4
128.752875287529 0.0595999790882975 2.11708513154939E-4
128.772877287729 0.059595903702074 2.11665086883212E-4
128.792879287929 0.0595918292886793 2.11621676914461E-4
128.812881288129 0.0595877558477178 2.11578283239817E-4
128.832883288329 0.0595836833787969 2.11534905850442E-4
128.852885288529 0.0595796118815164 2.11491544737424E-4
128.872887288729 0.0595755413554868 2.11448199891968E-4
128.892889288929 0.059571471800312 2.11404871305214E-4
128.912891289129 0.0595674032155912 2.11361558968251E-4
128.932893289329 0.0595633356009341 2.1131826287229E-4
128.952895289529 0.0595592689559455 2.1127498300849E-4
128.972897289729 0.0595552032802269 2.1123171936798E-4
128.992899289929 0.0595511385733847 2.11188471941944E-4
129.012901290129 0.0595470748350241 2.11145240721558E-4
129.032903290329 0.0595430120647459 2.11102025697955E-4
129.052905290529 0.0595389502621588 2.11058826862357E-4
129.072907290729 0.0595348894268643 2.1101564420591E-4
129.092909290929 0.0595308295584705 2.10972477719837E-4
129.112911291129 0.0595267706565763 2.10929327395266E-4
129.132913291329 0.059522712720789 2.10886193223418E-4
129.152915291529 0.0595186557507115 2.10843075195473E-4
129.172917291729 0.05951459974595 2.10799973302646E-4
129.192919291929 0.0595105447061067 2.10756887536118E-4
129.212921292129 0.0595064906307861 2.10713817887094E-4
129.232923292329 0.0595024375195949 2.1067076434681E-4
129.252925292529 0.0594983853721308 2.10627726906407E-4
129.272927292729 0.0594943341880035 2.10584705557161E-4

129.292929292929 0.0594902839668143 2.10541700290258E-4
129.312931293129 0.0594862347081685 2.10498711096934E-4
129.332933293329 0.0594821864116662 2.10455737968372E-4
129.352935293529 0.0594781390769149 2.1041278089584E-4
129.372937293729 0.05947409270352 2.10369839870582E-4
129.392939293929 0.0594700472910774 2.1032691488375E-4
129.412941294129 0.0594660028391979 2.10284005926656E-4
129.432943294329 0.0594619593474844 2.10241112990533E-4
129.452945294529 0.0594579168155362 2.1019823606658E-4
129.472947294729 0.0594538752429616 2.10155375146093E-4
129.49294929493 0.0594498346293585 2.10112530220262E-4
129.51295129513 0.0594457949743377 2.10069701280421E-4
129.53295329533 0.0594417562774953 2.10026888317748E-4
129.55295529553 0.0594377185384388 2.09984091323546E-4
129.57295729573 0.0594336817567699 2.09941310289065E-4
129.59295929593 0.0594296459320953 2.09898545205605E-4
129.61296129613 0.0594256110640111 2.09855796064361E-4
129.63296329633 0.0594215771521288 2.09813062856698E-4
129.65296529653 0.0594175441960448 2.09770345573819E-4
129.67296729673 0.0594135121953651 2.09727644207036E-4
129.69296929693 0.0594094811496918 2.09684958747622E-4
129.71297129713 0.0594054510586306 2.09642289186893E-4
129.73297329733 0.0594014219217804 2.09599635516098E-4
129.75297529753 0.0593973937387463 2.09556997726552E-4
129.77297729773 0.0593933665091311 2.09514375809552E-4
129.79297929793 0.0593893402325367 2.09471769756389E-4
129.81298129813 0.0593853149085679 2.09429179558388E-4
129.83298329833 0.0593812905368253 2.09386605206833E-4
129.85298529853 0.0593772671169129 2.09344046693048E-4
129.87298729873 0.0593732446484333 2.09301504008348E-4
129.89298929893 0.0593692231309888 2.09258977144046E-4
129.91299129913 0.0593652025641789 2.09216466091431E-4
129.93299329933 0.0593611829476133 2.091739708419E-4
129.95299529953 0.059357164280886 2.09131491386693E-4
129.97299729973 0.0593531465636056 2.09089027717206E-4
129.99299929993 0.0593491297953727 2.09046579824753E-4
130.01300130013 0.059345113975789 2.09004147700663E-4
130.03300330033 0.0593410991044604 2.08961731336317E-4
130.05300530053 0.0593370851809846 2.0891933072301E-4
130.07300730073 0.059333072204965 2.088769458521E-4
130.09300930093 0.0593290601760017 2.08834576714918E-4
130.11301130113 0.0593250490937029 2.08792223302881E-4
130.13301330133 0.0593210389576661 2.08749885607296E-4
130.15301530153 0.0593170297674944 2.08707563619537E-4

130.17301730173 0.0593130215227911 2.08665257330981E-4
130.19301930193 0.0593090142231563 2.08622966732975E-4
130.21302130213 0.0593050078681946 2.08580691816917E-4
130.23302330233 0.0593010024575043 2.08538432574145E-4
130.25302530253 0.0592969979906901 2.08496188996064E-4
130.27302730273 0.0592929944673525 2.08453961074042E-4
130.29302930293 0.059288991887094 2.08411748799472E-4
130.31303130313 0.0592849902495162 2.08369552163738E-4
130.33303330333 0.0592809895542219 2.08327371158242E-4
130.35303530353 0.0592769898008092 2.08285205774342E-4
130.37303730373 0.0592729909888847 2.08243056003487E-4
130.39303930393 0.0592689931180468 2.08200921837045E-4
130.41304130413 0.0592649961878973 2.08158803266423E-4
130.43304330433 0.0592610001980407 2.08116700283058E-4
130.45304530453 0.059257005148077 2.08074612878348E-4
130.47304730473 0.0592530110376022 2.08032541043647E-4
130.493049304931 0.0592490178662246 2.07990484770448E-4
130.513051305131 0.0592450256335455 2.07948444050171E-4
130.533053305331 0.0592410343391619 2.07906418874193E-4
130.553055305531 0.0592370439826786 2.0786440923398E-4
130.573057305731 0.0592330545636935 2.07822415120926E-4
130.593059305931 0.0592290660818148 2.07780436526541E-4
130.613061306131 0.0592250785366348 2.07738473442168E-4
130.633063306331 0.0592210919277604 2.0769652585931E-4
130.653065306531 0.059217106254792 2.07654593769403E-4
130.673067306731 0.0592131215173299 2.07612677163885E-4
130.693069306931 0.0592091377149747 2.07570776034205E-4
130.713071307131 0.0592051548473279 2.07528890371822E-4
130.733073307331 0.0592011729139884 2.07487020168172E-4
130.753075307531 0.0591971919145621 2.07445165414768E-4
130.773077307731 0.0591932118486473 2.07403326103047E-4
130.793079307931 0.0591892327158441 2.0736150222447E-4
130.813081308131 0.0591852545157547 2.07319693770522E-4
130.833083308331 0.059181277247977 2.07277900732648E-4
130.853085308531 0.0591773009121175 2.07236123102384E-4
130.873087308731 0.0591733255077697 2.07194360871139E-4
130.893089308931 0.0591693510345408 2.07152614030463E-4
130.913091309131 0.0591653774920287 2.07110882571814E-4
130.933093309331 0.0591614048798325 2.07069166486672E-4
130.953095309531 0.0591574331975563 2.07027465766565E-4
130.973097309731 0.0591534624447992 2.06985780402977E-4
130.993099309931 0.0591494926211584 2.06944110387374E-4
131.013101310131 0.0591455237262394 2.06902455711314E-4
131.033103310331 0.0591415557596411 2.06860816366288E-4

131.053105310531 0.0591375887209626 2.06819192343793E-4
131.073107310731 0.0591336226098035 2.06777583635338E-4
131.093109310931 0.059129657425769 2.06735990232489E-4
131.113111311131 0.0591256931684533 2.06694412126705E-4
131.133113311331 0.0591217298374632 2.06652849309578E-4
131.153115311531 0.0591177674323923 2.06611301772569E-4
131.173117311731 0.0591138059528448 2.06569769507247E-4
131.193119311931 0.0591098453984187 2.06528252505127E-4
131.213121312131 0.0591058857687197 2.06486750757804E-4
131.233123312331 0.0591019270633406 2.06445264256744E-4
131.253125312531 0.0590979692818852 2.06403792993529E-4
131.273127312731 0.0590940124239535 2.06362336959706E-4
131.293129312931 0.0590900564891427 2.063208961468E-4
131.313131313131 0.0590861014770588 2.06279470546425E-4
131.333133313331 0.0590821473872955 2.06238060150077E-4
131.353135313531 0.0590781942194569 2.06196664949359E-4
131.373137313731 0.059074241973142 2.06155284935826E-4
131.393139313931 0.0590702906479492 2.06113920101032E-4
131.413141314131 0.0590663402434771 2.06072570436535E-4
131.433143314331 0.0590623907593292 2.0603123593395E-4
131.453145314531 0.0590584421951049 2.05989916584849E-4
131.473147314731 0.0590544945503997 2.05948612380771E-4
131.493149314931 0.0590505478248199 2.05907323313368E-4
131.513151315132 0.059046602017959 2.05866049374163E-4
131.533153315332 0.0590426571294228 2.05824790554814E-4
131.553155315532 0.059038713158803 2.05783546846833E-4
131.573157315732 0.0590347701057046 2.05742318241873E-4
131.593159315932 0.0590308279697272 2.05701104731537E-4
131.613161316132 0.0590268867504708 2.05659906307434E-4
131.633163316332 0.0590229464475303 2.05618722961124E-4
131.653165316532 0.0590190070605084 2.05577554684253E-4
131.673167316732 0.0590150685890062 2.05536401468454E-4
131.693169316932 0.0590111310326219 2.0549526330533E-4
131.713171317132 0.0590071943909527 2.05454140186481E-4
131.733173317332 0.0590032586636004 2.05413032103556E-4
131.753175317532 0.0589993238501619 2.05371939048161E-4
131.773177317732 0.0589953899502395 2.05330861011956E-4
131.793179317932 0.0589914569634289 2.0528979798654E-4
131.813181318132 0.0589875248893343 2.05248749963601E-4
131.833183318332 0.0589835937275519 2.05207716934751E-4
131.853185318532 0.0589796634776796 2.05166698891621E-4
131.873187318732 0.058975734139319 2.05125695825883E-4
131.893189318932 0.0589718057120673 2.0508470772917E-4
131.913191319132 0.058967878195524 2.0504373459314E-4

131.933193319332 0.0589639515892897 2.05002776409469E-4
131.953195319532 0.0589600258929646 2.04961833169828E-4
131.973197319732 0.0589561011061417 2.0492090486582E-4
131.993199319932 0.0589521772284256 2.04879991489171E-4
132.013201320132 0.0589482542594124 2.04839093031524E-4
132.033203320332 0.0589443321987045 2.04798209484588E-4
132.053205320532 0.058940411045897 2.04757340840003E-4
132.073207320732 0.0589364908005882 2.04716487089442E-4
132.093209320932 0.0589325714623792 2.04675648224615E-4
132.113211321132 0.0589286530308709 2.04634824237231E-4
132.133213321332 0.0589247355056606 2.04594015118967E-4
132.153215321532 0.0589208188863452 2.04553220861495E-4
132.173217321732 0.0589169031725214 2.04512441456494E-4
132.193219321932 0.058912988363794 2.04471676895729E-4
132.213221322132 0.0589090744597572 2.04430927170857E-4
132.233223322332 0.058905161460012 2.04390192273609E-4
132.253225322532 0.0589012493641576 2.04349472195702E-4
132.273227322732 0.0588973381717879 2.04308766928804E-4
132.293229322932 0.0588934278825089 2.04268076464707E-4
132.313231323132 0.0588895184959125 2.04227400795065E-4
132.333233323332 0.058885610011601 2.0418673991164E-4
132.353235323532 0.0588817024291708 2.04146093806139E-4
132.373237323732 0.0588777957482191 2.04105462470279E-4
132.393239323932 0.0588738899683487 2.04064845895839E-4
132.413241324132 0.0588699850891583 2.04024244074561E-4
132.433243324332 0.0588660811102421 2.0398365699814E-4
132.453245324532 0.0588621780311989 2.03943084658326E-4
132.473247324732 0.0588582758516329 2.03902527046927E-4
132.493249324932 0.0588543745711331 2.03861984155598E-4
132.513251325133 0.0588504741893059 2.03821455976175E-4
132.533253325333 0.0588465747057456 2.03780942500376E-4
132.553255325533 0.0588426761200527 2.03740443719985E-4
132.573257325733 0.0588387784318246 2.03699959626755E-4
132.593259325933 0.0588348816406572 2.03659490212433E-4
132.613261326133 0.0588309857461519 2.0361903546882E-4
132.633263326333 0.0588270907479072 2.03578595387696E-4
132.653265326533 0.0588231966455163 2.03538169960785E-4
132.673267326733 0.0588193034385833 2.0349775917993E-4
132.693269326933 0.0588154111267024 2.03457363036877E-4
132.713271327133 0.058811519709474 2.03416981523433E-4
132.733273327333 0.0588076291864951 2.03376614631382E-4
132.753275327533 0.0588037395573679 2.0333626235256E-4
132.773277327733 0.0587998508216826 2.0329592467868E-4
132.793279327933 0.0587959629790411 2.03255601601588E-4

132.813281328133 0.058792076029045 2.03215293113122E-4
132.833283328333 0.0587881899712863 2.03174999205029E-4
132.853285328533 0.058784304805366 2.03134719869151E-4
132.873287328733 0.0587804205308813 2.03094455097294E-4
132.893289328933 0.0587765371474339 2.03054204881316E-4
132.913291329133 0.0587726546546139 2.03013969212957E-4
132.933293329333 0.0587687730520261 2.02973748084111E-4
132.953295329533 0.058764892339265 2.02933541486573E-4
132.973297329733 0.0587610125159329 2.02893349412218E-4
132.993299329933 0.0587571335816205 2.02853171852807E-4
133.013301330133 0.0587532555359307 2.0281300880023E-4
133.033303330333 0.0587493783784574 2.02772860246288E-4
133.053305330533 0.0587455021088041 2.02732726182885E-4
133.073307330733 0.0587416267265655 2.02692606601835E-4
133.093309330933 0.0587377522313385 2.02652501494982E-4
133.113311331133 0.0587338786227207 2.02612410854178E-4
133.133313331333 0.0587300059003127 2.0257233467131E-4
133.153315331533 0.0587261340637079 2.02532272938195E-4
133.173317331733 0.0587222631125103 2.02492225646764E-4
133.193319331933 0.05871839304631 2.02452192788803E-4
133.213321332133 0.0587145238647108 2.0241217435625E-4
133.233323332333 0.058710655567307 2.02372170340943E-4
133.253325332533 0.0587067881536959 2.02332180734756E-4
133.273327332733 0.0587029216234794 2.02292205529614E-4
133.293329332933 0.0586990559762515 2.02252244717366E-4
133.313331333133 0.0586951912116076 2.02212298289876E-4
133.333333333333 0.058691327329152 2.02172366239105E-4
133.353335333533 0.0586874643284747 2.02132448556869E-4
133.373337333733 0.0586836022091813 2.02092545235149E-4
133.393339333933 0.0586797409708602 2.02052656265752E-4
133.413341334133 0.0586758806131174 2.0201278164067E-4
133.433343334333 0.0586720211355429 2.01972921351732E-4
133.453345334533 0.0586681625377422 2.01933075390935E-4
133.473347334733 0.0586643048193064 2.01893243750124E-4
133.493349334934 0.0586604479798361 2.01853426421252E-4
133.513351335134 0.0586565920189281 2.01813623396231E-4
133.533353335334 0.0586527369361776 2.01773834666965E-4
133.553355335534 0.0586488827311878 2.01734060225439E-4
133.573357335734 0.0586450294035464 2.01694300063485E-4
133.593359335934 0.058641176952863 2.01654554173164E-4
133.613361336134 0.0586373253787248 2.01614822546308E-4
133.633363336334 0.0586334746807376 2.01575105174945E-4
133.653365336534 0.0586296248584916 2.01535402050945E-4
133.673367336734 0.0586257759115878 2.01495713166295E-4

133.693369336934 0.0586219278396216 2.01456038512925E-4
133.713371337134 0.0586180806421925 2.01416378082812E-4
133.733373337334 0.0586142343188988 2.01376731867925E-4
133.753375337534 0.0586103888693313 2.01337099860157E-4
133.773377337734 0.0586065442930951 2.01297482051554E-4
133.793379337934 0.0586027005897829 2.01257878434034E-4
133.813381338134 0.0585988577589944 2.01218288999597E-4
133.833383338334 0.058595015800324 2.01178713740185E-4
133.853385338534 0.0585911747133753 2.01139152647842E-4
133.873387338734 0.0585873344977399 2.01099605714493E-4
133.893389338934 0.0585834951530152 2.01060072932121E-4
133.913391339134 0.058579656678799 2.01020554292723E-4
133.933393339334 0.0585758190746897 2.009810497883E-4
133.953395339534 0.0585719823402872 2.00941559410875E-4
133.973397339734 0.0585681464751821 2.00902083152379E-4
133.993399339934 0.0585643114789771 2.00862621004868E-4
134.013401340134 0.0585604773512677 2.00823172960332E-4
134.033403340334 0.0585566440916496 2.00783739010762E-4
134.053405340534 0.0585528116997228 2.00744319148198E-4
134.073407340734 0.0585489801750827 2.00704913364637E-4
134.093409340934 0.0585451495173277 2.0066552165211E-4
134.113411341134 0.0585413197260521 2.00626144002609E-4
134.133413341334 0.0585374908008572 2.00586780408203E-4
134.153415341534 0.0585336627413409 2.00547430860925E-4
134.173417341734 0.058529835547095 2.00508095352753E-4
134.193419341934 0.0585260092177191 2.00468773875748E-4
134.213421342134 0.0585221837528109 2.00429466421953E-4
134.233423342334 0.0585183591519681 2.00390172983416E-4
134.253425342534 0.0585145354147882 2.00350893552186E-4
134.273427342734 0.058510712540866 2.00311628120288E-4
134.293429342934 0.0585068905298012 2.00272376679798E-4
134.313431343134 0.0585030693811899 2.00233139222764E-4
134.333433343334 0.058499249094629 2.00193915741239E-4
134.353435343534 0.0584954296697139 2.00154706227269E-4
134.373437343734 0.0584916111060468 2.00115510672972E-4
134.393439343934 0.0584877934032237 2.00076329070405E-4
134.413441344134 0.0584839765608371 2.00037161411591E-4
134.433443344334 0.0584801605784872 1.99998007688639E-4
134.453445344534 0.058476345455772 1.99958867893636E-4
134.473447344734 0.058472531192287 1.99919742018649E-4
134.493449344935 0.0584687177876292 1.9988063005576E-4
134.513451345135 0.0584649052414004 1.99841531997109E-4
134.533453345335 0.0584610935531904 1.99802447834711E-4
134.553455345535 0.0584572827226009 1.99763377560709E-4

134.573457345735 0.0584534727492289 1.99724321167203E-4
134.593459345935 0.0584496636326693 1.99685278646271E-4
134.613461346135 0.0584458553725225 1.99646249990053E-4
134.633463346335 0.0584420479683841 1.99607235190643E-4
134.653465346535 0.0584382414198518 1.99568234240158E-4
134.673467346735 0.0584344357265207 1.99529247130696E-4
134.693469346935 0.0584306308879887 1.99490273854383E-4
134.713471347135 0.0584268269038553 1.99451314403366E-4
134.733473347335 0.0584230237737141 1.99412368769736E-4
134.753475347535 0.0584192214971685 1.99373436945685E-4
134.773477347735 0.0584154200738073 1.99334518923263E-4
134.793479347935 0.058411619503236 1.99295614694691E-4
134.813481348135 0.0584078197850429 1.99256724252017E-4
134.833483348335 0.0584040209188326 1.99217847587462E-4
134.853485348535 0.0584002229041992 1.99178984693142E-4
134.873487348735 0.0583964257407435 1.99140135561242E-4
134.893489348935 0.0583926294280571 1.99101300183861E-4
134.913491349135 0.0583888339657398 1.9906247855318E-4
134.933493349335 0.0583850393533888 1.99023670661362E-4
134.953495349535 0.0583812455906017 1.98984876500575E-4
134.973497349735 0.0583774526769753 1.98946096062979E-4
134.993499349935 0.0583736606121081 1.98907329340761E-4
135.013501350135 0.0583698693955954 1.98868576326073E-4
135.033503350335 0.0583660790270362 1.98829837011113E-4
135.053505350535 0.0583622895060255 1.98791111388037E-4
135.073507350735 0.0583585008321624 1.9875239944905E-4
135.093509350935 0.058354713005045 1.98713701186346E-4
135.113511351135 0.0583509260242695 1.98675016592108E-4
135.133513351335 0.0583471398894307 1.98636345658503E-4
135.153515351535 0.0583433546001297 1.98597688377769E-4
135.173517351735 0.0583395701559653 1.98559044742123E-4
135.193519351935 0.0583357865565289 1.9852041474371E-4
135.213521352135 0.058332003801421 1.98481798374769E-4
135.233523352335 0.0583282218902415 1.98443195627539E-4
135.253525352535 0.0583244408225857 1.98404606494216E-4
135.273527352735 0.0583206605980477 1.98366030966983E-4
135.293529352935 0.0583168812162264 1.98327469038078E-4
135.313531353135 0.0583131026767229 1.98288920699766E-4
135.333533353335 0.0583093249791341 1.98250385944269E-4
135.353535353535 0.0583055481230537 1.98211864763782E-4
135.373537353735 0.0583017721080793 1.98173357150544E-4
135.393539353935 0.0582979969338134 1.98134863096846E-4
135.413541354135 0.0582942225998468 1.98096382594861E-4
135.433543354335 0.058290449105783 1.98057915636898E-4

135.453545354535 0.0582866764512161 1.98019462215171E-4
135.473547354735 0.0582829046357439 1.97981022321938E-4
135.493549354936 0.058279133658964 1.97942595949456E-4
135.513551355136 0.0582753635204746 1.9790418308999E-4
135.533553355336 0.0582715942198735 1.97865783735808E-4
135.553555355536 0.0582678257567583 1.97827397879178E-4
135.573557355736 0.0582640581307247 1.97789025512349E-4
135.593559355936 0.0582602913413738 1.97750666627631E-4
135.613561356136 0.0582565253882963 1.97712321217232E-4
135.633563356336 0.0582527602711002 1.97673989273538E-4
135.653565356536 0.0582489959893742 1.97635670788742E-4
135.673567356736 0.0582452325427215 1.97597365755187E-4
135.693569356936 0.0582414699307339 1.97559074165105E-4
135.713571357136 0.0582377081530134 1.97520796010835E-4
135.733573357336 0.0582339472091591 1.97482531284689E-4
135.753575357536 0.0582301870987662 1.97444279978941E-4
135.773577357736 0.0582264278214336 1.97406042085909E-4
135.793579357936 0.0582226693767572 1.97367817597883E-4
135.813581358136 0.0582189117643379 1.97329606507205E-4
135.833583358336 0.0582151549837699 1.97291408806154E-4
135.853585358536 0.0582113990346527 1.97253224487065E-4
135.873587358736 0.0582076439165861 1.97215053542282E-4
135.893589358936 0.0582038896291623 1.97176895964071E-4
135.913591359136 0.0582001361719847 1.97138751744818E-4
135.933593359336 0.0581963835446517 1.9710062087686E-4
135.953595359536 0.0581926317467597 1.97062503352519E-4
135.973597359736 0.0581888807779036 1.97024399164101E-4
135.993599359936 0.0581851306376826 1.96986308303964E-4
136.013601360136 0.0581813813256975 1.96948230764481E-4
136.033603360336 0.0581776328415437 1.96910166537976E-4
136.053605360536 0.058173885184819 1.968721156168E-4
136.073607360736 0.0581701383551253 1.96834077993349E-4
136.093609360936 0.0581663923520581 1.96796053659957E-4
136.113611361136 0.0581626471752143 1.96758042608975E-4
136.133613361336 0.0581589028241937 1.96720044832787E-4
136.153615361536 0.0581551592985919 1.96682060323739E-4
136.173617361736 0.0581514165980128 1.96644089074262E-4
136.193619361936 0.0581476747220476 1.96606131076663E-4
136.213621362136 0.0581439336703002 1.96568186323381E-4
136.233623362336 0.0581401934423675 1.96530254806786E-4
136.253625362536 0.0581364540378448 1.96492336519236E-4
136.273627362736 0.0581327154563339 1.9645443145316E-4
136.293629362936 0.0581289776974285 1.96416539600904E-4
136.313631363136 0.0581252407607351 1.96378660954949E-4

136.333633363336 0.0581215046458439 1.96340795507615E-4
136.353635363536 0.0581177693523579 1.96302943251354E-4
136.373637363736 0.0581140348798746 1.96265104178564E-4
136.393639363936 0.0581103012279896 1.96227278281631E-4
136.413641364136 0.0581065683963056 1.96189465553012E-4
136.433643364336 0.0581028363844224 1.9615166598514E-4
136.453645364536 0.0580991051919319 1.9611387957037E-4
136.473647364736 0.0580953748184358 1.9607610630116E-4
136.493649364936 0.0580916452635349 1.96038346169963E-4
136.513651365137 0.0580879165268267 1.96000599169199E-4
136.533653365337 0.0580841886079118 1.95962865291327E-4
136.553655365537 0.0580804615063835 1.95925144528732E-4
136.573657365737 0.0580767352218443 1.95887436873895E-4
136.593659365937 0.058073009753892 1.95849742319255E-4
136.613661366137 0.0580692851021302 1.95812060857312E-4
136.633663366337 0.0580655612661468 1.95774392480407E-4
136.653665366537 0.058061838245549 1.95736737181086E-4
136.673667366737 0.0580581160399329 1.95699094951781E-4
136.693669366937 0.0580543946489038 1.95661465785022E-4
136.713671367137 0.0580506740720536 1.95623849673204E-4
136.733673367337 0.0580469543089791 1.95586246608779E-4
136.753675367537 0.0580432353592876 1.95548656584305E-4
136.773677367737 0.0580395172225731 1.95511079592211E-4
136.793679367937 0.058035799898436 1.95473515624994E-4
136.813681368137 0.0580320833864705 1.95435964675086E-4
136.833683368337 0.0580283676862855 1.95398426735081E-4
136.853685368537 0.0580246527974731 1.95360901797395E-4
136.873687368737 0.058020938719635 1.95323389854553E-4
136.893689368937 0.058017225452369 1.9528589089904E-4
136.913691369137 0.0580135129952758 1.95248404923372E-4
136.933693369337 0.0580098013479564 1.95210931920075E-4
136.953695369537 0.0580060905100057 1.95173471881614E-4
136.973697369737 0.058002380481027 1.95136024800542E-4
136.993699369937 0.0579986712606162 1.95098590669342E-4
137.013701370137 0.0579949628483764 1.95061169480571E-4
137.033703370337 0.0579912552439066 1.95023761226748E-4
137.053705370537 0.0579875484468068 1.94986365900408E-4
137.073707370737 0.0579838424566733 1.94948983494049E-4
137.093709370937 0.0579801372731073 1.94911614000221E-4
137.113711371137 0.0579764328957096 1.94874257411474E-4
137.133713371337 0.0579727293240824 1.94836913720378E-4
137.153715371537 0.0579690265578206 1.94799582919428E-4
137.173717371737 0.0579653245965273 1.94762265001208E-4
137.193719371937 0.057961623439797 1.94724959958217E-4

137.213721372137 0.0579579230872401 1.94687667783118E-4
137.233723372337 0.0579542235384461 1.94650388468366E-4
137.253725372537 0.0579505247930205 1.9461312200658E-4
137.273727372737 0.0579468268505605 1.945758683903E-4
137.293729372937 0.0579431297106702 1.94538627612139E-4
137.313731373137 0.0579394333729462 1.94501399664634E-4
137.333733373337 0.0579357378369902 1.9446418454038E-4
137.353735373537 0.0579320431024017 1.94426982231952E-4
137.373737373737 0.0579283491687811 1.94389792731936E-4
137.393739373937 0.0579246560357313 1.94352616032948E-4
137.413741374137 0.0579209637028479 1.94315452127533E-4
137.433743374337 0.0579172721697325 1.94278301008299E-4
137.453745374537 0.0579135814359906 1.94241162667897E-4
137.473747374737 0.0579098915012146 1.94204037098848E-4
137.493749374937 0.0579062023650143 1.94166924293854E-4
137.513751375138 0.0579025140269853 1.94129824245475E-4
137.533753375338 0.0578988264867243 1.94092736946284E-4
137.553755375538 0.0578951397438399 1.94055662388981E-4
137.573757375738 0.0578914537979257 1.94018600566114E-4
137.593759375938 0.0578877686485881 1.93981551470364E-4
137.613761376138 0.0578840842954269 1.93944515094351E-4
137.633763376338 0.0578804007380406 1.93907491430682E-4
137.653765376538 0.0578767179760319 1.9387048047201E-4
137.673767376738 0.0578730360090002 1.93833482210961E-4
137.693769376938 0.0578693548365485 1.93796496640195E-4
137.713771377138 0.0578656744582754 1.93759523752334E-4
137.733773377338 0.0578619948737863 1.93722563540072E-4
137.753775377538 0.0578583160826822 1.93685615996061E-4
137.773777377738 0.0578546380845573 1.93648681112886E-4
137.793779377938 0.0578509608790186 1.93611758883268E-4
137.813781378138 0.0578472844656689 1.93574849299883E-4
137.833783378338 0.0578436088441049 1.93537952355355E-4
137.853785378538 0.057839934013932 1.93501068042393E-4
137.873787378738 0.0578362599747464 1.93464196353622E-4
137.893789378938 0.0578325867261573 1.93427337281794E-4
137.913791379138 0.0578289142677619 1.93390490819551E-4
137.933793379338 0.0578252425991634 1.93353656959591E-4
137.953795379538 0.0578215717199609 1.93316835694581E-4
137.973797379738 0.0578179016297585 1.93280027017236E-4
137.993799379938 0.0578142323281579 1.93243230920251E-4
138.013801380138 0.057810563814762 1.93206447396339E-4
138.033803380338 0.057806896089171 1.93169676438184E-4
138.053805380538 0.0578032291509856 1.93132918038484E-4
138.073807380738 0.0577995629998108 1.93096172189978E-4

138.093809380938 0.0577958976352487 1.93059438885381E-4
138.113811381138 0.0577922330568979 1.93022718117377E-4
138.133813381338 0.0577885692643675 1.92986009878757E-4
138.153815381538 0.0577849062572506 1.92949314162154E-4
138.173817381738 0.0577812440351573 1.92912630960377E-4
138.193819381938 0.0577775825976885 1.92875960266143E-4
138.213821382138 0.0577739219444467 1.92839302072189E-4
138.233823382338 0.0577702620750289 1.92802656371201E-4
138.253825382538 0.0577666029890442 1.92766023155988E-4
138.273827382738 0.0577629446860938 1.92729402419285E-4
138.293829382938 0.0577592871657786 1.92692794153823E-4
138.313831383138 0.0577556304277062 1.92656198352406E-4
138.333833383338 0.0577519744714726 1.92619615007726E-4
138.353835383538 0.0577483192966861 1.92583044112599E-4
138.373837383738 0.0577446649029481 1.92546485659773E-4
138.393839383938 0.0577410112898613 1.92509939642018E-4
138.413841384138 0.0577373584570271 1.92473406052088E-4
138.433843384338 0.0577337064040518 1.92436884882793E-4
138.453845384538 0.0577300551305363 1.92400376126891E-4
138.473847384738 0.0577264046360864 1.9236387977719E-4
138.493849384939 0.0577227549203057 1.92327395826481E-4
138.513851385139 0.0577191059827964 1.92290924267545E-4
138.533853385339 0.057715457823161 1.92254465093166E-4
138.553855385539 0.0577118104410015 1.92218018296129E-4
138.573857385739 0.0577081638359274 1.92181583869295E-4
138.593859385939 0.0577045180075378 1.92145161805424E-4
138.613861386139 0.0577008729554376 1.92108752097338E-4
138.633863386339 0.0576972286792312 1.92072354737858E-4
138.653865386539 0.0576935851785216 1.9203596971979E-4
138.673867386739 0.0576899424529138 1.91999597035968E-4
138.693869386939 0.057686300502012 1.91963236679217E-4
138.713871387139 0.0576826593254216 1.91926888642381E-4
138.733873387339 0.0576790189227436 1.91890552918257E-4
138.753875387539 0.0576753792935841 1.91854229499702E-4
138.773877387739 0.0576717404375466 1.91817918379547E-4
138.793879387939 0.0576681023542374 1.91781619550655E-4
138.813881388139 0.0576644650432573 1.91745333005835E-4
138.833883388339 0.0576608285042158 1.9170905873799E-4
138.853885388539 0.0576571927367154 1.91672796739951E-4
138.873887388739 0.0576535577403611 1.9163654700458E-4
138.893889388939 0.0576499235147568 1.91600309524732E-4
138.913891389139 0.057646290059506 1.91564084293257E-4
138.933893389339 0.0576426573742167 1.91527871303057E-4
138.953895389539 0.0576390254584932 1.91491670546998E-4

139.973897389739 0.0576353943119392 1.91455482017943E-4
138.993899389939 0.0576317639341652 1.91419305708826E-4
139.013901390139 0.057628134324768 1.91383141612448E-4
139.033903390339 0.0576245054833576 1.91346989721743E-4
139.053905390539 0.0576208774095403 1.91310850029613E-4
139.073907390739 0.057617250102919 1.91274722528928E-4
139.093909390939 0.0576136235631012 1.91238607212607E-4
139.113911391139 0.0576099977896919 1.91202504073546E-4
139.133913391339 0.0576063727822975 1.91166413104659E-4
139.153915391539 0.0576027485405199 1.9113033429882E-4
139.173917391739 0.057599125063974 1.91094267649032E-4
139.193919391939 0.0575955023522565 1.91058213148119E-4
139.213921392139 0.0575918804049793 1.91022170789065E-4
139.233923392339 0.0575882592217434 1.90986140564743E-4
139.253925392539 0.0575846388021575 1.90950122468107E-4
139.273927392739 0.0575810191458311 1.90914116492124E-4
139.293929392939 0.0575774002523658 1.90878122629681E-4
139.313931393139 0.0575737821213709 1.90842140873751E-4
139.333933393339 0.0575701647524542 1.90806171217287E-4
139.353935393539 0.0575665481452179 1.90770213653195E-4
139.373937393739 0.0575629322992691 1.90734268174432E-4
139.393939393939 0.0575593172142201 1.90698334774008E-4
139.413941394139 0.0575557028896741 1.90662413444847E-4
139.433943394339 0.0575520893252357 1.90626504179886E-4
139.453945394539 0.0575484765205149 1.90590606972126E-4
139.473947394739 0.0575448644751211 1.90554721814556E-4
139.49394939494 0.0575412531886584 1.90518848700123E-4
139.51395139514 0.0575376426607335 1.90482987621798E-4
139.53395339534 0.0575340328909538 1.90447138572564E-4
139.55395539554 0.0575304238789303 1.90411301545441E-4
139.57395739574 0.0575268156242666 1.9037547653338E-4
139.59395939594 0.0575232081265715 1.90339663529388E-4
139.61396139614 0.0575196013854562 1.90303862526494E-4
139.63396339634 0.057515995400523 1.90268073517644E-4
139.65396539654 0.0575123901713821 1.90232296495868E-4
139.67396739674 0.0575087856976426 1.90196531454184E-4
139.69396939694 0.0575051819789121 1.901607783856E-4
139.71397139714 0.0575015790148009 1.90125037283153E-4
139.73397339734 0.0574979768049121 1.90089308139811E-4
139.75397539754 0.0574943753488552 1.90053590948611E-4
139.77397739774 0.0574907746462444 1.90017885702635E-4
139.79397939794 0.0574871746966835 1.8998219239487E-4
139.81398139814 0.0574835754997831 1.89946511018368E-4
139.83398339834 0.0574799770551475 1.89910841566127E-4

139.85398539854 0.0574763793623906 1.89875184031238E-4
139.87398739874 0.0574727824211209 1.89839538406744E-4
139.89398939894 0.0574691862309441 1.89803904685662E-4
139.91399139914 0.0574655907914738 1.89768282861092E-4
139.93399339934 0.0574619961023143 1.89732672926042E-4
139.95399539954 0.0574584021630815 1.8969707487364E-4
139.97399739974 0.0574548089733781 1.89661488696886E-4
139.99399939994 0.0574512165328161 1.89625914388875E-4
140.01400140014 0.0574476248410058 1.89590351942684E-4
140.03400340034 0.057444033897558 1.89554801351403E-4
140.05400540054 0.057440443702081 1.895192626081E-4
140.07400740074 0.0574368542541827 1.89483735705837E-4
140.09400940094 0.0574332655534787 1.89448220637762E-4
140.11401140114 0.0574296775995744 1.89412717396918E-4
140.13401340134 0.0574260903920801 1.89377225976404E-4
140.15401540154 0.0574225039306094 1.89341746369352E-4
140.17401740174 0.0574189182147701 1.89306278568841E-4
140.19401940194 0.0574153332441712 1.89270822567964E-4
140.21402140214 0.0574117490184271 1.89235378359871E-4
140.23402340234 0.0574081655371482 1.89199945937674E-4
140.25402540254 0.0574045827999417 1.89164525294458E-4
140.27402740274 0.0574010008064223 1.89129116423382E-4
140.29402940294 0.0573974195561983 1.89093719317549E-4
140.31403140314 0.0573938390488845 1.89058333970125E-4
140.33403340334 0.0573902592840876 1.890229603742E-4
140.35403540354 0.057386680261422 1.88987598522943E-4
140.37403740374 0.0573831019804972 1.8895224840948E-4
140.39403940394 0.0573795244409286 1.88916910026993E-4
140.41404140414 0.0573759476423226 1.88881583368582E-4
140.43404340434 0.0573723715842954 1.88846268427445E-4
140.45404540454 0.0573687962664552 1.88810965196704E-4
140.47404740474 0.057365221688416 1.88775673669542E-4
140.494049404941 0.0573616478497885 1.8874039383911E-4
140.514051405141 0.0573580747501876 1.88705125698606E-4
140.534053405341 0.0573545023892251 1.88669869241199E-4
140.554055405541 0.0573509307665122 1.88634624460055E-4
140.574057405741 0.0573473598816616 1.88599391348356E-4
140.594059405941 0.0573437897342845 1.88564169899274E-4
140.614061406141 0.0573402203239968 1.8852896010603E-4
140.634063406341 0.0573366516504091 1.88493761961795E-4
140.654065406541 0.0573330837131325 1.88458575459748E-4
140.674067406741 0.0573295165117849 1.88423400593136E-4
140.694069406941 0.0573259500459778 1.88388237355151E-4
140.714071407141 0.0573223843153238 1.88353085738991E-4

140.734073407341 0.0573188193194362 1.88317945737871E-4
140.754075407541 0.057315255057927 1.8828281734499E-4
140.774077407741 0.0573116915304144 1.88247700553616E-4
140.794079407941 0.057308128736508 1.88212595356931E-4
140.814081408141 0.0573045666758226 1.88177501748173E-4
140.834083408341 0.0573010053479737 1.88142419720591E-4
140.854085408541 0.0572974447525718 1.88107349267382E-4
140.874087408741 0.0572938848892365 1.88072290381843E-4
140.894089408941 0.0572903257575783 1.88037243057179E-4
140.914091409141 0.0572867673572136 1.88002207286658E-4
140.934093409341 0.0572832096877535 1.87967183063497E-4
140.954095409541 0.0572796527488166 1.87932170380989E-4
140.974097409741 0.0572760965400181 1.87897169232402E-4
140.994099409941 0.0572725410609694 1.87862179610961E-4
141.014101410141 0.0572689863112869 1.87827201509949E-4
141.034103410341 0.0572654322905897 1.87792234922676E-4
141.054105410541 0.0572618789984853 1.87757279842342E-4
141.074107410741 0.0572583264345963 1.87722336262296E-4
141.094109410941 0.0572547745985336 1.87687404175776E-4
141.114111411141 0.0572512234899148 1.8765248357609E-4
141.134113411341 0.0572476731083567 1.8761757445654E-4
141.154115411541 0.0572441234534738 1.87582676810406E-4
141.174117411741 0.0572405745248836 1.87547790631002E-4
141.194119411941 0.0572370263221993 1.87512915911603E-4
141.214121412141 0.0572334788450379 1.87478052645525E-4
141.234123412341 0.0572299320930182 1.874432008261E-4
141.254125412541 0.057226386065756 1.87408360446638E-4
141.274127412741 0.0572228407628674 1.87373531500454E-4
141.294129412941 0.0572192961839677 1.87338713980853E-4
141.314131413141 0.0572157523286737 1.87303907881165E-4
141.334133413341 0.0572122091966064 1.87269113194759E-4
141.354135413541 0.0572086667873809 1.87234329914949E-4
141.374137413741 0.0572051251006138 1.8719955803507E-4
141.394139413941 0.0572015841359199 1.87164797548436E-4
141.414141414141 0.0571980438929212 1.8713004844844E-4
141.434143414341 0.0571945043712353 1.87095310728434E-4
141.454145414541 0.0571909655704778 1.87060584381748E-4
141.474147414741 0.0571874274902656 1.87025869401734E-4
141.494149414941 0.0571838901302211 1.86991165781795E-4
141.514151415142 0.0571803534899552 1.86956473515229E-4
141.534153415342 0.0571768175690937 1.86921792595479E-4
141.554155415542 0.0571732823672529 1.868871230159E-4
141.574157415742 0.0571697478840508 1.86852464769865E-4
141.594159415942 0.0571662141191006 1.86817817850703E-4

141.614161416142 0.0571626810720337 1.86783182251924E-4
141.634163416342 0.0571591487424579 1.86748557966808E-4
141.654165416542 0.0571556171299965 1.86713944988794E-4
141.674167416742 0.0571520862342681 1.86679343311273E-4
141.694169416942 0.0571485560548952 1.8664475292768E-4
141.714171417142 0.0571450265914927 1.86610173831375E-4
141.734173417342 0.0571414978436819 1.86575606015786E-4
141.754175417542 0.0571379698110834 1.86541049474338E-4
141.774177417742 0.0571344424933156 1.86506504200432E-4
141.794179417942 0.0571309158899998 1.86471970187505E-4
141.814181418142 0.0571273900007569 1.86437447428992E-4
141.834183418342 0.0571238648252063 1.86402935918314E-4
141.854185418542 0.0571203403629685 1.86368435648905E-4
141.874187418742 0.0571168166136613 1.86333946614181E-4
141.894189418942 0.05711329357691 1.86299468807626E-4
141.914191419142 0.0571097712523356 1.86265002222689E-4
141.934193419342 0.0571062496395572 1.86230546852805E-4
141.954195419542 0.0571027287381934 1.86196102691402E-4
141.974197419742 0.0570992085478699 1.86161669731983E-4
141.994199419942 0.0570956890682043 1.86127247967974E-4
142.014201420142 0.057092170298824 1.86092837392899E-4
142.034203420342 0.0570886522393461 1.86058438000186E-4
142.054205420542 0.0570851348893878 1.86024049783262E-4
142.074207420742 0.0570816182485812 1.85989672735708E-4
142.094209420942 0.0570781023165441 1.85955306850964E-4
142.114211421142 0.0570745870929004 1.85920952122533E-4
142.134213421342 0.0570710725772684 1.85886608543867E-4
142.154215421542 0.0570675587692712 1.85852276108466E-4
142.174217421742 0.0570640456685396 1.8581795480991E-4
142.194219421942 0.0570605332746853 1.85783644641594E-4
142.214221422142 0.0570570215873405 1.85749345597114E-4
142.234223422342 0.0570535106061201 1.85715057669904E-4
142.254225422542 0.0570500003306538 1.85680780853539E-4
142.274227422742 0.0570464907605621 1.85646515141513E-4
142.294229422942 0.0570429818954707 1.85612260527371E-4
142.314231423142 0.0570394737350013 1.85578017004619E-4
142.334233423342 0.0570359662787802 1.85543784566814E-4
142.354235423542 0.0570324595264282 1.85509563207462E-4
142.374237423742 0.0570289534775746 1.85475352920152E-4
142.394239423942 0.0570254481318358 1.85441153698352E-4
142.414241424142 0.0570219434888444 1.85406965535689E-4
142.434243424342 0.0570184395482238 1.85372788425704E-4
142.454245424542 0.0570149363095943 1.85338622361909E-4
142.474247424742 0.0570114337725836 1.85304467337895E-4

142.494249424943 0.0570079319368159 1.85270323347215E-4
142.514251425143 0.0570044308019184 1.85236190383458E-4
142.534253425343 0.057000930367517 1.85202068440204E-4
142.554255425543 0.0569974306332313 1.8516795751097E-4
142.574257425743 0.0569939315986962 1.85133857589428E-4
142.594259425943 0.0569904332635327 1.85099768669116E-4
142.614261426143 0.056986935627365 1.85065690743606E-4
142.634263426343 0.0569834386898211 1.8503162380651E-4
142.654265426543 0.056979942450531 1.84997567851461E-4
142.674267426743 0.0569764469091169 1.8496352287202E-4
142.694269426943 0.0569729520652012 1.84929488861751E-4
142.714271427143 0.0569694579184221 1.84895465814376E-4
142.734273427343 0.0569659644684009 1.84861453723453E-4
142.754275427543 0.0569624717147613 1.84827452582569E-4
142.774277427743 0.0569589796571374 1.84793462385413E-4
142.794279427943 0.0569554882951508 1.84759483125554E-4
142.814281428143 0.0569519976284366 1.84725514796696E-4
142.834283428343 0.0569485076566118 1.84691557392369E-4
142.854285428543 0.0569450183793137 1.84657610906305E-4
142.874287428743 0.0569415297961655 1.84623675332099E-4
142.894289428943 0.0569380419067985 1.84589750663428E-4
142.914291429143 0.0569345547108387 1.84555836893921E-4
142.934293429343 0.0569310682079148 1.84521934017235E-4
142.954295429543 0.0569275823976575 1.84488042027049E-4
142.974297429743 0.0569240972796957 1.84454160917026E-4
142.994299429943 0.0569206128536576 1.84420290680827E-4
143.014301430143 0.0569171291191726 1.84386431312127E-4
143.034303430343 0.0569136460758709 1.84352582804608E-4
143.054305430543 0.0569101637233812 1.84318745151946E-4
143.074307430743 0.0569066820613324 1.84284918347815E-4
143.094309430943 0.0569032010893563 1.84251102385923E-4
143.114311431143 0.0568997208070836 1.84217297259972E-4
143.134313431343 0.0568962412141403 1.84183502963615E-4
143.154315431543 0.0568927623101611 1.84149719490598E-4
143.174317431743 0.0568892840947779 1.84115946834639E-4
143.194319431943 0.0568858065676175 1.8408218498941E-4
143.214321432143 0.0568823297283132 1.84048433948647E-4
143.234323432343 0.0568788535764951 1.84014693706061E-4
143.254325432543 0.0568753781117949 1.83980964255379E-4
143.274327432743 0.0568719033338439 1.83947245590325E-4
143.294329432943 0.056868429242273 1.83913537704625E-4
143.314331433143 0.056864955836717 1.83879840592042E-4
143.334333433343 0.056861483116805 1.8384615424629E-4
143.354335433543 0.0568580110821694 1.83812478661114E-4

143.374337433743 0.0568545397324449 1.83778813830286E-4
143.394339433943 0.056851069067261 1.83745159747528E-4
143.414341434143 0.0568475990862544 1.83711516406638E-4
143.434343434343 0.0568441297890512 1.83677883801309E-4
143.454345434543 0.0568406611752918 1.83644261925379E-4
143.474347434743 0.0568371932446105 1.83610650772628E-4
143.494349434944 0.0568337259966284 1.8357705033671E-4
143.514351435144 0.0568302594309933 1.83543460611541E-4
143.534353435344 0.0568267935473299 1.83509881590816E-4
143.554355435544 0.0568233283452759 1.83476313268357E-4
143.574357435744 0.0568198638244644 1.83442755637946E-4
143.594359435944 0.0568163999845339 1.83409208693417E-4
143.614361436144 0.0568129368251085 1.83375672428469E-4
143.634363436344 0.056809474345835 1.83342146837025E-4
143.654365436544 0.056806012546342 1.8330863191283E-4
143.674367436744 0.0568025514262646 1.83275127649698E-4
143.694369436944 0.0567990909852378 1.83241634041443E-4
143.714371437144 0.0567956312228988 1.83208151081902E-4
143.734373437344 0.0567921721388834 1.83174678764904E-4
143.754375437544 0.0567887137328257 1.8314121708426E-4
143.774377437744 0.0567852560043608 1.83107766033796E-4
143.794379437944 0.0567817989531262 1.83074325607361E-4
143.814381438144 0.0567783425787622 1.83040895798839E-4
143.834383438344 0.0567748868808995 1.83007476602016E-4
143.854385438544 0.0567714318591752 1.8297406801075E-4
143.874387438744 0.0567679775132289 1.82940670018919E-4
143.894389438944 0.0567645238426954 1.82907282620362E-4
143.914391439144 0.0567610708472164 1.82873905808985E-4
143.934393439344 0.0567576185264237 1.828405395786E-4
143.954395439544 0.0567541668799557 1.82807183923086E-4
143.974397439744 0.0567507159074555 1.82773838836371E-4
143.994399439944 0.0567472656085544 1.82740504312272E-4
144.014401440144 0.0567438159828948 1.8270718034471E-4
144.034403440344 0.0567403670301122 1.8267386692755E-4
144.054405440544 0.0567369187498512 1.82640564054743E-4
144.074407440744 0.0567334711417409 1.82607271720091E-4
144.094409440944 0.0567300242054301 1.82573989917591E-4
144.114411441144 0.0567265779405516 1.8254071864109E-4
144.134413441344 0.0567231323467487 1.82507457884535E-4
144.154415441544 0.0567196874236586 1.82474207641819E-4
144.174417441744 0.0567162431709169 1.82440967906819E-4
144.194419441944 0.0567127995881709 1.82407738673532E-4
144.214421442144 0.0567093566750556 1.82374519935835E-4
144.234423442344 0.056705914431218 1.82341311687725E-4

144.254425442544 0.0567024728562917 1.8230811392307E-4
144.274427442744 0.0566990319499218 1.82274926635856E-4
144.294429442944 0.0566955917117458 1.82241749819992E-4
144.314431443144 0.0566921521414061 1.82208583469441E-4
144.334433443344 0.056688713238543 1.82175427578147E-4
144.354435443544 0.0566852750028015 1.82142282140101E-4
144.374437443744 0.0566818374338187 1.82109147149222E-4
144.394439443944 0.0566784005312431 1.82076022599539E-4
144.414441444144 0.0566749642947058 1.82042908484919E-4
144.434443444344 0.0566715287238619 1.82009804799458E-4
144.454445444544 0.0566680938183454 1.81976711537054E-4
144.474447444744 0.0566646595778 1.81943628691701E-4
144.494449444945 0.0566612260018726 1.81910556257427E-4
144.514451445145 0.056657793090203 1.81877494228192E-4
144.534453445345 0.0566543608424366 1.81844442598012E-4
144.554455445545 0.0566509292582138 1.8181140136086E-4
144.574457445745 0.0566474983371815 1.81778370510772E-4
144.594459445945 0.0566440680789788 1.8174535004171E-4
144.614461446145 0.0566406384832562 1.81712339947752E-4
144.634463446345 0.0566372095496541 1.81679340222878E-4
144.654465446545 0.0566337812778186 1.81646350861126E-4
144.674467446745 0.0566303536673939 1.81613371856517E-4
144.694469446945 0.0566269267180247 1.81580403203084E-4
144.714471447145 0.0566235004293569 1.81547444894867E-4
144.734473447345 0.0566200748010362 1.81514496925911E-4
144.754475447545 0.0566166498327051 1.81481559290233E-4
144.774477447745 0.0566132255240111 1.81448631981896E-4
144.794479447945 0.0566098018746029 1.81415714994982E-4
144.814481448145 0.0566063788841224 1.81382808323506E-4
144.834483448345 0.0566029565522189 1.81349911961561E-4
144.854485448545 0.0565995348785387 1.81317025903209E-4
144.874487448745 0.0565961138627273 1.81284150142508E-4
144.894489448945 0.0565926935044321 1.81251284673538E-4
144.914491449145 0.0565892738033045 1.81218429490418E-4
144.934493449345 0.0565858547589864 1.81185584587181E-4
144.954495449545 0.0565824363711257 1.81152749957916E-4
144.974497449745 0.0565790186393709 1.81119925596724E-4
144.994499449945 0.056575601563377 1.81087111497766E-4
145.014501450145 0.0565721851427791 1.81054307655019E-4
145.034503450345 0.0565687693772372 1.81021514062701E-4
145.054505450545 0.056565354266396 1.80988730714881E-4
145.074507450745 0.0565619398099041 1.80955957605672E-4
145.094509450945 0.0565585260074092 1.80923194729181E-4
145.114511451145 0.0565551128585634 1.80890442079555E-4

145.134513451345 0.0565517003630171 1.80857699650933E-4
145.154515451545 0.056548288520418 1.80824967437426E-4
145.174517451745 0.0565448773304139 1.80792245433149E-4
145.194519451945 0.05654146679266 1.80759533632294E-4
145.214521452145 0.0565380569068029 1.80726832028966E-4
145.234523452345 0.056534647672498 1.80694140617363E-4
145.254525452545 0.0565312390893905 1.80661459391586E-4
145.274527452745 0.0565278311571372 1.80628788345846E-4
145.294529452945 0.056524423875383 1.80596127474248E-4
145.314531453145 0.0565210172437846 1.80563476771007E-4
145.334533453345 0.0565176112619924 1.80530836230283E-4
145.354535453545 0.0565142059296576 1.80498205846246E-4
145.374537453745 0.0565108012464314 1.80465585613066E-4
145.394539453945 0.0565073972119732 1.80432975524996E-4
145.414541454145 0.0565039938259243 1.80400375576119E-4
145.434543454345 0.0565005910879503 1.80367785760749E-4
145.454545454545 0.0564971889976916 1.80335206072965E-4
145.474547454745 0.0564937875548115 1.80302636507065E-4
145.494549454946 0.0564903867589596 1.80270077057219E-4
145.514551455146 0.0564869866097891 1.80237527717635E-4
145.534553455346 0.056483587106957 1.80204988482558E-4
145.554555455546 0.0564801882501129 1.80172459346163E-4
145.574557455746 0.0564767900389178 1.80139940302742E-4
145.594559455946 0.05647339247302 1.80107431346461E-4
145.614561456146 0.0564699955520759 1.8007493247157E-4
145.634563456346 0.0564665992757453 1.80042443672352E-4
145.654565456546 0.0564632036436801 1.80009964943019E-4
145.674567456746 0.0564598086555342 1.79977496277801E-4
145.694569456946 0.0564564143109663 1.79945037670979E-4
145.714571457146 0.0564530206096302 1.79912589116788E-4
145.734573457346 0.056449627551186 1.79880150609526E-4
145.754575457546 0.0564462351352886 1.79847722143444E-4
145.774577457746 0.0564428433615937 1.79815303712801E-4
145.794579457946 0.0564394522297559 1.7978289531185E-4
145.814581458146 0.0564360617394411 1.79750496934954E-4
145.834583458346 0.0564326718902981 1.79718108576313E-4
145.854585458546 0.0564292826819865 1.79685730230233E-4
145.874587458746 0.0564258941141698 1.79653361891062E-4
145.894589458946 0.0564225061864977 1.79621003553016E-4
145.914591459146 0.0564191188986363 1.7958865521047E-4
145.934593459346 0.0564157322502419 1.79556316857708E-4
145.954595459546 0.0564123462409685 1.79523988488995E-4
145.974597459746 0.0564089608704808 1.794916700987E-4
145.994599459946 0.0564055761384414 1.79459361681173E-4

146.014601460146 0.0564021920444997 1.79427063230642E-4
146.034603460346 0.0563988085883218 1.79394774741496E-4
146.054605460546 0.0563954257695686 1.79362496208076E-4
146.074607460746 0.0563920435879011 1.79330227624726E-4
146.094609460946 0.0563886620429777 1.7929796898577E-4
146.114611461146 0.0563852811344562 1.79265720285525E-4
146.134613461346 0.056381900862005 1.79233481518414E-4
146.154615461546 0.0563785212252804 1.79201252678744E-4
146.174617461746 0.0563751422239445 1.79169033760884E-4
146.194619461946 0.0563717638576579 1.79136824759186E-4
146.214621462146 0.056368386126088 1.79104625668071E-4
146.234623462346 0.0563650090288919 1.79072436481866E-4
146.254625462546 0.0563616325657332 1.79040257194959E-4
146.274627462746 0.0563582567362777 1.79008087801763E-4
146.294629462946 0.0563548815401839 1.78975928296624E-4
146.314631463146 0.0563515069771209 1.78943778673989E-4
146.334633463346 0.0563481330467466 1.78911638928204E-4
146.354635463546 0.0563447597487261 1.78879509053681E-4
146.374637463746 0.0563413870827243 1.78847389044838E-4
146.394639463946 0.0563380150484088 1.78815278896116E-4
146.414641464146 0.0563346436454383 1.78783178601875E-4
146.434643464346 0.0563312728734808 1.78751088156568E-4
146.454645464546 0.056327902732199 1.78719007554596E-4
146.474647464746 0.0563245332212638 1.78686936790443E-4
146.494649464946 0.0563211643403309 1.78654875858449E-4
146.514651465147 0.0563177960890775 1.78622824753163E-4
146.534653465347 0.0563144284671606 1.78590783468941E-4
146.554655465547 0.056311061474254 1.78558752000302E-4
146.574657465747 0.0563076951100177 1.78526730341638E-4
146.594659465947 0.0563043293741183 1.78494718487402E-4
146.614661466147 0.0563009642662302 1.78462716432128E-4
146.634663466347 0.0562975997860168 1.78430724170242E-4
146.654665466547 0.05629423593314 1.78398741696165E-4
146.674667466747 0.0562908727072774 1.78366769004462E-4
146.694669466947 0.0562875101080896 1.78334806089544E-4
146.714671467147 0.0562841481352483 1.78302852945928E-4
146.734673467347 0.0562807867884224 1.78270909568104E-4
146.754675467547 0.0562774260672797 1.78238975950558E-4
146.774677467747 0.0562740659714865 1.78207052087761E-4
146.794679467947 0.0562707065007198 1.78175137974289E-4
146.814681468147 0.0562673476546454 1.78143233604614E-4
146.834683468347 0.0562639894329288 1.78111338973206E-4
146.854685468547 0.0562606318352425 1.78079454074604E-4
146.874687468747 0.0562572748612578 1.7804757890334E-4

146.894689468947 0.0562539185106468 1.7801571345395E-4
146.914691469147 0.0562505627830838 1.77983857721001E-4
146.934693469347 0.0562472076782311 1.77952011698942E-4
146.954695469547 0.0562438531957633 1.77920175382343E-4
146.974697469747 0.0562404993353583 1.77888348765808E-4
146.994699469947 0.0562371460966776 1.77856531843786E-4
147.014701470147 0.0562337934793999 1.77824724610893E-4
147.034703470347 0.0562304414831982 1.77792927061693E-4
147.054705470547 0.0562270901077416 1.77761139190713E-4
147.074707470747 0.0562237393527113 1.77729360992598E-4
147.094709470947 0.0562203892177678 1.77697592461802E-4
147.114711471147 0.0562170397025904 1.77665833592953E-4
147.134713471347 0.0562136908068582 1.77634084380685E-4
147.154715471547 0.0562103425302427 1.77602344819559E-4
147.174717471747 0.0562069948724124 1.7757061490411E-4
147.194719471947 0.0562036478330476 1.77538894628988E-4
147.214721472147 0.0562003014118208 1.77507183988772E-4
147.234723472347 0.0561969556084104 1.77475482978093E-4
147.254725472547 0.0561936104224846 1.77443791591498E-4
147.274727472747 0.0561902658537295 1.77412109823696E-4
147.294729472947 0.0561869219018103 1.77380437669204E-4
147.314731473147 0.0561835785664152 1.7734877512276E-4
147.334733473347 0.0561802358472074 1.77317122178866E-4
147.354735473547 0.0561768937438767 1.77285478832277E-4
147.374737473747 0.0561735522560899 1.77253845077535E-4
147.394739473947 0.0561702113835312 1.77222220909346E-4
147.414741474147 0.0561668711258697 1.77190606322276E-4
147.434743474347 0.0561635314827914 1.77159001311054E-4
147.454745474547 0.0561601924539732 1.77127405870326E-4
147.474747474747 0.0561568540390922 1.77095819994738E-4
147.494749474948 0.0561535162378246 1.77064243678934E-4
147.514751475148 0.056150179049853 1.77032676917615E-4
147.534753475348 0.0561468424748563 1.77001119705457E-4
147.554755475548 0.0561435065125153 1.76969572037149E-4
147.574757475748 0.0561401711625027 1.76938033907308E-4
147.594759475948 0.0561368364245039 1.76906505310674E-4
147.614761476148 0.0561335022982038 1.76874986241984E-4
147.634763476348 0.0561301687832785 1.76843476695892E-4
147.654765476548 0.0561268358794028 1.76811976667045E-4
147.674767476748 0.0561235035862676 1.76780486150241E-4
147.694769476948 0.05612017190355 1.76749005140151E-4
147.714771477148 0.0561168408309308 1.76717533631484E-4
147.734773477348 0.0561135103680943 1.76686071618983E-4
147.754775477548 0.0561101805147228 1.76654619097374E-4

147.774777477748 0.056106851270501 1.76623176061408E-4
147.794779477948 0.0561035226351047 1.76591742505755E-4
147.814781478148 0.0561001946082201 1.76560318425183E-4
147.834783478348 0.0560968671895364 1.76528903814492E-4
147.854785478548 0.0560935403787327 1.76497498668389E-4
147.874787478748 0.0560902141754876 1.76466102981578E-4
147.894789478948 0.0560868885794947 1.76434716748903E-4
147.914791479148 0.0560835635904333 1.76403339965083E-4
147.934793479348 0.0560802392079939 1.76371972624937E-4
147.954795479548 0.0560769154318535 1.76340614723162E-4
147.974797479748 0.0560735922617042 1.76309266254601E-4
147.994799479948 0.0560702696972269 1.76277927213989E-4
148.014801480148 0.0560669477381095 1.76246597596133E-4
148.034803480348 0.0560636263840395 1.76215277395837E-4
148.054805480548 0.0560603056347039 1.76183966607901E-4
148.074807480748 0.0560569854897906 1.76152665227137E-4
148.094809480948 0.0560536659489827 1.76121373248315E-4
148.114811481148 0.0560503470119645 1.76090090666214E-4
148.134813481348 0.0560470286784327 1.76058817475736E-4
148.154815481548 0.056043710948073 1.7602755367168E-4
148.174817481748 0.056040393820571 1.75996299248846E-4
148.194819481948 0.0560370772956166 1.75965054202075E-4
148.214821482148 0.0560337613728994 1.7593381852621E-4
148.234823482348 0.0560304460521059 1.75902592216066E-4
148.254825482548 0.0560271313329273 1.75871375266502E-4
148.274827482748 0.0560238172150557 1.7584016767239E-4
148.294829482948 0.0560205036981785 1.75808969428558E-4
148.314831483148 0.0560171907819842 1.75777780529849E-4
148.334833483348 0.0560138784661684 1.75746600971172E-4
148.354835483548 0.0560105667504187 1.75715430747363E-4
148.374837483748 0.0560072556344309 1.75684269853339E-4
148.394839483948 0.0560039451178908 1.75653118283923E-4
148.414841484148 0.0560006352004901 1.75621976033996E-4
148.434843484348 0.0559973258819289 1.7559084309852E-4
148.454845484548 0.055994017161894 1.75559719472336E-4
148.474847484748 0.0559907090400741 1.75528605150301E-4
148.494849484949 0.0559874015161682 1.75497500127372E-4
148.514851485149 0.0559840945898708 1.75466404398469E-4
148.534853485349 0.0559807882608674 1.75435317958424E-4
148.554855485549 0.0559774825288636 1.75404240802264E-4
148.574857485749 0.0559741773935416 1.75373172924798E-4
148.594859485949 0.0559708728546075 1.75342114321057E-4
148.614861486149 0.0559675689117481 1.75311064985897E-4
148.634863486349 0.0559642655646613 1.7528002491428E-4

148.654865486549 0.0559609628130421 1.75248994101141E-4
148.674867486749 0.0559576606565885 1.75217972541446E-4
148.694869486949 0.0559543590949878 1.75186960230063E-4
148.714871487149 0.0559510581279485 1.75155957162058E-4
148.734873487349 0.0559477577551614 1.75124963332336E-4
148.754875487549 0.055944457976325 1.75093978735878E-4
148.774877487749 0.0559411587911331 1.75063003367616E-4
148.794879487949 0.0559378601992883 1.75032037222573E-4
148.814881488149 0.0559345622004863 1.75001080295705E-4
148.834883488349 0.0559312647944226 1.74970132581972E-4
148.854885488549 0.0559279679808009 1.74939194076409E-4
148.874887488749 0.0559246717593175 1.74908264773986E-4
148.894889488949 0.0559213761296667 1.74877344669656E-4
148.914891489149 0.055918081091559 1.74846433758523E-4
148.934893489349 0.0559147866446839 1.748155320355E-4
148.954895489549 0.0559114927887473 1.74784639495653E-4
148.974897489749 0.0559081995234497 1.74753756134001E-4
148.994899489949 0.0559049068484889 1.74722881945538E-4
149.014901490149 0.0559016147635622 1.74692016925256E-4
149.034903490349 0.0558983232683833 1.74661161068302E-4
149.054905490549 0.0558950323626413 1.74630314369596E-4
149.074907490749 0.0558917420460432 1.74599476824226E-4
149.094909490949 0.055888452318289 1.74568648427218E-4
149.114911491149 0.0558851631790873 1.74537829173677E-4
149.134913491349 0.0558818746281374 1.74507019058627E-4
149.154915491549 0.0558785866651422 1.74476218077123E-4
149.174917491749 0.055875299289803 1.74445426224214E-4
149.194919491949 0.0558720125018255 1.74414643494985E-4
149.214921492149 0.0558687263009177 1.74383869884546E-4
149.234923492349 0.0558654406867776 1.74353105387919E-4
149.254925492549 0.0558621556591102 1.74322350000189E-4
149.274927492749 0.0558588712176254 1.74291603716491E-4
149.294929492949 0.0558555873620296 1.74260866531929E-4
149.314931493149 0.0558523040920245 1.74230138441563E-4
149.334933493349 0.055849021407318 1.74199419440516E-4
149.354935493549 0.0558457393076123 1.74168709523858E-4
149.374937493749 0.0558424577926165 1.74138008686724E-4
149.394939493949 0.0558391768620435 1.74107316924289E-4
149.414941494149 0.0558358965155936 1.74076634231609E-4
149.434943494349 0.0558326167529731 1.74045960603802E-4
149.454945494549 0.0558293375739003 1.74015296036096E-4
149.474947494749 0.0558260589780726 1.73984640523528E-4
149.49494949495 0.0558227809652048 1.73953994061299E-4
149.51495149515 0.0558195035350005 1.73923356644506E-4

149.53495349535 0.0558162266871722 1.73892728268333E-4
149.55495549555 0.0558129504214343 1.73862108927982E-4
149.57495749575 0.0558096747374878 1.73831498618531E-4
149.59495949595 0.0558063996350475 1.73800897335192E-4
149.61496149615 0.0558031251138245 1.73770305073141E-4
149.63496349635 0.0557998511735237 1.73739721827499E-4
149.65496549655 0.05579657781387 1.73709147593576E-4
149.67496749675 0.0557933050345588 1.73678582366408E-4
149.69496949695 0.0557900328353112 1.73648026141273E-4
149.71497149715 0.05578676121584 1.73617478913373E-4
149.73497349735 0.0557834901758559 1.73586940677893E-4
149.75497549755 0.0557802197150671 1.73556411429995E-4
149.77497749775 0.0557769498331945 1.73525891164962E-4
149.79497949795 0.0557736805299444 1.73495379877944E-4
149.81498149815 0.0557704118050404 1.73464877564253E-4
149.83498349835 0.0557671436581832 1.73434384218989E-4
149.85498549855 0.0557638760890979 1.73403899837481E-4
149.87498749875 0.055760609097499 1.73373424414961E-4
149.89498949895 0.0557573426830916 1.73342957946578E-4
149.91499149915 0.0557540768456045 1.733125004277E-4
149.93499349935 0.0557508115847446 1.73282051853494E-4
149.95499549955 0.0557475469002324 1.73251612219256E-4
149.97499749975 0.0557442827917845 1.73221181520247E-4
149.99499949995 0.0557410192591119 1.73190759751679E-4
150.01500150015 0.0557377563019366 1.73160346908865E-4
150.03500350035 0.0557344939199762 1.73129942987082E-4
150.05500550055 0.0557312321129497 1.7309954798162E-4
150.07500750075 0.0557279708805701 1.73069161887717E-4
150.09500950095 0.0557247102225616 1.73038784700715E-4
150.11501150115 0.0557214501386375 1.73008416415855E-4
150.13501350135 0.0557181906285228 1.72978057028494E-4
150.15501550155 0.0557149316919358 1.72947706533923E-4
150.17501750175 0.0557116733285897 1.72917364927388E-4
150.19501950195 0.0557084155382135 1.72887032204289E-4
150.21502150215 0.0557051583205228 1.72856708359896E-4
150.23502350235 0.0557019016752392 1.72826393389541E-4
150.25502550255 0.0556986456020848 1.72796087288561E-4
150.27502750275 0.0556953901007827 1.72765790052304E-4
150.29502950295 0.0556921351710519 1.72735501676084E-4
150.31503150315 0.0556888808126169 1.72705222155263E-4
150.33503350335 0.0556856270251945 1.72674951485137E-4
150.35503550355 0.0556823738085147 1.72644689661127E-4
150.37503750375 0.0556791211622971 1.72614436678556E-4
150.39503950395 0.0556758690862668 1.72584192532801E-4

150.41504150415 0.0556726175801502 1.72553957219254E-4
150.43504350435 0.0556693666436676 1.72523730733251E-4
150.45504550455 0.0556661162765422 1.72493513070159E-4
150.47504750475 0.0556628664785033 1.72463304225398E-4
150.495049504951 0.0556596172492724 1.72433104194322E-4
150.515051505151 0.0556563685885753 1.72402912972326E-4
150.535053505351 0.0556531204961456 1.72372730554879E-4
150.555055505551 0.0556498729717002 1.72342556937295E-4
150.575057505751 0.0556466260149695 1.72312392115016E-4
150.595059505951 0.0556433796256815 1.72282236083465E-4
150.615061506151 0.0556401338035599 1.72252088838024E-4
150.635063506351 0.0556368885483351 1.72221950374142E-4
150.655065506551 0.0556336438597421 1.7219182068731E-4
150.675067506751 0.0556303997374966 1.72161699772842E-4
150.695069506951 0.0556271561813341 1.72131587626238E-4
150.715071507151 0.0556239131909845 1.72101484242949E-4
150.735073507351 0.0556206707661714 1.72071389618367E-4
150.755075507551 0.0556174289066382 1.72041303748069E-4
150.775077507751 0.0556141876120949 1.72011226627327E-4
150.795079507951 0.0556109468822899 1.71981158251768E-4
150.815081508151 0.0556077067169449 1.71951098616776E-4
150.835083508351 0.0556044671157948 1.71921047717854E-4
150.855085508551 0.0556012280785647 1.71891005550421E-4
150.875087508751 0.0555979896049984 1.71860972110069E-4
150.895089508951 0.0555947516948168 1.71830947392179E-4
150.915091509151 0.0555915143477574 1.71800931392289E-4
150.935093509351 0.0555882775635576 1.71770924105936E-4
150.955095509551 0.0555850413419389 1.71740925528511E-4
150.975097509751 0.0555818056826478 1.71710935655642E-4
150.995099509951 0.0555785705854095 1.71680954482758E-4
151.015101510151 0.0555753360499649 1.71650982005435E-4
151.035103510351 0.0555721020760449 1.71621018219159E-4
151.055105510551 0.0555688686633815 1.71591063119429E-4
151.075107510751 0.055565635811721 1.71561116701877E-4
151.095109510951 0.0555624035207829 1.71531178961887E-4
151.115111511151 0.055559171790321 1.71501249895165E-4
151.135113511351 0.0555559406200615 1.71471329497161E-4
151.155115511551 0.0555527100097452 1.71441417763463E-4
151.175117511751 0.0555494799591026 1.71411514689565E-4
151.195119511951 0.0555462504678786 1.71381620271096E-4
151.215121512151 0.055543021535814 1.71351734503648E-4
151.235123512351 0.0555397931626375 1.71321857382704E-4
151.255125512551 0.0555365653480928 1.71291988903886E-4
151.275127512751 0.0555333380919188 1.71262129062776E-4

151.295129512951 0.0555301113938565 1.71232277854972E-4
151.315131513151 0.0555268852536439 1.7120243527605E-4
151.335133513351 0.055523659671019 1.71172601321589E-4
151.355135513551 0.0555204346457256 1.71142775987217E-4
151.375137513751 0.05551721017751 1.71112959268593E-4
151.395139513951 0.0555139862660985 1.7108315116119E-4
151.415141514151 0.0555107629112449 1.71053351660736E-4
151.435143514351 0.0555075401126876 1.71023560762821E-4
151.455145514551 0.0555043178701728 1.70993778463106E-4
151.475147514751 0.0555010961834353 1.70964004757151E-4
151.495149514951 0.0554978750522208 1.70934239640614E-4
151.515151515152 0.0554946544762784 1.70904483109192E-4
151.535153515352 0.0554914344553467 1.70874735158481E-4
151.555155515552 0.0554882149891701 1.70844995784135E-4
151.575157515752 0.0554849960774956 1.70815264981835E-4
151.595159515952 0.0554817777200641 1.70785542747204E-4
151.615161516152 0.0554785599166283 1.70755829075977E-4
151.635163516352 0.0554753426669233 1.70726123963728E-4
151.655165516552 0.0554721259707049 1.70696427406226E-4
151.675167516752 0.0554689098277191 1.70666739399146E-4
151.695169516952 0.0554656942376994 1.70637059938054E-4
151.715171517152 0.0554624792004083 1.70607389018783E-4
151.735173517352 0.0554592647155883 1.70577726636983E-4
151.755175517552 0.0554560507829879 1.70548072788363E-4
151.775177517752 0.055452837402349 1.7051842746857E-4
151.795179517952 0.0554496245734294 1.70488790673401E-4
151.815181518152 0.0554464122959744 1.70459162398537E-4
151.835183518352 0.0554432005697313 1.70429542639683E-4
151.855185518552 0.0554399893944525 1.7039993139259E-4
151.875187518752 0.0554367787698909 1.70370328653015E-4
151.895189518952 0.0554335686957878 1.70340734416609E-4
151.915191519152 0.0554303591718978 1.70311148679149E-4
151.935193519352 0.0554271501979756 1.70281571436416E-4
151.955195519552 0.0554239417737734 1.70252002684164E-4
151.975197519752 0.0554207338990409 1.70222442418132E-4
151.995199519952 0.055417526573527 1.70192890634048E-4
152.015201520152 0.0554143197969915 1.70163347327745E-4
152.035203520352 0.055411113569184 1.70133812494961E-4
152.055205520552 0.0554079078898561 1.70104286131458E-4
152.075207520752 0.0554047027587639 1.70074768233036E-4
152.095209520952 0.0554014981756638 1.70045258795505E-4
152.115211521152 0.0553982941403023 1.7001575781458E-4
152.135213521352 0.0553950906524421 1.69986265286131E-4
152.155215521552 0.0553918877118413 1.69956781205983E-4

152.175217521752 0.0553886853182447 1.69927305569843E-4
152.195219521952 0.0553854834714185 1.69897838373617E-4
152.215221522152 0.0553822821711126 1.6986837961306E-4
152.235223522352 0.0553790814170865 1.6983892928402E-4
152.255225522552 0.0553758812091005 1.6980948738235E-4
152.275227522752 0.0553726815469112 1.69780053903873E-4
152.295229522952 0.0553694824302689 1.69750628844358E-4
152.315231523152 0.0553662838589397 1.69721212199714E-4
152.335233523352 0.0553630858326835 1.69691803965801E-4
152.355235523552 0.0553598883512528 1.69662404138409E-4
152.375237523752 0.0553566914144161 1.69633012713479E-4
152.395239523952 0.0553534950219257 1.69603629686802E-4
152.415241524152 0.0553502991735422 1.69574255054249E-4
152.435243524352 0.0553471038690301 1.69544888811726E-4
152.455245524552 0.0553439091081489 1.69515530955097E-4
152.475247524752 0.0553407148906617 1.69486181480259E-4
152.495249524953 0.0553375212163339 1.69456840383134E-4
152.515251525153 0.0553343280849131 1.69427507659477E-4
152.535253525353 0.0553311354961778 1.69398183305335E-4
152.555255525553 0.0553279434498803 1.6936886731651E-4
152.575257525753 0.0553247519457926 1.6933955968899E-4
152.595259525953 0.0553215609836791 1.69310260418692E-4
152.615261526153 0.0553183705632868 1.69280969501378E-4
152.635263526353 0.055315180684405 1.69251686933197E-4
152.655265526553 0.0553119913467838 1.69222412709941E-4
152.675267526753 0.0553088025501909 1.69193146827566E-4
152.695269526953 0.0553056142943906 1.69163889281993E-4
152.715271527153 0.0553024265791517 1.69134640069195E-4
152.735273527353 0.0552992394042434 1.69105399185141E-4
152.755275527553 0.0552960527694218 1.69076166625688E-4
152.775277527753 0.0552928666744701 1.69046942386939E-4
152.795279527953 0.0552896811191447 1.69017726464753E-4
152.815281528153 0.0552864961032162 1.68988518855123E-4
152.835283528353 0.0552833116264576 1.68959319554064E-4
152.855285528553 0.0552801276886237 1.68930128557425E-4
152.875287528753 0.0552769442895052 1.68900945861387E-4
152.895289528953 0.0552737614288541 1.68871771461777E-4
152.915291529153 0.0552705791064477 1.68842605354655E-4
152.935293529353 0.0552673973220595 1.68813447536048E-4
152.955295529553 0.055264216075452 1.68784298001884E-4
152.975297529753 0.0552610353664039 1.6875515674824E-4
152.995299529953 0.0552578551946823 1.68726023771088E-4
153.015301530153 0.0552546755600626 1.68696899066481E-4
153.035303530353 0.0552514964623107 1.6866778263038E-4

153.055305530553 0.0552483179012052 1.68638674458871E-4
153.075307530753 0.0552451398765234 1.68609574548023E-4
153.095309530953 0.0552419623880265 1.68580482893763E-4
153.115311531153 0.0552387854355034 1.68551399492271E-4
153.135313531353 0.0552356090187156 1.6852232433948E-4
153.155315531553 0.0552324331374438 1.68493257431499E-4
153.175317531753 0.0552292577914655 1.68464198764407E-4
153.195319531953 0.0552260829805515 1.68435148334226E-4
153.215321532153 0.0552229087044753 1.68406106137003E-4
153.235323532353 0.0552197349630272 1.68377072168941E-4
153.255325532553 0.0552165617559681 1.68348046425973E-4
153.275327532753 0.0552133890830845 1.68319028904271E-4
153.295329532953 0.0552102169441493 1.68290019599882E-4
153.315331533153 0.0552070453389414 1.68261018508908E-4
153.335333533353 0.0552038742672413 1.6823202562747E-4
153.355335533553 0.05520070372883 1.68203040951693E-4
153.375337533753 0.055197533723479 1.68174064477619E-4
153.395339533953 0.0551943642509682 1.68145096201364E-4
153.415341534153 0.0551911953110902 1.68116136119164E-4
153.435343534353 0.0551880269036131 1.68087184227034E-4
153.455345534553 0.0551848590283208 1.68058240521131E-4
153.475347534754 0.0551816916849985 1.68029304997629E-4
153.495349534954 0.0551785248734205 1.68000377652601E-4
153.515351535154 0.055175358593373 1.67971458482232E-4
153.535353535354 0.0551721928446374 1.67942547482665E-4
153.555355535554 0.0551690276270021 1.67913644650105E-4
153.575357535754 0.055165862940239 1.6788474998061E-4
153.595359535954 0.0551626987841458 1.67855863470475E-4
153.615361536154 0.0551595351584968 1.67826985115784E-4
153.635363536354 0.055156372063077 1.67798114912716E-4
153.655365536554 0.0551532094976719 1.67769252857461E-4
153.675367536754 0.0551500474620657 1.67740398946195E-4
153.695369536954 0.0551468859560506 1.67711553175169E-4
153.715371537154 0.0551437249794069 1.67682715540527E-4
153.735373537354 0.0551405645319256 1.67653886038513E-4
153.755375537554 0.0551374046133872 1.67625064665276E-4
153.775377537754 0.05513424522358 1.67596251417035E-4
153.795379537954 0.0551310863622994 1.6756744629008E-4
153.815381538154 0.0551279280293206 1.67538649280514E-4
153.835383538354 0.055124770224446 1.67509860384691E-4
153.855385538554 0.0551216129474569 1.67481079598775E-4
153.875387538754 0.055118456198141 1.67452306918986E-4
153.895389538954 0.0551152999762932 1.67423542341617E-4
153.915391539154 0.0551121442816997 1.67394785862876E-4

153.935393539354 0.0551089891141533 1.67366037479038E-4
153.955395539554 0.055105834473445 1.6733729718636E-4
153.975397539754 0.0551026803593636 1.67308564981083E-4
153.995399539954 0.0550995267717033 1.67279840859495E-4
154.015401540154 0.0550963737102599 1.67251124817902E-4
154.035403540354 0.0550932211748196 1.67222416852524E-4
154.055405540554 0.0550900691651714 1.67193716959607E-4
154.075407540754 0.0550869176811213 1.67165025135555E-4
154.095409540954 0.0550837667224579 1.67136341376611E-4
154.115411541154 0.0550806162889725 1.67107665679047E-4
154.135413541354 0.0550774663804585 1.67078998039156E-4
154.155415541554 0.0550743169967198 1.67050338453326E-4
154.175417541754 0.0550711681375467 1.67021686917823E-4
154.195419541954 0.0550680198027323 1.66993043428941E-4
154.215421542154 0.0550648719920728 1.66964407983E-4
154.235423542354 0.0550617247053704 1.66935780576381E-4
154.255425542554 0.0550585779424186 1.66907161205383E-4
154.275427542754 0.0550554317030171 1.66878549866364E-4
154.295429542954 0.0550522859869633 1.66849946555664E-4
154.315431543154 0.0550491407940583 1.66821351269656E-4
154.335433543354 0.055045996124094 1.6679276400463E-4
154.355435543554 0.0550428519768676 1.66764184756929E-4
154.375437543754 0.0550397083521892 1.66735613523011E-4
154.395439543954 0.0550365652498555 1.66707050299214E-4
154.415441544154 0.0550334226696675 1.66678495081919E-4
154.435443544354 0.0550302806114186 1.66649947867434E-4
154.455445544554 0.0550271390749206 1.66621408652242E-4
154.475447544754 0.0550239980599713 1.66592877432696E-4
154.495449544955 0.055020857566367 1.66564354205136E-4
154.515451545155 0.0550177175939164 1.66535838966021E-4
154.535453545355 0.0550145781424259 1.66507331711783E-4
154.555455545555 0.0550114392116903 1.66478832438755E-4
154.575457545755 0.0550083008015196 1.66450341143408E-4
154.595459545955 0.0550051629117134 1.6642185782212E-4
154.615461546155 0.0550020255420801 1.6639338247135E-4
154.635463546355 0.0549988886924279 1.66364915087557E-4
154.655465546555 0.0549957523625504 1.66336455667068E-4
154.675467546755 0.0549926165522678 1.66308004206452E-4
154.695469546955 0.054989481261379 1.66279560702088E-4
154.715471547155 0.0549863464896921 1.66251125150437E-4
154.735473547355 0.0549832122370119 1.66222697547934E-4
154.755475547555 0.054980078503148 1.66194277891058E-4
154.775477547755 0.0549769452879114 1.66165866176301E-4
154.795479547955 0.0549738125911061 1.66137462400092E-4

154.815481548155 0.0549706804125462 1.66109066558957E-4
154.835483548355 0.054967548752037 1.66080678649338E-4
154.855485548555 0.0549644176093813 1.66052298667661E-4
154.875487548755 0.0549612869844029 1.66023926610538E-4
154.895489548955 0.0549581568769046 1.65995562474395E-4
154.915491549155 0.0549550272867004 1.65967206255762E-4
154.935493549355 0.0549518982136033 1.65938857951159E-4
154.955495549555 0.0549487696574193 1.65910517557044E-4
154.975497549755 0.0549456416179619 1.65882185069944E-4
154.995499549955 0.0549425140950506 1.65853860486444E-4
155.015501550155 0.0549393870884919 1.65825543803009E-4
155.035503550355 0.0549362605981 1.65797235016176E-4
155.055505550555 0.0549331346236877 1.65768934122473E-4
155.075507550755 0.0549300091650766 1.65740641118506E-4
155.095509550955 0.0549268842220803 1.6571235600081E-4
155.115511551155 0.0549237597945001 1.65684078765811E-4
155.135513551355 0.05492063588217 1.65655809410233E-4
155.155515551555 0.0549175124848954 1.65627547930539E-4
155.175517551755 0.0549143896024945 1.65599294323314E-4
155.195519551955 0.0549112672347881 1.65571048585161E-4
155.215521552155 0.0549081453815921 1.65542810712648E-4
155.235523552355 0.0549050240427244 1.65514580702356E-4
155.255525552555 0.0549019032180011 1.65486358550852E-4
155.275527552755 0.054898782907239 1.65458144254713E-4
155.295529552955 0.0548956631102656 1.65429937810612E-4
155.315531553155 0.0548925438268926 1.6540173921508E-4
155.335533553355 0.0548894250569411 1.65373548464736E-4
155.355535553555 0.0548863068002342 1.65345365556217E-4
155.375537553755 0.0548831890565938 1.6531719048615E-4
155.395539553955 0.0548800718258363 1.65289023251113E-4
155.415541554155 0.0548769551077875 1.65260863847774E-4
155.435543554355 0.0548738389022669 1.65232712272739E-4
155.455545554555 0.0548707232091 1.65204568522675E-4
155.475547554755 0.0548676080281073 1.65176432594202E-4
155.495549554956 0.0548644933591169 1.65148304484011E-4
155.515551555156 0.0548613792019435 1.65120184188671E-4
155.535553555356 0.0548582655564243 1.65092071704958E-4
155.555555555556 0.0548551524223717 1.65063967029425E-4
155.575557555756 0.054852039799615 1.65035870158777E-4
155.595559555956 0.0548489276879861 1.65007781089745E-4
155.615561556156 0.054845816087296 1.64979699818871E-4
155.635563556356 0.0548427049973895 1.64951626343005E-4
155.655565556556 0.0548395944180808 1.64923560658723E-4
155.675567556756 0.0548364843492056 1.64895502762792E-4

155.695569556956 0.0548333747905835 1.64867452651837E-4
155.715571557156 0.0548302657420425 1.64839410322563E-4
155.735573557356 0.0548271572034262 1.64811375771812E-4
155.755575557556 0.054824049174543 1.64783348996111E-4
155.775577557756 0.0548209416552338 1.64755329992285E-4
155.795579557956 0.0548178346453332 1.64727318757097E-4
155.815581558156 0.0548147281446623 1.64699315287194E-4
155.835583558356 0.0548116221530509 1.64671319579302E-4
155.855585558556 0.0548085166703432 1.64643331630276E-4
155.875587558756 0.054805411696356 1.64615351436727E-4
155.895589558956 0.0548023072309243 1.6458737899543E-4
155.915591559156 0.0547992032738905 1.64559414303228E-4
155.935593559356 0.0547960998250775 1.64531457356791E-4
155.955595559556 0.0547929968843263 1.64503508152951E-4
155.975597559756 0.054789894451462 1.64475566688399E-4
155.995599559956 0.0547867925263278 1.64447632959989E-4
156.015601560156 0.0547836911087501 1.64419706964426E-4
156.035603560356 0.0547805901985739 1.64391788698584E-4
156.055605560556 0.0547774897956253 1.64363878159167E-4
156.075607560756 0.0547743898997439 1.64335975343001E-4
156.095609560956 0.0547712905107685 1.64308080246907E-4
156.115611561156 0.0547681916285332 1.64280192867666E-4
156.135613561356 0.0547650932528786 1.64252313202116E-4
156.155615561556 0.0547619953836406 1.64224441247054E-4
156.175617561756 0.0547588980206517 1.6419657699925E-4
156.195619561956 0.0547558011637617 1.64168720455628E-4
156.215621562156 0.0547527048128016 1.64140871612945E-4
156.235623562356 0.0547496089676123 1.6411303046805E-4
156.255625562556 0.0547465136280398 1.64085197017835E-4
156.275627562756 0.054743418793918 1.64057371259087E-4
156.295629562956 0.0547403244650923 1.64029553188698E-4
156.315631563156 0.0547372306413921 1.64001742803415E-4
156.335633563356 0.0547341373226761 1.63973940100251E-4
156.355635563556 0.0547310445087776 1.63946145075991E-4
156.375637563756 0.0547279521995389 1.63918357727503E-4
156.395639563956 0.0547248603948071 1.63890578051697E-4
156.415641564156 0.054721769094424 1.63862806045441E-4
156.435643564356 0.0547186782982315 1.63835041705598E-4
156.455645564556 0.0547155880060765 1.63807285029084E-4
156.475647564756 0.0547124982178035 1.6377953601279E-4
156.495649564956 0.0547094089332518 1.63751794653562E-4
156.515651565157 0.0547063201522792 1.63724060948415E-4
156.535653565357 0.0547032318747203 1.63696334894156E-4
156.555655565557 0.0547001441004332 1.63668616487803E-4

156.575657565757 0.0546970568292519 1.63640905726159E-4
156.595659565957 0.0546939700610339 1.63613202606241E-4
156.615661566157 0.0546908837956226 1.63585507124936E-4
156.635663566357 0.0546877980328709 1.63557819279222E-4
156.655665566557 0.0546847127726245 1.63530139066012E-4
156.675667566757 0.054681628014728 1.63502466482208E-4
156.695669566957 0.0546785437590395 1.63474801524838E-4
156.715671567157 0.0546754600054076 1.63447144190842E-4
156.735673567357 0.0546723767536751 1.63419494477111E-4
156.755675567557 0.0546692940037015 1.63391852380686E-4
156.775677567757 0.054666211755339 1.63364217898545E-4
156.795679567957 0.0546631300084323 1.63336591027599E-4
156.815681568157 0.0546600487628407 1.6330897176489E-4
156.835683568357 0.0546569680184095 1.63281360107335E-4
156.855685568557 0.0546538877750004 1.63253756052003E-4
156.875687568757 0.0546508080324655 1.63226159595873E-4
156.895689568957 0.0546477287906497 1.63198570735863E-4
156.915691569157 0.0546446500494205 1.63170989469095E-4
156.935693569357 0.0546415718086249 1.63143415792508E-4
156.955695569557 0.054638494068121 1.63115849703139E-4
156.975697569757 0.0546354168277624 1.63088291197989E-4
156.995699569957 0.0546323400874142 1.63060740274161E-4
157.015701570157 0.0546292638469237 1.630331969286E-4
157.035703570357 0.0546261881061505 1.63005661158364E-4
157.055705570557 0.0546231128649572 1.62978132960536E-4
157.075707570757 0.0546200381231937 1.62950612332087E-4
157.095709570957 0.0546169638807261 1.62923099270136E-4
157.115711571157 0.0546138901374109 1.62895593771715E-4
157.135713571357 0.0546108168931002 1.62868095833817E-4
157.155715571557 0.0546077441476698 1.62840605453651E-4
157.175717571757 0.0546046719009617 1.62813122628122E-4
157.195719571957 0.0546016001528542 1.6278564735446E-4
157.215721572157 0.0545985289031994 1.62758179629666E-4
157.235723572357 0.0545954581518588 1.62730719450822E-4
157.255725572557 0.0545923878986981 1.62703266815049E-4
157.275727572757 0.0545893181435798 1.62675821719441E-4
157.295729572957 0.0545862488863648 1.62648384161079E-4
157.315731573157 0.0545831801269121 1.6262095413703E-4
157.335733573357 0.0545801118650954 1.62593531644486E-4
157.355735573557 0.0545770441007777 1.62566116680555E-4
157.375737573757 0.0545739768338152 1.62538709242275E-4
157.395739573957 0.0545709100640831 1.62511309326861E-4
157.415741574157 0.0545678437914435 1.62483916931411E-4
157.435743574357 0.0545647780157561 1.62456532053002E-4

157.455745574557	0.0545617127369023	1.62429154688903E-4
157.475747574757	0.0545586479547363	1.62401784836146E-4
157.495749574958	0.0545555836691382	1.6237442249199E-4
157.515751575158	0.0545525198799576	1.62347067653429E-4
157.535753575358	0.0545494565870809	1.6231972031778E-4
157.555755575558	0.0545463937903666	1.62292380482115E-4
157.575757575758	0.0545433314896892	1.62265048143651E-4
157.595759575958	0.0545402696849162	1.62237723299541E-4
157.615761576158	0.0545372083759118	1.62210405946909E-4
157.635763576358	0.0545341475625642	1.62183096083098E-4
157.655765576558	0.0545310872447272	1.6215579370514E-4
157.675767576758	0.0545280274222821	1.62128498810318E-4
157.695769576958	0.0545249680950989	1.62101211395812E-4
157.715771577158	0.0545219092630442	1.62073931458772E-4
157.735773577358	0.0545188509260022	1.6204665899651E-4
157.755775577558	0.0545157930838366	1.62019394006151E-4
157.775777577758	0.0545127357364258	1.61992136484955E-4
157.795779577958	0.0545096788836483	1.61964886430184E-4
157.815781578158	0.0545066225253702	1.6193764383899E-4
157.835783578358	0.0545035666614673	1.6191040870861E-4
157.855785578558	0.0545005112918222	1.61883181036347E-4
157.875787578758	0.0544974564163061	1.61855960819398E-4
157.895789578958	0.0544944020348007	1.6182874805506E-4
157.915791579158	0.0544913481471815	1.6180154274057E-4
157.935793579358	0.0544882947533162	1.61774344873101E-4
157.955795579558	0.0544852418530944	1.61747154450021E-4
157.975797579758	0.0544821894463883	1.61719971468541E-4
157.995799579958	0.0544791375330828	1.6169279592599E-4
158.015801580158	0.0544760861130522	1.61665627819601E-4
158.035803580358	0.0544730351861801	1.61638467146692E-4
158.055805580558	0.0544699847523413	1.61611313904501E-4
158.075807580758	0.0544669348114188	1.61584168090344E-4
158.095809580958	0.0544638853632961	1.6155702970154E-4
158.115811581158	0.0544608364078577	1.61529898735416E-4
158.135813581358	0.0544577879449746	1.61502775189182E-4
158.155815581558	0.0544547399745411	1.61475659060257E-4
158.175817581758	0.0544516924964309	1.61448550345877E-4
158.195819581958	0.0544486455105346	1.61421449043426E-4
158.215821582158	0.0544455990167321	1.61394355150199E-4
158.235823582358	0.0544425530149111	1.61367268663557E-4
158.255825582558	0.0544395075049491	1.61340189580772E-4
158.275827582758	0.0544364624867394	1.6131311789926E-4
158.295829582958	0.0544334179601659	1.61286053616352E-4
158.315831583158	0.0544303739251077	1.61258996729335E-4

158.335833583358 0.0544273303814623 1.61231947235666E-4
158.355835583558 0.0544242873291072 1.61204905132621E-4
158.375837583758 0.0544212447679356 1.61177870417617E-4
158.395839583958 0.0544182026978386 1.61150843088054E-4
158.415841584158 0.0544151611186974 1.61123823141243E-4
158.435843584358 0.0544121200303991 1.61096810574552E-4
158.455845584558 0.0544090794328414 1.61069805385441E-4
158.475847584759 0.054406039325905 1.6104280757122E-4
158.495849584959 0.0544029997094902 1.61015817129374E-4
158.515851585159 0.0543999605834832 1.60988834057264E-4
158.535853585359 0.0543969219477678 1.60961858352232E-4
158.555855585559 0.054393883802246 1.6093489001178E-4
158.575857585759 0.0543908461468053 1.60907929033285E-4
158.595859585959 0.0543878089813407 1.60880975414186E-4
158.615861586159 0.0543847723057423 1.60854029151885E-4
158.635863586359 0.0543817361199018 1.60827090243798E-4
158.655865586559 0.0543787004237237 1.60800158687452E-4
158.675867586759 0.054375665217082 1.60773234480107E-4
158.695869586959 0.0543726304998964 1.6074631761943E-4
158.715871587159 0.0543695962720385 1.6071940810266E-4
158.735873587359 0.0543665625334217 1.60692505927408E-4
158.755875587559 0.0543635292839319 1.60665611091043E-4
158.775877587759 0.05436049652347 1.60638723591067E-4
158.795879587959 0.0543574642519367 1.6061184342498E-4
158.815881588159 0.0543544324692176 1.60584970590152E-4
158.835883588359 0.0543514011752195 1.6055810508414E-4
158.855885588559 0.0543483703698409 1.60531246904426E-4
158.875887588759 0.0543453400529767 1.60504396048464E-4
158.895889588959 0.0543423102245293 1.60477552513775E-4
158.915891589159 0.0543392808843987 1.60450716297858E-4
158.935893589359 0.0543362520324781 1.60423887398153E-4
158.955895589559 0.0543332236686807 1.60397065812279E-4
158.975897589759 0.0543301957928937 1.60370251537624E-4
158.995899589959 0.0543271684050354 1.60343444571854E-4
159.015901590159 0.0543241415049863 1.60316644912299E-4
159.035903590359 0.0543211150926688 1.60289852556662E-4
159.055905590559 0.0543180891679742 1.60263067502371E-4
159.075907590759 0.0543150637308108 1.60236289747007E-4
159.095909590959 0.0543120387810815 1.60209519288101E-4
159.115911591159 0.0543090143186874 1.60182756123171E-4
159.135913591359 0.0543059903435359 1.60156000249791E-4
159.155915591559 0.0543029668555334 1.60129251665525E-4
159.175917591759 0.0542999438545833 1.60102510367914E-4
159.195919591959 0.0542969213405931 1.60075776354536E-4

159.215921592159 0.0542938993134616 1.60049049622892E-4
159.235923592359 0.0542908777731119 1.60022330170696E-4
159.255925592559 0.0542878567194421 1.59995617995446E-4
159.275927592759 0.054284836152355 1.59968913094682E-4
159.295929592959 0.0542818160717697 1.59942215466084E-4
159.315931593159 0.0542787964775837 1.59915525107146E-4
159.335933593359 0.054275777369716 1.59888842015554E-4
159.355935593559 0.0542727587480713 1.59862166188865E-4
159.375937593759 0.054269740612558 1.5983549762467E-4
159.395939593959 0.0542667229630951 1.59808836320656E-4
159.415941594159 0.0542637057995865 1.59782182274377E-4
159.435943594359 0.0542606891219485 1.59755535483495E-4
159.455945594559 0.0542576729300819 1.59728895945539E-4
159.475947594759 0.0542546572239139 1.59702263658271E-4
159.49594959496 0.0542516420033488 1.59675638619249E-4
159.51595159516 0.0542486272682971 1.59649020826091E-4
159.53595359536 0.0542456130186858 1.59622410276556E-4
159.55595559556 0.0542425992544135 1.59595806968158E-4
159.57595759576 0.0542395859754049 1.59569210898639E-4
159.59595959596 0.0542365731815714 1.59542622065626E-4
159.61596159616 0.0542335608728316 1.59516040466809E-4
159.63596359636 0.0542305490490981 1.59489466099827E-4
159.65596559656 0.0542275377102864 1.59462898962344E-4
159.67596759676 0.0542245268563163 1.59436339052064E-4
159.69596959696 0.0542215164871063 1.59409786366679E-4
159.71597159716 0.0542185066025763 1.59383240903895E-4
159.73597359736 0.0542154972026381 1.59356702661346E-4
159.75597559756 0.0542124882872071 1.59330171636701E-4
159.77597759776 0.0542094798562126 1.59303647827749E-4
159.79597959796 0.0542064719095715 1.59277131232172E-4
159.81598159816 0.0542034644472034 1.59250621847679E-4
159.83598359836 0.0542004574690301 1.59224119671994E-4
159.85598559856 0.054197450974964 1.59197624702761E-4
159.87598759876 0.0541944449649308 1.59171136937745E-4
159.89598959896 0.0541914394388691 1.5914465637482E-4
159.91599159916 0.054188434396676 1.591181830115E-4
159.93599359936 0.0541854298382878 1.59091716845641E-4
159.95599559956 0.054182425763625 1.59065257874963E-4
159.97599759976 0.0541794221726145 1.59038806097244E-4
159.99599959996 0.0541764190651713 1.59012361510152E-4
160.01600160016 0.0541734164412353 1.58985924111584E-4
160.03600360036 0.0541704143007178 1.58959493899177E-4
160.05600560056 0.0541674126435509 1.58933070870758E-4
160.07600760076 0.0541644114696562 1.5890665502406E-4

160.09600960096 0.0541614107789633 1.58880246356887E-4
160.11601160116 0.0541584105713993 1.58853844867021E-4
160.13601360136 0.0541554108468875 1.58827450552213E-4
160.15601560156 0.0541524116053609 1.58801063410298E-4
160.17601760176 0.0541494128467524 1.58774683439114E-4
160.19601960196 0.0541464145709786 1.58748310636352E-4
160.21602160216 0.05414341677975 1.58721944999872E-4
160.23602360236 0.0541404194676709 1.58695586527482E-4
160.25602560256 0.0541374226399945 1.58669235216977E-4
160.27602760276 0.0541344262948779 1.58642891066191E-4
160.29602960296 0.0541314304322525 1.58616554072949E-4
160.31603160316 0.0541284350520499 1.58590224235081E-4
160.33603360336 0.0541254401542008 1.58563901550409E-4
160.35603560356 0.054122445738635 1.58537586016746E-4
160.37603760376 0.054119451805288 1.58511277631959E-4
160.39603960396 0.0541164583540956 1.58484976393915E-4
160.41604160416 0.0541134653849865 1.58458682300421E-4
160.43604360436 0.0541104728978975 1.58432395349357E-4
160.45604560456 0.0541074808927687 1.58406115538629E-4
160.47604760476 0.05410448936952 1.58379842865971E-4
160.49604960496 0.0541014983281023 1.58353577329384E-4
160.516051605161 0.0540985077684429 1.58327318926668E-4
160.536053605361 0.0540955176904814 1.58301067655729E-4
160.556055605561 0.054092528094154 1.58274823514443E-4
160.576057605761 0.0540895389793924 1.5824858650065E-4
160.596059605961 0.0540865503461426 1.58222356612312E-4
160.616061606161 0.054083562194336 1.58196133847269E-4
160.636063606361 0.0540805745239197 1.58169918203492E-4
160.656065606561 0.0540775873348224 1.581437096788E-4
160.676067606761 0.0540746006269928 1.58117508271179E-4
160.696069606961 0.054071614400368 1.58091313978522E-4
160.716071607161 0.0540686286548851 1.58065126798716E-4
160.736073607361 0.0540656433904874 1.58038946729708E-4
160.756075607561 0.0540626586071158 1.58012773769422E-4
160.776077607761 0.0540596743047116 1.57986607915787E-4
160.796079607961 0.0540566904832169 1.57960449166737E-4
160.816081608161 0.0540537071425718 1.57934297520195E-4
160.836083608361 0.0540507242827247 1.5790815297415E-4
160.856085608561 0.0540477419036193 1.57882015526556E-4
160.876087608761 0.0540447600051956 1.57855885175332E-4
160.896089608961 0.0540417785873945 1.57829761918409E-4
160.916091609161 0.0540387976501701 1.57803645753829E-4
160.936093609361 0.0540358171934633 1.57777536679524E-4
160.956095609561 0.0540328372172168 1.5775143469344E-4

160.976097609761 0.0540298577213818 1.57725339793597E-4
160.996099609961 0.0540268787059028 1.57699251977959E-4
161.016101610161 0.0540239001707254 1.576731712445E-4
161.036103610361 0.0540209221157997 1.57647097591233E-4
161.056105610561 0.0540179445410662 1.57621031016087E-4
161.076107610761 0.0540149674464857 1.57594971517171E-4
161.096109610961 0.0540119908319979 1.5756891909241E-4
161.116111611161 0.05400901469756 1.57542873739881E-4
161.136113611361 0.0540060390431102 1.57516835457495E-4
161.156115611561 0.0540030638686075 1.57490804243348E-4
161.176117611761 0.0540000891739995 1.57464780095436E-4
161.196119611961 0.053997114959236 1.57438763011773E-4
161.216121612161 0.0539941412242723 1.57412752990424E-4
161.236123612361 0.0539911679690628 1.57386750029445E-4
161.256125612561 0.0539881951935489 1.5736075412678E-4
161.276127612761 0.0539852228976887 1.5733476528052E-4
161.296129612961 0.0539822510814409 1.57308783488759E-4
161.316131613161 0.0539792797447546 1.57282808749512E-4
161.336133613361 0.0539763088875832 1.57256841060827E-4
161.356135613561 0.0539733385098805 1.57230880420762E-4
161.376137613761 0.0539703686116098 1.57204926827452E-4
161.396139613961 0.0539673991927118 1.57178980278841E-4
161.416141614161 0.0539644302531546 1.5715304077311E-4
161.436143614361 0.0539614617928909 1.57127108308305E-4
161.456145614561 0.053958493811873 1.57101182882471E-4
161.476147614761 0.0539555263100695 1.57075264493796E-4
161.496149614962 0.0539525592874223 1.57049353140235E-4
161.516151615162 0.0539495927439028 1.5702344882E-4
161.536153615362 0.0539466266794703 1.56997551531199E-4
161.556155615562 0.0539436610940671 1.56971661271792E-4
161.576157615762 0.0539406959876739 1.56945778040074E-4
161.596159615962 0.0539377313602341 1.56919901834015E-4
161.616161616162 0.0539347672117119 1.56894032651769E-4
161.636163616362 0.0539318035420826 1.56868170491587E-4
161.656165616562 0.05392884035128 1.56842315351355E-4
161.676167616762 0.0539258776392964 1.56816467229473E-4
161.696169616962 0.0539229154060719 1.56790626123886E-4
161.716171617162 0.0539199536515707 1.56764792032749E-4
161.736173617362 0.0539169923757625 1.56738964954266E-4
161.756175617562 0.0539140315786109 1.56713144886587E-4
161.776177617762 0.0539110712600723 1.56687331827803E-4
161.796179617962 0.0539081114201217 1.56661525776163E-4
161.816181618162 0.0539051520587146 1.56635726729749E-4
161.836183618362 0.0539021931758256 1.56609934686812E-4

161.856185618562 0.053899234771409 1.56584149645423E-4
161.876187618762 0.053896276845436 1.56558371603802E-4
161.896189618962 0.0538933193978733 1.56532600560131E-4
161.916191619162 0.0538903624286886 1.56506836512602E-4
161.936193619362 0.053887405937852 1.56481079459425E-4
161.956195619562 0.0538844499253204 1.564553293987E-4
161.976197619762 0.0538814943910689 1.56429586328682E-4
161.996199619962 0.0538785393350681 1.5640385024759E-4
162.016201620162 0.0538755847572838 1.56378121153601E-4
162.036203620362 0.0538726306576858 1.56352399044926E-4
162.056205620562 0.053869677036248 1.56326683919813E-4
162.076207620762 0.0538667238929355 1.56300975776436E-4
162.096209620962 0.0538637712277205 1.56275274613027E-4
162.116211621162 0.053860819040574 1.56249580427813E-4
162.136213621362 0.0538578673314722 1.56223893219063E-4
162.156215621562 0.0538549161003796 1.56198212984946E-4
162.176217621762 0.0538519653472738 1.56172539723744E-4
162.196219621962 0.0538490150721251 1.56146873433679E-4
162.216221622162 0.0538460652749038 1.5612121411297E-4
162.236223622362 0.0538431159555924 1.56095561759947E-4
162.256225622562 0.0538401671141553 1.56069916372778E-4
162.276227622762 0.0538372187505794 1.56044277949831E-4
162.296229622962 0.0538342708648271 1.56018646489258E-4
162.316231623162 0.0538313234568813 1.55993021989395E-4
162.336233623362 0.0538283765267122 1.55967404448461E-4
162.356235623562 0.0538254300743072 1.55941793864831E-4
162.376237623762 0.0538224840996346 1.55916190236712E-4
162.396239623962 0.0538195386026656 1.55890593562336E-4
162.416241624162 0.0538165935833905 1.55865003840103E-4
162.436243624362 0.0538136490417782 1.55839421068226E-4
162.456245624562 0.0538107049778133 1.55813845245056E-4
162.476247624762 0.0538077613914681 1.55788276368837E-4
162.496249624963 0.053804818282732 1.55762714437962E-4
162.516251625163 0.0538018756515699 1.55737159450613E-4
162.536253625363 0.0537989334979797 1.55711611405256E-4
162.556255625563 0.0537959918219254 1.55686070300067E-4
162.576257625763 0.0537930506233958 1.55660536133435E-4
162.596259625963 0.0537901099023783 1.55635008903737E-4
162.616261626163 0.0537871696588458 1.55609488609226E-4
162.636263626363 0.0537842298927836 1.55583975248264E-4
162.656265626563 0.0537812906041703 1.55558468819152E-4
162.676267626763 0.0537783517929973 1.55532969320305E-4
162.696269626963 0.0537754134592405 1.55507476750005E-4
162.716271627163 0.0537724756028883 1.55481991106639E-4

162.736273627363 0.0537695382239211 1.55456512388528E-4
162.756275627563 0.0537666013223301 1.55431040594088E-4
162.776277627763 0.0537636648980968 1.55405575721647E-4
162.796279627963 0.0537607289512052 1.5538011776956E-4
162.816281628163 0.0537577934816444 1.55354666736223E-4
162.836283628363 0.0537548584893969 1.55329222619978E-4
162.856285628563 0.0537519239744531 1.55303785419233E-4
162.876287628763 0.0537489899368003 1.55278355132372E-4
162.896289628963 0.0537460563764265 1.55252931757785E-4
162.916291629163 0.0537431232933159 1.55227515293828E-4
162.936293629363 0.0537401906874653 1.55202105738968E-4
162.956295629563 0.0537372585588524 1.55176703091507E-4
162.976297629763 0.0537343269074788 1.55151307349954E-4
162.996299629963 0.0537313957333202 1.55125918512594E-4
163.016301630163 0.0537284650363832 1.55100536577979E-4
163.036303630363 0.0537255348166468 1.55075161544425E-4
163.056305630563 0.0537226050741053 1.55049793410379E-4
163.076307630763 0.0537196758087527 1.55024432174286E-4
163.096309630963 0.0537167470205751 1.54999077834524E-4
163.116311631163 0.0537138187095722 1.54973730389587E-4
163.136313631363 0.053710890875728 1.54948389837836E-4
163.156315631563 0.0537079635190438 1.5492305617778E-4
163.176317631763 0.0537050366395081 1.54897729407819E-4
163.196319631963 0.0537021102371191 1.54872409526438E-4
163.216321632163 0.0536991843118652 1.54847096532035E-4
163.236323632363 0.0536962588637506 1.54821790423146E-4
163.256325632563 0.0536933338927625 1.54796491198163E-4
163.276327632763 0.0536904093988959 1.54771198855543E-4
163.296329632963 0.0536874853821505 1.54745913393783E-4
163.316331633163 0.053684561842519 1.54720634811325E-4
163.336333633363 0.0536816387800079 1.54695363106724E-4
163.356335633563 0.0536787161946053 1.54670098278383E-4
163.376337633763 0.0536757940863134 1.5464484032482E-4
163.396339633963 0.0536728724551248 1.54619589244478E-4
163.416341634163 0.0536699513010383 1.54594345035848E-4
163.436343634363 0.0536670306240647 1.54569107697528E-4
163.456345634563 0.053664110424194 1.54543877227936E-4
163.476347634763 0.0536611907014223 1.54518653625543E-4
163.496349634964 0.0536582714557532 1.54493436888886E-4
163.516351635164 0.0536553526871906 1.54468227016502E-4
163.536353635364 0.0536524343957302 1.54443024006862E-4
163.556355635564 0.0536495165813823 1.5441782785856E-4
163.576357635764 0.0536465992441368 1.54392638570016E-4
163.596359635964 0.053643682383999 1.54367456139782E-4

163.616361636164 0.0536407660009814 1.54342280566473E-4
163.636363636364 0.0536378500950776 1.54317111848543E-4
163.656365636564 0.0536349346662912 1.54291949984529E-4
163.676367636764 0.0536320197146273 1.54266794972985E-4
163.696369636964 0.0536291052400906 1.5424164681246E-4
163.716371637164 0.0536261912426889 1.54216505501529E-4
163.736373637364 0.0536232777224168 1.54191371038656E-4
163.756375637564 0.0536203646792956 1.54166243422535E-4
163.776377637764 0.0536174521133176 1.54141122651609E-4
163.796379637964 0.0536145400244903 1.54116008724453E-4
163.816381638164 0.0536116284128287 1.54090901639707E-4
163.836383638364 0.0536087172783344 1.54065801395897E-4
163.856385638564 0.053605806621007 1.54040707991529E-4
163.876387638764 0.053602896440869 1.54015621425312E-4
163.896389638964 0.0535999867379236 1.53990541695783E-4
163.916391639164 0.0535970775121742 1.53965468801485E-4
163.936393639364 0.0535941687636376 1.53940402741076E-4
163.956395639564 0.0535912604923131 1.53915343513063E-4
163.976397639764 0.0535883526982203 1.53890291116127E-4
163.996399639964 0.0535854453813636 1.53865245548821E-4
164.016401640164 0.0535825385417516 1.53840206809733E-4
164.036403640364 0.0535796321794015 1.53815174897528E-4
164.056405640564 0.0535767262943216 1.53790149810789E-4
164.076407640764 0.0535738208865287 1.53765131548177E-4
164.096409640964 0.0535709159560237 1.53740120108216E-4
164.116411641164 0.0535680115028233 1.53715115489565E-4
164.136413641364 0.0535651075269508 1.53690117690941E-4
164.156415641564 0.0535622040284025 1.53665126710828E-4
164.176417641764 0.0535593010072023 1.53640142547951E-4
164.196419641964 0.0535563984633723 1.53615165201014E-4
164.216421642164 0.0535534963969084 1.53590194668502E-4
164.236423642364 0.0535505948078393 1.53565230949179E-4
164.256425642564 0.0535476936961705 1.53540274041609E-4
164.276427642764 0.0535447930619263 1.53515323944522E-4
164.296429642964 0.0535418929051143 1.53490380656501E-4
164.316431643164 0.053538993225762 1.534654441763E-4
164.336433643364 0.0535360940238756 1.53440514502495E-4
164.356435643564 0.0535331952994773 1.53415591633795E-4
164.376437643764 0.0535302970525853 1.53390675568875E-4
164.396439643964 0.0535273992832111 1.53365766306358E-4
164.416441644164 0.053524501991384 1.53340863845013E-4
164.436443644364 0.0535216051771124 1.53315968183435E-4
164.456445644564 0.0535187088404157 1.53291079320312E-4
164.476447644764 0.0535158129813214 1.53266197254401E-4

164.496449644965 0.053512917599852 1.53241321984419E-4
164.516451645165 0.0535100226960125 1.53216453508929E-4
164.536453645365 0.0535071282698345 1.53191591826727E-4
164.556455645565 0.0535042343213378 1.53166736936504E-4
164.576457645765 0.0535013408505393 1.53141888836931E-4
164.596459645965 0.0534984478574639 1.53117047526743E-4
164.616461646165 0.0534955553421358 1.53092213004672E-4
164.636463646365 0.0534926633045708 1.53067385269378E-4
164.656465646565 0.053489771744802 1.5304256431967E-4
164.676467646765 0.0534868806628432 1.5301775015419E-4
164.696469646965 0.0534839900587196 1.52992942771681E-4
164.716471647165 0.0534810999324625 1.52968142170935E-4
164.736473647365 0.0534782102840894 1.5294334835063E-4
164.756475647565 0.0534753211136224 1.52918561309479E-4
164.776477647765 0.0534724324210969 1.52893781046314E-4
164.796479647965 0.0534695442065335 1.52869007559836E-4
164.816481648165 0.0534666564699517 1.52844240848742E-4
164.836483648365 0.0534637692113845 1.52819480911839E-4
164.856485648565 0.0534608824308625 1.52794727747918E-4
164.876487648765 0.0534579961284039 1.52769981355663E-4
164.896489648965 0.053455110304037 1.52745241733844E-4
164.916491649165 0.0534522249577921 1.52720508881249E-4
164.936493649365 0.0534493400896994 1.52695782796666E-4
164.956495649565 0.0534464556997876 1.52671063478869E-4
164.976497649765 0.0534435717880704 1.52646350926505E-4
164.996499649965 0.0534406883546069 1.5262164513861E-4
165.016501650165 0.0534378053993988 1.52596946113727E-4
165.036503650365 0.0534349229224887 1.52572253850752E-4
165.056505650565 0.0534320409239075 1.5254756834848E-4
165.076507650765 0.0534291594036776 1.52522889605632E-4
165.096509650965 0.0534262783618398 1.52498217621089E-4
165.116511651165 0.0534233977984139 1.52473552393551E-4
165.136513651365 0.0534205177134462 1.52448893921947E-4
165.156515651565 0.053417638106961 1.52424242205015E-4
165.176517651765 0.0534147589789952 1.52399597241605E-4
165.196519651965 0.0534118803295725 1.52374959030449E-4
165.216521652165 0.0534090021587295 1.52350327570395E-4
165.236523652365 0.0534061244665028 1.52325702860288E-4
165.256525652565 0.0534032472529248 1.52301084898938E-4
165.276527652765 0.0534003705180335 1.52276473685204E-4
165.296529652965 0.0533974942618589 1.52251869217875E-4
165.316531653165 0.0533946184844353 1.52227271495779E-4
165.336533653365 0.0533917431857995 1.52202680517765E-4
165.356535653565 0.0533888683659883 1.5217809628268E-4

165.376537653765 0.0533859940250362 1.52153518789356E-4
165.396539653965 0.0533831201629813 1.52128948036653E-4
165.416541654165 0.0533802467798588 1.52104384023406E-4
165.436543654365 0.053377373875705 1.52079826748461E-4
165.456545654565 0.0533745014505609 1.52055276210705E-4
165.476547654765 0.0533716295044627 1.52030732408982E-4
165.496549654965 0.0533687580374485 1.52006195342153E-4
165.516551655166 0.0533658870495537 1.5198166500906E-4
165.536553655366 0.0533630165408173 1.5195714140857E-4
165.556555655566 0.0533601465112886 1.51932624539641E-4
165.576557655766 0.0533572769609882 1.51908114400988E-4
165.596559655966 0.0533544078899735 1.51883610991637E-4
165.616561656166 0.053351539298278 1.51859114310411E-4
165.636563656366 0.0533486711859454 1.51834624356224E-4
165.656565656566 0.0533458035530116 1.51810141127918E-4
165.676567656766 0.0533429363995202 1.51785664624404E-4
165.696569656966 0.0533400697255107 1.5176119484456E-4
165.716571657166 0.0533372035310305 1.51736731787327E-4
165.736573657366 0.0533343378161146 1.51712275451543E-4
165.756575657566 0.0533314725808124 1.51687825836169E-4
165.776577657766 0.0533286078251607 1.5166338294006E-4
165.796579657966 0.0533257435492085 1.51638946762173E-4
165.816581658166 0.053322879752994 1.51614517301374E-4
165.836583658366 0.0533200164365649 1.5159009455661E-4
165.856585658566 0.0533171535999699 1.51565678526837E-4
165.876587658766 0.0533142912432438 1.51541269210893E-4
165.896589658966 0.0533114293664362 1.51516866607741E-4
165.916591659166 0.0533085679695962 1.51492470716343E-4
165.936593659366 0.0533057070527655 1.51468081535594E-4
165.956595659566 0.0533028466159852 1.51443699064387E-4
165.976597659766 0.053299986659309 1.51419323301721E-4
165.996599659966 0.0532971271827848 1.51394954246548E-4
166.016601660166 0.053294268186452 1.51370591897745E-4
166.036603660366 0.0532914096703635 1.51346236254306E-4
166.056605660566 0.053288551634568 1.51321887315188E-4
166.076607660766 0.05328569407911 1.51297545079314E-4
166.096609660966 0.0532828370040347 1.51273209545613E-4
166.116611661166 0.0532799804093979 1.51248880713102E-4
166.136613661366 0.0532771242952521 1.51224558580774E-4
166.156615661566 0.0532742686616285 1.51200243147439E-4
166.176617661766 0.0532714135085912 1.51175934412184E-4
166.196619661966 0.0532685588361857 1.51151632373944E-4
166.216621662166 0.0532657046444729 1.51127337031781E-4
166.236623662366 0.0532628509334868 1.5110304838453E-4

166.256625662566 0.0532599977032885 1.51078766431255E-4
166.276627662766 0.0532571449539276 1.51054491170924E-4
166.296629662966 0.0532542926854517 1.51030222602489E-4
166.316631663166 0.0532514408979157 1.51005960724962E-4
166.336633663366 0.0532485895913764 1.50981705537372E-4
166.356635663566 0.053245738765873 1.50957457038599E-4
166.376637663766 0.0532428884214746 1.50933215227778E-4
166.396639663966 0.0532400385582173 1.50908980103761E-4
166.416641664166 0.0532371891761727 1.50884751665705E-4
166.436643664366 0.0532343402753816 1.50860529912505E-4
166.456645664566 0.053231491855906 1.50836314843233E-4
166.476647664766 0.0532286439177888 1.50812106456804E-4
166.496649664967 0.0532257964610937 1.50787904752306E-4
166.516651665167 0.0532229494858807 1.50763709728797E-4
166.536653665367 0.0532201029921972 1.50739521385228E-4
166.556655665567 0.0532172569800962 1.50715339720598E-4
166.576657665767 0.0532144114496375 1.50691164733964E-4
166.596659665967 0.0532115664008826 1.50666996424397E-4
166.616661666167 0.053208721833877 1.50642834790833E-4
166.636663666367 0.0532058777486857 1.50618679832373E-4
166.656665666567 0.0532030341453681 1.50594531548071E-4
166.676667666767 0.0532001910239731 1.50570389936893E-4
166.696669666967 0.0531973483845646 1.5054625499793E-4
166.716671667167 0.0531945062271999 1.50522126730219E-4
166.736673667367 0.0531916645519383 1.50498005132814E-4
166.756675667567 0.0531888233588299 1.5047389020469E-4
166.776677667767 0.053185982647945 1.50449781944996E-4
166.796679667967 0.0531831424193364 1.5042568035273E-4
166.816681668167 0.05318030267307 1.50401585427003E-4
166.836683668367 0.0531774634091913 1.50377497166751E-4
166.856685668567 0.0531746246277791 1.50353415571196E-4
166.876687668767 0.0531717863288871 1.50329340639344E-4
166.896689668967 0.0531689485125648 1.50305272370166E-4
166.916691669167 0.0531661111788879 1.50281210762858E-4
166.936693669367 0.0531632743279083 1.50257155816412E-4
166.956695669567 0.0531604379596947 1.50233107529962E-4
166.976697669767 0.0531576020743063 1.50209065902563E-4
166.996699669967 0.0531547666718054 1.50185030933297E-4
167.016701670167 0.0531519317522529 1.50161002621232E-4
167.036703670367 0.0531490973157118 1.50136980965457E-4
167.056705670567 0.0531462633622542 1.50112965965135E-4
167.076707670767 0.0531434298919296 1.50088957619239E-4
167.096709670967 0.0531405969048028 1.50064955926874E-4
167.116711671167 0.0531377644009423 1.50040960887172E-4

167.136713671367 0.0531349323804258 1.50016972499347E-4
167.156715671567 0.0531321008432928 1.49992990762287E-4
167.176717671767 0.0531292697896296 1.49969015675278E-4
167.196719671967 0.0531264392194841 1.49945047237281E-4
167.216721672167 0.0531236091329323 1.49921085447493E-4
167.236723672367 0.0531207795300311 1.49897130304952E-4
167.256725672567 0.0531179504108561 1.49873181808854E-4
167.276727672767 0.0531151217754675 1.49849239958263E-4
167.296729672967 0.0531122936239421 1.49825304752385E-4
167.316731673167 0.0531094659563301 1.49801376190202E-4
167.336733673367 0.0531066387726988 1.49777454270837E-4
167.356735673567 0.0531038120731422 1.49753538993645E-4
167.376737673767 0.0531009858576973 1.49729630357493E-4
167.396739673967 0.0530981601264454 1.49705728361626E-4
167.416741674167 0.0530953348794573 1.496818330052E-4
167.436743674367 0.053092510116789 1.49657944287245E-4
167.456745674567 0.0530896858385217 1.49634062207006E-4
167.476747674767 0.0530868620447165 1.49610186763556E-4
167.496749674968 0.0530840387354542 1.49586317956137E-4
167.516751675168 0.0530812159107852 1.49562455783731E-4
167.536753675368 0.0530783935707926 1.495386002456E-4
167.556755675568 0.0530755717155493 1.49514751340917E-4
167.576757675768 0.0530727503451155 1.49490909068749E-4
167.596759675968 0.0530699294595678 1.49467073428303E-4
167.616761676168 0.053067109058975 1.49443244418718E-4
167.636763676368 0.0530642891434105 1.49419422039173E-4
167.656765676568 0.0530614697129391 1.49395606288775E-4
167.676767676768 0.0530586507676364 1.4937179716672E-4
167.696769676968 0.0530558323075751 1.49347994672183E-4
167.716771677168 0.0530530143328307 1.4932419880436E-4
167.736773677368 0.0530501968434677 1.49300409562356E-4
167.756775677568 0.0530473798395668 1.49276626945413E-4
167.776777677768 0.0530445633211931 1.49252850952638E-4
167.796779677968 0.0530417472884193 1.49229081583207E-4
167.816781678168 0.0530389317413269 1.49205318836366E-4
167.836783678368 0.0530361166799838 1.49181562711252E-4
167.856785678568 0.053033302104467 1.49157813207072E-4
167.876787678768 0.0530304880148468 1.49134070322982E-4
167.896789678968 0.0530276744112046 1.49110334058229E-4
167.916791679168 0.0530248612936003 1.4908660441188E-4
167.936793679368 0.05302204866212 1.49062881383221E-4
167.956795679568 0.053019236516841 1.49039164971467E-4
167.976797679768 0.0530164248578345 1.49015455175779E-4
167.996799679968 0.053013613685175 1.48991751995348E-4

168.016801680168 0.0530108029989329 1.48968055429329E-4
168.036803680368 0.0530079927991993 1.48944365477053E-4
168.056805680568 0.0530051830860386 1.48920682137623E-4
168.076807680768 0.0530023738595312 1.48897005410281E-4
168.096809680968 0.0529995651197477 1.48873335294184E-4
168.116811681168 0.0529967568667725 1.48849671788606E-4
168.136813681368 0.0529939491006845 1.48826014892775E-4
168.156815681568 0.0529911418215556 1.48802364605859E-4
168.176817681768 0.0529883350294615 1.4877872092706E-4
168.196819681968 0.0529855287244873 1.48755083855656E-4
168.216821682168 0.0529827229067101 1.48731453390863E-4
168.236823682368 0.0529799175762022 1.48707829531852E-4
168.256825682568 0.0529771127330445 1.48684212277868E-4
168.276827682768 0.0529743083773177 1.48660601628155E-4
168.296829682968 0.0529715045090993 1.48636997581929E-4
168.316831683168 0.0529687011284731 1.4861340013846E-4
168.336833683368 0.0529658982355141 1.48589809296944E-4
168.356835683568 0.052963095830293 1.4856622505654E-4
168.376837683768 0.052960293912915 1.48542647416698E-4
168.396839683968 0.0529574924834335 1.48519076376433E-4
168.416841684168 0.0529546915419456 1.48495511935127E-4
168.436843684368 0.0529518910885175 1.48471954091901E-4
168.456845684568 0.052949091123248 1.48448402846153E-4
168.476847684768 0.0529462916462066 1.48424858197032E-4
168.496849684969 0.052943492657474 1.48401320143783E-4
168.516851685169 0.0529406941571382 1.4837778868571E-4
168.536853685369 0.0529378961452734 1.48354263822006E-4
168.556855685569 0.0529350986219672 1.48330745551971E-4
168.576857685769 0.0529323015873012 1.48307233874859E-4
168.596859685969 0.0529295050413467 1.48283728789833E-4
168.616861686169 0.0529267089842045 1.4826023029631E-4
168.636863686369 0.0529239134159495 1.48236738393483E-4
168.656865686569 0.052921118336654 1.48213253080529E-4
168.676867686769 0.0529183237464216 1.48189774356885E-4
168.696869686969 0.052915529645318 1.48166302221669E-4
168.716871687169 0.0529127360334338 1.4814283667421E-4
168.736873687369 0.0529099429108487 1.48119377713744E-4
168.756875687569 0.0529071502776504 1.48095925339575E-4
168.776877687769 0.0529043581339305 1.48072479551042E-4
168.796879687969 0.0529015664797662 1.48049040347359E-4
168.816881688169 0.0528987753152286 1.48025607727691E-4
168.836883688369 0.052895984640422 1.48002181691483E-4
168.856885688569 0.0528931944554268 1.47978762237978E-4
168.876887688769 0.0528904047603178 1.4795534936637E-4

168.896889688969 0.0528876155551954 1.47931943076072E-4
168.916891689169 0.0528848268401363 1.47908543366297E-4
168.936893689369 0.052882038615226 1.4788515023633E-4
168.956895689569 0.0528792508805463 1.47861763685425E-4
168.976897689769 0.0528764636361958 1.47838383712981E-4
168.996899689969 0.052873676882257 1.47815010318257E-4
169.016901690169 0.0528708906188006 1.47791643500416E-4
169.036903690369 0.0528681048459397 1.47768283258977E-4
169.056905690569 0.0528653195637309 1.47744929592982E-4
169.076907690769 0.0528625347722826 1.47721582501912E-4
169.096909690969 0.0528597504716826 1.47698241985072E-4
169.116911691169 0.0528569666620003 1.47674908041613E-4
169.136913691369 0.05285418334335 1.47651580671064E-4
169.156915691569 0.0528514005157943 1.4762825987252E-4
169.176917691769 0.0528486181794268 1.47604945645335E-4
169.196919691969 0.0528458363343465 1.47581637988911E-4
169.216921692169 0.0528430549806353 1.47558336902502E-4
169.236923692369 0.0528402741183749 1.47535042385366E-4
169.256925692569 0.0528374937476588 1.47511754436856E-4
169.276927692769 0.0528347138685799 1.47488473056321E-4
169.296929692969 0.0528319344812218 1.47465198243032E-4
169.316931693169 0.052829155585679 1.47441929996353E-4
169.336933693369 0.0528263771820319 1.47418668315528E-4
169.356935693569 0.0528235992703722 1.47395413199896E-4
169.376937693769 0.052820821850801 1.47372164648875E-4
169.396939693969 0.0528180449233985 1.47348922661708E-4
169.416941694169 0.0528152684882546 1.47325687237722E-4
169.436943694369 0.0528124925454529 1.47302458376185E-4
169.456945694569 0.0528097170950878 1.47279236076461E-4
169.476947694769 0.0528069421372549 1.47256020337922E-4
169.49694969497 0.0528041676720474 1.47232811159922E-4
169.51695169517 0.0528013936995427 1.47209608541678E-4
169.53695369537 0.0527986202198427 1.47186412482617E-4
169.55695569557 0.0527958472330311 1.47163222982012E-4
169.57695769577 0.0527930747392069 1.47140040039263E-4
169.59695969597 0.0527903027384554 1.47116863653657E-4
169.61696169617 0.0527875312308656 1.4709369382451E-4
169.63696369637 0.0527847602165306 1.47070530551177E-4
169.65696569657 0.0527819896955498 1.4704737383306E-4
169.67696769677 0.0527792196680074 1.47024223669436E-4
169.69696969697 0.052776450133996 1.47001080059654E-4
169.71697169717 0.0527736810936065 1.46977943003047E-4
169.73697369737 0.0527709125469332 1.46954812498977E-4
169.75697569757 0.0527681444940682 1.46931688546787E-4

169.77697769777 0.0527653769351032 1.46908571145819E-4
169.79697969797 0.0527626098701376 1.46885460295476E-4
169.81698169817 0.0527598432992565 1.46862355995044E-4
169.83698369837 0.0527570772225426 1.46839258243788E-4
169.85698569857 0.0527543116401123 1.46816167041256E-4
169.87698769877 0.0527515465520441 1.46793082386676E-4
169.89698969897 0.0527487819584302 1.46770004279396E-4
169.91699169917 0.0527460178593668 1.4674693271879E-4
169.93699369937 0.0527432542549594 1.46723867704318E-4
169.95699569957 0.0527404911452742 1.46700809235105E-4
169.97699769977 0.0527377285304307 1.46677757310725E-4
169.99699969997 0.0527349664105191 1.46654711930507E-4
170.01700170017 0.0527322047856117 1.46631673093629E-4
170.03700370037 0.0527294436558231 1.46608640799622E-4
170.05700570057 0.0527266830212452 1.46585615047831E-4
170.07700770077 0.0527239228819696 1.46562595837594E-4
170.09700970097 0.0527211632380931 1.46539583168296E-4
170.11701170117 0.0527184040897107 1.46516577039305E-4
170.13701370137 0.0527156454369034 1.46493577449872E-4
170.15701570157 0.0527128872797801 1.46470584399483E-4
170.17701770177 0.0527101296184344 1.46447597887494E-4
170.19701970197 0.052707372452954 1.46424617913214E-4
170.21702170217 0.0527046157834479 1.46401644476127E-4
170.23702370237 0.0527018596099928 1.46378677575451E-4
170.25702570257 0.0526991039326951 1.46355717210648E-4
170.27702770277 0.0526963487516512 1.46332763381099E-4
170.29702970297 0.0526935940669538 1.46309816086155E-4
170.31703170317 0.0526908398786988 1.46286875325192E-4
170.33703370337 0.0526880861869792 1.46263941097562E-4
170.35703570357 0.0526853329918977 1.46241013402698E-4
170.37703770377 0.0526825802935342 1.46218092239843E-4
170.39703970397 0.0526798280920086 1.46195177608574E-4
170.41704170417 0.0526770763873933 1.46172269508072E-4
170.43704370437 0.052674325179808 1.46149367937912E-4
170.45704570457 0.0526715744693262 1.46126472897283E-4
170.47704770477 0.0526688242560564 1.46103584385668E-4
170.49704970497 0.0526660745400868 1.4608070240238E-4
170.517051705171 0.0526633253215299 1.46057826946933E-4
170.537053705371 0.0526605766004657 1.46034958018571E-4
170.557055705571 0.052657828377004 1.46012095616787E-4
170.577057705771 0.0526550806512286 1.45989239740858E-4
170.597059705971 0.0526523334232412 1.45966390390209E-4
170.617061706171 0.052649586693143 1.45943547564261E-4
170.637063706371 0.0526468404610215 1.45920711262319E-4

170.657065706571 0.0526440947269813 1.45897881483836E-4
170.677067706771 0.0526413494911197 1.45875058228199E-4
170.697069706971 0.0526386047535382 1.45852241494831E-4
170.717071707171 0.0526358605143211 1.45829431283015E-4
170.737073707371 0.0526331167735707 1.45806627592178E-4
170.757075707571 0.0526303735313879 1.45783830421742E-4
170.777077707771 0.0526276307878728 1.45761039771117E-4
170.797079707971 0.0526248885431079 1.4573825563957E-4
170.817081708171 0.0526221467972123 1.45715478026671E-4
170.837083708371 0.0526194055502583 1.45692706931601E-4
170.857085708571 0.0526166648023689 1.45669942353963E-4
170.877087708771 0.0526139245536277 1.45647184293031E-4
170.897089708971 0.0526111848041363 1.45624432748231E-4
170.917091709171 0.0526084455539851 1.45601687718895E-4
170.937093709371 0.0526057068032801 1.45578949204483E-4
170.957095709571 0.0526029685521207 1.45556217204404E-4
170.977097709771 0.0526002308005975 1.45533491717989E-4
170.997099709971 0.0525974935488171 1.45510772744706E-4
171.017101710171 0.0525947567968669 1.45488060283862E-4
171.037103710371 0.0525920205448477 1.45465354334876E-4
171.057105710571 0.0525892847928682 1.45442654897229E-4
171.077107710771 0.0525865495410158 1.45419961970232E-4
171.097109710971 0.0525838147893918 1.45397275553306E-4
171.117111711171 0.0525810805380952 1.45374595645854E-4
171.137113711371 0.052578346787213 1.4535192224718E-4
171.157115711571 0.052575613536869 1.45329255356895E-4
171.177117711771 0.0525728807871429 1.45306594974241E-4
171.197119711971 0.0525701485381336 1.45283941098621E-4
171.217121712171 0.0525674167899376 1.45261293729419E-4
171.237123712371 0.0525646855426614 1.452386528661E-4
171.257125712571 0.0525619547964048 1.45216018508075E-4
171.277127712771 0.0525592245512553 1.45193390654652E-4
171.297129712971 0.0525564948073209 1.45170769305309E-4
171.317131713171 0.0525537655646924 1.45148154459384E-4
171.337133713371 0.0525510368234803 1.45125546116374E-4
171.357135713571 0.0525483085837734 1.45102944275599E-4
171.377137713771 0.0525455808456629 1.450803489364E-4
171.397139713971 0.0525428536092603 1.45057760098281E-4
171.417141714171 0.0525401268746629 1.45035177760636E-4
171.437143714371 0.0525374006419698 1.45012601922867E-4
171.457145714571 0.0525346749112787 1.44990032584369E-4
171.477147714771 0.0525319496826807 1.4496746974448E-4
171.497149714972 0.0525292249562876 1.4494491340271E-4
171.517151715172 0.0525265007321743 1.44922363558264E-4

171.537153715372 0.052523777010469 1.44899820210789E-4
171.557155715572 0.05252105379125 1.44877283359516E-4
171.577157715772 0.0525183310746268 1.44854753003937E-4
171.597159715972 0.0525156088606934 1.44832229143415E-4
171.617161716172 0.05251288714955 1.44809711777365E-4
171.637163716372 0.0525101659412907 1.4478720090515E-4
171.657165716572 0.052507445236024 1.44764696526253E-4
171.677167716772 0.0525047250338346 1.44742198639962E-4
171.697169716972 0.0525020053348307 1.44719707245754E-4
171.717171717172 0.0524992861391089 1.44697222343018E-4
171.737173717372 0.0524965674467663 1.4467474393114E-4
171.757175717572 0.0524938492579043 1.44652272009545E-4
171.777177717772 0.0524911315726189 1.44629806577613E-4
171.797179717972 0.0524884143910105 1.44607347634761E-4
171.817181718172 0.0524856977131735 1.44584895180355E-4
171.837183718372 0.0524829815392108 1.44562449213832E-4
171.857185718572 0.0524802658692189 1.44540009734575E-4
171.877187718772 0.0524775507032961 1.44517576741986E-4
171.897189718972 0.0524748360415393 1.4449515023545E-4
171.917191719172 0.0524721218840582 1.44472730214462E-4
171.937193719372 0.0524694082309322 1.44450316678264E-4
171.957195719572 0.0524666950822714 1.44427909626353E-4
171.977197719772 0.0524639824381677 1.44405509058075E-4
171.997199719972 0.0524612702987335 1.44383114972946E-4
172.017201720172 0.0524585586640508 1.44360727370231E-4
172.037203720372 0.0524558475342192 1.44338346249339E-4
172.057205720572 0.0524531369093432 1.44315971609723E-4
172.077207720772 0.0524504267895251 1.44293603450813E-4
172.097209720972 0.0524477171748496 1.44271241771897E-4
172.117211721172 0.0524450080654171 1.44248886572393E-4
172.137213721372 0.0524422994613468 1.44226537851872E-4
172.157215721572 0.0524395913627078 1.44204195609492E-4
172.177217721772 0.0524368837696057 1.44181859844713E-4
172.197219721972 0.0524341766821509 1.44159530557036E-4
172.217221722172 0.0524314701004302 1.44137207745766E-4
172.237223722372 0.0524287640245447 1.44114891410324E-4
172.257225722572 0.0524260584545841 1.44092581550039E-4
172.277227722772 0.0524233533906587 1.44070278164412E-4
172.297229722972 0.0524206488328591 1.44047981252777E-4
172.317231723172 0.0524179447812837 1.44025690814536E-4
172.337233723372 0.0524152412360321 1.440034068491E-4
172.357235723572 0.0524125381971948 1.43981129355804E-4
172.377237723772 0.0524098356648699 1.43958858334047E-4
172.397239723972 0.0524071336391656 1.4393659378331E-4

172.417241724172 0.052404432120161 1.43914335702835E-4
172.437243724372 0.0524017311079666 1.43892084092123E-4
172.457245724572 0.0523990306026801 1.4386983895057E-4
172.477247724773 0.0523963306043957 1.43847600277541E-4
172.497249724973 0.0523936311132002 1.43825368072343E-4
172.517251725173 0.0523909321292029 1.43803142334466E-4
172.537253725373 0.0523882336524975 1.43780923063273E-4
172.557255725573 0.0523855356831759 1.43758710258112E-4
172.577257725773 0.0523828382213336 1.43736503918361E-4
172.597259725973 0.0523801412670698 1.43714304043427E-4
172.617261726173 0.0523774448204765 1.43692110632659E-4
172.637263726373 0.0523747488816681 1.43669923685589E-4
172.657265726573 0.052372053450726 1.43647743201481E-4
172.677267726773 0.052369358527739 1.43625569179657E-4
172.697269726973 0.0523666641128095 1.4360340161955E-4
172.717271727173 0.0523639702060451 1.43581240520639E-4
172.737273727373 0.0523612768075215 1.43559085882137E-4
172.757275727573 0.0523585839173408 1.4353693770348E-4
172.777277727773 0.052355891535609 1.43514795984128E-4
172.797279727973 0.0523531996624092 1.4349266072336E-4
172.817281728173 0.0523505082978381 1.43470531920562E-4
172.837283728373 0.0523478174420058 1.43448409575235E-4
172.857285728573 0.0523451270949758 1.43426293686491E-4
172.877287728773 0.0523424372568774 1.43404184253988E-4
172.897289728973 0.0523397479277866 1.43382081276943E-4
172.917291729173 0.0523370591077921 1.43359984754679E-4
172.937293729373 0.052334370797008 1.43337894686728E-4
172.957295729573 0.0523316829955101 1.43315811072305E-4
172.977297729773 0.0523289957034032 1.43293733910866E-4
172.997299729973 0.0523263089207655 1.43271663201648E-4
173.017301730173 0.0523236226477197 1.43249598944252E-4
173.037303730373 0.052320936884334 1.43227541137833E-4
173.057305730573 0.0523182516307238 1.43205489781933E-4
173.077307730773 0.0523155668869555 1.43183444875692E-4
173.097309730973 0.0523128826531438 1.43161406418647E-4
173.117311731173 0.0523101989293751 1.43139374410102E-4
173.137313731373 0.0523075157157448 1.43117348849434E-4
173.157315731573 0.05230483301234 1.43095329735955E-4
173.177317731773 0.0523021508192567 1.43073317069047E-4
173.197319731973 0.0522994691365919 1.43051310848102E-4
173.217321732173 0.0522967879644363 1.4302931107246E-4
173.237323732373 0.0522941073028683 1.4300731774136E-4
173.257325732573 0.0522914271520044 1.42985330854353E-4
173.277327732773 0.0522887475119176 1.42963350410635E-4

173.297329732973 0.052286068382708 1.42941376409623E-4
173.317331733173 0.0522833897644556 1.42919408850569E-4
173.337333733373 0.0522807116572696 1.42897447732965E-4
173.357335733573 0.0522780340612437 1.42875493056176E-4
173.377337733773 0.0522753569764458 1.42853544819356E-4
173.397339733973 0.0522726804029792 1.42831603021949E-4
173.417341734173 0.052270004340946 1.42809667663388E-4
173.437343734373 0.0522673287904194 1.4278773874287E-4
173.457345734573 0.052264653751491 1.42765816259745E-4
173.477347734773 0.0522619792242638 1.42743900213453E-4
173.497349734974 0.0522593052088256 1.42721990603312E-4
173.517351735174 0.0522566317052507 1.42700087428528E-4
173.537353735374 0.0522539587136508 1.42678190688615E-4
173.557355735574 0.0522512862340903 1.42656300382698E-4
173.577357735774 0.0522486142666846 1.4263441651032E-4
173.597359735974 0.0522459428115062 1.42612539070673E-4
173.617361736174 0.0522432718686526 1.42590668063155E-4
173.637363736374 0.0522406014382058 1.42568803487037E-4
173.657365736574 0.0522379315202568 1.42546945341661E-4
173.677367736774 0.0522352621148964 1.42525093626371E-4
173.697369736974 0.0522325932222125 1.42503248340485E-4
173.717371737174 0.0522299248422914 1.42481409483308E-4
173.737373737374 0.05222725697522 1.42459577054152E-4
173.757375737574 0.0522245896210877 1.42437751052348E-4
173.777377737774 0.0522219227799796 1.42415931477192E-4
173.797379737974 0.0522192564519891 1.42394118328048E-4
173.817381738174 0.052216590637197 1.42372311604177E-4
173.837383738374 0.0522139253356884 1.42350511304877E-4
173.857385738574 0.052211260547551 1.42328717429464E-4
173.877387738774 0.0522085962728829 1.42306929977341E-4
173.897389738974 0.0522059325117562 1.422851489477E-4
173.917391739174 0.0522032692642544 1.42263374339823E-4
173.937393739374 0.0522006065304898 1.4224160615323E-4
173.957395739574 0.0521979443105146 1.42219844386948E-4
173.977397739774 0.0521952826044266 1.4219808904038E-4
173.997399739974 0.0521926214123039 1.42176340112766E-4
174.017401740174 0.0521899607342544 1.42154597603587E-4
174.037403740374 0.0521873005703405 1.42132861511957E-4
174.057405740574 0.0521846409206461 1.42111131837163E-4
174.077407740774 0.0521819817852689 1.42089408578606E-4
174.097409740974 0.0521793231642848 1.42067691735508E-4
174.117411741174 0.0521766650577748 1.42045981307133E-4
174.137413741374 0.0521740074658312 1.42024277292838E-4
174.157415741574 0.0521713503885267 1.42002579691821E-4

174.177417741774 0.0521686938259517 1.41980888503421E-4
174.197419741974 0.0521660377781845 1.41959203726883E-4
174.217421742174 0.0521633822453086 1.4193752536149E-4
174.237423742374 0.0521607272274036 1.41915853406498E-4
174.257425742574 0.0521580727245551 1.41894187861207E-4
174.277427742774 0.0521554187368466 1.41872528724903E-4
174.297429742974 0.052152765264354 1.41850875996812E-4
174.317431743174 0.0521501123071618 1.41829229676226E-4
174.337433743374 0.0521474598653388 1.41807589762311E-4
174.357435743574 0.0521448079389808 1.41785956254453E-4
174.377437743774 0.0521421565281613 1.41764329151857E-4
174.397439743974 0.0521395056329649 1.41742708453819E-4
174.417441744174 0.0521368552534632 1.41721094159527E-4
174.437443744374 0.052134205389741 1.41699486268279E-4
174.457445744574 0.0521315560418779 1.41677884779329E-4
174.477447744774 0.0521289072099408 1.41656289691829E-4
174.497449744975 0.0521262588940299 1.41634701005201E-4
174.517451745175 0.0521236110942086 1.41613118718568E-4
174.537453745375 0.0521209638105523 1.41591542831153E-4
174.557455745575 0.0521183170431508 1.41569973342292E-4
174.577457745775 0.0521156707920733 1.41548410251157E-4
174.597459745975 0.0521130250573872 1.41526853556903E-4
174.617461746175 0.0521103798391938 1.41505303258964E-4
174.637463746375 0.0521077351375442 1.41483759356363E-4
174.657465746575 0.052105090952522 1.4146222184839E-4
174.677467746775 0.0521024472842116 1.41440690734337E-4
174.697469746975 0.0520998041326888 1.41419166013432E-4
174.717471747175 0.0520971614980222 1.4139764768484E-4
174.737473747375 0.0520945193802782 1.41376135747712E-4
174.757475747575 0.052091877779539 1.41354630201323E-4
174.777477747775 0.052089236695889 1.41333131044972E-4
174.797479747975 0.0520865961293865 1.41311638277739E-4
174.817481748175 0.0520839560801155 1.4129015189892E-4
174.837483748375 0.0520813165481448 1.41268671907683E-4
174.857485748575 0.0520786775335444 1.41247198303206E-4
174.877487748775 0.0520760390363964 1.4122573108477E-4
174.897489748975 0.0520734010567511 1.4120427025139E-4
174.917491749175 0.0520707635947094 1.41182815802499E-4
174.937493749375 0.0520681266503257 1.4116136773715E-4
174.957495749575 0.0520654902236718 1.41139926054537E-4
174.977497749775 0.052062854314815 1.41118490753819E-4
174.997499749975 0.0520602189238448 1.41097061834333E-4
175.017501750175 0.0520575840508076 1.41075639295069E-4
175.037503750375 0.0520549496957854 1.41054223135304E-4

175.057505750575 0.0520523158588505 1.41032813354239E-4
175.077507750775 0.0520496825400595 1.41011409950944E-4
175.097509750975 0.0520470497394961 1.40990012924711E-4
175.117511751175 0.0520444174572115 1.40968622274571E-4
175.137513751375 0.0520417856932879 1.40947237999801E-4
175.157515751575 0.0520391544477911 1.4092586009955E-4
175.177517751775 0.0520365237207857 1.40904488572956E-4
175.197519751975 0.0520338935123302 1.40883123419106E-4
175.217521752175 0.0520312638225014 1.40861764637239E-4
175.237523752375 0.0520286346513697 1.40840412226538E-4
175.257525752575 0.0520260059989934 1.40819066186092E-4
175.277527752775 0.052023377865427 1.40797726514956E-4
175.297529752975 0.0520207502507628 1.40776393212496E-4
175.317531753175 0.0520181231550413 1.40755066277652E-4
175.337533753375 0.0520154965783319 1.40733745709604E-4
175.357535753575 0.052012870520709 1.40712431507571E-4
175.377537753775 0.0520102449822222 1.40691123670569E-4
175.397539753975 0.0520076199629364 1.40669822197741E-4
175.417541754175 0.0520049954629274 1.40648527088317E-4
175.437543754375 0.0520023714822471 1.40627238341334E-4
175.457545754575 0.0519997480209484 1.40605955955838E-4
175.477547754775 0.0519971250791047 1.4058467993104E-4
175.497549754975 0.0519945026567796 1.40563410266072E-4
175.517551755176 0.0519918807540345 1.40542146960049E-4
175.537553755376 0.0519892593709142 1.4052089001195E-4
175.557555755576 0.0519866385074869 1.40499639420947E-4
175.577557755776 0.0519840181638198 1.40478395186202E-4
175.597559755976 0.0519813983399632 1.40457157306739E-4
175.617561756176 0.0519787790359709 1.40435925781612E-4
175.637563756376 0.0519761602519114 1.40414700609996E-4
175.657565756576 0.0519735419878415 1.40393481790969E-4
175.677567756776 0.0519709242438123 1.40372269323562E-4
175.697569756976 0.0519683070198821 1.40351063206868E-4
175.717571757176 0.0519656903161141 1.40329863440018E-4
175.737573757376 0.0519630741325448 1.40308670021925E-4
175.757575757576 0.0519604584692581 1.40287482951891E-4
175.777577757776 0.0519578433262862 1.40266302228795E-4
175.797579757976 0.0519552287036877 1.40245127851731E-4
175.817581758176 0.0519526146015269 1.40223959819843E-4
175.837583758376 0.0519500010198508 1.40202798132128E-4
175.857585758576 0.0519473879587137 1.40181642787649E-4
175.877587758776 0.0519447754181565 1.40160493785357E-4
175.897589758976 0.0519421633982474 1.40139351124425E-4
175.917591759176 0.0519395518990359 1.40118214803878E-4

175.937593759376 0.0519369409205683 1.40097084822708E-4
175.957595759576 0.0519343304628971 1.40075961179966E-4
175.977597759776 0.0519317205260725 1.40054843874679E-4
175.997599759976 0.0519291111101523 1.40033732905936E-4
176.017601760176 0.0519265022151718 1.40012628272647E-4
176.037603760376 0.0519238938411815 1.39991529973842E-4
176.057605760576 0.0519212859882355 1.39970438008584E-4
176.077607760776 0.0519186786563915 1.39949352375962E-4
176.097609760976 0.0519160718456749 1.39928273074804E-4
176.117611761176 0.051913465556155 1.39907200104297E-4
176.137613761376 0.0519108597878615 1.39886133463304E-4
176.157615761576 0.051908254540849 1.39865073150892E-4
176.177617761776 0.0519056498151568 1.39844019166002E-4
176.197619761976 0.0519030456108398 1.39822971507704E-4
176.217621762176 0.0519004419279288 1.39801930174873E-4
176.237623762376 0.0518978387664841 1.39780895166621E-4
176.257625762576 0.0518952361265345 1.39759866481808E-4
176.277627762776 0.0518926340081279 1.39738844119446E-4
176.297629762976 0.0518900324113078 1.39717828078513E-4
176.317631763176 0.0518874313361264 1.39696818358058E-4
176.337633763376 0.0518848307825988 1.3967581495683E-4
176.357635763576 0.0518822307507992 1.39654817874056E-4
176.377637763776 0.0518796312407431 1.39633827108489E-4
176.397639763976 0.0518770322524753 1.39612842659117E-4
176.417641764176 0.0518744337860352 1.39591864524889E-4
176.437643764376 0.0518718358414717 1.39570892704827E-4
176.457645764576 0.0518692384188151 1.39549927197803E-4
176.477647764776 0.0518666415180978 1.3952896800271E-4
176.497649764977 0.0518640451393718 1.39508015118596E-4
176.517651765177 0.0518614492826573 1.39487068544254E-4
176.537653765377 0.0518588539480029 1.39466128278706E-4
176.557655765577 0.0518562591354354 1.39445194320798E-4
176.577657765777 0.0518536648449974 1.39424266669505E-4
176.597659765977 0.0518510710767131 1.39403345323653E-4
176.617661766177 0.0518484778306316 1.39382430282269E-4
176.637663766377 0.0518458851067691 1.39361521544116E-4
176.657665766577 0.0518432929051737 1.39340619108212E-4
176.677667766777 0.0518407012258706 1.39319722973393E-4
176.697669766977 0.0518381100688817 1.39298833138468E-4
176.717671767177 0.0518355194342577 1.3927794960248E-4
176.737673767377 0.0518329293220168 1.39257072364206E-4
176.757675767577 0.0518303397321915 1.39236201422543E-4
176.777677767777 0.0518277506648138 1.39215336776382E-4
176.797679767977 0.0518251621199029 1.39194478424512E-4

176.817681768177 0.0518225740975011 1.39173626365907E-4
176.837683768377 0.0518199865976232 1.39152780599322E-4
176.857685768577 0.051817399620309 1.39131941123713E-4
176.877687768777 0.0518148131655679 1.39111107937788E-4
176.897689768977 0.0518122272334404 1.39090281040512E-4
176.917691769177 0.0518096418239407 1.39069460430633E-4
176.937693769377 0.051807056937113 1.39048646107145E-4
176.957695769577 0.0518044725729464 1.39027838068595E-4
176.977697769777 0.051801888731501 1.39007036314105E-4
176.997699769977 0.051799305412779 1.38986240842328E-4
177.017701770177 0.0517967226168029 1.38965451652084E-4
177.037703770377 0.0517941403436106 1.38944668742316E-4
177.057705770577 0.0517915585932034 1.38923892111672E-4
177.077707770777 0.05178897736561 1.38903121759021E-4
177.097709770977 0.0517863966608518 1.38882357683174E-4
177.117711771177 0.0517838164789432 1.38861599882887E-4
177.137713771377 0.0517812368199026 1.38840848356948E-4
177.157715771577 0.0517786576837507 1.3882010310416E-4
177.177717771777 0.0517760790705108 1.38799364123353E-4
177.197719771977 0.0517735009801838 1.38778631413175E-4
177.217721772177 0.051770923412805 1.38757904972549E-4
177.237723772377 0.0517683463683665 1.38737184800054E-4
177.257725772577 0.0517657698469049 1.38716470894625E-4
177.277727772777 0.0517631938484139 1.38695763254854E-4
177.297729772977 0.0517606183729213 1.38675061879605E-4
177.317731773177 0.0517580434204406 1.3865436676763E-4
177.337733773377 0.0517554689909865 1.38633677917691E-4
177.357735773577 0.0517528950845505 1.38612995328362E-4
177.377737773777 0.0517503217011563 1.38592318998476E-4
177.397739773977 0.0517477488408179 1.3857164892679E-4
177.417741774177 0.0517451765035336 1.38550985111936E-4
177.437743774377 0.0517426046893169 1.38530327552665E-4
177.457745774577 0.0517400333981855 1.38509676247766E-4
177.477747774778 0.0517374626301279 1.38489031195791E-4
177.497749774978 0.0517348923851671 1.38468392395571E-4
177.517751775178 0.051732322663304 1.38447759845758E-4
177.537753775378 0.0517297534645292 1.38427133544924E-4
177.557755775578 0.0517271847888722 1.38406513491952E-4
177.577757775778 0.0517246166363182 1.38385899685372E-4
177.597759775978 0.0517220490068845 1.38365292123969E-4
177.617761776178 0.0517194819005593 1.38344690806296E-4
177.637763776378 0.0517169153173556 1.38324095731107E-4
177.657765776578 0.0517143492572581 1.38303506896926E-4
177.677767776778 0.0517117837202957 1.38282924302635E-4

177.697769776978 0.0517092187064366 1.38262347946629E-4
177.717771777178 0.0517066542156953 1.38241777827673E-4
177.737773777378 0.0517040902480753 1.38221213944446E-4
177.757775777578 0.0517015268035554 1.38200656295427E-4
177.777777777778 0.0516989638821566 1.38180104879438E-4
177.797779777978 0.0516964014838521 1.38159559694913E-4
177.817781778178 0.0516938396086512 1.3813902074058E-4
177.837783778378 0.0516912782565421 1.38118488014995E-4
177.857785778578 0.0516887174275232 1.38097961516799E-4
177.877787778778 0.0516861571215751 1.3807744124449E-4
177.897789778978 0.0516835973387072 1.38056927196794E-4
177.917791779178 0.0516810380788996 1.38036419372208E-4
177.937793779378 0.0516784793421513 1.38015917769376E-4
177.957795779578 0.051675921128438 1.37995422386759E-4
177.977797779778 0.0516733634377677 1.37974933223076E-4
177.997799779978 0.0516708062701198 1.37954450276817E-4
178.017801780178 0.0516682496254847 1.3793397354656E-4
178.037803780378 0.051665693503841 1.37913503030792E-4
178.057805780578 0.0516631379051707 1.37893038728023E-4
178.077807780778 0.0516605828294802 1.37872580636963E-4
178.097809780978 0.0516580282767326 1.37852128755972E-4
178.117811781178 0.0516554742469238 1.37831683083677E-4
178.137813781378 0.051652920740037 1.378112436186E-4
178.157815781578 0.0516503677560537 1.37790810359253E-4
178.177817781778 0.0516478152949526 1.37770383304124E-4
178.197819781978 0.0516452633567109 1.37749962451689E-4
178.217821782178 0.0516427119413145 1.37729547800496E-4
178.237823782378 0.0516401610487444 1.37709139349053E-4
178.257825782578 0.0516376106789706 1.37688737095781E-4
178.277827782778 0.0516350608319776 1.37668341039218E-4
178.297829782978 0.0516325115077356 1.37647951177785E-4
178.317831783178 0.0516299627062293 1.37627567510024E-4
178.337833783378 0.0516274144274198 1.37607190034284E-4
178.357835783578 0.051624866671306 1.37586818749222E-4
178.377837783778 0.0516223194378267 1.37566453653009E-4
178.397839783978 0.0516197727269804 1.37546094744299E-4
178.417841784178 0.051617226538729 1.3752574202145E-4
178.437843784378 0.0516146808730456 1.37505395482913E-4
178.457845784578 0.0516121357298941 1.37485055127063E-4
178.477847784778 0.0516095911092672 1.37464720952509E-4
178.497849784979 0.0516070470111028 1.3744439295742E-4
178.517851785179 0.0516045034353837 1.37424071140325E-4
178.537853785379 0.0516019603820783 1.37403755499638E-4
178.557855785579 0.0515994178511402 1.37383446033656E-4

178.577857785779 0.051596875842547 1.37363142740869E-4
178.597859785979 0.0515943343562593 1.37342845619629E-4
178.617861786179 0.051591793392232 1.37322554668245E-4
178.637863786379 0.0515892529504382 1.37302269885172E-4
178.657865786579 0.0515867130308453 1.37281991268818E-4
178.677867786779 0.0515841736333929 1.37261718817372E-4
178.697869786979 0.0515816347580571 1.37241452529314E-4
178.717871787179 0.0515790964047906 1.37221192402937E-4
178.737873787379 0.0515765585735466 1.37200938436539E-4
178.757875787579 0.0515740212642964 1.37180690628563E-4
178.777877787779 0.0515714844769858 1.37160448977249E-4
178.797879787979 0.0515689482115718 1.37140213480927E-4
178.817881788179 0.0515664124680054 1.37119984137879E-4
178.837883788379 0.0515638772462431 1.37099760946433E-4
178.857885788579 0.0515613425462437 1.37079543904934E-4
178.877887788779 0.0515588083679459 1.37059333011568E-4
178.897889788979 0.0515562747113173 1.37039128264751E-4
178.917891789179 0.0515537415762951 1.3701892966266E-4
178.937893789379 0.0515512089628333 1.36998737203604E-4
178.957895789579 0.0515486768708774 1.36978550885824E-4
178.977897789779 0.0515461453003828 1.36958370707644E-4
178.997899789979 0.0515436142512731 1.36938196667131E-4
179.017901790179 0.0515410837235164 1.36918028762709E-4
179.037903790379 0.0515385537170473 1.36897866992538E-4
179.057905790579 0.0515360242318124 1.36877711354869E-4
179.077907790779 0.0515334952677509 1.36857561847899E-4
179.097909790979 0.051530966824805 1.36837418469848E-4
179.117911791179 0.0515284389029217 1.36817281218973E-4
179.137913791379 0.0515259115020353 1.36797150093434E-4
179.157915791579 0.0515233846220857 1.36777025091433E-4
179.177917791779 0.0515208582630033 1.36756906211098E-4
179.197919791979 0.0515183324247382 1.36736793450716E-4
179.217921792179 0.0515158071072183 1.36716686808395E-4
179.237923792379 0.051513282310377 1.3669658628229E-4
179.257925792579 0.0515107580341503 1.36676491870574E-4
179.277927792779 0.0515082342784657 1.36656403571356E-4
179.297929792979 0.0515057110432667 1.36636321382873E-4
179.317931793179 0.0515031883284835 1.36616245303253E-4
179.337933793379 0.0515006661340278 1.36596175330483E-4
179.357935793579 0.0514981444598493 1.36576111462847E-4
179.377937793779 0.0514956233058674 1.36556053698394E-4
179.397939793979 0.0514931026720059 1.36536002035204E-4
179.417941794179 0.0514905825581999 1.36515956471451E-4
179.437943794379 0.0514880629643601 1.36495917005112E-4

179.457945794579 0.0514855438904208 1.36475883634356E-4
179.477947794779 0.0514830253363097 1.36455856357299E-4
179.49794979498 0.0514805073019471 1.36435835171996E-4
179.51795179518 0.0514779897872357 1.36415820076367E-4
179.53795379538 0.0514754727921154 1.36395811068625E-4
179.55795579558 0.0514729563164949 1.36375808146738E-4
179.57795779578 0.0514704403602944 1.36355811308764E-4
179.59795979598 0.0514679249234395 1.36335820552806E-4
179.61796179618 0.0514654100058375 1.36315835876821E-4
179.63796379638 0.051462895607395 1.36295857278761E-4
179.65796579658 0.0514603817280408 1.36275884756758E-4
179.67796779678 0.0514578683676767 1.36255918308726E-4
179.69796979698 0.0514553555262231 1.3623595793273E-4
179.71797179718 0.051452843203589 1.36216003626744E-4
179.73797379738 0.0514503313996675 1.36196055388617E-4
179.75797579758 0.0514478201143897 1.36176113216499E-4
179.77797779778 0.0514453093476567 1.36156177108306E-4
179.79797979798 0.0514427990993784 1.3613624706202E-4
179.81798179818 0.0514402893694318 1.36116323075364E-4
179.83798379838 0.0514377801577547 1.36096405146548E-4
179.85798579858 0.0514352714642371 1.36076493273397E-4
179.87798779878 0.0514327632887863 1.36056587453878E-4
179.89798979898 0.0514302556312934 1.36036687685829E-4
179.91799179918 0.0514277484916602 1.36016793967175E-4
179.93799379938 0.0514252418697934 1.35996906295878E-4
179.95799579958 0.0514227357655847 1.35977024669784E-4
179.97799779978 0.0514202301789289 1.35957149086762E-4
179.99799979998 0.0514177251097407 1.35937279544842E-4
180.01800180018 0.0514152205578767 1.35917416041592E-4
180.03800380038 0.0514127165232631 1.35897558575133E-4
180.05800580058 0.0514102130057825 1.35877707143243E-4
180.07800780078 0.0514077100053163 1.35857861743688E-4
180.09800980098 0.0514052075217715 1.35838022374439E-4
180.11801180118 0.0514027055550133 1.35818189033137E-4
180.13801380138 0.0514002041049533 1.35798361717791E-4
180.15801580158 0.0513977031714645 1.35778540426105E-4
180.17801780178 0.051395202754428 1.35758725155847E-4
180.19801980198 0.0513927028537509 1.35738915904993E-4
180.21802180218 0.0513902034692779 1.35719112671025E-4
180.23802380238 0.0513877046009279 1.35699315452012E-4
180.25802580258 0.0513852062485633 1.35679524245579E-4
180.27802780278 0.0513827084120726 1.35659739049555E-4
180.29802980298 0.051380211091323 1.35639959861605E-4
180.31803180318 0.0513777142862019 1.35620186679553E-4

180.33803380338 0.0513752179965837 1.35600419501118E-4
180.35803580358 0.0513727222223354 1.35580658323964E-4
180.37803780378 0.0513702269633343 1.35560903145839E-4
180.39803980398 0.0513677322194611 1.35541153964514E-4
180.41804180418 0.0513652379905782 1.35521410777621E-4
180.43804380438 0.0513627442765473 1.35501673582783E-4
180.45804580458 0.0513602510772616 1.35481942377876E-4
180.47804780478 0.0513577583925769 1.35462217160478E-4
180.498049804981 0.0513552662223486 1.35442497928166E-4
180.518051805181 0.0513527745664489 1.35422784678653E-4
180.538053805381 0.0513502834247558 1.35403077409694E-4
180.558055805581 0.0513477927971129 1.35383376118777E-4
180.578057805781 0.0513453026833898 1.35363680803594E-4
180.598059805981 0.0513428130834578 1.35343991461852E-4
180.618061806181 0.0513403239971558 1.35324308091001E-4
180.638063806381 0.0513378354243611 1.35304630688799E-4
180.658065806581 0.0513353473649182 1.35284959252741E-4
180.678067806781 0.0513328598186726 1.35265293780335E-4
180.698069806981 0.0513303727855096 1.35245634269401E-4
180.718071807181 0.0513278862652544 1.35225980717287E-4
180.738073807381 0.0513254002577687 1.35206333121628E-4
180.758075807581 0.0513229147629089 1.35186691480022E-4
180.778077807781 0.051320429780523 1.35167055789996E-4
180.798079807981 0.0513179453104472 1.3514742604899E-4
180.818081808181 0.051315461352539 1.35127802254608E-4
180.838083808381 0.051312977906633 1.35108184404278E-4
180.858085808581 0.0513104949725962 1.35088572495682E-4
180.878087808781 0.0513080125502491 1.35068966526137E-4
180.898089808981 0.0513055306394452 1.35049366493223E-4
180.918091809181 0.0513030492400093 1.3502977239429E-4
180.938093809381 0.0513005683517956 1.35010184226924E-4
180.958095809581 0.0512980879746428 1.3499060198859E-4
180.978097809781 0.0512956081083851 1.34971025676716E-4
180.998099809981 0.0512931287528564 1.3495145528873E-4
181.018101810181 0.0512906499078881 1.3493189082204E-4
181.038103810381 0.051288171573322 1.3491233227414E-4
181.058105810581 0.0512856937489752 1.34892779642325E-4
181.078107810781 0.051283216434687 1.3487323292407E-4
181.098109810981 0.0512807396302859 1.34853692116764E-4
181.118111811181 0.0512782633355965 1.34834157217767E-4
181.138113811381 0.0512757875504398 1.3481462822441E-4
181.158115811581 0.0512733122746499 1.3479510513413E-4
181.178117811781 0.0512708375080532 1.34775587944303E-4
181.198119811981 0.0512683632504609 1.34756076652187E-4

181.218121812181 0.0512658895017042 1.34736571255197E-4
181.238123812381 0.0512634162615979 1.34717071750621E-4
181.258125812581 0.0512609435299578 1.34697578135755E-4
181.278127812781 0.0512584713065973 1.34678090407878E-4
181.298129812981 0.0512559995913358 1.34658608564316E-4
181.318131813181 0.0512535283839909 1.34639132602382E-4
181.338133813381 0.051251057684384 1.3461966251942E-4
181.358135813581 0.0512485874923057 1.34600198312534E-4
181.378137813781 0.0512461178075726 1.34580739979029E-4
181.398139813981 0.0512436486299893 1.34561287516122E-4
181.418141814181 0.0512411799593808 1.34541840921189E-4
181.438143814381 0.0512387117955409 1.3452240019136E-4
181.458145814581 0.0512362441382626 1.34502965323762E-4
181.478147814781 0.0512337769873733 1.34483536315792E-4
181.498149814982 0.0512313103426581 1.34464113164517E-4
181.518151815182 0.0512288442039114 1.34444695867075E-4
181.538153815382 0.051226378570954 1.34425284420815E-4
181.558155815582 0.0512239134435639 1.34405878822751E-4
181.578157815782 0.0512214488215453 1.34386479070101E-4
181.598159815982 0.0512189847046969 1.34367085160045E-4
181.618161816182 0.0512165210927895 1.34347697089539E-4
181.638163816382 0.0512140579856564 1.34328314856036E-4
181.658165816582 0.0512115953830438 1.34308938456305E-4
181.678167816782 0.0512091332847653 1.34289567887642E-4
181.698169816982 0.0512066716905897 1.34270203146995E-4
181.718171817182 0.0512042106003321 1.34250844231677E-4
181.738173817382 0.0512017500137515 1.34231491138559E-4
181.758175817582 0.0511992899306448 1.34212143864813E-4
181.778177817782 0.0511968303507786 1.34192802407373E-4
181.798179817982 0.0511943712739525 1.34173466763433E-4
181.818181818182 0.0511919126999272 1.34154136929882E-4
181.838183818382 0.0511894546284902 1.34134812903822E-4
181.858185818582 0.051186997059415 1.34115494682245E-4
181.878187818782 0.0511845399924781 1.34096182262168E-4
181.898189818982 0.0511820834274439 1.34076875640513E-4
181.918191819182 0.0511796273640824 1.34057574814249E-4
181.938193819382 0.0511771718021947 1.34038279780588E-4
181.958195819582 0.051174716741495 1.34018990536061E-4
181.978197819782 0.051172262181803 1.33999707078031E-4
181.998199819982 0.0511698081228407 1.33980429403092E-4
182.018201820182 0.0511673545643984 1.33961157508376E-4
182.038203820382 0.0511649015062292 1.33941891390725E-4
182.058205820582 0.0511624489481032 1.33922631047115E-4
182.078207820782 0.0511599968897645 1.33903376474318E-4

182.098209820982 0.0511575453309783 1.33884127669274E-4
182.118211821182 0.0511550942715042 1.33864884628878E-4
182.138213821382 0.0511526437110792 1.33845647349852E-4
182.158215821582 0.0511501936494835 1.33826415829257E-4
182.178217821782 0.0511477440864544 1.33807190063815E-4
182.198219821982 0.051145295021741 1.33787970050348E-4
182.218221822182 0.0511428464550865 1.33768755785626E-4
182.238223822382 0.05114039838625 1.3374954726655E-4
182.258225822582 0.0511379508149634 1.33730344489807E-4
182.278227822782 0.0511355037409918 1.33711147452344E-4
182.298229822982 0.0511330571640484 1.33691956150704E-4
182.318231823182 0.0511306110838973 1.3367277058183E-4
182.338233823382 0.0511281655002584 1.33653590742319E-4
182.358235823582 0.0511257204128832 1.33634416629016E-4
182.378237823782 0.0511232758214995 1.33615248238584E-4
182.398239823982 0.0511208317258629 1.33596085567902E-4
182.418241824182 0.0511183881256699 1.3357692861339E-4
182.438243824382 0.0511159450206744 1.33557777371917E-4
182.458245824582 0.0511135024106014 1.33538631840124E-4
182.478247824783 0.0511110602951738 1.33519492014645E-4
182.498249824983 0.0511086186741235 1.33500357892175E-4
182.518251825183 0.0511061775471764 1.33481229469371E-4
182.538253825383 0.0511037369140515 1.33462106742836E-4
182.558255825583 0.0511012967744716 1.33442989709199E-4
182.578257825783 0.0510988571281512 1.33423878365031E-4
182.598259825983 0.0510964179748133 1.33404772706969E-4
182.618261826183 0.0510939793141772 1.33385672731619E-4
182.638263826383 0.0510915411459541 1.3336657843553E-4
182.658265826583 0.0510891034698503 1.33347489815209E-4
182.678267826783 0.0510866662855953 1.33328406867349E-4
182.698269826983 0.0510842295928731 1.33309329588286E-4
182.718271827183 0.0510817933914193 1.33290257974764E-4
182.738273827383 0.0510793576809155 1.33271192023102E-4
182.758275827583 0.0510769224610788 1.33252131729901E-4
182.778277827783 0.0510744877316191 1.33233077091704E-4
182.798279827983 0.051072053492218 1.33214028104836E-4
182.818281828183 0.0510696197425843 1.33194984765835E-4
182.838283828383 0.0510671864824355 1.33175947071309E-4
182.858285828583 0.0510647537114402 1.33156915017484E-4
182.878287828783 0.0510623214293054 1.33137888600889E-4
182.898289828983 0.0510598896357047 1.33118867817789E-4
182.918291829183 0.0510574583303669 1.33099852664887E-4
182.938293829383 0.0510550275129465 1.33080843138305E-4
182.958295829583 0.0510525971831566 1.33061839234621E-4

182.978297829783 0.0510501673406724 1.33042840950123E-4
182.998299829983 0.0510477379851623 1.33023848281042E-4
183.018301830183 0.0510453091163503 1.33004861224049E-4
183.038303830383 0.0510428807338702 1.32985879775109E-4
183.058305830583 0.0510404528374216 1.32966903930703E-4
183.078307830783 0.0510380254266846 1.32947933687163E-4
183.098309830983 0.0510355985013295 1.32928969040741E-4
183.118311831183 0.0510331720610307 1.32910009987726E-4
183.138313831383 0.0510307461054651 1.32891056524428E-4
183.158315831583 0.0510283206343042 1.32872108647115E-4
183.178317831783 0.0510258956472012 1.32853166351912E-4
183.198319831983 0.0510234711438471 1.32834229635242E-4
183.218321832183 0.0510210471238766 1.32815298493091E-4
183.238323832383 0.051018623586983 1.32796372921901E-4
183.258325832583 0.0510162005328037 1.32777452917681E-4
183.278327832783 0.0510137779610021 1.32758538476643E-4
183.298329832983 0.0510113558712509 1.32739629595074E-4
183.318331833183 0.0510089342631992 1.32720726269079E-4
183.338333833383 0.0510065131364907 1.32701828494718E-4
183.358335833583 0.0510040924907872 1.32682936268199E-4
183.378337833783 0.0510016723257436 1.32664049585673E-4
183.398339833983 0.0509992526409941 1.32645168443134E-4
183.418341834183 0.0509968334362016 1.32626292836799E-4
183.438343834383 0.0509944147109987 1.32607422762653E-4
183.458345834583 0.0509919964650338 1.32588558216801E-4
183.478347834783 0.050989578697941 1.32569699195243E-4
183.498349834984 0.0509871614093752 1.32550845694138E-4
183.518351835184 0.0509847445989556 1.32531997709371E-4
183.538353835384 0.0509823282663308 1.32513155237056E-4
183.558355835584 0.0509799124111337 1.32494318273189E-4
183.578357835784 0.0509774970329877 1.32475486813687E-4
183.598359835984 0.0509750821315298 1.32456660854582E-4
183.618361836184 0.0509726677063826 1.32437840391788E-4
183.638363836384 0.0509702537571837 1.32419025421345E-4
183.658365836584 0.050967840283547 1.32400215939102E-4
183.678367836784 0.0509654272850978 1.32381411941005E-4
183.698369836984 0.0509630147614517 1.32362613422919E-4
183.718371837184 0.0509606027122482 1.32343820380903E-4
183.738373837384 0.0509581911370812 1.32325032810659E-4
183.758375837584 0.0509557800355563 1.32306250707982E-4
183.778377837784 0.0509533694073235 1.32287474069014E-4
183.798379837984 0.0509509592519751 1.32268702889449E-4
183.818381838184 0.0509485495691121 1.32249937165051E-4
183.838383838384 0.050946140358343 1.32231176891644E-4

183.858385838584 0.0509437316192791 1.32212422065074E-4
183.878387838784 0.0509413233515266 1.3219367268115E-4
183.898389838984 0.0509389155546852 1.32174928735631E-4
183.918391839184 0.0509365082283496 1.32156190224239E-4
183.938393839384 0.0509341013721214 1.32137457142751E-4
183.958395839584 0.0509316949855912 1.3211872948686E-4
183.978397839784 0.0509292890683632 1.32100007252366E-4
183.998399839984 0.0509268836200227 1.32081290434925E-4
184.018401840184 0.0509244786401385 1.32062579030063E-4
184.038403840384 0.0509220741283405 1.32043873033786E-4
184.058405840584 0.0509196700841842 1.32025172441521E-4
184.078407840784 0.0509172665072579 1.32006477248952E-4
184.098409840984 0.0509148633971498 1.31987787451765E-4
184.118411841184 0.0509124607534368 1.31969103045556E-4
184.138413841384 0.0509100585756928 1.31950424025904E-4
184.158415841584 0.0509076568634938 1.31931750388402E-4
184.178417841784 0.0509052556164173 1.31913082128659E-4
184.198419841984 0.050902854834033 1.31894419242223E-4
184.218421842184 0.0509004545159129 1.31875761724662E-4
184.238423842384 0.0508980546616199 1.31857109571476E-4
184.258425842584 0.0508956552707194 1.31838462778183E-4
184.278427842784 0.0508932563427931 1.31819821340434E-4
184.298429842984 0.0508908578773645 1.31801185253422E-4
184.318431843184 0.050888459874026 1.31782554512886E-4
184.338433843384 0.0508860623323269 1.31763929114225E-4
184.358435843584 0.0508836652518023 1.31745309052732E-4
184.378437843784 0.0508812686320349 1.3172669432407E-4
184.398439843984 0.0508788724725591 1.3170808492353E-4
184.418441844184 0.0508764767729353 1.31689480846605E-4
184.438443844384 0.0508740815326874 1.31670882088507E-4
184.458445844584 0.0508716867513917 1.31652288644857E-4
184.478447844784 0.050869292428572 1.3163370051087E-4
184.498449844985 0.050866898563768 1.31615117681884E-4
184.518451845185 0.0508645051565148 1.31596540153205E-4
184.538453845385 0.0508621122063643 1.3157796792027E-4
184.558455845585 0.0508597197128479 1.3155940097836E-4
184.578457845785 0.0508573276754847 1.3154083932266E-4
184.598459845985 0.0508549360938072 1.31522282948462E-4
184.618461846185 0.0508525449673542 1.3150373185111E-4
184.638463846385 0.0508501542956397 1.31485186025753E-4
184.658465846585 0.0508477640782054 1.31466645467762E-4
184.678467846785 0.0508453743145471 1.31448110172148E-4
184.698469846985 0.0508429850042046 1.31429580134269E-4
184.718471847185 0.0508405961466807 1.31411055349196E-4

184.738473847385 0.0508382077414991 1.31392535812166E-4
184.758475847585 0.0508358197881801 1.31374021518387E-4
184.778477847785 0.0508334322862116 1.31355512462822E-4
184.798479847985 0.0508310452351308 1.31337008640815E-4
184.818481848185 0.0508286586344214 1.31318510047298E-4
184.838483848385 0.050826272483587 1.31300016677359E-4
184.858485848585 0.0508238867821482 1.31281528526221E-4
184.878487848785 0.0508215015295935 1.31263045588855E-4
184.898489848985 0.0508191167254201 1.31244567860307E-4
184.918491849185 0.0508167323691134 1.31226095335531E-4
184.938493849385 0.0508143484601824 1.31207628009664E-4
184.958495849585 0.0508119649981175 1.31189165877702E-4
184.978497849785 0.0508095819823986 1.31170708934563E-4
184.998499849985 0.0508071994125145 1.31152257175234E-4
185.018501850185 0.0508048172879516 1.31133810594685E-4
185.038503850385 0.0508024356081948 1.31115369187875E-4
185.058505850585 0.0508000543727178 1.31096932949682E-4
185.078507850785 0.0507976735810013 1.31078501875036E-4
185.098509850985 0.0507952932325082 1.31060075958734E-4
185.118511851185 0.0507929133267283 1.3104165519578E-4
185.138513851385 0.0507905338631338 1.31023239581046E-4
185.158515851585 0.0507881548411776 1.31004829109255E-4
185.178517851785 0.0507857762603349 1.30986423775305E-4
185.198519851985 0.0507833981200756 1.30968023574057E-4
185.218521852185 0.0507810204198453 1.3094962850018E-4
185.238523852385 0.0507786431591096 1.30931238548506E-4
185.258525852585 0.0507762663373286 1.30912853713822E-4
185.278527852785 0.0507738899539635 1.30894473990927E-4
185.298529852985 0.0507715140084529 1.30876099374448E-4
185.318531853185 0.0507691385002514 1.30857729859135E-4
185.338533853385 0.0507667634288008 1.30839365439643E-4
185.358535853585 0.0507643887935667 1.30821006110812E-4
185.378537853785 0.0507620145939744 1.30802651867173E-4
185.398539853985 0.0507596408294638 1.3078430270337E-4
185.418541854185 0.0507572674994789 1.30765958614084E-4
185.438543854385 0.0507548946034632 1.30747619593989E-4
185.458545854585 0.0507525221408329 1.30729285637555E-4
185.478547854785 0.0507501501110284 1.30710956739438E-4
185.498549854986 0.0507477785134867 1.30692632894269E-4
185.518551855186 0.0507454073476194 1.30674314096488E-4
185.538553855386 0.0507430366128531 1.30656000340651E-4
185.558555855586 0.0507406663086179 1.30637691621341E-4
185.578557855786 0.0507382964343272 1.30619387933015E-4
185.598559855986 0.0507359269894012 1.30601089270188E-4

185.618561856186 0.0507335579732569 1.30582795627346E-4
185.638563856386 0.0507311893853017 1.30564506998909E-4
185.658565856586 0.0507288212249371 1.3054622337925E-4
185.678567856786 0.0507264534915741 1.30527944762817E-4
185.698569856986 0.0507240861846294 1.30509671144104E-4
185.718571857186 0.0507217193035057 1.30491402517501E-4
185.738573857386 0.0507193528475849 1.30473138877239E-4
185.758575857586 0.0507169868162826 1.30454880217808E-4
185.778577857786 0.0507146212089843 1.30436626533473E-4
185.798579857986 0.0507122560250823 1.30418377818546E-4
185.818581858186 0.0507098912639662 1.30400134067328E-4
185.838583858386 0.0507075269250375 1.3038189527421E-4
185.858585858586 0.0507051630076532 1.30363661433241E-4
185.878587858786 0.050702799511224 1.30345432538888E-4
185.898589858986 0.0507004364351203 1.30327208585308E-4
185.918591859186 0.0506980737787218 1.30308989566734E-4
185.938593859386 0.0506957115413895 1.30290775477253E-4
185.958595859586 0.050693349722514 1.30272566311185E-4
185.978597859786 0.0506909883214676 1.3025436206271E-4
185.998599859986 0.0506886273376071 1.30236162725892E-4
186.018601860186 0.0506862667702871 1.30217968294779E-4
186.038603860386 0.0506839066188945 1.30199778763669E-4
186.058605860586 0.0506815468827759 1.30181594126553E-4
186.078607860786 0.0506791875613048 1.30163414377632E-4
186.098609860986 0.0506768286538173 1.30145239510818E-4
186.118611861186 0.0506744701596601 1.3012706952011E-4
186.138613861386 0.0506721120782125 1.30108904399757E-4
186.158615861586 0.0506697544087959 1.30090744143568E-4
186.178617861786 0.0506673971507624 1.30072588745587E-4
186.198619861986 0.0506650403034622 1.30054438199849E-4
186.218621862186 0.0506626838662247 1.30036292500226E-4
186.238623862386 0.0506603278383931 1.30018151640704E-4
186.258625862586 0.0506579722193016 1.30000015615197E-4
186.278627862786 0.0506556170082783 1.29981884417578E-4
186.298629862986 0.0506532622046617 1.299637580418E-4
186.318631863186 0.0506509078077706 1.29945636481666E-4
186.338633863386 0.0506485538169265 1.29927519731004E-4
186.358635863586 0.0506462002314606 1.29909407783719E-4
186.378637863786 0.0506438470506807 1.29891300633534E-4
186.398639863986 0.0506414942739254 1.29873198274416E-4
186.418641864186 0.0506391419004793 1.29855100699917E-4
186.438643864386 0.050636789929672 1.29837007903937E-4
186.458645864586 0.0506344383608126 1.2981891988022E-4
186.478647864787 0.0506320871931969 1.29800836622411E-4

186.498649864987 0.0506297364261287 1.29782758124218E-4
186.518651865187 0.0506273860589056 1.29764684379305E-4
186.538653865387 0.0506250360908417 1.29746615381462E-4
186.558655865587 0.0506226865212217 1.2972855112426E-4
186.578657865787 0.0506203373493425 1.2971049160136E-4
186.598659865987 0.0506179885744805 1.29692436806274E-4
186.618661866187 0.0506156401959423 1.29674386732742E-4
186.638663866387 0.0506132922130006 1.29656341374251E-4
186.658665866587 0.050610944624928 1.29638300724288E-4
186.678667866787 0.050608597431035 1.29620264776631E-4
186.698669866987 0.0506062506305711 1.29602233524595E-4
186.718671867187 0.050603904222818 1.29584206961743E-4
186.738673867387 0.0506015582070367 1.29566185081481E-4
186.758675867587 0.0505992125825128 1.29548167877407E-4
186.778677867787 0.0505968673485009 1.29530155342883E-4
186.798679867987 0.0505945225042642 1.29512147471338E-4
186.818681868187 0.050592178049057 1.29494144256139E-4
186.838683868387 0.0505898339821392 1.29476145690693E-4
186.858685868587 0.0505874903027921 1.29458151768578E-4
186.878687868787 0.0505851470102383 1.2944016248292E-4
186.898689868987 0.0505828041037226 1.29422177827022E-4
186.918691869187 0.0505804615825077 1.29404197794323E-4
186.938693869387 0.0505781194458281 1.29386222378049E-4
186.958695869587 0.0505757776929248 1.29368251571479E-4
186.978697869787 0.0505734363230467 1.29350285367955E-4
186.998699869987 0.0505710953354075 1.29332323760551E-4
187.018701870187 0.0505687547292614 1.29314366742654E-4
187.038703870387 0.0505664145038247 1.29296414307361E-4
187.058705870587 0.0505640746583245 1.29278466447857E-4
187.078707870787 0.0505617351919864 1.29260523157317E-4
187.098709870987 0.0505593961040161 1.29242584428766E-4
187.118711871187 0.0505570573936802 1.29224650255697E-4
187.138713871387 0.0505547190601396 1.29206720630796E-4
187.158715871587 0.0505523811026203 1.29188795547251E-4
187.178717871787 0.0505500435203484 1.29170874998253E-4
187.198719871987 0.0505477063125224 1.29152958976781E-4
187.218721872187 0.050545369478344 1.29135047475848E-4
187.238723872387 0.0505430330170105 1.29117140488427E-4
187.258725872587 0.0505406969277238 1.29099238007536E-4
187.278727872787 0.0505383612096755 1.29081340026112E-4
187.298729872987 0.0505360258620656 1.2906344653716E-4
187.318731873187 0.0505336908840738 1.29045557533533E-4
187.338733873387 0.0505313562748871 1.29027673008143E-4
187.358735873587 0.0505290220336996 1.29009792953956E-4

187.378737873787 0.0505266881596837 1.28991917363776E-4
187.398739873987 0.0505243546520155 1.28974046230438E-4
187.418741874187 0.050522021509884 1.28956179546879E-4
187.438743874387 0.05051968873243 1.28938317305668E-4
187.458745874587 0.0505173563188489 1.28920459499795E-4
187.478747874788 0.0505150242682955 1.28902606121942E-4
187.498749874988 0.050512692579939 1.28884757164903E-4
187.518751875188 0.050510361252935 1.28866912621373E-4
187.538753875388 0.0505080302864379 1.28849072484036E-4
187.558755875588 0.0505056996796085 1.28831236745632E-4
187.578757875788 0.0505033694315883 1.28813405398753E-4
187.598759875988 0.0505010395415313 1.2879557843609E-4
187.618761876188 0.0504987100085833 1.28777755850275E-4
187.638763876388 0.0504963808318897 1.28759937633937E-4
187.658765876588 0.0504940520105727 1.28742123779533E-4
187.678767876788 0.0504917235437844 1.2872431427975E-4
187.698769876988 0.050489395430655 1.2870650912711E-4
187.718771877188 0.0504870676703113 1.28688708314112E-4
187.738773877388 0.0504847402618767 1.28670911833231E-4
187.758775877588 0.0504824132044796 1.28653119676982E-4
187.778777877788 0.0504800864972384 1.28635331837811E-4
187.798779877988 0.0504777601392805 1.28617548308228E-4
187.818781878188 0.0504754341297047 1.28599769080534E-4
187.838783878388 0.0504731084676311 1.28581994147191E-4
187.858785878588 0.0504707831521693 1.28564223500585E-4
187.878787878788 0.0504684581824161 1.28546457133007E-4
187.898789878988 0.0504661335574825 1.2852869503686E-4
187.918791879188 0.0504638092764721 1.28510937204491E-4
187.938793879388 0.0504614853384621 1.28493183628049E-4
187.958795879588 0.0504591617425553 1.28475434299882E-4
187.978797879788 0.0504568384878567 1.28457689212357E-4
187.998799879988 0.050454515573422 1.28439948357467E-4
188.018801880188 0.050452192998372 1.28422211727706E-4
188.038803880388 0.0504498707617564 1.28404479315025E-4
188.058805880588 0.0504475488626639 1.2838675111168E-4
188.078807880788 0.0504452273001728 1.28369027109847E-4
188.098809880988 0.0504429060733326 1.28351307301483E-4
188.118811881188 0.0504405851812504 1.28333591678994E-4
188.138813881388 0.0504382646229538 1.28315880234176E-4
188.158815881588 0.0504359443975254 1.28298172959251E-4
188.178817881788 0.0504336245040138 1.28280469846182E-4
188.198819881988 0.050431304941474 1.28262770886986E-4
188.218821882188 0.0504289857089698 1.28245076073747E-4
188.238823882388 0.0504266668055324 1.28227385398307E-4

188.258825882588 0.0504243482302273 1.28209698852768E-4
188.278827882788 0.0504220299820797 1.28192016428931E-4
188.298829882988 0.0504197120601323 1.28174338118732E-4
188.318831883188 0.0504173944634262 1.28156663914098E-4
188.338833883388 0.0504150771909835 1.28138993806813E-4
188.358835883588 0.0504127602418456 1.28121327788812E-4
188.378837883788 0.0504104436150398 1.28103665851925E-4
188.398839883988 0.0504081273095861 1.28086007987929E-4
188.418841884188 0.0504058113244895 1.28068354188492E-4
188.438843884388 0.0504034956587916 1.28050704445559E-4
188.458845884588 0.0504011803114806 1.28033058750676E-4
188.478847884788 0.0503988652815817 1.28015417095669E-4
188.498849884989 0.0503965505681033 1.27997779472244E-4
188.518851885189 0.0503942361700441 1.27980145872032E-4
188.538853885389 0.0503919220863897 1.2796251628657E-4
188.558855885589 0.0503896083161597 1.27944890707655E-4
188.578857885789 0.0503872948583427 1.27927269126851E-4
188.598859885989 0.0503849817119175 1.27909651535651E-4
188.618861886189 0.0503826688758791 1.27892037925674E-4
188.638863886389 0.0503803563492059 1.27874428288417E-4
188.658865886589 0.0503780441308869 1.27856822615457E-4
188.678867886789 0.0503757322198953 1.27839220898258E-4
188.698869886989 0.0503734206151942 1.27821623128208E-4
188.718871887189 0.0503711093157668 1.27804029296852E-4
188.738873887389 0.0503687983205708 1.27786439395541E-4
188.758875887589 0.0503664876285711 1.27768853415687E-4
188.778877887789 0.0503641772387352 1.27751271348724E-4
188.798879887989 0.0503618671500113 1.27733693185943E-4
188.818881888189 0.0503595573613524 1.27716118918672E-4
188.838883888389 0.050357247871719 1.27698548538302E-4
188.858885888589 0.0503549386800427 1.27680982036004E-4
188.878887888789 0.0503526297852694 1.27663419403065E-4
188.898889888989 0.0503503211863509 1.27645860630817E-4
188.918891889189 0.0503480128822148 1.27628305710412E-4
188.938893889389 0.0503457048717863 1.27610754632985E-4
188.958895889589 0.0503433971539958 1.27593207389718E-4
188.978897889789 0.0503410897277884 1.27575663971904E-4
188.998899889989 0.0503387825920694 1.27558124370537E-4
189.018901890189 0.0503364757457574 1.27540588576716E-4
189.038903890389 0.0503341691877719 1.27523056581552E-4
189.058905890589 0.0503318629170218 1.27505528376075E-4
189.078907890789 0.0503295569324171 1.27488003951328E-4
189.098909890989 0.0503272512328808 1.27470483298457E-4
189.118911891189 0.0503249458172683 1.27452966408095E-4

189.138913891389 0.0503226406845332 1.27435453271628E-4
189.158915891589 0.0503203358335129 1.27417943879558E-4
189.178917891789 0.0503180312631451 1.27400438223156E-4
189.198919891989 0.0503157269723099 1.27382936293257E-4
189.218921892189 0.0503134229598751 1.27365438080606E-4
189.238923892389 0.0503111192247106 1.27347943575966E-4
189.258925892589 0.0503088157657179 1.27330452770345E-4
189.278927892789 0.0503065125817584 1.2731296565445E-4
189.298929892989 0.0503042096717148 1.27295482219151E-4
189.318931893189 0.0503019070344449 1.27278002455133E-4
189.338933893389 0.0502996046688034 1.27260526353062E-4
189.358935893589 0.0502973025736436 1.27243053903595E-4
189.378937893789 0.0502950007478441 1.27225585097585E-4
189.398939893989 0.0502926991902475 1.27208119925616E-4
189.418941894189 0.050290397899692 1.27190658378239E-4
189.438943894389 0.0502880968750422 1.27173200446213E-4
189.458945894589 0.0502857961151105 1.27155746119901E-4
189.478947894789 0.0502834956187504 1.27138295389982E-4
189.49894989499 0.0502811953848074 1.27120848247078E-4
189.51895189519 0.0502788954120905 1.27103404681536E-4
189.53895389539 0.0502765956994366 1.27085964683921E-4
189.55895589559 0.0502742962456597 1.27068528244624E-4
189.57895789579 0.0502719970496004 1.27051095354245E-4
189.59895989599 0.0502696981100436 1.27033666002962E-4
189.61896189619 0.0502673994258275 1.27016240181361E-4
189.63896389639 0.0502651009957435 1.26998817879678E-4
189.65896589659 0.0502628028185989 1.26981399088273E-4
189.67896789679 0.0502605048932034 1.26963983797524E-4
189.69896989699 0.0502582072183388 1.26946571997606E-4
189.71897189719 0.0502559097928149 1.26929163678907E-4
189.73897389739 0.0502536126154075 1.26911758831561E-4
189.75897589759 0.0502513156849233 1.2689435744594E-4
189.77897789779 0.050249019000123 1.26876959512069E-4
189.79897989799 0.050246722559779 1.26859565020069E-4
189.81898189819 0.0502444263626776 1.26842173960167E-4
189.83898389839 0.0502421304076008 1.26824786322561E-4
189.85898589859 0.0502398346933053 1.26807402097261E-4
189.87898789879 0.0502375392185452 1.26790021274263E-4
189.89898989899 0.0502352439820942 1.26772643843712E-4
189.91899189919 0.0502329489827097 1.26755269795635E-4
189.93899389939 0.0502306542191192 1.26737899119834E-4
189.95899589959 0.0502283596901045 1.26720531806528E-4
189.97899789979 0.0502260653943876 1.26703167845487E-4
189.99899989999 0.0502237713307249 1.26685807226743E-4

190.01900190019 0.0502214774978429 1.26668449940106E-4
190.03900390039 0.050219183894454 1.26651095975284E-4
190.05900590059 0.0502168905193188 1.26633745322351E-4
190.07900790079 0.0502145973711548 1.26616397971062E-4
190.09900990099 0.0502123044486885 1.2659905391124E-4
190.11901190119 0.0502100117506223 1.26581713132531E-4
190.13901390139 0.0502077192756743 1.26564375624706E-4
190.15901590159 0.0502054270225646 1.26547041377548E-4
190.17901790179 0.050203134989991 1.26529710380683E-4
190.19901990199 0.0502008431766642 1.26512382623832E-4
190.21902190219 0.0501985515812566 1.26495058096433E-4
190.23902390239 0.0501962602024918 1.26477736788315E-4
190.25902590259 0.0501939690390502 1.26460418688982E-4
190.27902790279 0.0501916780896188 1.26443103787994E-4
190.29902990299 0.0501893873528734 1.26425792074829E-4
190.31903190319 0.0501870968274987 1.26408483539035E-4
190.33903390339 0.0501848065121665 1.26391178170069E-4
190.35903590359 0.050182516405554 1.2637387595743E-4
190.37903790379 0.050180226506317 1.26356576890459E-4
190.39903990399 0.0501779368131278 1.26339280958626E-4
190.41904190419 0.0501756473246573 1.26321988151397E-4
190.43904390439 0.0501733580395256 1.26304698457853E-4
190.45904590459 0.0501710689564196 1.26287411867587E-4
190.47904790479 0.0501687800739518 1.26270128369632E-4
190.499049904991 0.0501664913908025 1.26252847953539E-4
190.519051905191 0.0501642029055806 1.26235570608321E-4
190.539053905391 0.0501619146169292 1.26218296323254E-4
190.559055905591 0.0501596265234986 1.26201025087674E-4
190.579057905791 0.0501573386238861 1.26183756890518E-4
190.599059905991 0.0501550509167339 1.26166491721067E-4
190.619061906191 0.050152763400643 1.26149229568296E-4
190.639063906391 0.050150476074259 1.26131970421518E-4
190.659065906591 0.0501481889361649 1.2611471426958E-4
190.679067906791 0.0501459019849766 1.2609746110158E-4
190.699069906991 0.0501436152192958 1.26080210906512E-4
190.719071907191 0.0501413286377055 1.26062963673234E-4
190.739073907391 0.0501390422388538 1.26045719391099E-4
190.759075907591 0.0501367560212708 1.26028478048574E-4
190.779077907791 0.0501344699835588 1.26011239634673E-4
190.799079907991 0.0501321841243217 1.25994004138427E-4
190.819081908191 0.0501298984421145 1.25976771548504E-4
190.839083908391 0.0501276129355246 1.25959541853816E-4
190.859085908591 0.050125327603123 1.2594231504316E-4
190.879087908791 0.0501230424434504 1.25925091105106E-4

190.899089908991 0.0501207574550973 1.25907870028603E-4
190.919091909191 0.0501184726365924 1.2589065180214E-4
190.939093909391 0.0501161879865385 1.25873436414768E-4
190.959095909591 0.0501139035034437 1.25856223854827E-4
190.979097909791 0.0501116191858513 1.25839014110928E-4
190.999099909991 0.0501093350323236 1.25821807171828E-4
191.019101910191 0.0501070510413816 1.25804603025978E-4
191.039103910391 0.0501047672115742 1.25787401662042E-4
191.059105910591 0.0501024835413876 1.25770203068217E-4
191.079107910791 0.0501002000294009 1.25753007233403E-4
191.099109910991 0.0500979166741122 1.25735814145895E-4
191.119111911191 0.0500956334740276 1.25718623794051E-4
191.139113911391 0.0500933504276637 1.25701436166314E-4
191.159115911591 0.0500910675335204 1.25684251251002E-4
191.179117911791 0.050088784790128 1.25667069036671E-4
191.199119911991 0.0500865021959389 1.25649889511291E-4
191.219121912191 0.0500842197494994 1.25632712663544E-4
191.239123912391 0.0500819374492549 1.25615538481358E-4
191.259125912591 0.0500796552937147 1.25598366953143E-4
191.279127912791 0.050077373281352 1.25581198067045E-4
191.299129912991 0.0500750914106623 1.2556403181138E-4
191.319131913191 0.0500728096800802 1.25546868174011E-4
191.339133913391 0.0500705280881182 1.2552970714339E-4
191.359135913591 0.0500682466332016 1.25512548707319E-4
191.379137913791 0.0500659653138106 1.25495392854016E-4
191.399139913991 0.0500636841284131 1.25478239571612E-4
191.419141914191 0.0500614030754386 1.25461088847951E-4
191.439143914391 0.0500591221533436 1.25443940671087E-4
191.459145914591 0.0500568413605541 1.25426795028847E-4
191.479147914792 0.0500545606955169 1.25409651909221E-4
191.499149914992 0.0500522801566728 1.25392511300156E-4
191.519151915192 0.0500499997424682 1.25375373189647E-4
191.539153915392 0.0500477194512965 1.25358237565293E-4
191.559155915592 0.0500454392815705 1.25341104414845E-4
191.579157915792 0.0500431592317536 1.25323973726438E-4
191.599159915992 0.0500408793002108 1.25306845487473E-4
191.619161916192 0.0500385994853782 1.25289719685888E-4
191.639163916392 0.050036319785638 1.2527259630922E-4
191.659165916592 0.0500340401994097 1.25255475345293E-4
191.679167916792 0.0500317607250693 1.25238356781607E-4
191.699169916992 0.0500294813610016 1.25221240605733E-4
191.719171917192 0.0500272021056144 1.25204126805419E-4
191.739173917392 0.0500249229572819 1.25187015368165E-4
191.759175917592 0.0500226439143624 1.25169906281357E-4

191.779177917792 0.0500203649752488 1.25152799532645E-4
191.799179917992 0.0500180861382955 1.25135695109392E-4
191.819181918192 0.0500158074018649 1.25118592999029E-4
191.839183918392 0.0500135287643277 1.25101493189054E-4
191.859185918592 0.0500112502240186 1.25084395666695E-4
191.879187918792 0.0500089717792971 1.25067300419374E-4
191.899189918992 0.0500066934285171 1.25050207434476E-4
191.919191919192 0.0500044151700029 1.25033116699167E-4
191.939193919392 0.0500021370020951 1.25016028200739E-4
191.959195919592 0.049999858923119 1.24998941926378E-4
191.979197919792 0.0499975809314118 1.24981857863358E-4
191.999199919992 0.0499953030252797 1.2496477599873E-4
192.019201920192 0.0499930252030696 1.24947696319851E-4
192.039203920392 0.0499907474630583 1.24930618813561E-4
192.059205920592 0.0499884698035728 1.24913543467077E-4
192.079207920792 0.0499861922229202 1.24896470267475E-4
192.099209920992 0.04998391471939 1.24879399201701E-4
192.119211921192 0.0499816372912655 1.24862330256661E-4
192.139213921392 0.0499793599368684 1.24845263419551E-4
192.159215921592 0.0499770826544439 1.24828198677002E-4
192.179217921792 0.0499748054423085 1.24811136016181E-4
192.199219921992 0.0499725282987163 1.24794075423795E-4
192.219221922192 0.0499702512219321 1.24777016886633E-4
192.239223922392 0.0499679742102625 1.24759960391802E-4
192.259225922592 0.0499656972619116 1.2474290592565E-4
192.279227922792 0.0499634203751616 1.24725853475109E-4
192.299229922992 0.0499611435482761 1.2470880302698E-4
192.319231923192 0.0499588667795065 1.24691754567978E-4
192.339233923392 0.049956590067061 1.24674708084496E-4
192.359235923592 0.0499543134092122 1.24657663563418E-4
192.379237923792 0.0499520368041666 1.24640620991135E-4
192.399239923992 0.0499497602501739 1.24623580354367E-4
192.419241924192 0.0499474837454554 1.24606541639627E-4
192.439243924392 0.049945207288219 1.24589504833331E-4
192.459245924592 0.0499429308766855 1.24572469921999E-4
192.479247924792 0.0499406545090603 1.24555436892039E-4
192.499249924993 0.0499383781835474 1.24538405729852E-4
192.519251925193 0.0499361018983647 1.2452137642195E-4
192.539253925393 0.0499338256516772 1.24504348954455E-4
192.559255925593 0.0499315494416827 1.24487323313737E-4
192.579257925793 0.0499292732665885 1.24470299486242E-4
192.599259925993 0.0499269971245571 1.24453277458089E-4
192.619261926193 0.0499247210137741 1.2443625721557E-4
192.639263926393 0.0499224449323976 1.24419238744782E-4

192.659265926593 0.0499201688786225 1.24402222032096E-4
192.679267926793 0.0499178928505506 1.24385207063196E-4
192.699269926993 0.0499156168463983 1.24368193824627E-4
192.719271927193 0.0499133408642915 1.24351182302263E-4
192.739273927393 0.0499110649023664 1.24334172482059E-4
192.759275927593 0.0499087889587933 1.24317164350229E-4
192.779277927793 0.0499065130316905 1.24300157892607E-4
192.799279927993 0.0499042371191957 1.24283153095172E-4
192.819281928193 0.049901961219441 1.24266149943872E-4
192.839283928393 0.0498996853305264 1.24249148424415E-4
192.859285928593 0.0498974094505905 1.24232148522806E-4
192.879287928793 0.0498951335777386 1.24215150224805E-4
192.899289928993 0.0498928577100729 1.24198153516153E-4
192.919291929193 0.0498905818457062 1.24181158382679E-4
192.939293929393 0.0498883059827284 1.24164164810043E-4
192.959295929593 0.0498860301192404 1.24147172783991E-4
192.979297929793 0.0498837542533253 1.24130182290146E-4
192.999299929993 0.0498814783830448 1.24113193313971E-4
193.019301930193 0.0498792025065197 1.24096205841381E-4
193.039303930393 0.0498769266217835 1.2407921985764E-4
193.059305930593 0.0498746507269161 1.24062235348367E-4
193.079307930793 0.049872374819988 1.24045252299113E-4
193.099309930993 0.0498700988990513 1.24028270695299E-4
193.119311931193 0.0498678229621614 1.24011290522375E-4
193.139313931393 0.0498655470073536 1.23994311765648E-4
193.159315931593 0.0498632710326849 1.2397733441059E-4
193.179317931793 0.0498609950361881 1.23960358442498E-4
193.199319931993 0.0498587190158831 1.23943383846581E-4
193.219321932193 0.0498564429698161 1.23926410608246E-4
193.239323932393 0.049854166896 1.23909438712658E-4
193.259325932593 0.0498518907924485 1.23892468144995E-4
193.279327932793 0.0498496146571771 1.23875498890451E-4
193.299329932993 0.0498473384881857 1.23858530934113E-4
193.319331933193 0.0498450622834851 1.2384156426115E-4
193.339333933393 0.0498427860410737 1.23824598856649E-4
193.359335933593 0.0498405097589436 1.23807634705651E-4
193.379337933793 0.0498382334350547 1.23790671792965E-4
193.399339933993 0.0498359570674274 1.23773710103855E-4
193.419341934193 0.0498336806540096 1.23756749623053E-4
193.439343934393 0.0498314041927737 1.23739790335475E-4
193.459345934593 0.0498291276817039 1.23722832226135E-4
193.479347934793 0.0498268511187403 1.23705875279719E-4
193.499349934994 0.0498245745018592 1.23688919481191E-4
193.519351935194 0.0498222978289935 1.23671964815198E-4

193.539353935394 0.0498200210980826 1.23655011266644E-4
193.559355935594 0.049817744307074 1.23638058819683E-4
193.579357935794 0.0498154674539064 1.23621107459633E-4
193.599359935994 0.0498131905365075 1.23604157170919E-4
193.619361936194 0.0498109135527942 1.23587207938097E-4
193.639363936394 0.049808636500698 1.23570259745836E-4
193.659365936594 0.0498063593781065 1.23553312578483E-4
193.679367936794 0.0498040821829581 1.23536366420773E-4
193.699369936994 0.049801804913127 1.23519421256962E-4
193.719371937194 0.0497995275665412 1.2350247707172E-4
193.739373937394 0.04979725014106 1.23485533849204E-4
193.759375937594 0.0497949726346059 1.23468591574053E-4
193.779377937794 0.0497926950450289 1.23451650230368E-4
193.799379937994 0.0497904173702186 1.23434709802554E-4
193.819381938194 0.0497881396080436 1.23417770274862E-4
193.839383938394 0.049785861756359 1.23400831631451E-4
193.859385938594 0.0497835838130428 1.23383893856656E-4
193.879387938794 0.0497813057759489 1.23366956934638E-4
193.899389938994 0.0497790276429182 1.23350020849464E-4
193.919391939194 0.0497767494117903 1.23333085585204E-4
193.939393939394 0.0497744710804024 1.23316151125911E-4
193.959395939594 0.0497721926466045 1.23299217455739E-4
193.979397939794 0.0497699141082128 1.232822845586E-4
193.999399939994 0.0497676354630501 1.23265352418457E-4
194.019401940194 0.0497653567089325 1.23248421019233E-4
194.039403940394 0.0497630778436721 1.23231490344825E-4
194.059405940594 0.0497607988650801 1.23214560379127E-4
194.079407940794 0.0497585197709401 1.2319763110584E-4
194.099409940994 0.0497562405590638 1.23180702508874E-4
194.119411941194 0.0497539612272461 1.23163774572024E-4
194.139413941394 0.0497516817732448 1.23146847278812E-4
194.159415941594 0.049749402194863 1.23129920613107E-4
194.179417941794 0.0497471224898584 1.23112994558442E-4
194.199419941994 0.0497448426560209 1.23096069098597E-4
194.219421942194 0.0497425626910922 1.23079144217002E-4
194.239423942394 0.0497402825928165 1.23062219897111E-4
194.259425942594 0.0497380023589891 1.23045296122761E-4
194.279427942794 0.0497357219872992 1.23028372877009E-4
194.299429942994 0.0497334414755368 1.23011450143668E-4
194.319431943194 0.049731160821404 1.22994527905904E-4
194.339433943394 0.0497288800226449 1.22977606147199E-4
194.359435943594 0.0497265990769779 1.22960684850851E-4
194.379437943794 0.0497243179821302 1.22943764000231E-4
194.399439943994 0.049722036735801 1.22926843578509E-4

194.419441944194 0.0497197553356924 1.22909923568879E-4
194.439443944394 0.0497174737795199 1.22893003954644E-4
194.459445944594 0.0497151920649738 1.22876084718926E-4
194.479447944794 0.0497129101897337 1.22859165844774E-4
194.499449944995 0.0497106281514972 1.22842247315374E-4
194.519451945195 0.0497083459479423 1.22825329113778E-4
194.539453945395 0.0497060635767319 1.22808411222926E-4
194.559455945595 0.0497037810355496 1.22791493625924E-4
194.579457945795 0.0497014983220303 1.2277457630552E-4
194.599459945995 0.0496992154338398 1.22757659244697E-4
194.619461946195 0.0496969323686331 1.22740742426367E-4
194.639463946395 0.0496946491240622 1.22723825833424E-4
194.659465946595 0.0496923656977642 1.22706909448658E-4
194.679467946795 0.0496900820873513 1.22689993254681E-4
194.699469946995 0.0496877982904708 1.22673077234374E-4
194.719471947195 0.0496855143047294 1.22656161370321E-4
194.739473947395 0.0496832301277543 1.22639245645264E-4
194.759475947595 0.0496809457571512 1.22622330041795E-4
194.779477947795 0.0496786611905307 1.22605414542545E-4
194.799479947995 0.0496763764254783 1.2258849912997E-4
194.819481948195 0.0496740914595906 1.22571583786612E-4
194.839483948395 0.0496718062904702 1.22554668495064E-4
194.859485948595 0.0496695209156724 1.22537753237575E-4
194.879487948795 0.0496672353327886 1.22520837996672E-4
194.899489948995 0.0496649495393811 1.22503922754666E-4
194.919491949195 0.0496626635330173 1.22487007493919E-4
194.939493949395 0.0496603773112522 1.22470092196705E-4
194.959495949595 0.0496580908716328 1.22453176845245E-4
194.979497949795 0.049655804211729 1.22436261421937E-4
194.999499949995 0.0496535173290529 1.22419345908759E-4
195.019501950195 0.0496512302211562 1.22402430287986E-4
195.039503950395 0.049648942885544 1.22385514541558E-4
195.059505950595 0.0496466553197636 1.22368598651731E-4
195.079507950795 0.0496443675213137 1.22351682600412E-4
195.099509950995 0.0496420794877322 1.223347663698E-4
195.119511951195 0.0496397912164933 1.22317849941635E-4
195.139513951395 0.0496375027051026 1.22300933297892E-4
195.159515951595 0.0496352139510689 1.22284016420577E-4
195.179517951795 0.0496329249518627 1.22267099291421E-4
195.199519951995 0.0496306357049816 1.22250181892363E-4
195.219521952195 0.0496283462078853 1.22233264205066E-4
195.239523952395 0.049626056458033 1.22216346211198E-4
195.259525952595 0.049623766452907 1.22199427892605E-4
195.279527952795 0.0496214761899653 1.22182509230959E-4

195.299529952995 0.0496191856666493 1.22165590207817E-4
195.319531953195 0.049616894880419 1.2214867080488E-4
195.339533953395 0.0496146038286854 1.22131751003496E-4
195.359535953595 0.0496123125088925 1.22114830785261E-4
195.379537953795 0.0496100209184941 1.22097910131852E-4
195.399539953995 0.0496077290548763 1.22080989024452E-4
195.419541954195 0.0496054369154669 1.22064067444561E-4
195.439543954395 0.0496031444976786 1.22047145373572E-4
195.459545954595 0.0496008517989104 1.22030222792786E-4
195.479547954795 0.0495985588165552 1.22013299683465E-4
195.499549954996 0.0495962655480084 1.21996376026897E-4
195.519551955196 0.0495939719906542 1.21979451804295E-4
195.539553955396 0.0495916781418734 1.21962526996852E-4
195.559555955596 0.0495893839990392 1.21945601585715E-4
195.579557955796 0.0495870895595155 1.21928675551966E-4
195.599559955996 0.0495847948206361 1.21911748876478E-4
195.619561956196 0.049582499779797 1.21894821540584E-4
195.639563956396 0.0495802044343226 1.21877893525098E-4
195.659565956596 0.0495779087815705 1.21860964811088E-4
195.679567956796 0.0495756128188549 1.21844035379307E-4
195.699569956996 0.0495733165435097 1.21827105210663E-4
195.719571957196 0.049571019952874 1.21810174286107E-4
195.739573957396 0.0495687230442432 1.21793242586276E-4
195.759575957596 0.0495664258149443 1.21776310092049E-4
195.779577957796 0.0495641282622772 1.21759376784109E-4
195.799579957996 0.0495618303835463 1.21742442643185E-4
195.819581958196 0.0495595321760177 1.21725507649726E-4
195.839583958396 0.0495572336369886 1.2170857178442E-4
195.859585958596 0.0495549347637612 1.21691635028E-4
195.879587958796 0.0495526355535975 1.21674697360907E-4
195.899589958996 0.0495503360037453 1.21657758763489E-4
195.919591959196 0.049548036111482 1.21640819216317E-4
195.939593959396 0.0495457358740472 1.21623878699692E-4
195.959595959596 0.0495434352887081 1.21606937194126E-4
195.979597959796 0.0495411343527017 1.21589994679916E-4
195.999599959996 0.0495388330632568 1.21573051137305E-4
196.019601960196 0.049536531417607 1.21556106546582E-4
196.039603960396 0.0495342294129643 1.2153916088788E-4
196.059605960596 0.0495319270465694 1.21522214141555E-4
196.079607960796 0.0495296243155856 1.215052662874E-4
196.099609960996 0.04952732121728 1.21488317305979E-4
196.119611961196 0.0495250177487974 1.21471367176962E-4
196.139613961396 0.0495227139073578 1.21454415880584E-4
196.159615961596 0.0495204096901256 1.21437463396677E-4

196.179617961796 0.049518105094289 1.21420509705254E-4
196.199619961996 0.0495158001170303 1.21403554786294E-4
196.219621962196 0.0495134947554801 1.21386598619404E-4
196.239623962396 0.0495111890068252 1.21369641184612E-4
196.259625962596 0.0495088828682342 1.21352682461818E-4
196.279627962796 0.0495065763368174 1.21335722430504E-4
196.299629962996 0.0495042694097307 1.21318761070493E-4
196.319631963196 0.0495019620841088 1.21301798361461E-4
196.339633963396 0.0494996543570653 1.21284834282939E-4
196.359635963596 0.0494973462257355 1.21267868814621E-4
196.379637963796 0.0494950376872031 1.21250901935831E-4
196.399639963996 0.0494927287386217 1.21233933626418E-4
196.419641964196 0.0494904193770577 1.21216963865595E-4
196.439643964396 0.0494881095996215 1.21199992632907E-4
196.459645964596 0.0494857994033888 1.21183019907653E-4
196.479647964797 0.0494834887854301 1.21166045669099E-4
196.499649964997 0.0494811777428646 1.21149069896882E-4
196.519651965197 0.0494788662726977 1.21132092569806E-4
196.539653965397 0.0494765543720432 1.21115113667483E-4
196.559655965597 0.0494742420379261 1.21098133168878E-4
196.579657965797 0.0494719292674117 1.21081151053263E-4
196.599659965997 0.0494696160575113 1.21064167299519E-4
196.619661966197 0.049467302405287 1.21047181886911E-4
196.639663966397 0.049464988307779 1.2103019479455E-4
196.659665966597 0.0494626737619813 1.21013206001214E-4
196.679667966797 0.0494603587649036 1.20996215485809E-4
196.699669966997 0.0494580433135656 1.20979223227318E-4
196.719671967197 0.0494557274049831 1.20962229204709E-4
196.739673967397 0.0494534110361406 1.20945233396723E-4
196.759675967597 0.0494510942040017 1.20928235781961E-4
196.779677967797 0.0494487769055899 1.20911236339468E-4
196.799679967997 0.0494464591378591 1.20894235047789E-4
196.819681968197 0.0494441408977685 1.20877231885514E-4
196.839683968397 0.049441822182291 1.20860226831347E-4
196.859685968597 0.0494395029883648 1.2084321986374E-4
196.879687968797 0.0494371833129642 1.20826210961422E-4
196.899689968997 0.0494348631530261 1.20809200102855E-4
196.919691969197 0.0494325425054782 1.20792187266442E-4
196.939693969397 0.0494302213672142 1.20775172430346E-4
196.959695969597 0.0494278997352027 1.20758155573287E-4
196.979697969797 0.0494255776063548 1.20741136673572E-4
196.999699969997 0.0494232549775505 1.2072411570929E-4
197.019701970197 0.0494209318457062 1.20707092658803E-4
197.039703970397 0.0494186082077066 1.20690067500251E-4

197.059705970597 0.0494162840604527 1.20673040211904E-4
197.079707970797 0.0494139594008074 1.20656010771757E-4
197.099709970997 0.0494116342256591 1.20638979158005E-4
197.119711971197 0.0494093085318632 1.20621945348607E-4
197.139713971397 0.0494069823162908 1.20604909321647E-4
197.159715971597 0.0494046555757736 1.2058787105493E-4
197.179717971797 0.0494023283071736 1.2057083052649E-4
197.199719971997 0.0494000005073351 1.20553787714241E-4
197.219721972197 0.0493976721730677 1.20536742595853E-4
197.239723972397 0.0493953433012159 1.20519695149258E-4
197.259725972597 0.0493930138885857 1.20502645352119E-4
197.279727972797 0.049390683932014 1.20485593182328E-4
197.299729972997 0.0493883534282503 1.20468538617153E-4
197.319731973197 0.0493860223741617 1.20451481634728E-4
197.339733973397 0.0493836907664679 1.20434422212116E-4
197.359735973597 0.0493813586020063 1.20417360327253E-4
197.379737973797 0.0493790258775257 1.20400295957436E-4
197.399739973997 0.049376692589804 1.20383229080183E-4
197.419741974197 0.0493743587355995 1.20366159672876E-4
197.439743974397 0.0493720243116804 1.20349087712983E-4
197.459745974597 0.0493696893147683 1.20332013177634E-4
197.479747974797 0.0493673537416251 1.2031493604427E-4
197.499749974998 0.0493650175890028 1.20297856290263E-4
197.519751975198 0.0493626808535635 1.20280773892341E-4
197.539753975398 0.0493603435321066 1.20263688828243E-4
197.559755975598 0.0493580056212816 1.20246601074621E-4
197.579757975798 0.0493556671178561 1.20229510609E-4
197.599759975998 0.0493533280184556 1.20212417407877E-4
197.619761976198 0.0493509883198351 1.20195321448703E-4
197.639763976398 0.0493486480186503 1.20178222708215E-4
197.659765976598 0.0493463071115775 1.20161121163308E-4
197.679767976798 0.0493439655952765 1.20144016790766E-4
197.699769976998 0.0493416234664576 1.20126909567751E-4
197.719771977198 0.0493392807217346 1.20109799470733E-4
197.739773977398 0.0493369373577583 1.20092686476456E-4
197.759775977598 0.0493345933711837 1.2007557056171E-4
197.779777977798 0.0493322487586522 1.2005845170319E-4
197.799779977998 0.0493299035167576 1.20041329877258E-4
197.819781978198 0.0493275576421699 1.20024205060837E-4
197.839783978398 0.049325211131463 1.20007077230163E-4
197.859785978598 0.0493228639812499 1.19989946361763E-4
197.879787978798 0.0493205161881517 1.19972812432235E-4
197.899789978998 0.0493181677487167 1.19955675417655E-4
197.919791979198 0.0493158186595836 1.19938535294768E-4

197.939793979398 0.049313468917277 1.19921392039497E-4
197.959795979598 0.049311118518401 1.19904245628353E-4
197.979797979798 0.049308767459504 1.19887096037452E-4
197.999799979998 0.0493064157371579 1.19869943243092E-4
198.019801980198 0.0493040633478926 1.19852787221272E-4
198.039803980398 0.0493017102882452 1.19835627948057E-4
198.059805980598 0.0492993565547844 1.19818465399748E-4
198.079807980798 0.0492970021439642 1.19801299551822E-4
198.099809980998 0.0492946470523674 1.19784130380706E-4
198.119811981198 0.0492922912764974 1.19766957862256E-4
198.139813981398 0.0492899348128501 1.19749781972284E-4
198.159815981598 0.0492875776579078 1.19732602686515E-4
198.179817981798 0.0492852198081694 1.19715419980801E-4
198.199819981998 0.0492828612601136 1.19698233830861E-4
198.219821982198 0.0492805020102344 1.19681044212531E-4
198.239823982398 0.049278142054983 1.19663851101351E-4
198.259825982598 0.0492757813908123 1.19646654472879E-4
198.279827982798 0.0492734200141875 1.19629454302775E-4
198.299829982998 0.04927105792155 1.19612250566535E-4
198.319831983198 0.0492686951093333 1.19595043239607E-4
198.339833983398 0.0492663315739676 1.19577832297427E-4
198.359835983598 0.0492639673118841 1.19560617715448E-4
198.379837983798 0.0492616023194952 1.19543399468995E-4
198.399839983998 0.0492592365931811 1.19526177533172E-4
198.419841984198 0.0492568701293885 1.19508951883574E-4
198.439843984398 0.0492545029244953 1.19491722495308E-4
198.459845984598 0.0492521349748684 1.19474489343409E-4
198.479847984798 0.0492497662768871 1.19457252403017E-4
198.499849984999 0.0492473968269367 1.19440011649322E-4
198.519851985199 0.0492450266213783 1.19422767057349E-4
198.539853985399 0.049242655656567 1.19405518602093E-4
198.559855985599 0.0492402839288421 1.1938826625844E-4
198.579857985799 0.0492379114345378 1.19371010001254E-4
198.599859985999 0.0492355381700152 1.19353749805601E-4
198.619861986199 0.0492331641315766 1.19336485646135E-4
198.639863986399 0.0492307893155318 1.19319217497571E-4
198.659865986599 0.0492284137181942 1.19301945334661E-4
198.679867986799 0.0492260373359006 1.19284669132342E-4
198.699869986999 0.0492236601648923 1.19267388864862E-4
198.719871987199 0.0492212822014976 1.19250104507117E-4
198.739873987399 0.0492189034419709 1.19232816033473E-4
198.759875987599 0.0492165238826024 1.19215523418571E-4
198.779877987799 0.0492141435196401 1.19198226636753E-4
198.799879987999 0.0492117623493475 1.19180925662483E-4

198.819881988199 0.0492093803680042 1.19163620470359E-4
198.839883988399 0.0492069975717771 1.19146311034165E-4
198.859885988599 0.0492046139569642 1.19128997328653E-4
198.879887988799 0.0492022295197602 1.19111679327832E-4
198.899889988999 0.0491998442564019 1.19094357006031E-4
198.919891989199 0.0491974581630793 1.19077030337246E-4
198.939893989399 0.049195071235998 1.19059699295604E-4
198.959895989599 0.0491926834713794 1.19042363855351E-4
198.979897989799 0.0491902948653775 1.19025023990259E-4
198.999899989999 0.0491879054141833 1.19007679674378E-4
199.019901990199 0.04918551511398 1.18990330881714E-4
199.039903990399 0.0491831239609013 1.18972977585925E-4
199.059905990599 0.0491807319511476 1.18955619761162E-4
199.079907990799 0.0491783390808235 1.18938257380894E-4
199.099909990999 0.0491759453461061 1.18920890419129E-4
199.119911991199 0.0491735507430887 1.18903518849275E-4
199.139913991399 0.0491711552679332 1.1888614264525E-4
199.159915991599 0.0491687589167255 1.18868761780435E-4
199.179917991799 0.0491663616855974 1.18851376228553E-4
199.199919991999 0.0491639635706394 1.18833985963038E-4
199.219921992199 0.0491615645679488 1.18816590957386E-4
199.239923992399 0.0491591646735952 1.18799191184906E-4
199.259925992599 0.0491567638836833 1.18781786619168E-4
199.279927992799 0.0491543621942541 1.18764377233298E-4
199.299929992999 0.0491519596013998 1.18746963000799E-4
199.319931993199 0.0491495561011536 1.18729543894761E-4
199.339933993399 0.0491471516895668 1.18712119888419E-4
199.359935993599 0.049144746362673 1.18694690954886E-4
199.379937993799 0.0491423401164996 1.18677257067246E-4
199.399939993999 0.0491399329470916 1.18659818198723E-4
199.419941994199 0.0491375248504362 1.18642374322131E-4
199.439943994399 0.0491351158225529 1.18624925410531E-4
199.459945994599 0.0491327058594229 1.18607471436719E-4
199.479947994799 0.0491302949570626 1.18590012373758E-4
199.499949995 0.0491278831114283 1.18572548194288E-4
199.5199519952 0.049125470318511 1.1855507887121E-4
199.5399539954 0.0491230565742961 1.18537604377401E-4
199.5599559956 0.0491206418747046 1.18520124685279E-4
199.5799579958 0.0491182262156917 1.18502639767524E-4
199.599959996 0.0491158095932094 1.18485149596806E-4
199.6199619962 0.0491133920031972 1.18467654145714E-4
199.6399639964 0.0491109734415689 1.18450153386666E-4
199.6599659966 0.0491085539042536 1.18432647292201E-4
199.6799679968 0.0491061333871297 1.18415135834505E-4

199.699969997 0.049103711886149 1.18397618986306E-4
199.7199719972 0.0491012893971752 1.18380096719707E-4
199.7399739974 0.0490988659161027 1.18362569007046E-4
199.7599759976 0.04909644143879 1.18345035820416E-4
199.7799779978 0.0490940159611404 1.18327497132241E-4
199.799979998 0.0490915894789771 1.18309952914384E-4
199.8199819982 0.0490891619881653 1.1829240313902E-4
199.8399839984 0.0490867334845606 1.18274847778269E-4
199.8599859986 0.0490843039639989 1.18257286804123E-4
199.8799879988 0.0490818734222909 1.18239720188399E-4
199.899989999 0.049079441855291 1.1822214790325E-4
199.9199919992 0.049077009258767 1.18204569920209E-4
199.9399939994 0.0490745756285679 1.18186986211412E-4
199.9599959996 0.0490721409604691 1.18169396748475E-4
199.9799979998 0.0490697052502242 1.18151801502871E-4
200 0.0490672684936662 1.18134200446656E-4
