# Supplementary material for: Protocol for measuring above-threshold ionization spectra driven by few-cycle laser pulses in frustrated tunneling ionization regime
Source: STAR Protoc. 2026 Sep 23;7(4):104845. doi: 10.1016/j.xpro.2026.104845 (PMC13632067; doi:10.1016/j.xpro.2026.104845)
Supplement: Supplementary file 3 [file mmc3.pdf]

MATLAB code for ATI spectra data analysis (related to Step9)

```
% function scan_CEP_effect
clear
clc

%%load calibration file
CaliCurve=load('D:\20231206Electron interference\TOF\calibration\cali for TOF\calicurve.txt');
PhotonEnergy=CaliCurve(:,1);%unit:eV;
Time=CaliCurve(:,2);%unit:us;
CountCoefficient=CaliCurve(:,3);
FittedPhotonEnergy=interp1(Time*1000,PhotonEnergy,([1:10048]-286)*0.1);
FittedCoefficient=interp1(Time*1000,CountCoefficient,([1:10048]-286)*0.1);

% %%%load TOF original data
TOF_CEPscan=zeros(10048,92);
  FittedCount_CEPscan=zeros(10048,92);
Count_CEPscan=zeros(10048,92);
Count_CEPscansmooth=zeros(10048,92);
  for n=1:35
        foldername=['D:\20231206Electron interference\TOF\20240114\'];
TOF=load([foldername 's' num2str(n-1) '.asc']);
  TOF_CEPscan(:,n)=TOF;
FittedPhotonEnergy=interp1(Time*1000, PhotonEnergy,([1:length(TOF)]-286)*0.1);
  FittedCoefficient=interp1(Time*1000, CountCoefficient,([1:length(TOF)]-286)*0.1);
  FittedCount=FittedCoefficient.*TOF';
  Count_CEPscan(:,n)=TOF;
  Count_CEPscansmooth(:,n)=smooth(TOF,10);
  FittedCount_CEPscan(:,n)=FittedCount;
end

%%%%%Plot figures
[X,Y]=meshgrid([1:MT],[1:10048]*0.1);
h1=figure;
mesh(X,Y,Count_CEPscan);view(90,90);shading interp;
set(gca,'FontName','Arial','FontSize',12,'FontWeight','bold');
xlabel('CEP scan');%xlim([1,32]);
ylabel('TOF (ns)');%xlim([min(x_prime*1e6),max(x_prime*1e6)])
set(h1,'unit','centimeters','position',[3 6 12 10]);


CEPs=[0:pi/17:2*pi];
[scanCEP,ElectronPhotonEnergy]=meshgrid(CEPs,FittedPhotonEnergy);
h2=figure;
```

```
mesh(scanCEP/pi,ElectronPhotonEnergy,Count_CEPscan);view(90,90);
set(gca,'FontName','Arial','FontSize',12,'FontWeight','bold');
xlabel('Relative CEP (\pi)');xlim([0,2]);
ylabel('Electron energy (eV)');%xlim([min(x_prime*1e6),max(x_prime*1e6)])
set(h2,'unit','centimeters','position',[3 6 12 10]);

h3=figure;
mesh(scanCEP/pi,ElectronPhotonEnergy,log10(FittedCount_CEPscan));view(90,90);shading
interp;
set(gca,'FontName','Arial','FontSize',12,'FontWeight','bold');
xlabel('Relative CEP (\pi)');xlim([0,2]);
ylabel('Electron energy (eV)');%xlim([min(x_prime*1e6),max(x_prime*1e6)])
set(h3,'unit','centimeters','position',[3 6 12 10]);
```
